# Design, Synthesis, Molecular Docking Analysis and Biological Evaluations of 4-[(Quinolin-4-yl)amino]benzamide Derivatives as Novel Anti-Influenza Virus Agents

**DOI:** 10.3390/ijms23116307

**Published:** 2022-06-04

**Authors:** Chao Zhang, Yun-Sang Tang, Chu-Ren Meng, Jing Xu, De-Liang Zhang, Jian Wang, Er-Fang Huang, Pang-Chui Shaw, Chun Hu

**Affiliations:** 1 Key Laboratory of Structure-Based Drug Design & Discovery, Ministry of Education, Shenyang Pharmaceutical University, Shenyang 110016, China; zhangchaoylh@126.com (C.Z.); mengchuren@163.com (C.-R.M.); xujing9998881111@163.com (J.X.); zhangdeliang1230@163.com (D.-L.Z.); jianwang@email.com (J.W.); huang222fang@163.com (E.-F.H.); 2 School of Life Sciences, The Chinese University of Hong Kong, Shatin, Hong Kong 999077, China; samtys0910@gmail.com

**Keywords:** anti-influenza virus, 4-[(quinolin-4-yl)amino]benzamide derivatives, molecular dynamics simulation, RNA-dependent RNA polymerase, pharmacophore

## Abstract

In this study, a series of 4-[(quinolin-4-yl)amino]benzamide derivatives as the novel anti-influenza agents were designed and synthesized. Cytotoxicity assay, cytopathic effect assay and plaque inhibition assay were performed to evaluate the anti-influenza virus A/WSN/33 (H1N1) activity of the target compounds. The target compound **G07** demonstrated significant anti-influenza virus A/WSN/33 (H1N1) activity both in cytopathic effect assay (EC_50_ = 11.38 ± 1.89 µM) and plaque inhibition assay (IC_50_ = 0.23 ± 0.15 µM). **G07** also exhibited significant anti-influenza virus activities against other three different influenza virus strains A/PR/8 (H1N1), A/HK/68 (H3N2) and influenza B virus. According to the result of ribonucleoprotein reconstitution assay, **G07** could interact well with ribonucleoprotein with an inhibition rate of 80.65% at 100 µM. Furthermore, **G07** exhibited significant activity target PA−PB1 subunit of RNA polymerase according to the PA−PB1 inhibitory activity prediction by the best pharmacophore Hypo1. In addition, **G07** was well drug-likeness based on the results of Lipinski’s rule and ADMET prediction. All the results proved that 4-[(quinolin-4-yl)amino]benzamide derivatives could generate potential candidates in discovery of anti-influenza virus agents.

## 1. Introduction

Influenza virus remains to be a grand challenge to public health due to the high incidence and mortality. It belongs to pathogenic microorganisms causing acute respiratory diseases [1]. The course of the influenza mainly depends on the different flu strains in addition to the host immune response. Usually, influenza viruses are classified into four types (A, B, C and D) according to their nucleoprotein (NP) and matrix protein 1 (M1). Among the four types of influenza viruses, influenza A virus was further divided into many kinds of subtypes based on their surface glycoproteins, hemagglutinin (HA) and neuraminidase (NA). The influenza A virus infected a wide range of avian and mammalian hosts, while influenza B virus infected only humans [2]. These differences increased the severity of the disease [3,4].

Currently, influenza vaccination was the preferred method for prophylaxis of influenza. However, the initial supply of the vaccines might not be sufficient to meet the demand for them [5,6] due to the rapid mutations of the virus genes and the constraints in manufacturing capacity and efficiency of pharmaceutical companies [7,8,9]. Nowadays, three classes of influenza antiviral drugs have been approved by the U.S. Food and Drug Administration (FDA): M2 ion-channel protein inhibitors (amantadine and rimantadine), neuraminidase inhibitors (oseltamivir, zanamivir and peramivir) and RNA-dependent RNA polymerase (RDRP) inhibitors (favipiravir and baloxavir) [10,11,12,13,14]. However, drug resistance of these anti-influenza virus drugs have been emerged in varying degrees [15]. Thus, there is an urgent demand for the development of new anti-influenza drugs.

Influenza virus belongs to the orthomyxoviridae family. It contains a segmented single-stranded negative sense RNA genome [16] which is encapsidated into a ribonucleoprotein (RNP) molecule. RNP plays a crucial role in the life cycle of influenza virus and the viral genome transcription and replication in infected cells. It is composed of RNA-dependent RNA polymerase (RdRp) complex (including the polymerase acidic protein (PA), polymerase basic protein 1 (PB1) and polymerase basic protein 2 (PB2)) and nucleoprotein (NP). The C-terminal region of PA (PA_C_) contacts with the N-terminal region of PB1 (PB1_N_). The C-terminal region of PB1 (PB1_C_) was involved in protein−protein interaction with the N-terminal region of PB2 (PB2_N_) [13,14]. The viral RNA polymerase is validated targets for the development of new antiviral therapies and the protein−protein interactions between PA, PB1 and PB2 make it possible to develop protein−protein interaction inhibitors.

**Figure 1:**
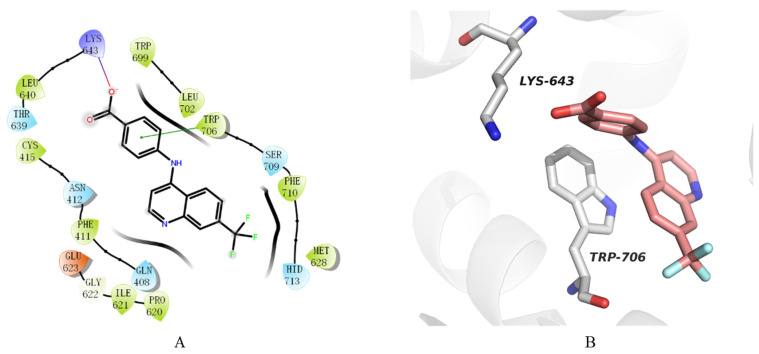
Docking model of 4-{[7-(trifluoromethyl)quinolin-4-yl]amino}benzoic acid in the active site of RNA polymerase (PDB ID: 3CM8). (**A**) The two-dimensional (2D) diagrams of the interactions between 4-{[7-(trifluoromethyl)quinolin-4-yl]amino}benzoic acid and 3CM8. The Pi-Pi stacking interactions with residues were represented by a green line segment. The salt bridge interactions with residues were represented by the red and purple line. (**B**) The three-dimensional (3D) diagrams of the interactions between 4-{[7-(trifluoromethyl)quinolin-4-yl]amino}benzoic acid and 3CM8.

**Figure 2:**
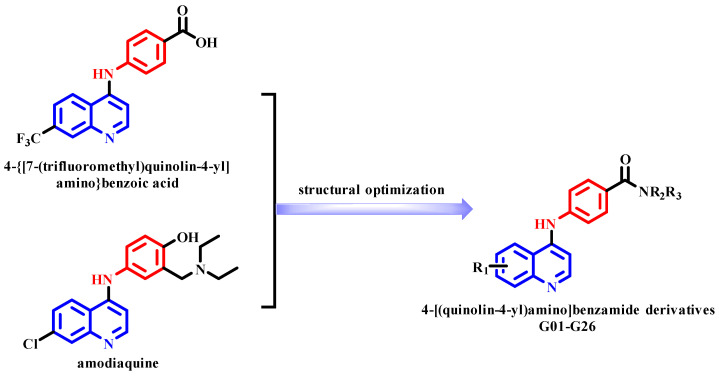
Design of 4-[(quinolin-4-yl)amino]benzamide derivatives (**G01**–**G26**).

In this study, we attempted to employ computer aided drug design to discover potential hit compounds against the RNA polymerase (PA−PB1, PDB ID: 3CM8) from our in-house compound database (2500 compounds). Firstly, the molecular docking calculations were implied using Glide program in Schrödinger software. Then, 5 compounds with docking scores less than −5.000 kcal/mol were subjected to biological activity assays [cytotoxicity assay, cytopathic effect (CPE) assay and plaque inhibition assay]. To our surprise, 4-{[7-(trifluoromethyl)quinolin-4-yl]amino}benzoic acid exhibited moderated anti-influenza virus (A/WSN/33, H1N1) activity with an EC_50_ value of 22.94 µM based on the CPE assay. Furthermore, 4-{[7-(trifluoromethyl)quinolin-4-yl] amino}benzoic acid had an inhibition rate of 50.00% at 50 µM according to plaque inhibition assay. The benzene ring and the oxygen atom in 4-aminobenzoic acid of this compound revealed pivotal interactions with the RNA polymerase (PA−PB1, PDB ID: 3CM8) according to the docking study (the benzene ring of 4-aminobenzoic acid formed Pi-Pi stacking interaction with TRP706 and the oxygen atom in carboxylic acid of 4-aminobenzoic acid generated salt bridge with LYS643) (Figure 1). We infered that 4-{[7-(trifluoromethyl)quinolin-4-yl]amino}benzoic acid could interact with PA_C_ to disrupts PA−PB1 interaction. Although 4-{[7-(trifluoromethyl)quinolin-4-yl]amino}ben- zoic acid exhibited moderate anti-influenza virus activity, the structural skeleton of 4-{[7-(trifluoromethyl)quinolin-4-yl]amino}benzoic acid was similar with available broad-spectra anti-influenza virus inhibitors amodiaquine [17] (EC_50_ = 6.3 µM; A/WSN/33, H1N1; Figure 2). In addition, quinoline derivatives were widely known as a significant group in anti-influenza virus inhibitors [17]. Therefore, taking 4-{[7-(trifluoromethyl)quinolin-4-yl]amino}benzoic acid as the lead compound, a series of new 26 4-[(quinolin-4-yl)amino]benzamide derivatives were designed (**G01**–**G26**, Figure 2) based on structural optimization. We wished to determine the impact of different modifications on anti-influenza virus activities. Herein, the synthesis and comprehensive in vitro and in silico investigations were reported about a novel series of 4-[(quinolin-4-yl)amino]benzamide derivatives.

## 2. Results and Discussion

### 2.1. Chemistry

**Scheme 1:**
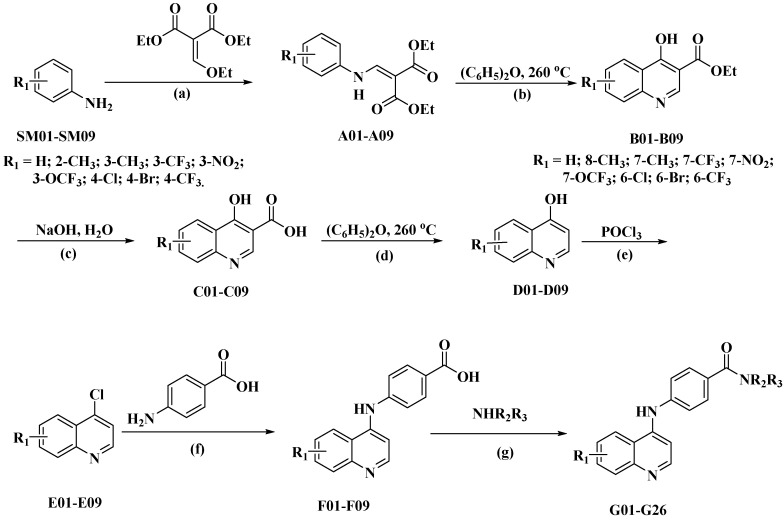
Synthetic route of 4-(quinolin-4-ylamino)benzamide derivates (**G01**–**G26**). Reagents and conditions: (**a**) Ethanol, 70 °C, 4 h; (**b**) Diphenyl ether, 260 °C, 0.8 h; (**c**) NaOH, 100 °C, 6 h; (**d**) Diphenyl ether, 260 °C, 0.8 h; (**e**) POCl_3_, 120 °C, 1 h; (**f**) Ethanol, 78 °C, 2 h; (**g**) R_2_R_3_NH, EDCI, HOBt, triethylamine, DMF, r.t., 24 h.

The synthetic route of compounds (**G01**–**G26**) was outlined in Scheme 1.

Anilines (**SM01**–**SM09**) with different substituent groups as raw materials condensed with diethyl ethoxymethylenemalonate in ethanol at reflux temperature to afford diethyl 2-[(phenylamino)methylene]malonate derivatives (**A01**–**A09**), respectively, which were heated and thermally cyclized in diphenyl ether to obtain the corresponding ethyl 4-hydroxyquinoline-3-carboxylate derivates (**B01**–**B09**). 4-hydroxyquinoline-3-carboxylic acid derivates (**C01**–**C09**) were achieved by hydrolysis. Then, quinolin-4-ol derivates (**D01**–**D09**) were obtained after decarboxylation by heating 4-hydroxyquinoline-3-carboxylic acid derivates (**C01**–**C09**) in diphenyl ether. The corresponding key intermediates 4-chloroquinoline derivates (**E01**–**E09**) were achieved by chlorination of quinolin-4-ol derivates (**D01**–**D09**) with refluxing phosphorus oxychloride [18,19,20,21,22]. Subsequently, 4-[(quinolin-4-yl)amino]benzoic acids (**F01**–**F09**) were yielded through the reaction of the key intermediates (**E01**–**E09**) with 4-aminobenzoic acid in ethanol at reflux temperature. Finally, 26 target quinoline analogues (**G01**–**G26**) were yielded through condensation between 4-[(quinolin-4-yl)amino]benzoic acid (**F01**–**F09**) and different substituted amines as illustrated in Scheme 1.

The structures of target compounds were confirmed by spectroscopic studies (MS, HRMS, ^1^H NMR and ^13^C NMR). The specfic spectrogram are available in the the Supplementary Information (Figures S9–S126).

### 2.2. Biological Activities of All the Molecules

Each compound was performed to cytotoxicity assay to determine their CC_50_ values which was responsible for 50% reduction in cell viability (CC_50_). The results of the 26 target molecules in cytotoxicity study implied that no effect had emerged on Madin-Darby canine kidney (MDCK) cells until 100 µM except for molecules **G16**, **G20** and **G26** (Table 1). 26 target molecules were carried out CPE assay to evaluated their cytopathic inhibitory effect of MDCK cells infected by influenza A virus strain (A/WSN/33, H1N1). The results were expressed as the 50% effective concentration (EC_50_) which was defined as the compound concentration required to protect 50% of the MDCK cells against viral infection. Cytopathic cells were fewer in some target molecule-treated infected cells according to the CPE results. Subsequently, they were subjected to plaque inhibition screening assay to discover potential anti-influenza virus inhibitors (Table 1 and Table 2). Furthermore, some compounds were fulfilled to RNP reconstitution assay to assess whether these compounds could interect with RNP of influenza virus.

### 2.3. Structure-Activity Relationships

The cytotoxicities of all target compounds (**G01**–**G26**) were tested. The cytopathic inhibitory effects of these target compounds were evaluated by CPE assay using influenza virus-infected MDCK cells (Table 1 and Table S1–S4). For the target compounds (**G01**–**G18**), the CC_50_ values of all these compounds were more than 100 µM as enlisted in Table 1. Based on the cytopathic effect assay, **G01**, **G02**, **G03**, **G07**, **G10** and **G11** exhibited significant cytopathic protection ability with EC_50_ values of 16.48 ± 5.57, 10.1 ± 1.66, 10.04 ± 1.90, 11.38 ± 5.64, 10.36 ± 0.22 and 7.17 ± 1.53 µM, respectively, which clarified that 4-{[7-(trifluoromethyl)quinolin-4-yl]amino}benzamide derivatives (**G01**–**G11**) with primary amines as substituent groups demonstrated better cytopathic inhibitory effects than those compounds (**G12**–**G18**) with secondary amines as substituent groups. **G01**, **G05** and **G07** demonstrated anti-influenza virus activity with inhibition rates of 81.63%, 82.86% and 95.91% (Table 1), respectively. In generally, **G07** demonstrated significant anti-influenza virus activity both in CPE assay and plaque inhibition assay. Based on the above, retention of the substituent group (-NR_2_R_3_) as 2-(2-methoxyphenoxy)ethan-1-amino and modification of the substituent group of quinoline ring resulted in target compounds *N*-[2-(2-methoxyphenoxy)ethyl]-4-(quinolin-4-ylamino)benzamide derivatives (**G19**–**G26**). **G19** and **G23** exhibited significant anti-influenza virus activity (EC_50_ values of **G19** and **G23** were less than 10 µM and inhibition rates of **G19** and **G23** at 100 µM were 82.30% and 77.88%, respectively), which implied that the positions of substituent groups in quinoline ring were essential for the anti-influenza virus activity. Unsubstituted group (**G19**) or the nitro group (**G23**) at 7-position in quinoline ring contributed a lot to the increase of anti-influenza virus activities. In addition, the relative contributions to anti-influenza virus activity of the substituent groups at 7-position in the scaffold were trifluoromethyl (95.91%) > nitro (77.88%) > methyl (11.50%). However, none of the target compounds (**G19**–**G26**) exhibited better anti-influenza virus activities than **G07**.

**Figure 3:**
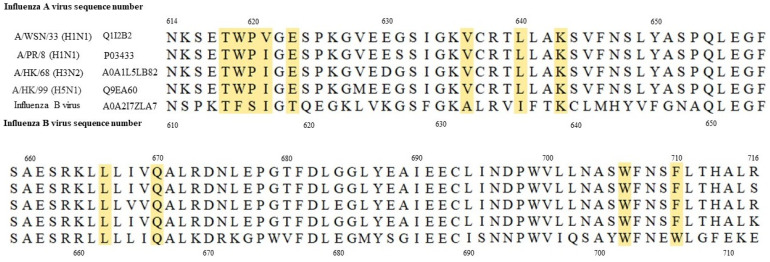
Amino acid sequence alignment of influenza virus PA protein (residues 614–716 for influenza A virus and residues 610–712 for influenza B virus) including strain A/WSN/33 (H1N1, Uniprot accession number Q1I2B2), strain A/PR/8 (H1N1, Uniprot accession number P03433), strain A/HK/68 (H3N2, Uniprot accession number A0A1L5LB82), strain A/HK/99 (H5N1, Uniprot accession number Q9EA60) and influenza B virus (Uniprot accession number A0A2I7ZLA7). Residues that are required for PA–PB1 interface are labelled with yellow.

Residues 618−621, GLU623, VAL636, LEU640, LEU666, GLN670, ARG673, TRP706, and PHE710 of influenza A virus PA protein were several reported residues on the PA−PB1 interface [23,24]. Amino sequence alignment was used to analyze the full sequences of influenza A virus PA protein and influenza B virus PA protein. The specific report was shown in the Supplementary Information (Figure S1). Amino sequence alignment of influenza A virus PA protein (residues 614−716 of H1N1, H3N2 and H5N1 subtypes) and influenza B virus PA protein (residues 610−712) have the greatest similarity. As shown in Figure 3, amino sequence alignment of influenza A virus PA protein (residues 614−716 of H1N1, H3N2 and H5N1 subtypes) and influenza B virus PA protein (residues 610−712) are highly conserved. So, we hypothesized that **G07** had antiviral potency against multiple types of influenza strains. Plaque inhibition assaies were conducted to test the antiviral activities of **G07**. The IC_50_ value of **G07** against influenza virus A/WSN/33 (H1N1) was 0.23 ± 0.15 µM (Table 2). **G07** exhibited better anti-influenza virus activity than the control inhibitor amantadine (IC_50_ > 100 µM) targeting influenza viral strain A/WSN/33 (H1N1) based on the plaque inhibition assay. In addition, **G07** exhibited significanted anti-influenza virus activities against A/PR/8 (H1N1) and A/HK/68 (H3N1) with IC_50_ values of 11.37 ± 2.38 and 7.51 ± 1.76 µM (Table 2), respectively. **G07** also demonstrated significanted anti-influenza virus activities against influenza B virus (IC_50_ = 10.99 ± 1.16 µM, Table 2). Obviously, **G07** exhibited better anti-influenza virus activities than the control compound amantadine against these three influenza virus strains A/WSN/33 (H1N1), A/PR/8 (H1N1) and influenza B virus according to the plaque inhibition assay. The IC_50_ value of amantadine against influenza virus A/HK/68 (H3N2) was 2.22 ± 0.57 µM (Table 2), which demonstrated stronger anti-influenza virus activity than **G07** against influenza virus A/HK/68 (H3N2). According to the RNP reconstitution assay results, **G07** could interect well with RNP of influenza virus with an inhibition rate of 80.65% at 100 µM.

The biological activities of all the target molecules implied that: (1) 4-{[7-(Trifluoromethyl)quinolin-4-yl]amino}benzamide derivatives (**G01**–**G11**) with primary amines as substituent groups demonstrated better cytopathic inhibitory effect than those compounds (**G12**–**G18**) with secondary amines as substituent groups. (2) 4-{[7-(trifluoromethyl)quinolin-4-yl]amino}benzamide derivatives (**G01**–**G08**) with substituted primary amines with long chains demonstrated better anti-influenza virus activity than those compounds (**G09**–**G11**) with aromatic rings or naphthenic groups. (3) The taregt compounds with electron withdrawing substituent groups in 7-position of quinoline ring exhibited anti-influenza virus activity than the taregt compounds with electron donor substituent groups. 

### 2.4. PA−PB1 Inhibitory Activity Prediction by the Best Pharmacophore Hypo1

The generated pharmacophoric model Hypo1 was used to forecasted the activity of the 26 target molecules against PA−PB1 of RNA endonuclease. The results of the cost analysis, Fischer’s randomization test and test set analysis confirm that the best pharmacophore model (Hypo1) could be selected as a significant pharmacophore model [2,25,26,27,28,29,30,31,32,33,34,35] to further predicting the biological activities of the diverse structural entity (Figures S2–S8, Tables S5–S7). The estimated values (IC_50_) of 26 target molecules were enlisted in Table 1. **G06**, **G07** and **G22** exhibited significant inhibitory activities against PA−PB1 subunit of the viral RNA with estimated values of 42.842, 35.4716 and 4.91662 µM, respectively. Four features (hydrogen bond acceptor (HBA), hydrophobic (HYD) and ring aromatic (RA)) were shown in the best pharmacophoric model Hypo1. However, the specific biological activities of these molecules targeting the PA−PB1 of RNA endonuclease need further experimental studies.

**Figure 4:**
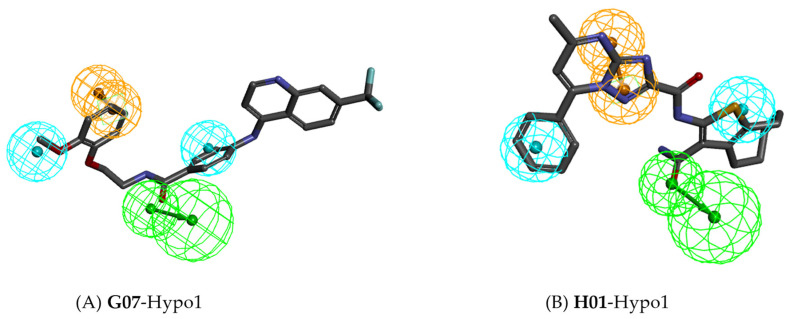
Pharmacophore mapping of the most active, less active compounds in the training set. (**A**) Hypo1 mapped on to the most active compound **G07**; (**B**) Hypo1 mapped on to the least active compound **H01**.

A typical compound **G07** and a training set compound (**H01**) [25] with significant anti-influenza virus activities were selected to analyze the pharmacophore mapping between compounds and Hypo1 (Figure 4). The benzene ring of 2-(2-methoxyphenoxy)ethan-1-amino group in **G07** mapped well with the RA feature. The methyl group in 2-(2-methoxyphenoxy)ethan-1-amino group and the benzene ring of 4-aminobenzamide in **G07** mapped well with the HYD features, respectively. Oxygen of the amide group in benzamide represented as the hydrogen acceptor and mapped well with the HBA feature. All these pharmacophore mapping results of **G07** with Hypo1 were similar to that of **H01** with Hypo1. **H01** was by far a significant anti-influenza virus agent with the best anti-influenza virus activities against PA−PB1 subunit of RNA endonuclease. The estimated activity value of **H01** was 0.982586 μM and experimental value was 1.1 μM. The estimated activity value of **G07** was 35.4716 μM. Although **G07** exhibited lower activity against PA−PB1 subunit of RNA endonuclease compared with **H01**, it still was of great advantage compared with other inhibitors targeting PA−PB1 subunit. As a corollary, **G07** can strongly interact with PA−PB1 subunit of RNA endonuclease.

### 2.5. Lipinski’s Rule and ADMET Prediction

In this study, the ADMET descriptors algorithm and toxicity prediction (extensible) module of Discovery Studio 3.0 were used to calculate the Lipinski’s rule-of-five drug-likeness properties for oral bioavailability and ADMET properties [36].

The results of Lipinski’s rule calculation for **G01**–**G26** included Alop, molecular weight (MW), number of hydrogen-bond acceptors, number of hydrogen-bond donors and number of rotatable bonds (Table S8). It was noticeable that all compounds were in line with the Lipinski’s rule, except for **G17** and **G18** (the MW of **G17** and **G18** were more than 500) [37,38].

The ADMET prediction results (including ADME Solubility Level, ADME BBB Level, ADME Absorption Level, Hepatoxic, PPB Prediction and Toxicity Probability) were enlisted in Table S9. It was worth noting that **G07** exhibited good absorption and low toxicity based on the results of ADME absorption level and toxicity probability [36]. 

In general, **G07** was of well drug-likeness according to the results of Lipinski’s rule and ADMET prediction.

### 2.6. Molecular Docking Study

To figure out the binding poses of the 4-(quinolin-4-ylamino)benzamide derivatives with the active site of RNA polymerase (PA−PB1, PDB ID: 3CM8) [39], Schrödinger’s Glide docking protocol [40,41,42,43] was performed. PAc of the PA chain was the main activity site and was conducted into the protein preparation. Based on the in vitro inhibition results, three active compounds (**G07**, **G19** and **G23**) and a control compound known as PA−PB1 endonuclease inhibitor (**H02**, *N*-(3-carbamoyl-4,5,6,7-tetrahydrobenzo[*b*]thiophen-2-yl)-5-phenyl-7-(trifluoromethyl)-[1,2,4]triazolo[1,5-a]pyrimidine-2-carboxamide) [29] were selected as the ligand examples. The docking scores of **G07**, **G19**, **G23** and **H02** were −7.370, −6.677, −6.537 and −6.852 kcal/mol, respectively (Table 3). The MM/GBSA values of **G07**, **G19**, **G23** and **H02** were −67.77, −55.10, −68.94 and −56.41 kcal/mol, respectively (Table 3).

**Figure 5:**
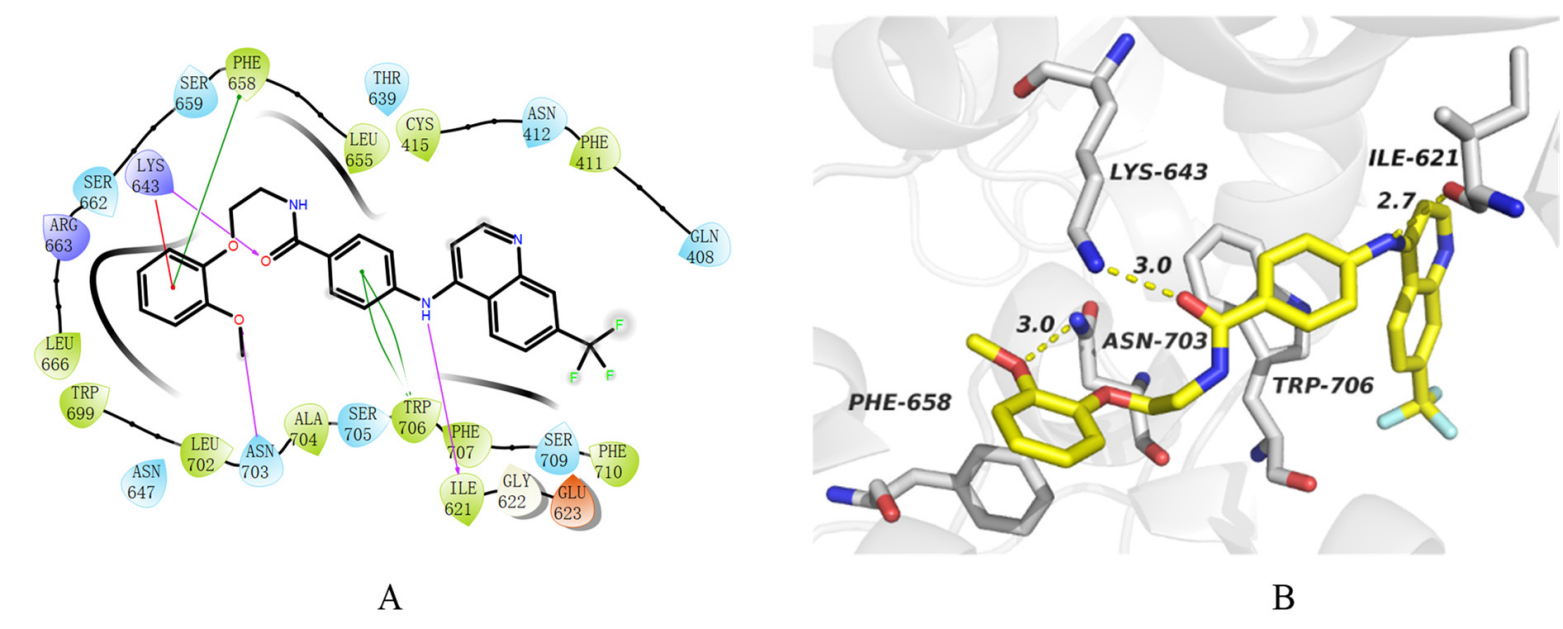
Docking model of **G07** in the active site of RNA polymerase (PDB ID: 3CM8). (**A**) Two-dimensional (2D) diagrams of **G07**-3CM8 interactions. The hydrogen-bond interactions with residues were represented by a purple dashed arrow directed towards the electron donor. The Pi-Pi stacking interactions with residues were represented by the green line segment. The Pi-cation interactions with residues were represented by the red line. (**B**) Three-dimensional (3D) diagrams of **G07**-3CM8 interactions. The hydrogen-bond interactions with residues were represented by a yellow dashed line.

**Figure 6:**
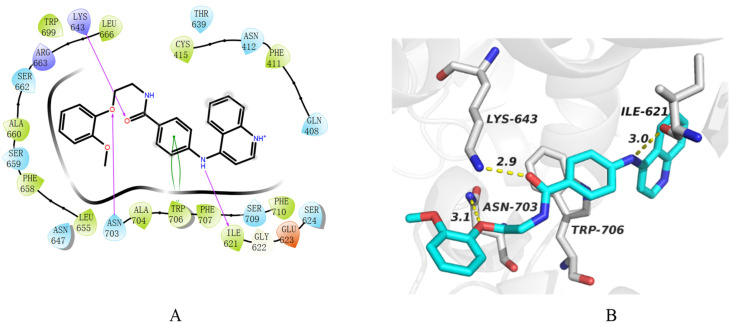
Docking model of **G19** in the active site of RNA polymerase (PDB ID: 3CM8). (**A**) Two-dimensional (2D) diagrams of **G19**-3CM8 interactions. The hydrogen-bond interactions with residues were represented by a purple dashed arrow directed towards the electron donor. The Pi-Pi stacking interactions with residues were represented by the green line segment. (**B**) Three-dimensional (3D) diagrams of **G19**-3CM8 interactions. The hydrogen-bond interactions with residues were represented by a yellow dashed line.

**Figure 7:**
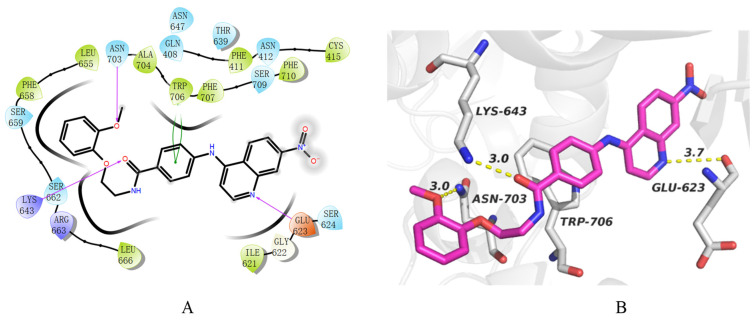
Docking model of **G23** in the active site of RNA polymerase (PDB ID: 3CM8). (**A**) The two-dimensional (2D) diagrams of the interactions between **G23** and 3CM8. The hydrogen-bond interactions with residues were represented by a purple dashed arrow directed towards the electron donor. The Pi-Pi stacking interactions with residues were represented by the green line segment. (**B**) The three-dimensional (3D) diagrams of the interactions between **G23** and 3CM8. The hydrogen-bond interactions with residues were represented by a yellow dashed line.

**Figure 8:**
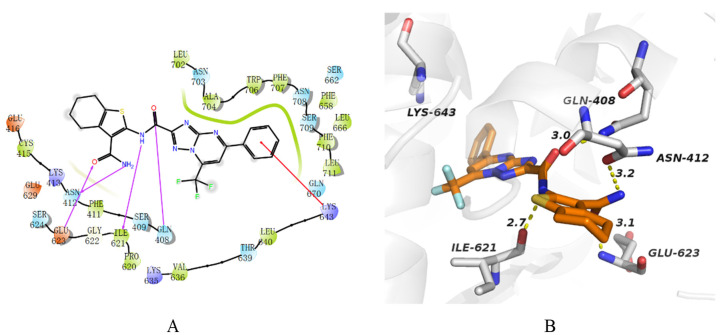
Docking model of **H02** in the active site of RNA polymerase (PDB ID: 3CM8). (**A**) The two-dimensional (2D) diagrams of the interactions between **H02** and 3CM8. The hydrogen-bond interactions with residues are represented by a purple dashed arrow directed towards the electron donor. The Pi-cation interactions with residues were represented by the red line. (**B**) The three-dimensional (3D) diagrams of the interactions between **H02** and 3CM8. The hydrogen-bond interactions with residues were represented by a yellow dashed line.

The predicted binding modes of 3CM8 with four compounds (**G07**, **G19**, **G23** and **H02**) indicated that the binding interface surrounded by these amino acid residues: GLN408-PHE411-ASN412-CYS415, ILE621-GLY622-GLU623, LEU655-PHE658-SER659- LYS643-SER662-ARG663-LEU666 and TRP699-ASN647-LEU702-ASN703-ALA704- SER705-TRP706-PHE707-SER709-PHE710 (Figure 5, Figure 6, Figure 7 and Figure 8), which was similar to the previous reported activity cavity [2,44]. Based on the docking results, it emerged that three hydrogen-bonds were essential for the interactions of compounds with amino acid residues (ILE621, LYS643 and ASN703), which seemed sufficient to impart inhibitory activity against PA−PB1 interaction [2,44]. The oxygen atoms in amide group of **H02** generated hydrogen bonds with GLU623 (3.1 Å) and GLN408 (3.0 Å). Furthermore, two hydrogen bonds were generated between the two amino groups of the control compound (**H02**) and two residues (ASN412 and ILE621) with the distance of 3.2 Å and 2.7 Å, respectively. Evidently, more hydrogen bonds were generated between the control compound (**H02**) and 3CM8 with relatively shorter distance. The interactions between the 2-(2-methoxyphenoxy)ethan-1-amine of these three compounds (**G07**, **G19** and **G23**) and the key active site of PA−PB1 were much essential. For **G07**, **G19** and **G23**, the oxygen atom of 2-(2-methoxyphenoxy)ethan-1- amino group generated hydrogen-bond with ASN703. Therefore, it is conjectured that the anti-influenza virus activity of the target compound was related to the binding mode of the 2-(2-methoxyphenoxy)ethan-1-amino group on the cavity. The amino group in **G07** (2.7 Å) and **G19** (3.0 Å) formed hydrogen-bond interaction with ILE621. Thus, it could be concluded that amino acting as a bridge connecting the benzene ring and quinoline ring played a vital role in the interaction between the active site and the target compounds. The nitrogen atom in quinoline ring of **G23** generated hydrogen-bond interaction with GLU623. The benzene ring of 4-(quinolin-4-ylamino)benzamide derivatives seemed to be essential in generating Pi-Pi stacking. It was obvious that **G07**, **G19** and **G23** built Pi-Pi stacking with TRP706. In addition, the MM/GBSA value of **G23** was lower than the MM/GBSA value of **G07**, which might imply that the interaction between GLU623 and **G23** was essential in stabilizing the complex (**G23**-3CM8).

Generally, 4-amino-*N*-[2-(2-methoxyphenoxy)ethyl]benzamide fragment contribu- ted a lot to the interaction between the active site and the target compounds. GLU623, LYS643 and TRP706 are three key amino acid residues interacted with ligands based on the docking results. We can speculate that these small molecules exhibited anti-influenza virus activities after blocking the PA−PB1 interface by competing with the PB1.

### 2.7. Molecular Dynamics Simulations

**Figure 9:**
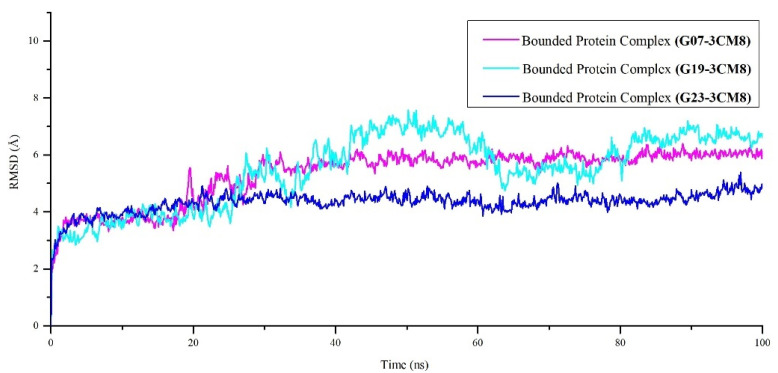
The RMSD curves of **G07**-3CM8, **G19**-3CM8 and **G23**-3CM8 during 100 ns simulations.

**Figure 10:**
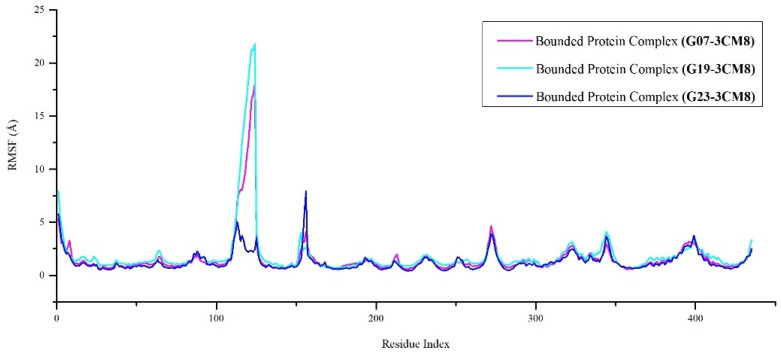
The RMSF curves of **G07**-3CM8, **G19**-3CM8 and **G23**-3CM8 during 100 ns simulations.

**Figure 11:**
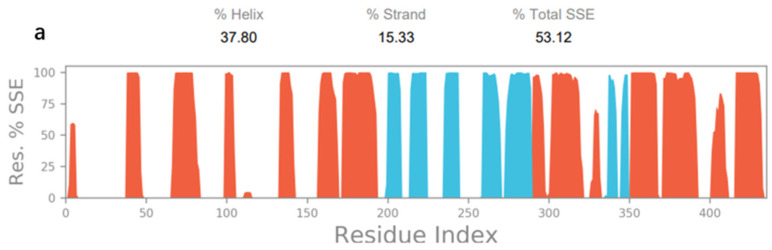

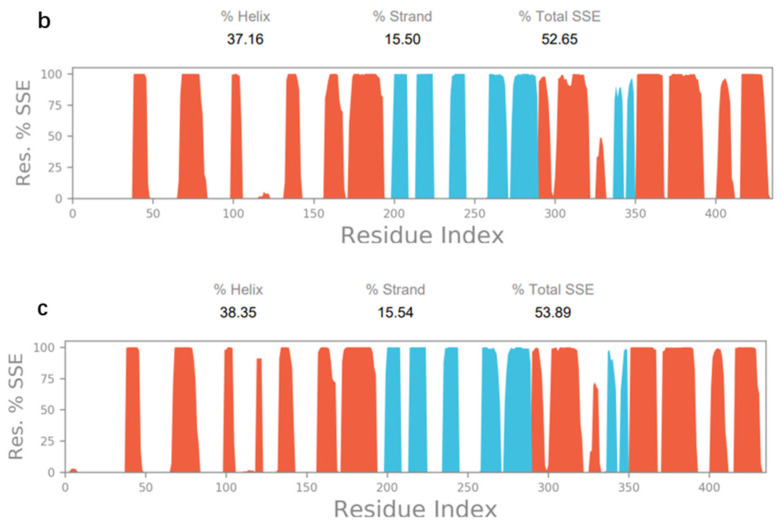
Protein secondary structure elements (SSE) were monitored throughout the simulation. The alpha-helices were labled by orange and the beta-strands were labled by blue. (**a**) Protein secondary structure elements of **G07**-3CM8 during the simulation; (**b**) Protein secondary structure elements of **G19**-3CM8 during the simulation; (**c**) Protein secondary structure elements of **G23**-3CM8 during the simulation.

The 100 ns molecular dynamics (MD) simulations were performed with three selected complexes (**G07**-3CM8, **G19**-3CM8 and **G23**-3CM8) to examine their interactions between proteins and ligands. In addition, the contribution of key residues was elucidated during the binding process. The RMSD and RMSF values of three complexes during the whole MD simulations were enlisted in Figure 9 and Figure 10. In Figure 9, the **G07**-3CM8 complex reached equilibrium after 30 ns and the protein RMSD value fluctuated around 5.5–6.0 Å. The **G23**-3CM8 complex reached equilibrium after 20 ns and the protein RMSD value fluctuated around 3.5–4.5 Å. However, **G19**-3CM8 showed obvious fluctuations and reached equilibrium after 80 ns, indicating that **G19** had poor binding affinity with 3CM8 compared with **G07** and **G23**. The RMSF plots were displayed in Figure 10. Majority fluctuations of residues were less than 2.5 Å. The states of protein secondary structures were monitored during the whole MD simulations (Figure 11). Obviously, the huge fluctuations in RMSF curves of **G07**-3CM8, **G19**-3CM8 and **G23**-3CM8 located in the non-alpha-helices and non-beta-strands according to the protein secondary structure elements (Figure 11). Among all of fluctuations in RMSF curves, the huge fluctuations of **G07**-3CM8 and **G19**-3CM8 exhibited from MET374 to PRO398, which was relatively small fluctuation of **G23**-3CM8 due to the generation of alpha-helices.

**Figure 12:**
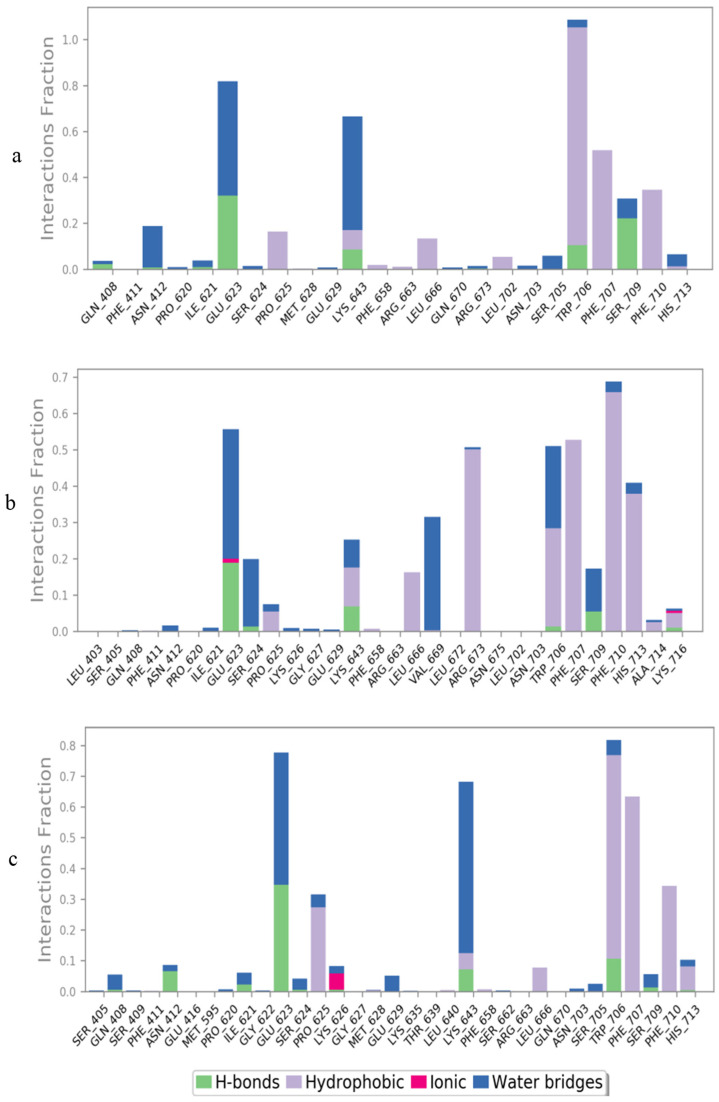
Protein-ligand contacts during the 100ns MD simulations. (**a**) Interaction fraction of complex **G07**-3CM8. (**b**) Interaction fraction of complex **G19**-3CM8. (**c**) Interaction fraction of complex **G23**-3CM8.

Figure 12 listed the type and ratio of interactions between proteins and ligands, including hydrogen bonds, hydrophobic contacts, ionic interactions, and water bridges. In Figure 12a, **G07** formed hydrogen bonds with residues GLU623, LYS643, TRP706, SER709, which accounted for 31.8%, 8.5%, 10.3% and 22.2% of the simulation time, respectively. **G07** generated water bridges with residues ASN412, GLU623 and LYS643. The hydrophobic interactions were generated between **G07** and residues PRO625, LYS643, LEU666, TRP706, PHE707 and PHE710. In Figure 12b, **G19** formed hydrogen bonds with residues ILE621, LYS643 and SER709, and generated water bridges with residues GLU623, SER624, VAL669, TRP706 and SER709. Among them, **G19** formed strong hydrogen bonds with GLU623, which accounted for 18.7% of the simulation time. In addition, water bridges were also significant for the interactions, such as **G19** formed water bridges with GLU623, SER624, VAL669, and TRP706, which accounted for 34.3%, 18.6%, 31.2% and 22.6% of the simulation time, respectively. The hydrophobic interactions were generated between **G19** and residues PRO625, LYS643, LEU666, ARG673, TRP706, PHE707, PHE710 and HIS713. In Figure 12c, **G23** formed hydrogen bonds with residues ASN412, GLU623, LYS643 and TRP706, which accounted for 6.6%, 34.7%, 7.2% and 10.6% of the simulation time, respectively. **G23** generated water bridges with residues GLN408, GLU623 and LYS643. Among them, **G23** formed strong water bridge interactions with GLU623 and LYS643, which accounted for 43.1% and 55.7% of the simulation time. The hydrophobic interactions were generated between **G23** and residues PRO625, LYS643, LEU666, TRP706, PHE707, PHE710 and HIS713.

The results of 100ns MD simulations indicated that **G07**-3CM8 and **G23**-3CM8 were more stable during MD simulations, which revealed that **G07** and **G23** might have stronger interactions with 3CM8. Generally, GLU623, LYS643, TRP706, PHE707 and PHE710 were key amino acid residues interacted with the ligands, which was similar with the previous reports [2,44].

### 2.8. Alanine Scanning Mutagenesis Analysis

Alanine scanning mutagenesis (ASM) analysis was used to assess the role of the specific amino acid residue participating in protein–protein and protein–ligand interactions. It was usually based on the hypothesis that the main chain conformation did not modify and the side chains beyond the β-carbon for ligand-protein complexes were reduced after substituting an amino acid residue into alanine. As reported, ASM analysis has been regarded as an appealing alternative to in vitro experiments [45,46]. In this study, ASM analysis was used to validate the binding free energy decomposition analysis.

**Figure 13:**
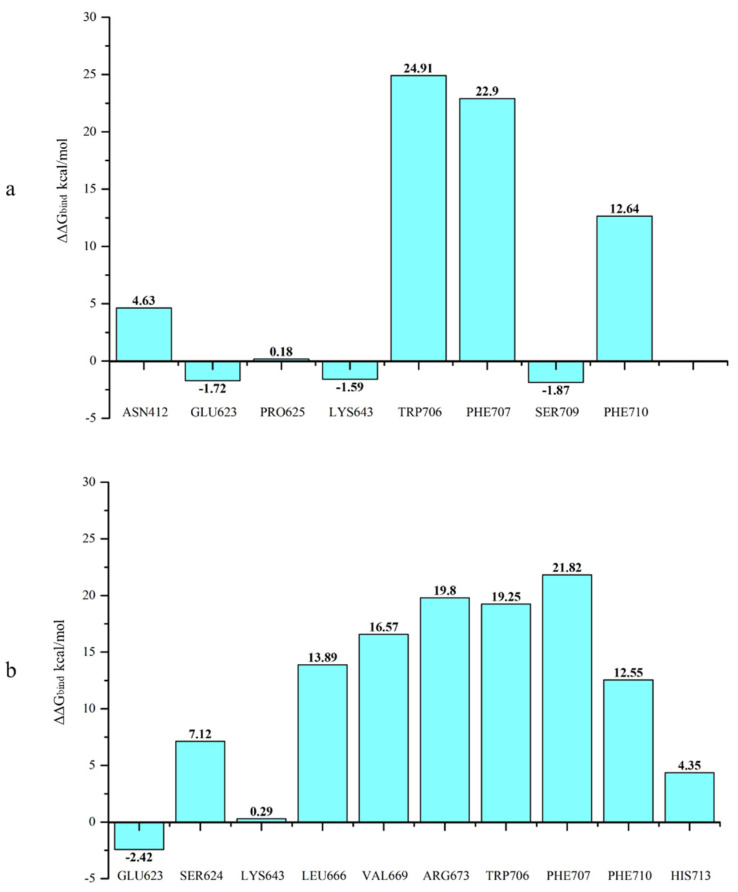

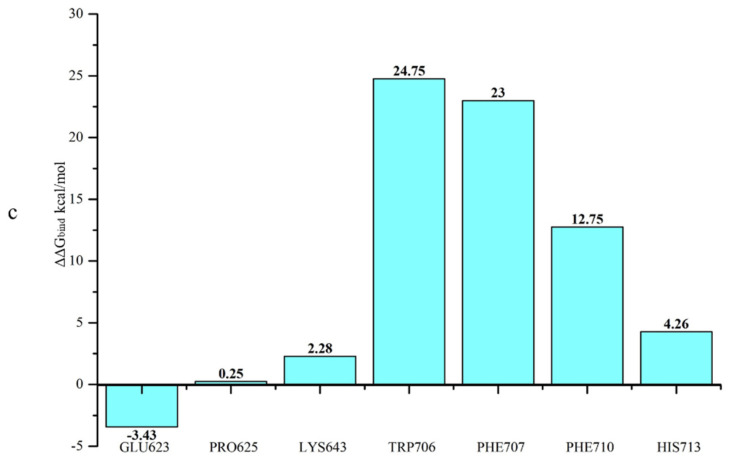
Alanine scanning mutagenesis analysis of (**a**) complex **G07**-3CM8, (**b**) complex **G19**-3CM8 and (**c**) complex **G23**-3CM8.

Residues showing high interaction fraction from MD simulations were selected to mutate to alanine (Figure 13). Positive ΔΔG values indicate that the wild-type residues could produce more favorable interactions with ligands than the mutated residues. Mutations like GLU623, PRO625, LYS643 and SER709 in complex **G07**-3CM8, mutations like GLU623 and LYS643 in complex **G19**-3CM8, and mutations like GLU623 and PRO625 in complex **G23**-3CM8 did not influence their binding behavior, indicating these residues interacted with ligands mainly through the scaffold atoms while the side chains did not contribute much to binding energy. The binding free energy changes caused by residue mutations in complex **G07**-3CM8 (residue mutations ASN412, TRP706, PHE707 and PHE710), complex **G19**-3CM8 (residue mutations SER624, LEU666, VAL669, ARG673, TRP706, PHE707, PHE710 and HIS713), as well as complex **G23**-3CM8(residue mutations TRP706, PHE707, PHE710 and HIS713) emphasized the importance of these unmutated residues. This reasonable result indicated that the mutations of the above residues into alanine shorten the length of residue side chains, and therefore decreased the interaction opportunity of residue with ligands. Interestingly, TRP706, PHE707 and PHE710 showed unexpected remarkable energy changes. It will be helpful to design more significant anti-influenza virus inhibitors through forming interactions with the side chain of TRP706, PHE707 and PHE710.

## 3. Materials and Methods

### 3.1. Synthesis

Unless otherwise required, all solvents and reagents were purchased from commercial sources and were used without further purification. All chemical reactions were monitored through GF_254_ thin layer chromatography (TLC) plate and spots were visualized by UV light (254 nm). The structures of the target compounds were characterized by ^1^H NMR spectra and ^13^C NMR spectra on a Bruker 400 MHz or 101 MHz NMR spectrometer (Faellanden, Switzerland) with TMS as an internal standard and DMSO-*d_6_* or CDCl_3_ as the solvent, chemical shifts (d values) and coupling constants (*J* values) are respectively given in ppm and Hz. The melting points were determined on a Buchi B-540 melting-point apparatus with a microscope, and were uncorrected. The IR spectra were recorded with KBr pellets on a Bruker IFS55 spectrometer (Faellanden, Switzerland). High-resolution mass (HRMS) spectral were performed on an Agilent Technologies 6530 Accurate-Mass Q-TOF Mass Spectrometer (Santa Clara, CA, USA). Compounds **A** (**A01**–**A09**), **B (B01**–**B09**), **C** (**C01**–**C09**), **D** (**D01**–**D09**) and **E** (**E01**–**E09**) [19,21,47,48,49,50,51] were previously prepared.

#### 3.1.1. General Procedure for Synthesis of 4-[(Quinolin-4-yl)amino]benzoic Acid (**F01**–**F09**)

4-Chloroquinoline (**E01**–**E09**, 0.01 mol), 4-aminobenzamide (0.011 mol), 20 mL ethanol and a catalytic amount (4 drops) of 37% hydrochloric acid were refluxed for 2 h. The reaction was allowed to cool to room temperature, and the precipitated was filtered off, washed with water (3 × 5 mL), and recrystallized by methanol [48] to afford pure 4-[(quinolin-4-yl)amino]benzoic acid (**F01**–**F09**).

4-{[7-(Trifluoromethyl)quinolin-4-yl]amino}benzoic acid (**F01**). Yellow powder. m.p.: 323.7–324.9 °C, yield in 89.35%. MS (ESI): calcd for C_17_H_11_F_3_N_2_O_2_, *m*/*z* 333.1 ([M+H]^+^), found 333.0 ([M+H]^+^). ^1^H NMR (400 MHz, DMSO-*d*_6_) δ 13.09 (s, 1H), 11.57 (s, 1H), 9.19 (d, *J* = 8.9 Hz, 1H), 8.72 (d, *J* = 6.9 Hz, 1H), 8.59 (s, 1H), 8.16–8.11 (m, 8.6 Hz, 3H), 7.67 (d, *J* = 8.2 Hz, 2H), 7.14 (d, *J* = 6.9 Hz, 1H). ^13^C NMR (101 MHz, DMSO-*d*_6_) δ 167.04, 154.74, 144.88, 141.66, 138.52, 133.45, 131.46, 129.69, 126.90, 125.07, 122.94, 120.35, 118.45, 102.38.

4-(Quinolin-4-ylamino)benzoic acid (**F02**). Yellow powder. m.p.: 315.7–317.6 °C, yield in 86.53%. MS (ESI): calcd for C_16_H_12_N_2_O_2_, *m*/*z* 265.1 ([M+H]^+^), found 265.0 ([M+H]^+^). ^1^H NMR (400 MHz, DMSO-*d*_6_) δ 14.96 (s, 1H), 11.18 (s, 1H), 8.89 (d, *J* = 8.5 Hz, 1H), 8.60 (d, *J* = 6.9 Hz, 1H), 8.16–8.04 (m, 4H), 7.86–7.82 (m, 1H), 7.66 (d, *J* = 8.4 Hz, 2H), 7.06 (d, *J* = 6.9 Hz, 1H).

4-[(8-Methylquinolin-4-yl)amino]benzoic acid (**F03**). Yellow powder. m.p.: 278.7–279.4 °C, yield in 82.47%. MS (ESI): calcd for C_17_H_14_N_2_O_2_, *m*/*z* 279.1 ([M+H]^+^), found 279.0 ([M+H]^+^). ^1^H NMR (400 MHz, DMSO-*d*_6_) δ 14.12 (s, 1H), 11.36 (s, 1H), 8.84 (d, *J* = 8.6 Hz, 1H), 8.51 (d, *J* = 6.9 Hz, 1H), 8.08 (d, *J* = 8.1 Hz, 2H), 7.86 (d, *J* = 7.2 Hz, 1H), 7.69–7.66 (m, 3H), 7.09 (d, *J* = 6.8 Hz, 1H), 2.74 (s, 3H). ^13^C NMR (101 MHz, DMSO-*d*_6_) δ 167.04, 155.24, 143.53, 142.09, 137.52, 135.09, 131.32, 129.52, 129.30, 127.21, 125.06, 122.39, 118.25, 101.26, 18.56.

4-[(7-Methylquinolin-4-yl)amino]benzoic acid (**F04**). Yellow powder. m.p.: >350 °C, yield in 81.11%. MS (ESI): calcd for C_17_H_14_N_2_O_2_, *m*/*z* 279.1 ([M+H]^+^), found 279.0 ([M+H]^+^). ^1^H NMR (400 MHz, DMSO-*d*_6_) δ 14.92 (s, 1H), 11.14 (s, 1H), 8.79 (d, *J* = 8.2 Hz, 1H), 8.53 (d, *J* = 6.8 Hz, 1H), 8.10 (d, *J* = 8.4 Hz, 2H), 7.90 (s, 1H), 7.68–7.63 (m, 2H), 7.01 (d, *J* = 6.9 Hz, 1H), 6.73 (s, 1H), 2.58 (s, 3H).

4-[(7-Nitroquinolin-4-yl)amino]benzoic acid (**F05**). Yellow powder. m.p.: 271.4–272.9 °C, yield in 90.60%. MS (ESI): calcd for C_16_H_11_N_3_O_4_, *m*/*z* 310.1 ([M+H]^+^), found 310.0 ([M+H]^+^). ^1^H NMR (400 MHz, DMSO-*d*_6_) δ 13.05 (s, 1H), 11.47 (s, 1H), 9.10 (d, *J* = 9.2 Hz, 1H), 8.98–8.97 (m, 1H), 8.77 (d, *J* = 6.9 Hz, 1H), 8.14–8.03 (m, 1H), 7.99 (d, *J* = 8.3 Hz, 1H), 7.66 (d, *J* = 8.6 Hz, 2H), 7.30 (d, *J* = 8.0 Hz, 1H), 7.17 (d, *J* = 6.9 Hz, 1H).

4-[(6-Chloroquinolin-4-yl)amino]benzoic acid (**F06**). Yellow powder. m.p.: 318.4–321.3 °C, yield in 89.10%. MS (ESI): calcd for C_16_H_11_ClN_2_O_2_, *m*/*z* 299.1 ([M+H]^+^), found 299.0 ([M+H]^+^). ^1^H NMR (400 MHz, DMSO-*d*_6_) δ 15.55 (s, 1H), 11.44 (s, 1H), 9.19 (s, 1H), 8.61 (s, 1H), 8.24 (d, *J* = 9.0 Hz, 1H), 8.10 (d, *J* = 7.9 Hz, 3H), 7.66 (d, *J* = 8.3 Hz, 2H), 7.09 (d, *J* = 6.7 Hz, 1H). ^13^C NMR (101 MHz, DMSO-*d*_6_) δ 167.04, 154.74, 144.88, 141.66, 138.52, 133.45, 133.12, 131.46, 129.69, 126.89, 125.07, 122.94, 122.27, 120.35, 118.45, 102.38.

4-[(6-Bromoquinolin-4-yl)amino]benzoic acid (**F07**). Yellow powder. m.p.: 335.2–336.9 °C, yield in 76.35%. MS (ESI): calcd for C_16_H_11_BrN_2_O_2_, *m*/*z* 343.0 ([M+H]^+^), found 343.1 ([M+H]^+^). ^1^H NMR (400 MHz, DMSO-*d*_6_) δ 14.96 (s, 1H), 11.08 (s, 1H), 9.13 (d, *J* = 2.0 Hz, 1H), 8.62 (d, *J* = 6.9 Hz, 1H), 8.20 (dd, *J*_1_ = 9.0 Hz, *J*_2_ = 2.0 Hz, 1H), 8.11 (d, *J* = 8.6 Hz, 2H), 8.07 (d, *J* = 9.0 Hz, 1H), 7.62 (d, *J* = 8.6 Hz, 2H), 7.10 (d, *J* = 6.9 Hz, 1H).

4-{[6-(Trifluoromethyl)quinolin-4-yl]amino}benzoic acid (**F08**). Yellow powder. m.p.: 313.7–314.9 °C, yield in 89.63%. MS (ESI): calcd for C_17_H_11_F_3_N_2_O_2_, *m*/*z* 333.1 ([M+H]^+^), found 333.0 ([M+H]^+^). ^1^H NMR (400 MHz, DMSO-*d*_6_) δ 11.56 (s, 1H), 9.42 (s, 1H), 8.69 (s, 1H), 8.32 (s, 2H), 8.12 (d, *J* = 8.1 Hz, 2H), 7.66 (d, *J* = 8.1 Hz, 3H), 7.13 (s, 1H). ^13^C NMR (101 MHz, DMSO-*d*_6_) δ 167.04, 154.74, 144.88, 141.66, 138.52, 133.45, 133.12, 131.46, 129.69, 126.89, 125.07, 122.94, 122.27, 120.35, 118.45, 102.38.

4-{[7-(Trifluoromethoxy)quinolin-4-yl]amino}benzoic acid (**F09**). Yellow powder. m.p.: >350 °C, yield in 87.95%. MS (ESI): calcd for C_17_H_11_F_3_N_2_O_3_, *m*/*z* 349.1 ([M+H]^+^), found 349.0 ([M+H]^+^). ^1^H NMR (400 MHz, DMSO-*d*_6_) δ 13.14 (s, 1H), 11.39 (s, 1H), 9.06 (d, *J* = 9.4 Hz, 1H), 8.65 (d, *J* = 7.0 Hz, 1H), 8.12 (d, *J* = 8.6 Hz, 3H), 7.88–7.85 (m, 1H), 7.66–7.63 (m, 2H), 7.06 (d, *J* = 7.0 Hz, 1H).

#### 3.1.2. General Procedure for Synthesis of 4-{[7-(Trifluoromethyl)quinolin-4-yl]amino}benzamide Derivatives (**G01**–**G26**)

A solution of 4-[(quinolin-4-yl)amino]benzoic acid (**F01–F09**, 2 mmol), 1-[3-(dimethylamino)propyl]-3-ethyl-carbodiimide hydrochloride (EDCI, 3 mmol), 1-hydroxybenzotriazole (HOBt, 3 mmol) and triethylamine (6 mmol) in N,N-dimethylformamide (DMF, 20 mL) was stirred at ambient temperature for 30 min. Then, a solution of 3-aminopropan-1-ol (24 mmol) in *N*,*N*-dimethylformamide (DMF 10mL) was added and the mixture was stirred at ambient temperature for 24 h. Aqueous NaHCO_3_ water (200 mL) was added and the reaction mixture was stirred for 1 h. Subsequently, the mixture was filtered to afford crude residue. The crude residue was purified through chromatography on a silica gel column by using CH_3_OH/CH_2_Cl_2_ (1/100) as eluent to yield 4-[(quinolin-4-yl)amino]benzamide derivatives (**G01**–**G26**).

*N*-Ethyl-4-{[7-(trifluoromethyl)quinolin-4-yl]amino}benzamide (**G01**). White powder. m.p.: 262.8–264.5 °C, yield in 39.00%. HRMS (ESI): calcd for C_19_H_16_F_3_N_3_O, *m*/*z* 360.12455 ([M+H]^+^), found 360.13135 ([M+H]^+^). ^1^H NMR (400 MHz, DMSO-*d*_6_) δ 9.39 (s, 1H), 8.66–8.62 (m, 2H), 8.43–8.40 (m, 1H), 8.22 (s, 1H), 7.92 (d, *J* = 8.2 Hz, 2H), 7.85–7.82 (m, 1H), 7.44 (d, *J* = 8.2 Hz, 2H), 7.23 (d, *J* = 5.3 Hz, 1H), 3.34–3.29 (m, 2H),1.14 (t, *J* = 7.2 Hz, 3H). ^13^C NMR (101 MHz, DMSO-*d*_6_) δ 165.78, 152.82, 148.44, 147.38, 143.49, 129.89, 129.02, 126.99, 124.95, 122.64, 120.96, 120.42, 104.83, 34.47, 15.33. IR: (KBr, cm^−1^) υ 3308.31, 2977.60, 2937.44, 2878.02, 1627.76, 1582.42, 1567.59, 1528.22, 1431.87, 1376.17, 1322.23, 1260.29, 1183.97, 1161.45, 1119.18, 1074.00, 920.21, 893.54, 865.65, 822.68, 757.59, 738.75, 681.32, 594.89, 497.60.

*N*-Isopropyl-4-{[7-(trifluoromethyl)quinolin-4-yl]amino}benzamide (**G02**). White powder. m.p.: 243.7–245.9 °C, yield in 14.30%. HRMS (ESI): calcd for C_20_H_18_F_3_N_3_O, *m*/*z* 374.14020 ([M+H]^+^), found 374.14688 ([M+H]^+^). ^1^H NMR (400 MHz, DMSO-*d*_6_) δ 9.39 (s, 1H), 8.66 (d, *J* = 5.3 Hz, 1H), 8.63 (d, *J* = 8.8 Hz, 1H), 8.23 (s, 1H), 8.16 (d, *J* = 7.8 Hz, 1H), 7.92 (d, *J* = 8.6 Hz, 2H), 7.85 (dd, *J*_1_ = 8.8 Hz, *J*_2_ = 1.9 Hz, 1H), 7.44 (d, *J* = 8.6 Hz, 2H), 7.23 (d, *J* = 5.3 Hz, 1H), 4.16–4.08 (m, 1H), 1.18 (d, *J* = 6.6 Hz, 6H). ^13^C NMR (101 MHz, DMSO-*d*_6_) δ 165.17, 152.92, 148.51, 147.38, 143.40, 130.03, 129.15, 127.09, 127.05, 124.95, 122.62, 120.96, 120.43, 104.79, 41.37, 22.87. IR: (KBr, cm^−1^) υ 3318.08, 2924.08, 2853.42, 1623.32, 1582.64, 1527.68, 1460.31, 1432.30, 1375.36, 1322.54, 1259.37, 1185.73, 1160.00, 1119.55, 1074.27, 920.08, 893.84, 865.45, 823.29, 759.08, 739.56, 681.47, 595.14, 505.52, 477.08.

*N*-Propyl-4-{[7-(trifluoromethyl)quinolin-4-yl]amino}benzamide (**G03**). White powder. m.p.: 242.5–243.4 °C, yield in 45.47%. HRMS (ESI): calcd for C_20_H_18_F_3_N_3_O, *m*/*z* 374.14020 ([M+H]^+^), found 374.14731 ([M+H]^+^). ^1^H NMR (400 MHz, DMSO-*d*_6_) δ 9.41 (s, 1H), 8.67–8.63 (m, 2H), 8.43–8.41 (m, 1H), 8.24 (s, 1H), 7.93 (d, *J* = 8.6 Hz, 2H), 7.85 (dd, *J*_1_ = 9.0 Hz, *J*_2_ = 2.0 Hz, 1H), 7.46 (d, *J* = 8.7 Hz, 2H), 7.25 (d, *J* = 5.3 Hz, 1H), 3.27–3.23 (m, 2H), 1.61–1.52 (m, 2H), 0.92 (t, *J* = 7.4 Hz, 3H). ^13^C NMR (101 MHz, DMSO-*d*_6_) δ 165.97, 152.90, 148.50, 147.36, 143.47, 129.90, 129.05, 127.06, 124.96, 122.65, 120.97, 120.45, 120.42, 104.84, 41.44, 22.95, 11.94. IR: (KBr, cm^−1^) υ 3307.72, 2965.99, 2874.79, 1628.12, 1582.23, 1527.63, 1431.97, 1375.85, 1322.67, 1260.19, 1184.62, 1161.30, 1121.06, 1074.19, 919.75, 893.99, 864.80, 822.59, 757.42, 738.91, 681.53, 593.05, 478.04.

*N*-(2-Hydroxyethyl)-4-{[7-(trifluoromethyl)quinolin-4-yl]amino}benzamide (**G04**). White powder. m.p.: 262.5–264.1 °C, yield in 42.67%. HRMS (ESI): calcd for C_19_H_16_F_3_N_3_O_2_, *m*/*z* 376.11946 ([M+H]^+^), found 376.12628 ([M+H]^+^). ^1^H NMR (400 MHz, DMSO-*d*_6_) δ 9.48 (s, 1H), 8.70–8.65 (m, 2H), 8.44–8.40 (m, 1H), 8.28–8.22 (m, 1H), 8.00–7.85 (m, 3H), 7.51–7.44 (m, 2H), 7.27–7.23 (m, 1H), 4.78 (s, 1H), 3.58–3.37 (m, 4H). ^13^C NMR (101 MHz, DMSO-*d*_6_) δ 166.20, 152.61, 148.15, 147.57, 143.48, 129.80, 129.13, 126.75, 125.01, 122.58, 121.03, 120.52, 104.81, 60.33, 42.64. IR: (KBr, cm^−1^) υ 3252.15, 1637.11, 1573.22, 1534.13, 1434.45, 1382.06, 1323.99, 1265.16, 1205.06, 1147.49, 1123.47, 1079.36, 1050.80, 898.67, 876.76, 826.05, 738.61, 682.47, 620.69, 476.66.

*N*-(3-Hydroxypropyl)-4-{[7-(trifluoromethyl)quinolin-4-yl]amino}benzamide (**G05**). White powder. m.p.: 249.6–250.4 °C, yield in 39.42%. HRMS (ESI): calcd for C_20_H_18_F_3_N_3_O_2_, *m*/*z* 390.13511 ([M+H]^+^), found 390.14175 ([M+H]^+^). ^1^H NMR (400 MHz, DMSO-*d*_6_) δ 9.43 (s, 1H), 8.65–8.63 (m, 2H), 8.44–8.41 (m, 1H), 8.22 (s, 1H), 7.93 (d, *J* = 8.6 Hz, 2H), 7.82 (d, *J* = 9.0 Hz, 1H), 7.45 (d, *J* = 8.6 Hz, 2H), 7.23 (d, *J* = 5.3 Hz, 1H), 4.54 (s, 1H), 3.52–3.49 (m, 2H), 3.38–3.34 (m, 2H), 1.75–1.69 (m, 2H). ^13^C NMR (101 MHz, DMSO-*d*_6_) δ 166.13, 152.73, 148.37, 147.42, 143.53, 129.82, 129.04, 126.91, 124.97, 122.63, 120.98, 120.42, 120.39, 104.82, 59.12, 37.04, 32.97. IR: (KBr, cm^−1^) υ 3286.63, 2941.49, 2866.79, 1627.10, 1533.89, 1433.70, 1382.91, 1326.38, 1262.75, 1192.02, 1155.06, 1121.21, 1080.00, 1048.33, 923.23, 901.69, 866.32, 822.06, 759.56, 681.81, 622.15, 476.95.

*N*-[3-(Diethylamino)propyl]-4-{[7-(trifluoromethyl)quinolin-4-yl]amino}benzamide (**G06**). White powder. m.p.: 216.5–219.3 °C, yield in 45.05%. HRMS (ESI): calcd for C_24_H_27_F_3_N_4_O, *m*/*z* 445.21370 ([M+H]^+^), found 445.21991 ([M+H]^+^). ^1^H NMR (400 MHz, DMSO-*d*_6_) δ 9.46 (s, 1H), 8.67–8.65 (m, 2H), 8.54–8.51 (m, 1H), 8.22 (s, 1H), 7.92 (d, *J* = 8.2 Hz, 2H), 7.81 (d, *J* = 8.8 Hz, 1H), 7.46 (d, *J* = 8.2 Hz, 2H), 7.23 (d, *J* = 5.3 Hz, 1H), 3.34–3.29 (m, 2H), 2.52–2.50 (m, 6H), 1.71–1.64 (m, 2H), 0.95 (t, *J* = 7.1 Hz, 6H). ^13^C NMR (101 MHz, DMSO-*d*_6_) δ 165.99, 152.83, 148.52, 147.32, 143.55, 129.78, 128.97, 127.03, 125.01, 122.67, 120.90, 120.33, 104.82, 50.60, 46.72, 38.40, 26.83, 11.82. IR: (KBr, cm^−1^) υ 3314.44, 2969.29, 2806.83, 1628.59, 1582.14, 1527.65, 1431.90, 1375.96, 1323.35, 1261.87, 1191.74, 1159.56, 1128.43, 1073.73, 920.08, 895.46, 864.15, 822.30, 756.93, 739.24, 682.03, 595.47, 479.03.

*N*-[2-(2-Methoxyphenoxy)ethyl]-4-{[7-(trifluoromethyl)quinolin-4-yl]amino}benzamide (**G07**). White powder. m.p.: 1997.6–199.4 °C, yield in 27.72%. HRMS (ESI): calcd for C_26_H_22_F_3_N_3_O_3_, *m*/*z* 482.16133 ([M+H]^+^), found 482.16843 ([M+H]^+^). ^1^H NMR (400 MHz, DMSO-*d*_6_) δ 9.42 (s, 1H), 8.67–8.62 (m, 3H), 8.23 (s, 1H), 7.95 (d, *J* = 8.2 Hz, 2H), 7.84 (d, *J* = 8.8 Hz, 1H), 7.46 (d, *J* = 8.2 Hz, 2H), 7.26–7.24 (m, 1H), 7.03 (s, 1H), 6.97 (d, *J* = 7.6 Hz, 1H), 6.93–6.86 (m, 2H), 4.12 (t, *J* = 6.1 Hz, 2H), 3.76 (s, 3H), 3.65–3.64 (m, 2H). ^13^C NMR (101 MHz, DMSO-*d*_6_) δ 166.37, 152.86, 149.68, 148.41, 147.28, 129.15, 124.99, 121.73, 121.23, 120.89, 120.46, 114.37, 112.90, 105.01, 67.37, 55.96. IR: (KBr, cm^−1^) υ 3309.99, 1626.49, 1583.30, 1528.38, 1505.80, 1432.59, 1376.22, 1323.49, 1253.86, 1160.85, 1120.34, 1075.22, 919.71, 897.68, 865.56, 823.68, 736.99, 682.62, 593.58, 495.55, 479.98.

*N*-(3-Morpholinopropyl)-4-{[7-(trifluoromethyl)quinolin-4-yl]amino}benzamide (**G08**). White powder. m.p.: 207.3–209.1 °C, yield in 33.48%. HRMS (ESI): calcd for C_24_H_25_F_3_N_4_O_2_, *m*/*z* 459.19296 ([M+H]^+^), found 459.19928 ([M+H]^+^). ^1^H NMR (400 MHz, DMSO-*d*_6_) δ 9.40 (s, 1H), 8.66–8.62 (m, 2H), 8.47–8.44 (m, 1H), 8.23 (s, 1H), 7.92 (d, *J* = 8.2 Hz, 2H), 7.82 (d, *J* = 8.8 Hz, 1H), 7.46 (d, *J* = 8.2 Hz, 2H), 7.24 (d, *J* = 5.3 Hz, 1H), 3.57 (t, *J* = 4.6 Hz, 4H), 3.34–3.29 (m, 2H), 2.35–2.32 (m, 6H), 1.73–1.66 (m, 2H). ^13^C NMR (101 MHz, DMSO-*d*_6_) δ 166.02, 152.85, 148.52, 147.31, 143.53, 129.81, 129.03, 127.06, 124.93, 122.64, 120.93, 120.38, 104.84, 66.66, 56.59, 53.81, 38.23, 26.50. IR: (KBr, cm^−1^) υ 3311.75, 2937.88, 2858.05, 2812.95, 1628.77, 1582.50, 1527.85, 1432.47, 1376.71, 1323.55, 1261.57, 1182.99, 1161.82, 1115.97, 1074.54, 920.09, 894.38, 863.71, 822.62, 757.33, 739.72, 682.25, 595.85.

*N*-Cyclohexyl-4-{[7-(trifluoromethyl)quinolin-4-yl]amino}benzamide (**G09**). White powder. m.p.: 236.5–237.3 °C, yield in 38.74%. HRMS (ESI): calcd for C_23_H_22_F_3_N_3_O, *m*/*z* 414.17150 ([M+H]^+^), found 414.17871 ([M+H]^+^). ^1^H NMR (400 MHz, DMSO-*d*_6_) δ 9.40 (s, 1H), 8.66–8.62 (m, 2H), 8.23 (s, 1H), 8.14 (d, *J* = 7.9 Hz, 1H), 7.91 (d, *J* = 8.8 Hz, 2H), 7.86–7.83 (m, 1H), 7.43 (d, *J* = 8.6 Hz, 2H), 7.22 (d, *J* = 5.3 Hz, 1H), 3.80–3.76 (m 1H), 1.82–1.60 (m, 6H), 1.38–1.23 (m, 4H). ^13^C NMR (101 MHz, DMSO-*d*_6_) δ 165.14, 152.88, 148.45, 147.43, 143.37, 130.08, 129.19, 127.00, 124.97, 122.60, 120.99, 120.47, 104.76, 48.75, 32.97, 25.76, 25.46. IR: (KBr, cm^−1^) υ 3219.24, 2934.11, 2856.95, 1633.01, 1582.49, 1530.64, 1379.21, 1323.67, 1260.81, 1158.83, 1125.73, 1072.30, 919.35, 892.37, 863.93, 816.92, 739.71, 683.61, 568.21, 481.12.

*N*-(4-Hydroxycyclohexyl)-4-{[7-(trifluoromethyl)quinolin-4-yl]amino}benzamide (**G10**). White powder. m.p.: 324.0–325.0 °C, yield in 41.96%. HRMS (ESI): calcd for C_23_H_22_F_3_N_3_O_2_, *m*/*z* 430.16641 ([M+H]^+^), found 430.17310 ([M+H]^+^). ^1^H NMR (400 MHz, DMSO-*d*_6_) δ 9.40 (s, 1H), 8.66–8.62 (m, 2H), 8.23 (s, 1H), 8.10 (d, *J* = 7.8 Hz, 1H), 7.90 (d, *J* = 8.4 Hz, 2H), 7.86–7.83 (m, 1H), 7.43 (d, *J* = 8.7 Hz, 2H), 7.22 (d, *J* = 5.3 Hz, 1H), 4.57 (d, *J* = 4.4 Hz, 1H), 3.78–3.69 (m, 1H), 3.33 (s, 1H), 1.88–1.81 (m, 4H), 1.43–1.21 (m, 4H). ^13^C NMR (101 MHz, DMSO-*d*_6_) δ 165.35, 152.92, 148.52, 147.39, 143.44, 129.97, 129.18, 127.08, 124.98, 122.63, 120.96, 120.46, 104.82, 68.84, 48.34, 34.76, 30.85. IR: (KBr, cm^−1^) υ 3260.93, 2934.68, 1642.46, 1529.99, 1430.77, 1379.65, 1320.12, 1261.47, 1180.96, 1152.52, 1129.93, 1081.83, 1051.36, 892.99, 837.04, 757.96, 685.43, 627.19, 476.94.

*N*-Benzyl-4-{[7-(trifluoromethyl)quinolin-4-yl]amino}benzamide (**G11**). White powder. m.p.: 263.4–265.8 °C, yield in 45.92%. HRMS (ESI): calcd for C_24_H_18_F_3_N_3_O, *m*/*z* 422.14020 ([M+H]^+^), found 422.14749 ([M+H]^+^). ^1^H NMR (400 MHz, DMSO-*d*_6_) δ 9.44 (s, 1H), 9.06–9.03 (m, 1H), 8.67–8.64 (m, 2H), 8.25 (s, 1H), 8.02 (d, *J* = 8.3 Hz, 2H), 7.83 (d, *J* = 8.8 Hz, 1H), 7.49 (d, *J* = 8.4 Hz, 2H), 7.37–7.31 (m, 4H), 7.27–7.22 (m, 2H), 4.54 (d, *J* = 5.9 Hz, 2H). ^13^C NMR (101 MHz, DMSO-*d*_6_) δ 166.14, 152.78, 148.48, 147.31, 143.83, 140.31, 129.44, 129.23, 128.69, 127.67, 127.12, 124.95, 122.70, 120.91, 120.40, 104.95, 43.11. IR: (KBr, cm^−1^) υ 3301.70, 3031.23, 2871.88, 1625.87, 1581.37, 1525.59, 1431.34, 1376.87, 1321.75, 1260.57, 1190.03, 1155.01, 1126.26, 1074.94, 921.01, 895.44, 867.80, 821.80, 751.76, 694.17, 579.52, 524.41, 501.98, 480.82.

Morpholino{4-{[7-(trifluoromethyl)quinolin-4-yl]amino}phenyl}methanone (**G12**). White powder. m.p.: 277.9–278.7 °C, yield in 43.23%. HRMS (ESI): calcd for C_21_H_18_F_3_N_3_O_2_, *m*/*z* 402.13511 ([M+H]^+^), found 402.14197 ([M+H]^+^). ^1^H NMR (400 MHz, DMSO-*d*_6_) δ 9.38 (s, 1H), 8.65–8.62 (m, 2H), 8.23 (s, 1H), 7.85 (dd, *J*_1_ = 8.9 Hz, *J*_2_ = 2.0 Hz, 1H), 7.50–7.43 (m, 4H), 7.23 (d, *J* = 5.3 Hz, 1H), 3.64–3.62 (m, 4H), 3.56–3.52 (m, 4H). ^13^C NMR (101 MHz, DMSO-*d*_6_) δ 169.28, 152.85, 148.42, 147.56, 142.20, 130.69, 130.24, 129.93, 129.25, 126.98, 124.95, 122.56, 121.48, 120.46, 120.42, 104.57, 66.60. IR: (KBr, cm^−1^) υ 3300.39, 1605.07, 1579.94, 1525.28, 1460.28, 1423.13, 1375.23, 1320.29, 1253.57, 1130.02, 1074.49, 1009.77, 914.69, 868.78, 831.15, 775.83, 754.96, 737.03, 684.25, 551.59, 475.16.

[4-(O-tolyl)piperazin-1-yl]{4-{[7-(trifluoromethyl)quinolin-4-yl]amino}phenyl}methanone (**G13**). White powder. m.p.: 194.7–195.6 °C, yield in 31.29%. HRMS (ESI): calcd for C_28_H_25_F_3_N_4_O, *m*/*z* 491.19805 ([M+H]^+^), found 491.20551 ([M+H]^+^). ^1^H NMR (400 MHz, DMSO-*d*_6_) δ 9.39 (s, 1H), 8.66–8.63 (m, 2H), 8.23 (s, 1H), 7.85 (dd, *J*_1_ = 8.9 Hz, *J*_2_ = 1.9 Hz, 1H), 7.53–7.45 (m, 4H), 7.25 (d, *J* = 5.3 Hz, 1H), 7.05 (d, *J* = 8.3 Hz, 2H), 6.88 (d, *J* = 8.6 Hz, 2H), 3.71–3.65 (m, 4H), 3.14–3.11 (m, 4H), 2.21 (s, 3H). ^13^C NMR (101 MHz, DMSO-*d*_6_) δ 169.21, 152.90, 149.17, 148.50, 147.50, 142.21, 130.91, 129.89, 129.23, 128.76, 127.03, 124.94, 122.57, 121.46, 120.43, 116.70, 104.58, 49.59, 20.52. IR: (KBr, cm^−1^) υ 3161.73, 2920.05, 2861.80, 1624.33, 1578.97, 1516.33, 1427.86, 1378.74, 1326.49, 1236.32, 1180.42, 1165.03, 1124.18, 1077.07, 1010.84, 920.53, 905.17, 871.23, 826.91, 749.08, 682.76, 568.47, 475.57.

[4-(P-tolyl)piperazin-1-yl]{4-{[7-(trifluoromethyl)quinolin-4-yl]amino}phenyl}methanone (**G14**). White powder. m.p.: 192.0–193.8 °C, yield in 34.01%. HRMS (ESI): calcd for C_28_H_25_F_3_N_4_O, *m*/*z* 491.19805 ([M+H]^+^), found 491.20471 ([M+H]^+^). ^1^H NMR (400 MHz, DMSO-*d*_6_) δ 9.38 (s, 1H), 8.66–8.63 (m, 2H), 8.23 (s, 1H), 7.85 (dd, *J*_1_ = 8.8 Hz, *J*_2_ = 2.0 Hz, 1H), 7.53–7.45 (m, 4H), 7.25 (d, *J* = 5.3 Hz, 1H), 7.05 (d, *J* = 8.1 Hz, 2H), 6.88 (d, *J* = 8.3 Hz, 2H), 3.73–3.63 (m, 4H), 3.14–3.12 (m, 4H), 2.21 (s, 3H). ^13^C NMR (101 MHz, DMSO-*d*_6_) δ 169.22, 152.92, 149.18, 148.52, 147.50, 142.22, 130.93, 129.89, 129.23, 128.76, 127.07, 124.94, 122.59, 121.47, 120.43, 120.40, 116.71, 104.60, 49.60, 20.52. IR: (KBr, cm^−1^) υ 3061.55, 2920.72, 2828.32, 1626.17, 1579.09, 1516.46, 1427.64, 1379.18, 1327.32, 1235.24, 1180.85, 1165.51, 1123.80, 1077.18, 1010.69, 920.59, 905.24, 871.15, 827.19, 749.70, 682.65, 568.45, 476.00.

{4-[4-(Trifluoromethoxy)phenyl]piperazin-1-yl}{4-{[7-(trifluoromethyl)quinolin-4-yl]amino}phenyl}methanone (**G15**). White powder. m.p.: 195.9–196.4 °C, yield in 42.67%. HRMS (ESI): calcd for C_28_H_22_F_6_N_4_O_2_, *m*/*z* 561.16470 ([M+H]^+^), found 561.17194 ([M+H]^+^). ^1^H NMR (400 MHz, DMSO-*d*_6_) δ 9.39 (s, 1H), 8.67–8.63 (m, 2H), 8.23 (d, *J* = 1.9 Hz, 1H), 7.85 (dd, *J*_1_ = 8.9 Hz, *J*_2_ = 1.9 Hz, 1H), 7.53 (d, *J* = 8.6 Hz, 2H), 7.47 (d, *J* = 8.6 Hz, 2H), 7.26–7.21 (m, 3H), 7.07–7.03 (m, 2H), 3.73–3.64 (m, 4H), 3.26–3.22 (m, 4H). ^13^C NMR (101 MHz, DMSO-*d*_6_) δ 168.23, 151.86, 149.27, 147.47, 146.48, 141.26, 140.29, 129.80, 129.22, 128.23, 126.02, 124.88, 123.92, 121.56, 121.32, 120.42, 119.41, 119.38, 116.14, 103.58, 47.82. IR: (KBr, cm^−1^) υ 3161.55, 2925.20, 1625.56, 1579.31, 1512.13, 1428.85, 1379.67, 1327.92, 1259.14, 1207.05, 1154.27, 1122.87, 1076.70, 1009.67, 920.34, 871.61, 827.07, 739.75, 611.88, 475.55.

{4-[3-(Trifluoromethyl)phenyl]piperazin-1-yl}{4-{[7-(trifluoromethyl)quinolin-4-yl]amino}phenyl}methanone (**G16**). White powder. m.p.: 217.0–218.5 °C, yield in 28.19%. HRMS (ESI): calcd for C_28_H_22_F_6_N_4_O, *m*/*z* 545.16978 ([M+H]^+^), found 545.17645 ([M+H]^+^). ^1^H NMR (400 MHz, DMSO-*d*_6_) δ 9.41 (s, 1H), 8.67–8.64 (m, 2H), 8.24 (s, 1H), 7.86 (dd, *J*_1_ = 8.9 Hz, *J*_2_ = 1.9 Hz, 1H), 7.55 (d, *J* = 8.5 Hz, 2H), 7.50–7.44 (m, 3H), 7.26 (dd, *J*_1_ = 9.6 Hz, *J*_2_ = 5.0 Hz, 3H), 7.12 (d, *J* = 7.6 Hz, 1H), 3.74–3.70 (m, 4H), 3.42–3.40 (m, 4H). ^13^C NMR (101 MHz, DMSO-*d*_6_) δ 169.30, 152.84, 151.47, 148.49, 147.50, 142.31, 130.77, 130.57, 130.47, 130.26, 129.92, 129.26, 127.03, 125.90, 124.93, 123.19, 122.58, 121.43, 120.40, 120.37, 119.62, 115.56, 111.82, 104.57, 48.33. IR: (KBr, cm^−1^) υ 1620.97, 1579.11, 1530.60, 1429.09, 1378.01, 1321.85, 1256.37, 1154.90, 1114.82, 1074.00, 1011.09, 946.43, 902.88, 870.96, 825.82, 784.66, 739.44, 682.17, 474.80.

[4-(4-Chlorophenyl)piperazin-1-yl]{4-{[7-(trifluoromethyl)quinolin-4-yl]amino}phenyl}methanone (**G17**). White powder. m.p.: 242.0–242.9 °C, yield in 27.45%. HRMS (ESI): calcd for C_27_H_22_ClF_3_N_4_O, *m*/*z* 511.14342 ([M+H]^+^), found 511.15131 ([M+H]^+^). ^1^H NMR (400 MHz, DMSO-*d*_6_) δ 9.39 (s, 1H), 8.66–8.62 (m, 2H), 8.23 (s, 1H), 7.86 (d, *J* = 6.9 Hz, 1H), 7.53–7.45 (m, 4H), 7.28–7.24 (m, 3H), 6.99 (d, *J* = 8.8 Hz, 2H), 3.70–3.66 (m, 4H), 3.22–3.19 (m, 4H). ^13^C NMR (101 MHz, DMSO-*d*_6_) δ 169.25, 152.88, 150.04, 148.45, 147.53, 142.25, 130.84, 129.25, 127.02, 124.96, 123.32, 122.58, 121.46, 120.46, 117.80, 104.60, 48.79. IR: (KBr, cm^−1^) υ 3159.21, 2828.99, 1625.71, 1579.11, 1531.13, 1496.65, 1427.24, 1378.80, 1326.80, 1234.13, 1181.05, 1164.67, 1123.67, 1076.96, 1011.72, 920.34, 871.48, 826.80, 749.93, 651.62, 518.84, 476.05.

[4-(4-Ethoxyphenyl)piperazin-1-yl]{4-{[7-(trifluoromethyl)quinolin-4-yl]amino}phenyl}methanone (**G18**)**.** White powder. m.p.: 210.0–212.3 °C, yield in 21.79%. HRMS (ESI): calcd for C_29_H_27_F_3_N_4_O_2_, *m*/*z* 521.20861 ([M+H]^+^), found 521.21588 ([M+H]^+^). ^1^H NMR (400 MHz, DMSO-*d*_6_) δ 9.39 (s, 1H), 8.66–8.63 (m, 2H), 8.23 (s, 1H), 7.85 (dd, *J*_1_ = 8.9 Hz, *J*_2_ = 2.0 Hz, 1H), 7.53–7.45 (m, 4H), 7.24 (d, *J* = 5.3 Hz, 1H), 6.92 (d, *J* = 8.9 Hz, 2H), 6.83 (d, *J* = 8.9 Hz, 2H), 3.97–3.92 (m, 2H), 3.71–3.65 (m, 4H), 3.07–3.04 (m, 4H), 1.29 (t, *J* = 7.0 Hz, 3H). ^13^C NMR (101 MHz, DMSO-*d*_6_) δ 169.19, 153.03, 152.86, 148.43, 147.56, 145.49, 142.19, 130.96, 129.22, 127.00, 124.95, 122.56, 121.49, 120.47, 120.42, 118.52, 115.35, 104.56, 63.56, 50.53, 15.23. IR: (KBr, cm^−1^) υ 3157.07, 2977.56, 2820.65, 1630.27, 1579.43, 1511.51, 1425.29, 1377.65, 1325.07, 1246.01, 1164.49, 1123.51, 1076.52, 1046.55, 1012.80, 920.37, 871.12, 824.80, 739.39, 682.44, 474.20.

*N*-{2-(2-Methoxyphenoxy)ethyl}-4-(quinolin-4-ylamino)benzamide (**G19**). White powder. m.p.: 319.5–320.2 °C, yield in 43.58%. HRMS (ESI): calcd for C_25_H_23_N_3_O_3_, *m*/*z* 414.17394 ([M+H]^+^), found 414.17886 ([M+H]^+^). ^1^H NMR (400 MHz, DMSO-*d*_6_) δ 9.18 (s, 1H), 8.62–8.59 (m, 1H), 8.55 (d, *J* = 5.2 Hz, 1H), 8.36 (d, *J* = 8.3 Hz, 1H), 7.93–7.89 (m, 3H), 7.75–7.70 (m, 1H), 7.58–7.54 (m, 1H), 7.42 (d, *J* = 8.7 Hz, 2H), 7.16 (d, *J* = 5.2 Hz, 1H), 7.03 (dd, *J*_1_ = 7.3 Hz, *J*_2_ = 2.2 Hz, 1H), 6.97 (dd, *J*_1_ = 7.6 Hz, *J*_2_ = 2.1 Hz, 1H), 6.93–6.86 (m, 2H), 4.10 (t, *J* = 6.1 Hz, 2H), 3.75 (s, 3H), 3.65–3.60 (m, 2H). ^13^C NMR (101 MHz, DMSO-*d*_6_) δ 166.46, 151.20, 149.71, 149.52, 148.43, 146.87, 144.44, 129.86, 129.74, 129.10, 128.66, 125.42, 122.73, 121.76, 121.27, 120.83, 120.26, 114.41, 112.94, 103.99, 67.40, 56.00, 39.44.

*N*-[2-(2-Methoxyphenoxy)ethyl]-4-[(8-methylquinolin-4-yl)amino]benzamide (**G20**). White powder. m.p.: 150.2–151.7 °C, yield in 35.91%. HRMS (ESI): calcd for C_26_H_25_N_3_O_3_, *m*/*z* 428.18959 ([M+H]^+^), found 428.19495 ([M+H]^+^). ^1^H NMR (400 MHz, DMSO-*d*_6_) δ 9.11 (s, 1H), 8.60–8.58 (m, 2H), 8.19 (d, *J* = 8.5 Hz, 1H), 7.91–7.88 (m, 2H), 7.59 (d, *J* = 6.9 Hz, 1H), 7.46–7.39 (m, 3H), 7.18 (d, *J* = 5.1 Hz, 1H), 7.03 (dd, *J*_1_ = 7.4 Hz, *J*_2_ = 2.2 Hz, 1H), 6.97 (dd, *J*_1_ = 7.6 Hz, *J*_2_ = 2.1 Hz, 1H), 6.93–6.86 (m, 2H), 4.10 (t, *J* = 6.0 Hz, 2H), 3.75 (s, 3H), 3.65–3.60 (m, 2H), 2.69 (s, 3H). ^13^C NMR (101 MHz, DMSO-*d*_6_) δ 165.39, 148.98, 148.63, 147.39, 145.99, 143.59, 135.98, 128.89, 128.02, 123.93, 120.68, 120.19, 119.66, 119.53, 118.93, 113.32, 111.86, 103.20, 66.33, 54.92, 38.36, 17.77.

*N*-[2-(2-Methoxyphenoxy)ethyl]-4-[(7-methylquinolin-4-yl)amino]benzamide (**G21**). White powder. m.p.: 209.6–210.6 °C, yield in 28.10%. HRMS (ESI): calcd for C_26_H_25_N_3_O_3_, *m*/*z* 428.18959 ([M+H]^+^), found 428.19495 ([M+H]^+^); ^1^H NMR (400 MHz, DMSO-*d*_6_) δ 9.13 (s, 1H), 8.59 (d, *J* = 5.5 Hz, 1H), 8.50 (d, *J* = 5.2 Hz, 1H), 8.25 (d, *J* = 8.6 Hz, 1H), 7.90 (d, *J* = 8.7 Hz, 2H), 7.70 (s, 1H), 7.41 (d, *J* = 8.6 Hz, 3H), 7.10 (d, *J* = 5.2 Hz, 1H), 7.03 (dd, *J*_1_ = 7.4 Hz, *J*_2_ = 2.2 Hz, 1H), 6.97 (dd, *J*_1_ = 7.6 Hz, *J*_2_ = 2.1 Hz, 1H), 6.93–6.85 (m, 2H), 4.10 (t, *J* = 6.1 Hz, 2H), 3.75 (s, 3H), 3.65–3.60 (m, 2H), 2.50 (s, 3H). ^13^C NMR (101 MHz, DMSO-*d*_6_) δ 166.47, 151.16, 149.80, 149.70, 148.43, 146.66, 144.54, 139.55, 129.09, 128.77, 128.48, 127.42, 122.54, 121.75, 121.26, 120.07, 118.79, 114.39, 112.92, 103.53, 67.39, 55.99, 39.44, 21.62.

*N*-[2-(2-Methoxyphenoxy)ethyl]-4-{[7-(trifluoromethoxy)quinolin-4-yl]amino}benzamide (**G22**). White powder. m.p.: 229.5–230.7 °C, yield in 40.24%. HRMS (ESI): calcd for C_26_H_22_F_3_N_3_O_4_, *m*/*z* 498.15624 ([M+H]^+^), found 498.16077 ([M+H]^+^). ^1^H NMR (400 MHz, DMSO-*d*_6_) δ 9.36 (s, 1H), 8.64–8.61 (m, 1H), 8.58 (d, *J* = 5.3 Hz, 1H), 8.53 (d, *J* = 9.3 Hz, 1H), 7.94–7.91 (m, 2H), 7.79 (d, *J* = 1.3 Hz, 1H), 7.57 (dd, *J*_1_ = 9.0 Hz, *J*_2_ =2.6 Hz, 1H), 7.45–7.41 (m, 2H), 7.16 (d, *J* = 5.3 Hz, 1H), 7.03 (dd, *J*_1_ = 7.4 Hz, *J*_2_ = 2.2 Hz, 1H), 6.97 (dd, *J*_1_ = 7.6 Hz, *J*_2_ = 2.1 Hz, 1H), 6.94–6.86 (m, 2H), 4.11 (t, *J* = 6.1 Hz, 2H), 3.75 (s, 3H), 3.65–3.61 (m, 2H). ^13^C NMR (101 MHz, DMSO-*d*_6_) δ 165.35, 151.74, 148.23, 148.21, 147.37, 146.41, 143.00, 128.09, 124.72, 120.70, 120.20, 119.78, 118.48, 117.72, 113.34, 111.87, 111.84, 102.95, 66.33, 54.93, 38.40.

*N*-[2-(2-Methoxyphenoxy)ethyl]-4-[(7-nitroquinolin-4-yl)amino]benzamide (**G23**). White powder. m.p.: 217.0–218.5 °C, yield in 40.76%. HRMS (ESI): calcd for C_25_H_22_N_4_O_5_, *m*/*z* 459.15902 ([M+H]^+^), found 459.16367 ([M+H]^+^). ^1^H NMR (400 MHz, DMSO-*d*_6_) δ 9.50 (s, 1H), 8.70 (d, *J* = 5.3 Hz, 1H), 8.68–8.63 (m, 3H), 8.30 (dd, *J*_1_ = 9.3 Hz, *J*_2_ = 2.5 Hz, 1H), 7.94 (d, *J* = 8.6 Hz, 2H), 7.46 (d, *J* = 8.4 Hz, 2H), 7.28 (d, *J* = 5.4 Hz, 1H), 7.03 (dd, *J*_1_ = 7.4 Hz, *J*_2_ = 2.2 Hz, 1H), 6.97 (dd, *J*_1_ = 7.6 Hz, *J*_2_ = 2.1 Hz, 1H), 6.93–6.86 (m, 2H), 4.11 (t, *J* = 6.1 Hz, 2H), 3.75 (s, 3H), 3.66–3.61 (m, 2H). ^13^C NMR (101 MHz, DMSO-*d*_6_) δ 166.37, 153.74, 149.72, 148.58, 148.43, 148.24, 147.46, 143.52, 129.64, 129.19, 125.34, 125.17, 124.26, 121.77, 121.26, 121.09, 118.31, 114.43, 112.95, 105.57, 67.40, 56.00, 39.48.

*N*-[2-(2-Methoxyphenoxy)ethyl]-4-[(6-bromoquinolin-4-yl)amino]benzamide (**G24**). White powder. m.p.: 240.5–241.3 °C, yield in 27.16%. HRMS (ESI): calcd for C_25_H_22_BrN_3_O_3_, *m*/*z* 492.08445 ([M+H]^+^), found 492.08942 ([M+H]^+^). ^1^H NMR (400 MHz, DMSO-*d*_6_) δ 9.27 (s, 1H), 8.66 (s, 1H), 8.64–8.61 (m, 1H), 8.57 (d, *J* = 5.3 Hz, 1H), 8.92 (d, *J* = 8.7 Hz, 2H), 7.85 (s, 2H), 7.43 (d, *J* = 8.5 Hz, 2H), 7.18 (d, *J* = 5.3 Hz, 1H), 7.04 (dd, *J*_1_ = 7.4 Hz, *J*_2_ = 2.2 Hz, 1H), 6.98 (dd, *J*_1_ = 7.6 Hz, *J*_2_ = 2.1 Hz, 1H), 6.94–6.86 (m, 2H), 4.11 (t, *J* = 6.1 Hz, 2H), 3.75 (s, 3H), 3.65–3.61 (m, 2H). ^13^C NMR (101 MHz, DMSO-*d*_6_) δ 166.42, 151.70, 149.72, 148.43, 148.10, 146.36, 143.94, 132.93, 131.90, 129.14, 129.10, 125.11, 122.02, 121.77, 121.26, 120.56, 118.55, 114.42, 112.94, 104.25, 67.40, 56.00, 39.46.

*N*-[2-(2-Methoxyphenoxy)ethyl]-4-[(6-chloroquinolin-4-yl)amino]benzamide (**G25**). White powder. m.p.: 247.7–248.4 °C, yield in 34.30%. HRMS (ESI): calcd for C_25_H_22_ClN_3_O_3_, *m*/*z* 448.13497 ([M+H]^+^), found 448.13965 ([M+H]^+^). ^1^H NMR (400 MHz, DMSO-*d*_6_) δ 9.25 (s, 1H), 6.64–8.61 (m, 1H), 8.56 (d, *J* = 5.3 Hz, 1H), 8.52–8.51(m, 1H), 7.94–7.91 (m, 3H), 7.74 (dd, *J*_1_ = 9.0 Hz, *J*_2_ = 2.3 Hz, 1H), 7.43 (d, *J* = 8.4 Hz, 2H), 7.18 (d, *J* = 5.3 Hz, 1H), 7.04 (dd, *J*_1_ = 7.4 Hz, *J*_2_ = 2.2 Hz, 1H), 6.98 (dd, *J*_1_ = 7.6 Hz, *J*_2_ = 2.1 Hz, 1H), 6.94–6.86 (m, 2H), 4.11 (t, *J* = 6.1 Hz, 2H), 3.75 (s, 3H), 3.65–3.61 (m, 2H). ^13^C NMR (101 MHz, DMSO-*d*_6_) δ 166.42, 151.69, 149.73, 148.45, 148.01, 146.41, 143.96, 131.88, 130.32, 130.08, 129.14, 129.07, 121.91, 121.77, 121.50, 121.27, 120.52, 114.45, 112.97, 104.28, 67.42, 56.01, 39.47.

*N*-[2-(2-Methoxyphenoxy)ethyl]-4-{[6-(trifluoromethyl)quinolin-4-yl]amino}benzamide (**G26**). White powder. m.p.: 225.3–227.3 °C, yield in 31.88%. HRMS (ESI): calcd for C_26_H_22_F_3_N_3_O_3_, *m*/*z* 482.16133 ([M+H]^+^), found 482.16592 ([M+H]^+^). ^1^H NMR (400 MHz, DMSO-*d*_6_) δ 9.53 (s, 1H), 8.89 (s, 1H), 8.67–8.64 (m, 2H), 8.08 (d, *J* = 8.8 Hz, 1H), 7.96 (dd, *J*_1_ = 8.9 Hz, *J*_2_ = 2.3 Hz, 3H), 7.46 (d, *J* = 8.6 Hz, 2H), 7.20 (d, *J* = 5.4 Hz, 1H), 7.04 (dd, *J*_1_ = 7.3 Hz, *J*_2_ = 2.2 Hz, 1H), 6.98 (dd, *J*_1_ = 7.5 Hz, *J*_2_ = 2.1 Hz, 1H), 6.94–6.86 (m, 2H), 4.12 (t, *J* = 6.0 Hz, 2H), 3.76 (s, 3H), 3.67–3.63 (m, 2H). ^13^C NMR (101 MHz, DMSO-*d*_6_) δ 166.41, 153.47, 150.72, 149.69, 148.42, 143.82, 131.13, 129.49, 126.33, 125.54, 123.62, 121.75, 119.80, 114.37, 112.90, 104.23, 67.37, 55.97, 39.47.

### 3.2. Biology

#### 3.2.1. Cell and Compounds

The MDCK cells and HEK293T cells were routinely cultured in minimum essential medium (MEM, Gibco) supplemented with 10% (*v*/*v*) fetal bovine serum (FBS, Gibco) at 37 °C in a humidified 5% CO_2_ incubator. The 26 compounds which were dissolved in dimethyl sulfoxide (DMSO) during the cytotoxicity assay, CPE assay, anti-influenza virus assay and RNP reconstitution assay.

#### 3.2.2. Cytotoxicity Assay

The 3-(4,5-dimethylthiazol-2-yl)-2,5-diphenyltetrazolium bromide (MTT) method was performed to assess the cytotoxicity of target compounds in MDCK cells and HEK293T cells [27,52,53]. Firstly, MDCK cells or HEK293T cells were seeded into the 96-well plate. Then, the media containing test compounds replaced the growth media after 48 h. Incubate MDCK cells or HEK293T cells with test compounds for 72 h at 37 °C in a humidified 5% CO_2_ incubator. Later, MTT solution (5 mg/mL in PBS) was added into each well and plates were incubated for 4 h at 37 °C. Then, add a solubilization solution to lyse cells. Finally, absorbance was read at 620 nm using an ELISA plate reader (Tecan Sunrise) after 3 h of further incubation at 37 °C. We set the values obtained from the wells treated with only DMSO as 100% of viable cells. The 50%-cytotoxic concentrations (CC_50_) were gained by a non-linear least-squares fit in the software GraphPad Prism 7.

#### 3.2.3. CPE Assay

The target compounds were assessed for their abilities in inhibiting influenza virus replication in MDCK cells by CPE reduction assay [54,55,56,57]. MDCK cells were seeded into a 96-well plate and were infected with virus at an MOI of 50 CCID_50_ (50% cell culture infective dose) per well. The target compounds were added in serial dilutions. Then, replace the growth media by Dulbecco’s Modified Eagle’s Medium (DMEM) supplemented with 0.2 μg/mL of TPCK-treated trypsin. Microscopy was performed to score virus-induced CPE after 72 h incubation at 35 °C. The concentration of test compound that protected half of the cells (EC_50_ values) was calculated using a non-linear least-squares fit in the software GraphPad Prism 7. It is worth noting that cases where the culture was protected less than 50% before cytotoxicity became dominant as inhibitor concentrations were increased were designated “No effect”.

#### 3.2.4. Plaque Inhibition Assay

The anti-influenza virus activities of the selected compounds were evaluated by plaque inhibition assay. MDCK cells were seeded at 5000 cells/well on 96-well plates for a day before being infected with the influenza virus (A/WSN/33, H1N1). The infection medium was DMEM (High Glucose) containing 1% FBS and 0.2% trypsin (1 µg/mL) [58]. The selected compounds were added to the cell culture at 100 μM. Unless otherwise indicated, the MDCK cells were infected with influenza A virus at a multiplicity of infection (MOI) of 0.1. We added Promega CellTiter-Glo^®^ reagen to each well following the protocol provided by the supplier after 45 h of incubation. Molecular Device SpectraMax M2 plate reader was utilized to quantified the luminescence (RLU) emitted from each well. The concentration required to inhibit 50% (IC_50_ values) of A/WSN/33 was calculated using the software GraphPad Prism 7.

#### 3.2.5. RNP Reconstitution Assay

As previously reported [59], plasmids pcDNA-PB1, pcDNA-PB2, pcDNA-PA, pcDNA-NP, pEGFP and pPolI-Luc-RT were used in RNP reconstitution assay. 1 × 10^5^ HEK293T cells were cultured in 96-well plates overnight. 125 ng of pcDNA-PB1, pcDNA-PB2, pcDNA-PA, pcDNA-NP, pPolI-Luc-RT and pEGFP were co-transfected to HEK293T cells to reconstitute the RNP complexes with Lipofectamine 2000 (Invitrogen, Carlsbad, CA, USA). Then, growth medium with selected compounds were added after 6 h of transfection. 24 h later, the luciferase activity was assayed by Steady-Glo luciferase substrate (Promega, Beijing, China). We used VICTOR 3 Multilabel plate reader (Perkin Elmer, Waltham, MA, USA) to read the fluorescence from GFP expression and luminescence.

### 3.3. PA−PB1 Inhibitory Activity Prediction by the Best Pharmacophore Hypo1

Firstly, 27 active and moderately active compounds [2,25,26,27,28,29,30,31] were selected as training set compounds to generate a pharmacophore model [32,33,34,35] by Hypogen algorithm 3D-QSAR pharmacophore generation protocol. The best pharmacophore Hypo1 enlisted in Table S5 was characterized with lowest total cost value (94.2536), the highest cost difference (75.318), the lowest RMSD (0.973343), and the best correlation coefficient (0.937771). Then, cost analysis, Fischer’s randomization test and test set analysis were used to validate the best pharmacophore model (Hypo1). The specific steps of pharmacophore model generation and validation were shown in the supporting information (Figures S2–S8, Tables S5–S7). The target compounds were all screened by the best pharmacophore Hypo1 and the estimated values were enlisted in Table 1.

### 3.4. Lipinski’s Rule and ADMET Prediction

A major filtration criterion for the drug design process was the prediction of adsorption, distribution, metabolism, excretion and toxicity (ADMET) properties. In this study, the ADMET modules in Discovery Studio 3.0 were used to calculate various mathematical predictive ADMET pharmacokinetic parameters, such as blood-brain-barrier penetration, human intestinal absorption, aqueous solubility, hepatotoxicity, plasma protein binding. Then, the 26 compounds were subjected to toxicity screening models using TOPKAT module of Discovery Studio 3.0.

### 3.5. Molecular Docking

Schrödinger’s Glide docking protocol was performed to the virtual screening and study the interactions of the selected target compounds with the crystal structure (PDB ID: 3CM8) [40,41,42,43] which was retrieved from the protein data base (RCSB PDB, http://www.rcsb.org, accessed on 12 August 2021). Then, Schrödinger’s protein preparation wizards were used to prepare the protein (remove the cofactors and water molecules; add missing residues; add hydrogens; generate Het states, and optimize the selected protein) [42]. Subsequently, the prepared protein structure was further processed for generating grid [43]. The active sites were defined based on the key residues (Asn412, Gln408, Glu623, Trp706, Trp618, Ile621, Lys643, Arg673, and Gln670) of in-bound ligand according to the previous literature sources [2,39,44]. Finally, the selected target compounds were prepared to implement the molecular docking by Glide-XP (extra precision). The docking poses were visually analyzed.

### 3.6. Preparation for Molecular Dynamics Simulation

The 100 ns MD simulations were performed by Desmond v3.8 module in the Schrödinger suite (version 9.6, Schrödinger Inc., New York, NY, USA). Three complexes (**G07**-3CM8, **G19**-3CM8 and **G22**-3CM8) were carried out for 100 ns MD simulations. The system was solvated with SPC water and neutralized by adding an appropriate amount of counter ions in the orthorhombic box (10 Å × 10 Å × 10 Å) in order to generate a buffer area between the protein atoms and the side of the box. Then, OPLS_2005 force field was used to minimize the energy of the complex system. The maximum number of iterations was set as 5000 during the minimization process. The temperature was set to 300 K and the pressure was set to 1.01325 bar. Finally, the 100 ns MD simulations were carried out (record the time interval of each trajectory at every 100 PS) [60,61,62].

The simulation quality analysis tool was used to analysis the MD simulations. The quality of MD simulations was predicted by the simulation event analysis tool. The protein-ligand interactions were identified through the simulation interaction diagram tool.

### 3.7. Prime/MM–GBSA Simulation

The molecular mechanics generalized born surface area (MM-GBSA) method was used to calculate the binding-free energy (ΔG_bind_) of each ligand according to the following equation [63]:

ΔG_bind_ = ΔE_MM_ + ΔG_solv_ + ΔG_SA_

In the equation, ΔE_MM_ was the difference in the minimized energies between the ligand-protein complexes and the sum of the energies of the protein and ligand in the unbound state. ΔG_solv_ was the difference in the GBSA solvation energy of the ligand-protein complexes and the sum of the solvation energies for the protein and ligand in free form. ΔG_SA_ was the difference in surface area energies for the ligand-protein complexes and the sum of the surface area energies for protein and ligand.

### 3.8. Alanine Scanning Mutagenesis

ASM analysis was usually used to investigate the role of a specific amino acid residue participating in protein-protein or protein-ligand interactions [45,46]. It was applied to further validate the binding free energy decomposition analysis.

ΔΔG_bind_ = ΔG_bind,mutant_ − ΔG_bind,wild type_

The difference between the binding free energies for the wild type (ΔG_bind,wild type_) and the mutant (ΔG_bind,mutant_) yield the changes of binding free energy (ΔΔG_bind_) arising from the substitution of a specific amino acid by an alanine. More positive ΔΔG_bind_ value indicates that the single mutation caused more significant effects and this specific residue played an important role in ligand binding [45,46].

## 4. Conclusions

This study focused on the synthesis and antiviral activity studies of 4-(quinolin-4-ylamino)benzamide derivatives. All the new compounds were evaluated for their cytotoxicity in MDCK cells and the anti-influenza virus (A/WSN/33, H1N1) activities. The results indicated that 4-(quinolin-4-ylamino)benzamide derivatives exhibited anti-influenza virus activities. **G07** demonstrated significant anti-influenza virus both in cytopathic effect assay (EC_50_ = 11.38 ± 1.89 µM) and anti-influenza assay (IC_50_ = 0.23 ± 0.15 µM) of influenza A virus strain (A/WSN/33, H1N1) in MDCK cells. In addition, **G07** exhibited significant anti-influenza virus activities against other three different influenza virus strains A/PR/8 (H1N1), A/HK/68 (H3N2) and influenza B virus. According to the RNP reconstitution assay, **G07** could interect well with RNP with an inhibition rate of 80.65% at 100 µM, and **G07** exhibited significant activity against PA−PB1 subunit of RNA polymerase based on the PA−PB1 inhibitory activity prediction by the best pharmacophore Hypo1. Therefore, it can be concluded that **G07** is a potential anti-influenza virus agent. The molecular docking and molecular dynamics simulation results indicated that **G07**, **G19** and **G23** could interact well with the PA−PB1 active site, and GLU623, LYS643, TRP706, PHE707 and PHE710 are key amino acid residues interacted with the ligands. It can be speculated that these small molecules exhibited anti-influenza virus activities after blocking the PA−PB1 interface by competing with the PB1. This study can enrich the diverse library of quinoline-based compounds and provides a novel series of molecules for developing potential anti-influenza agents against PA−PB1.

## Figures and Tables

**Table 1:** The results of compounds **G01**–**G26** in cytotoxicity assay, cytopathic effect assay, plaque inhibition assay and PA–PB1 inhibitory activity prediction.

No.	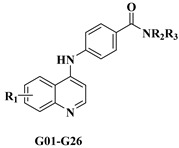		Cytotoxicity Assay CC_50_ ^a^ (µM)		Cytopathic Effect Assay (A/WSN/33, H1N1)	Plaque Inhibition Assay (A/WSN/33, H1N1)		RNP Reconstitution Assay		PA−PB1 Inhibitory Activities
	R_1_	-NR_2_R_3_	MDCK Cell	HEK293T Cell	EC_50_ ^b^ (µM)	Concentration (µM)	Inhibition Rate (%)	Concentration (µM)	Inhibition Rate (%)	Estimated Values (IC_50_ ^c^, µM)
**G01**	7-CF_3_	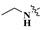	>100	>100	16.48 ± 5.57	100	81.63	100	20.96	>300
**G02**	7-CF_3_	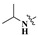	>100	>100	10.10 ± 1.66	100	<10	100	3.10	>300
**G03**	7-CF_3_	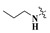	>100	>100	10.04 ± 1.90	100	<10	NT	NT	>300
**G04**	7-CF_3_	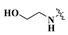	>100	>100	>100	100	59.32	100	14.03	>300
**G05**	7-CF_3_	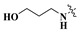	>100	>100	40.85 ± 5.64	100	82.86	100	17.55	>300
**G06**	7-CF_3_	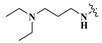	>100	>100	>100	12.5	<10	NT	NT	42.842
**G07**	7-CF_3_	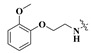	>100	>100	11.38 ± 1.89	100	95.91	100	80.65	35.4716
**G08**	7-CF_3_	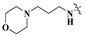	>100	>100	>100	25	11.54	NT	NT	>300
**G09**	7-CF_3_	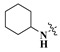	>100	>100	>100	12.5	46.94	NT	NT	>300
**G10**	7-CF_3_	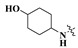	>100	>100	10.36 ± 0.22	100	7.41	NT	NT	>300
**G11**	7-CF_3_	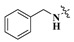	>100	>100	7.17 ± 1.53	100	50.00	NT	NT	>300
**G12**	7-CF_3_	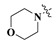	>100	>100	>100	100	<10	100	50.05	>300
**G13**	7-CF_3_	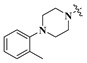	>100	>100	>100	100	<10	NT	NT	>300
**G14**	7-CF_3_	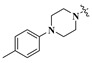	>100	>100	>100	25	<10	NT	NT	>300
**G15**	7-CF_3_	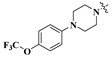	>100	>100	>100	50	61.02	NT	NT	213.79
**G16**	7-CF_3_	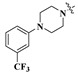	>100	>100	>100	NT	NT	NT	NT	>300
**G17**	7-CF_3_	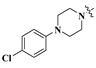	>100	>100	>100	100	73.47	12.5	40.18	>300
**G18**	7-CF_3_	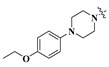	>100	>100	>100	25	16.33	NT	NT	>300
**G19**	H	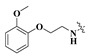	>100	>100	<10	100	82.30	100	13.89	208.688
**G20**	8-CH_3_	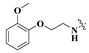	<10	>100	>100	6	74.34	12.5	6.69	>300
**G21**	7-CH_3_	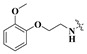	>100	>100	>100	100	11.50	NT	NT	284.787
**G22**	7-OCF_3_	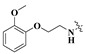	>100	>100	<10	100	20.03	100	43.87	4.91662
**G23**	7-NO_2_	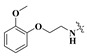	>100	>100	<10	100	77.88	100	56.60	>300
**G24**	6-Br	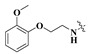	>100	>100	>100	100	<10	NT	NT	>300
**G25**	6-Cl	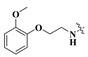	>100	>100	>100	100	0.00	NT	NT	250.6
**G26**	6-CF_3_	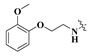	>100	>100	>100	100	<10	100	22.55	234.886
nucleozin			NT	NT	0.21 ± 0.15	100	99.5	100	92.92	NT

^a^ CC_50_: 50% cytotoxic concentration; ^b^ EC_50_: 50% effective concentration; ^c^ IC_50_: 50% inhibitory concentration; NT: not test.

**Table 2:** The results of compounds in plaque inhibition assay.

Compound	Plaque Inhibition Assay IC_50_ (µM)			
	A/WSN/33 (H1N1)	A/PR/8 (H1N1)	A/HK/68 (H3N2)	Influenza B Virus
**G07**	0.23 ± 0.15	11.37 ± 2.38	7.51 ± 1.76	10.99 ± 1.16
nucleozin	0.15 ± 0.11	NT	NT	NT
amantadine	>100	64.20 ± 7.21	2.22 ± 0.57	>100

IC_50_: 50% inhibitory concentration. NT: not test.

**Table 3:** Hydrogen-bond interactions of **G07**, **G19**, **G23** and **H02** with PA−PB1 endonuclease protein (PDB ID: 3CM8).

No.	Hydrogen-Bond Interaction	Hydrogen-Bond Distance (Å)	Docking Scores (kcal/mol)	MM/GBSA (kcal/mol)
**G07**	LYS643	3.0	−7.370	−67.77
	ASN703	3.0		
	ILE621	2.7		
**G19**	LYS643	2.9	−6.677	−55.01
	ASN703	3.1		
	ILE621	3.0		
**G23**	LYS643	3.0	−6.537	−68.94
	ASN703	3.0		
	GLU623	3.7		
**H02**	GLN408	3.0	−6.852	−56.41
	ASN412	3.2		
	GLU623	3.1		
	ILE621	2.7		

## Data Availability

Not applicable.

## Supplementary Materials

The following supporting information can be downloaded at: https://www.mdpi.com/article/10.3390/ijms23116307/s1.
