# Supplementary material for: Design, Synthesis, Molecular Docking Analysis and Biological Evaluations of 4-[(Quinolin-4-yl)amino]benzamide Derivatives as Novel Anti-Influenza Virus Agents
Source: Int J Mol Sci. 2022 Jun 4;23(11):6307. doi: 10.3390/ijms23116307 (PMC9181126; doi:10.3390/ijms23116307)
Supplement: Supplementary file 1 [file ijms-23-06307-s001.zip › ijms-1732038-supplementary.pdf]

International Journal of *Molecular Sciences*

MDPI

Supplementary Materials

Design, Synthesis, Molecular Docking Analysis and Biological Evaluations of 4-[(Quinolin-4-yl)amino]benzamide Derivatives as Novel Anti-Influenza Virus Agents

Chao Zhang [1,†], Yun-Sang Tang [2,†], Chu-Ren Meng [1], Jing Xu [1], De-Liang Zhang [1], Jian Wang [1], Er-Fang Huang [1], Pang-Chui Shaw [2,*] and Chun Hu [1,*]

1 Key Laboratory of Structure-Based Drug Design & Discovery, Ministry of Education, Shenyang Pharmaceutical University, Shenyang 110016, China; zhangchaoylh@126.com (C.Z.); mengchuren@163.com (C.-R.M.); xujing9998881111@163.com (J.X.); zhangdeliang1230@163.com (D.-L.Z.); jianwang@email.com (J.W.); huang222fang@163.com (E.-F.H.)

2 School of Life Sciences, The Chinese University of Hong Kong, Shatin, Hong Kong SAR 999077, China; samtys0910@gmail.com

* Correspondence: pcshaw@cuhk.edu.hk (P.-C.S.); chunhu@syphu.edu.cn (C.H.); Tel.: +86-24-43520246 (C.H.)

† These authors contributed equally to this work.

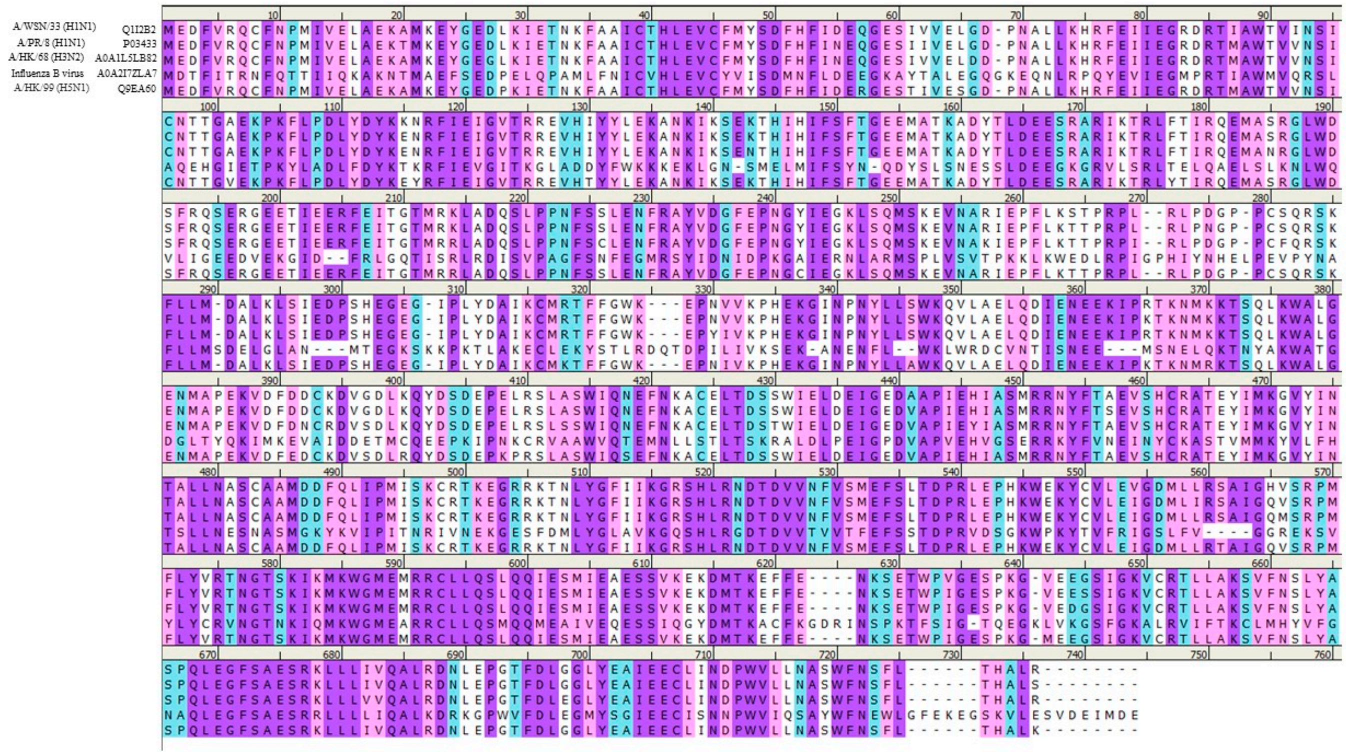

Figure S1. Amino acid sequence alignment of influenza virus PA protein including strain A/WSN/33 (H1N1, Uniprot accession number Q1I2B2), strain A/PR/8 (H1N1, Uniprot accession number P03433), strain A/HK/68 (H3N2, Uniprot accession number A0A1L5LB82), strain A/HK/99 (H5N1, Uniprot accession number Q9EA60) and influenza B virus (Uniprot accession number A0A2I7ZLA7). Residues that are required for PA–PB1 interface are labelled with yellow.

Table S1. MDCK cells survival rate by the target compounds (**G01-G26**) at different concentration.

No.	MDCK cells survival rate (%)			
	**100 μmol·L⁻¹**	**50 μmol·L⁻¹**	**25 μmol·L⁻¹**	**12.5 μmol·L⁻¹**
**G01**	160.62±4.70	121.65±8.73	112.08±3.35	123.45±5.51
**G02**	203.79±6.42	140.08±12.49	137.54±0.71	153.38±36.06
**G03**	144.22±4.46	132.67±1.65	128.70±3.04	107.23±8.32
**G04**	124.89±12.49	126.43±7.03	110.54±13.59	118.54±1.29
**G05**	111.87±2.49	101.63±4.85	110.22±4.44	113.26±1.76
**G06**	79.75±2.72	84.01±5.28	96.67±0.91	95.22±3.96
**G07**	115.75±5.12	256.24±17.89	115.87±11.06	140.55±9.82
**G08**	78.41±2.97	75.20±0.25	76.43±4.79	95.86±1.24
**G09**	133.61±3.89	130.46±12.65	124.74±4.87	156.36±6.13
**G10**	110.17±2.79	127.95±8.15	109.11±15.21	84.64±7.10
**G11**	137.86±9.32	127.54±0.33	123.28±5.69	148.07±0.17
**G12**	120.48±10.37	130.46±5.65	137.81±3.21	92.77±4.37
**G13**	65.91±7.51	92.20±10.16	87.73±16.36	89.39±3.87
**G14**	56.76±2.08	72.26±17.99	91.16±1.34	82.40±6.45
**G15**	76.18±1.27	93.36±4.01	82.80±5.22	92.13±3.70
**G16**	103.91±10.21	92.23±7.81	85.52±2.56	83.03±1.26
**G17**	51.32±5.29	63.22±11.25	65.78±1.15	71.49±1.03
**G18**	104.99±4.01	138.28±6.89	111.55±2.20	112.99±2.36
**G19**	111.55±9.83	102.17±1.95	93.23±8.08	90.89±5.31
**G20**	8.66±0.17	8.88±0.51	8.61±0.42	45.97±9.1
**G21**	88.84±3.59	93.28±9.93	80.18±7.27	83.41±3.67

**G22**	118.12±6.49	104.22±9.18	99.56±3.73	95.78±2.47
**G23**	91.28±3.45	100.33±3.53	106.22±9.35	101.22±6.39
**G24**	105.38±8.08	87.44±3.70	71.98±13.89	97.97±11.82
**G25**	79.32±11.90	86.83±3.19	85.96±5.73	92.58±5.23
**G26**	133.91±11.97	122.08±7.85	105.52±6.56	103.03±1.26

Table S2. HEK293T cells survival rate by the target compounds (**G01-G26**) at different concentration.

**No.**	**HEK293T cells survival rate (%)**					
	**100 μmol·L⁻¹**	**50 μmol·L⁻¹**	**25 μmol·L⁻¹**	**12.5 μmol·L⁻¹**	**6.25 μmol·L⁻¹**	**3.125 μmol·L⁻¹**
**G01**	94.92±8.61	88.18±3.27	92.83±4.26	93.22±5.27	90.01±2.87	91.00±7.40
**G02**	66.13±2.32	75.54±11.17	74.04±2.91	85.31±11.55	87.90±2.52	95.91±3.00
**G03**	74.22±4.02	84.12±2.84	91.10±7.09	97.78±4.47	89.61±8.87	98.97±9.80
**G04**	96.30±10.52	98.62±11.78	91.99±5.43	94.50±4.33	100.01±1.56	112.00±4.22
**G05**	122.90±10.09	93.06±1.61	95.07±1.76	96.82±2.21	109.11±25.17	106.06±4.46
**G06**	76.47±9.94	85.25±2.63	85.50±2.49	96.59±4.26	90.35±0.85	107.15±2.76
**G07**	81.39±7.21	90.17±2.90	87.01±2.19	91.58±3.34	97.91±1.23	106.60±2.14
**G08**	112.13±1.41	98.07±1.80	95.56±2.29	95.72±3.30	97.00±3.10	100.86±3.39
**G09**	81.22±3.80	101.55±2.23	96.79±0.62	92.83±3.70	98.20±3.74	106.48±6.90
**G10**	109.24±7.99	99.09±8.41	98.50±8.84	98.12±8.96	104.56±4.92	108.01±12.84
**G11**	86.44±8.60	103.77±9.35	108.76±5.63	105.25±12.82	101.78±7.08	129.49±22.76
**G12**	74.00±4.57	80.68±3.36	88.50±7.54	86.67±6.98	89.50±2.30	100.47±5.92
**G13**	81.89±2.67	80.60±2.01	88.23±4.29	89.92±2.14	96.29±4.65	99.14±6.42
**G14**	77.49±2.55	76.77±5.03	80.67±2.20	89.38±1.59	95.00±7.82	105.78±7.34
**G15**	81.59±1.91	89.01±2.08	103.00±3.15	93.38±3.30	104.46±0.45	105.64±1.62
**G16**	66.51±2.68	70.68±3.36	75.35±5.51	80.26±3.54	79.52±2.87	89.31±3.06
**G17**	59.17±1.33	52.36±1.02	62.61±12.04	78.02±11.06	89.66±6.31	86.81±4.81
**G18**	86.90±1.67	72.68±1.96	65.43±6.72	70.43±2.98	87.82±9.55	98.42±9.98
**G19**	112.18±7.45	97.40±3.36	97.97±3.74	103.11±7.45	103.54±9.29	103.14±6.28
**G20**	60.29±0.35	80.21±1.35	87.46±21.36	94.07±9.07	91.48±3.65	96.66±8.01
**G21**	50.09±0.70	110.67±8.55	102.64±2.91	105.56±6.72	103.95±2.65	117.39±1.03
**G22**	86.07±5.65	88.83±5.80	89.97±4.08	91.65±2.90	103.91±6.77	117.25±6.52
**G23**	88.11±4.02	96.62±7.74	89.02±3.47	95.29±3.05	96.34±4.31	93.57±3.31
**G24**	72.95±4.52	69.53±3.05	91.06±10.23	91.88±3.61	96.70±5.40	113.31±4.70
**G25**	110.35±11.54	101.61±11.65	65.73±7.64	99.13±1.95	103.50±6.33	104.71±1.87
**G26**	70.01±9.07	72.10±1.56	74.64±5.24	78.70±2.98	83.92±2.40	104.54±2.55

Table S3. The results of the target compounds (**G01-G26**) in CPE assay.

**No.**	**Antiviral activity (%)**			
	**100 μmol·L⁻¹**	**50 μmol·L⁻¹**	**25 μmol·L⁻¹**	**12.5 μmol·L⁻¹**
**G01**	70.86±2.63	53.02±4.28	56.59±3.96	47.99±4.04
**G02**	80.63±9.03	76.65±2.26	61.59±2.13	53.61±3.38
**G03**	78.65±8.35	70.28±9.33	56.70±11.65	54.88±4.62
**G04**	49.29±0.74	57.10±9.30	54.64±4.19	55.30±4.28
**G05**	56.04±4.37	50.22±1.80	48.58±2.31	41.99±5.50
**G06**	11.19±0.23	12.81±0.69	13.13±0.69	16.70±1.15
**G07**	111.81±4.30	106.66±2.43	78.16±8.28	61.4±6.86
**G08**	15.40±0.23	31.13±8.71	45.56±9.40	44.26±3.44
**G09**	23.19±3.90	21.40±0.92	22.86±3.90	31.94±0.69
**G10**	63.82±5.18	66.21±2.87	58.04±1.69	48.88±2.01
**G11**	47.34±0.92	44.10±1.38	52.37±3.44	47.67±9.63
**G12**	76.69±5.27	75.72±9.49	97.61±3.67	70.86±4.82

**G13**	34.33±7.15	36.85±4.41	33.13±4.47	27.01±2.20
**G14**	27.85±2.05	23.35±2.31	20.69±3.82	16.43±0.12
**G15**	45.63±5.12	35.30±1.81	39.84±4.98	36.89±0.82
**G16**	45.67±2.09	33.78±2.71	36.84±3.16	15.65±1.33
**G17**	28.52±7.81	21.09±1.62	27.32±3.16	33.50±1.23
**G18**	35.46±9.67	38.69±4.29	39.45±1.72	42.79±0.57
**G19**	122.29±5.08	137.86±9.95	131.63±4.75	130.64±11.05
**G20**	31.14±0.49	30.86±0.88	28.59±1.61	72.05±6.36
**G21**	50.11±5.45	39.77±6.58	39.77±7.16	43.03±7.31
**G22**	97.38±10.19	105.73±5.94	108.00±8.75	112.24±6.15
**G23**	119.46±7.71	119.75±0.68	143.1±6.11	145.65±3.38
**G24**	18.38±1.27	19.17±0.42	19.22±0.69	20.99±2.86
**G25**	22.28±0.40	22.51±1.29	25.71±2.29	26.10±0.67
**G26**	49.67±6.09	36.78±1.79	16.82±2.11	15.65±1.06

Table S4. The results of the target compound (**G07**) in plaque inhibition assay against different influenza virus strains.

**Influenza virus strains**	**Viral Yield (%)**				
	**100 $\mu mol\cdot L^{-1}$**	**50 $\mu mol\cdot L^{-1}$**	**25 $\mu mol\cdot L^{-1}$**	**12.5 $\mu mol\cdot L^{-1}$**	**6.25 $\mu mol\cdot L^{-1}$**
**A/WSN/33 (H1N1)**	4.09±0.14	39.70±0.88	5.00±2.18	24.55±0.43	18.86±1.30
**Influenza B virus**	7.79±1.49	11.93±1.77	39.86±6.11	43.38±6.70	70.02±5.44
**A/PR/8 (H1N1)**	0	1.18±0.22	16.64±3.83	55.79±5.58	70.77±8.80
**A/HK/68 (H1N1)**	0.05±0.04	0.16±0.12	1.10±8.27	45.00±9.04	77.00±5.61

1. Materials and Method

1.1. Pharmacophore Model Generation

1.1.1. Compound Preparations

39 PA-PB1 endonuclease inhibitors collected from difference literatures [1-7] were split into training set and test set. The two-dimensional (2D) structures of these compounds were drawn by BIOVIA Draw 2016. The corresponding three-dimensional (3D) form were converted by Discovery Studio 2016 [8]. The training set compounds and test set compounds were enlisted in Figures **S2** and **S3**. The pharmacophore models were generated using training set. The test set was utilized to validate the generated pharmacophore model. We minimized each compound using Smart Minimizer algorithm based on CHARMM force field method. In addition, each compound could form a variety of maximum 255 different conformations to generate pharmacophore hypothesis [9].

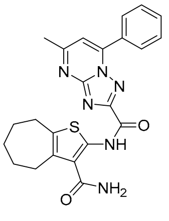

H01

(IC_{50} = 1.1 μM)

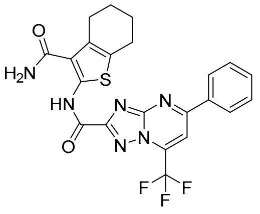

H02

(IC_{50} = 7.5 μM)

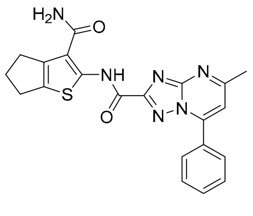

H03

(IC_{50} = 6.2 μM)

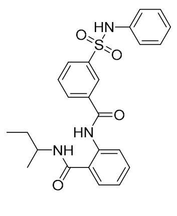

H04

(IC_{50} = 9.2 μM)

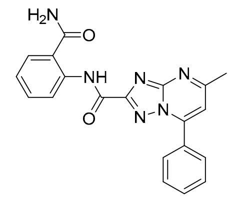

H05

(IC$_{50}$ = 11 μM)

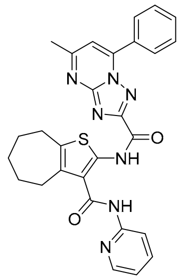

H06

(IC$_{50}$ = 23 μM)

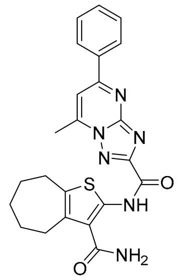

H07

(IC$_{50}$ = 28 μM)

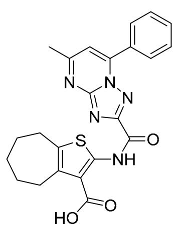

H08

(IC$_{50}$ = 19 μM)

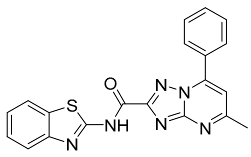

H09

(IC$_{50}$ = 21 μM)

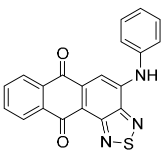

H10

(IC$_{50}$ = 9.5 μM)

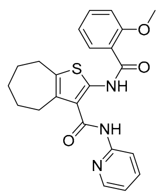

H11

(IC$_{50}$ = 22 μM)

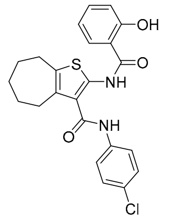

H12

(IC$_{50}$ = 18 μM)

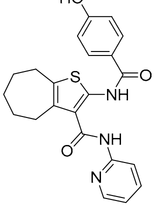

H13

(IC$_{50}$ = 26 μM)

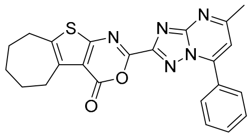

H14

(IC$_{50}$ = 19 μM)

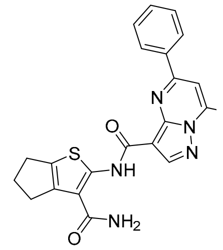

H15

(IC$_{50}$ = 44.8 μM)

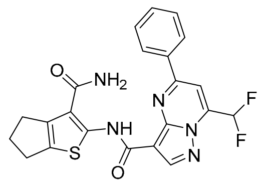

H16

(IC$_{50}$ = 25.4 μM)

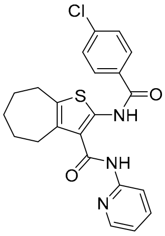

H17

(IC$_{50}$ = 32 μM)

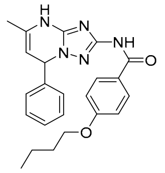

H18

(IC$_{50}$ = 37 μM)

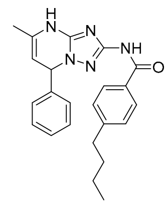

H19

(IC$_{50}$ = 48 μM)

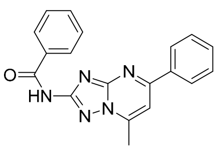

H20

(IC$_{50}$ = 26 μM)

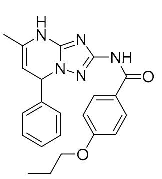

H21

(IC$_{50}$ = 40 μM)

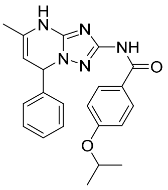

H22

(IC$_{50}$ = 75 μM)

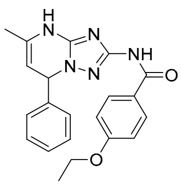

H23

(IC$_{50}$ = 139 μM)

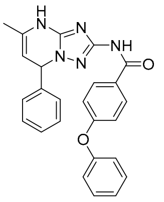

H24

(IC$_{50}$ = 156 μM)

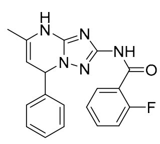

H25

(IC_{50} = 185 μM)

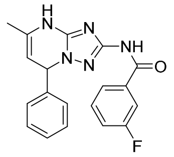

H26

(IC_{50} = 156 μM)

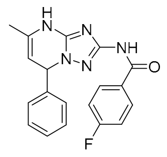

H27

(IC_{50} = 138 μM)

Figure S2. Chemical structures of PA-PB1 endonuclease inhibitors in the training set together with their biological activity data (IC_{50} value, μM).

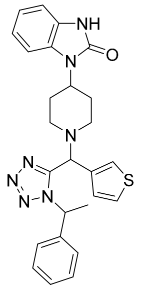

H28

(IC_{50} = 7.6 μM)

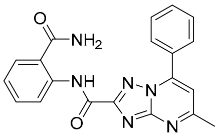

H29

(IC_{50} = 11 μM)

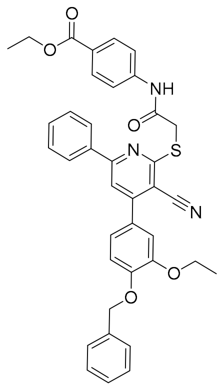

H30

(IC_{50} = 30 μM)

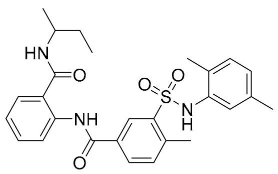

H31

(IC_{50} = 30.5 μM)

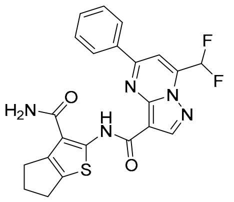

H32

(IC_{50} = 25.4 μM)

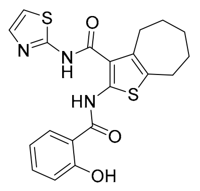

H33

(IC_{50} = 69 μM)

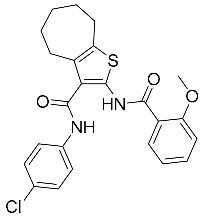

H34

(IC_{50} = 78 μM)

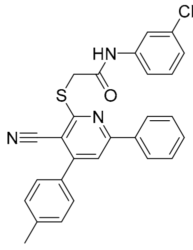

H35

(IC_{50} = 180 μM)

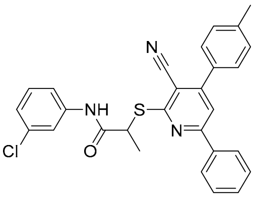

H36

(IC_{50} = 125 μM)

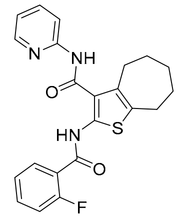

H37

(IC_{50} = 145 μM)

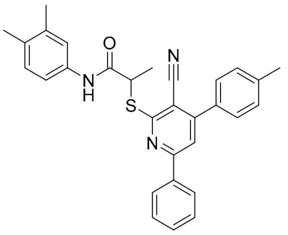

H38

(IC_{50} = 180 μM)

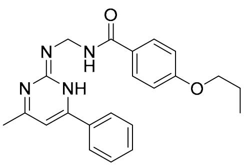

H39

(IC_{50} = 200 μM)

Figure S3. Chemical structures of PA-PB1 endonuclease inhibitors in the test set together with their biological activity data (IC_{50} value, μM).

1.1.2. Generation of Pharmacophore Models

The 3D-QSAR pharmacophore generation protocol in Discovery Studio 2016 were used to generate the pharmacophore models. Usually, there were six different chemical features [hydrogen bond acceptor (HBA), hydrogen bond donor (HBD), hydrophobic (HYD), positive ionizable (PI), ring aromatic (RA) and negative ionizable (NI)]. Feature

Mapping protocol was used to seek the presenting chemical features of the training set molecules. FAST algorithm method was used to generate acceptable conformations with 10 kcal/mol as the energy threshold. We set uncertainty values of each compound (training set and test set compounds) as 1.5. The active property represented the IC_{50} values of compounds. We usually set the maximum excluded volume as zero during the pharmacophore generation. Furthermore, Minimum Inter feature Distance was ranged from 2.97 to 2.0. The energy threshold was maintained at 20 kcal/mol [10].

1.1.3. Pharmacophore Validation

Three kinds of methods (Cost analysis, Fischer's randomization test and Test set analysis) were usually to validate the generated pharmacophore models.

ΔCost (Null cost - Total cost) is a key parameter in cost analysis. It infers more than 90% prediction range if the cost difference is more than 60 bits. The model might exhibit 70-90% prediction range if the cost difference is in the range of 40-60 bits. The model is unreliable when the cost difference is less than 40 bits [11]. Fischer's randomization test also could be used to validate the selected pharmacophore hypothesis [12]. Test set analysis is another method to validate the pharmacophore model by Ligand Pharmacophore Mapping protocol (Discovery Studio 2016) [11].

2. Result and Discussion

2.1. Pharmacophore Model Generation

The Hypogen algorithm 3D-QSAR Pharmacophore (Discovery Studio 2016) was used to generate pharmacophore models (Table S5) using 27 training set compounds (1.1 μM to 185 μM). Figure **S4** demonstrated the features (HBA, RA and HYD) of the training set molecules. The statistical parameters (cost, correlation coefficient, RMSD and chemical features) of the top 10 pharmacophore hypotheses were enlisted in Table **S5**. The null cost and fixed cost of the generated pharmacophore models were 169.572 and 79.8661, respectively. The total costs were ranging from 94.2536 to 111.805. Hypo1 with the highest cost difference (75.318), the best correlation coefficient (0.937771), the least RMSD (0.973343), and the lowest total cost value (94.2536) was regarded as the best hypothesis.

The molecules selected from previously articles were divided into four groups of magnitude based on their corresponding experimental activity value (IC_{50}): most active (≤ 10 μM, ++++), active (10 to 100 μM, +++), moderately active (100 to 150 μM, ++) and inactive (>150 μM, +). The specific experimental and estimate activity values of the training set compounds were enlisted in Table **S6**. Compound **H01** mapped well with all the essential features and was considered as the most active molecules (Figure **S5A**). Compound **H27** (Figure **S5B**) as the least active molecules in the test set were not nicely mapped with all the essential features.

Table S5. Statistical results of the top 10 pharmacophore hypotheses generated by HypoGenalgorithm.

Hypo.no.	Total cost	Cost difference	RMSD	Correlation	Max.Fit	Features
1	94.2536	75.318	0.973343	0.937771	5.84065	HBA, HYD, HYD, RA
2	95.5169	74.055	1.05636	0.926099	6.69214	HBD, HYD, HYD, RA
3	103.639	65.933	1.26886	0.891562	5.56148	HBD, HYD, HYD, RA
4	103.659	65.913	1.32377	0.881017	7.36907	HBA, HYD, HYD, RA

5	105.824	63.748	1.33186	0.87975	5.5772	HBD, HYD, HYD, RA
6	109.041	60.531	1.35845	0.874663	4.47324	HBA, HBA, HYD, RA
7	110.744	58.828	1.5048	0.843123	7.05175	HBA, HYD, HYD, RA
8	111.258	58.314	1.37231	0.871908	3.82642	HBA, HBA, RA
9	111.479	58.093	1.46451	0.852467	3.911	HBA, HBA, RA
10	111.805	57.767	1.48427	0.848056	5.46743	HBD, HYD, HYD, RA

Null cost = 169.572, *Fixed cost = 79.8661, *Best record in pass = 4.

(RA-ring aromatic, HBA-hydrogen bond acceptor, HBD-hydrogen bond donor, HYD-hydrophobic).

Table S6. Experimental and Estimate activity of the training set compounds based on pharmacophore model Hypo1.

**Comp No.**	**IC_{50} value(μM)**		Errors[a]	Fit value[b]	Activity scale[c]	
	**Experimental**	Estimated			Experimental	Estimated
**H01**	**1.1**	1.13348	1.03044	5.51979	++++	++++
H02	6.2	3.1319	-1.97963	5.07839	++++	++++
H03	7.5	10.9565	1.46087	4.53453	++++	++++
H04	9.2	11.6099	1.26195	4.50937	++++	+++
H05	11	11.7061	1.06419	4.50579	+++	+++
H06	23	18.6451	-1.23357	4.30364	+++	+++
H07	28	19.6764	-1.42302	4.28025	+++	+++
H08	19	20.3469	1.07089	4.2657	+++	+++
H09	21	21.1091	1.0052	4.24973	+++	+++
H10	9.5	22.2311	2.34012	4.22724	++++	+++
H11	22	22.8343	1.03792	4.21561	+++	+++
H12	18	23.2991	1.29439	4.20686	+++	+++
H13	26	23.425	-1.10993	4.20452	+++	+++
H14	19	23.5925	1.24171	4.20143	+++	+++
H15	44.8	23.8032	-1.8821	4.19756	+++	+++
H16	25.4	25.1077	-1.01164	4.17439	+++	+++
H17	32	25.4979	-1.25501	4.1677	+++	+++
H18	37	32.333	-1.14434	4.06455	+++	+++
H19	48	57.8447	1.2051	3.81194	+++	+++
H20	26	60.705	2.33481	3.79098	+++	+++
H21	40	80.8886	2.02222	3.66631	+++	+++
H22	75	83.5914	1.11455	3.65204	+++	+++
H23	139	84.0055	-1.65465	3.64989	++	+++
H24	156	92.7544	-1.68186	3.60687	+	+++
H25	185	113.674	-1.62746	3.51854	+	++
H26	156	130.409	-1.19624	3.45889	+	++
H27	138	143.644	1.0409	3.41691	++	++

[a] Error factor calculated as the ratio of the measured activity to the estimated activity; positive value indicates that the estimated IC_{50} is higher than the experimental IC_{50}; a negative value indicates that the estimated IC_{50} is lower than the experimental IC_{50} value.

[b] Fit value indicates how well the features in the pharmacophore map with the chemical features present in the compound.

[c] Activity scale: ++++, $IC_{50} \leq 10$ µM (most active); +++, IC_{50} 10 to 100 µM (active); ++, IC_{50} 100 to 150 µM (moderately active); +, IC_{50} >150 µM (inactive)

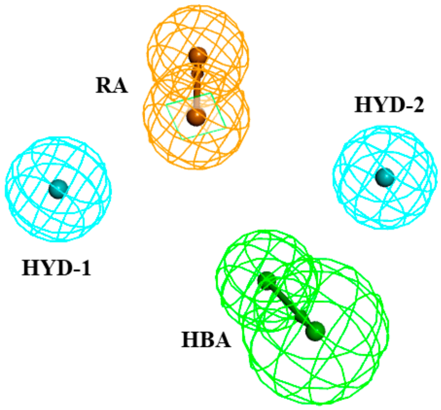

Figure S4. The best HypoGen Pharmacophore model (Hypo1). Green color represents HBA, Purple color represents HBD, Blue color represents HYD.

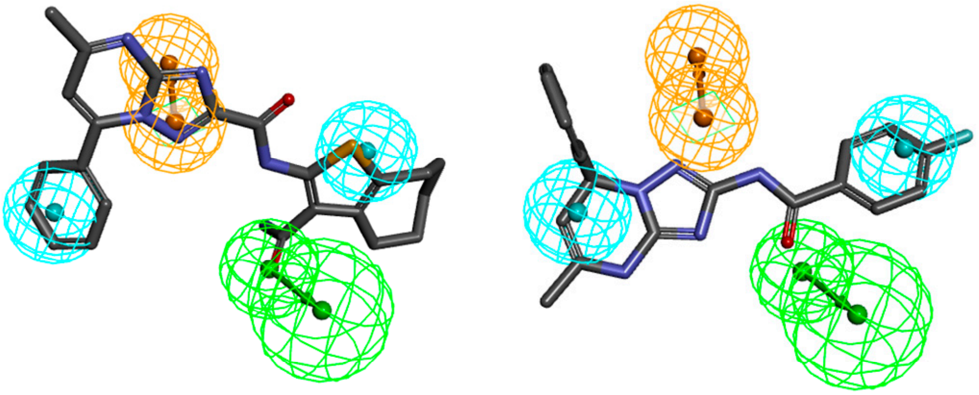

(A) Hypo1-**H01** (B) Hypo1-**H27**

Figure S5. Pharmacophore mapping of the most active and less active compounds in the training set. (A) Hypo1 mapped on to the most active compound **H01**; (B) Hypo1 mapped on to the least active compound **H27**.

2.2. Validation of the Pharmacophore Models

2.2.1. Cost Analysis

Three cost values (total cost, fixed cost, and null cost) (**Table S5**) were used to analyze the ability of Hypo1 to predict the experimental activity of training set compounds. The highest cost difference value (75.318) of Hypo1 demonstrated that Hypo1 exhibited significant predicted probability. Thus, Hypo1 could be considered as a significant pharmacophore model depended on the cost analysis.

2.2.2. Fischer's Randomization Test

The experimental activity values of training set compounds were scrambled randomly. With a 95% confidence level, these values were used in pharmacophore generation and put forth 19 random spreadsheets. Then we compared these results with the originally generated pharmacophore (Hypo1). Figure S6 revealed the differences of correlations (Figure S6A) and costs values (Figure S6B) between the HypoGen and Fischer's randomizations. None of the randomly generated pharmacophores obtained a better statistical value than Hypo1.

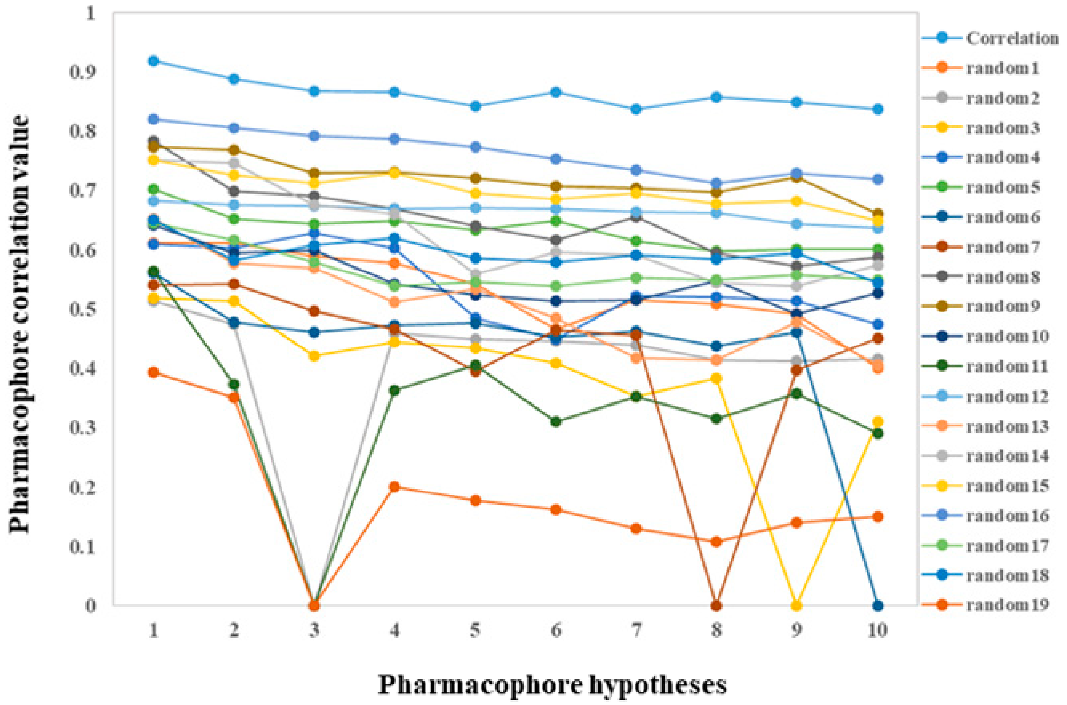

(A)

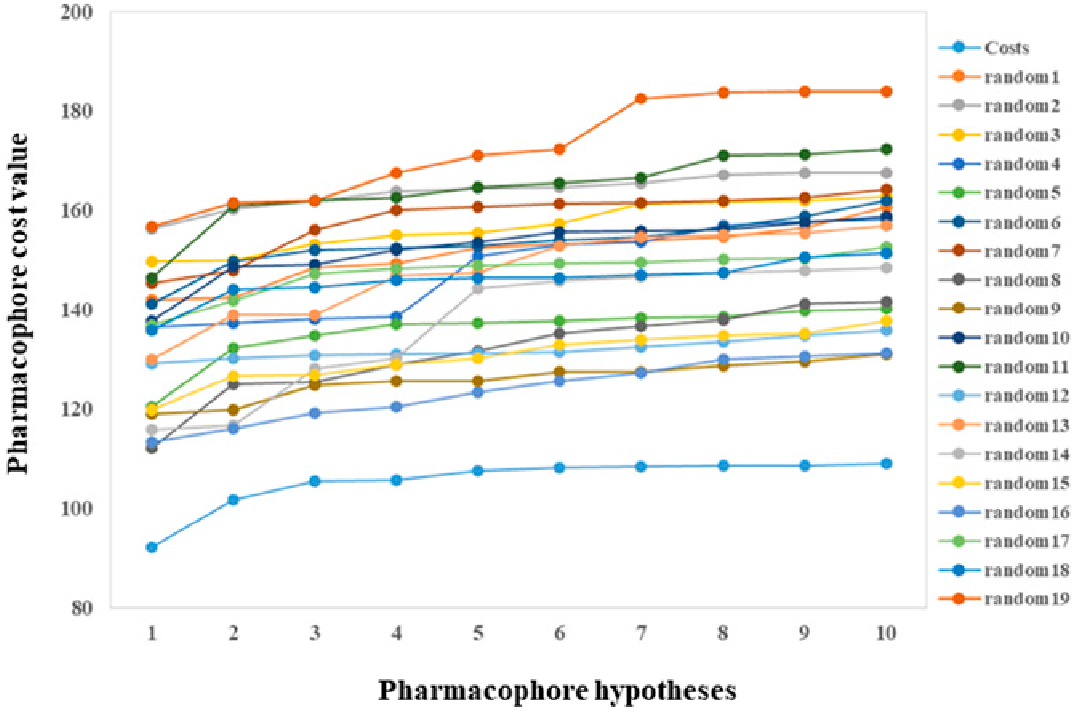

(B)

Figure S6. (**A**) & (**B**): The difference in correlation and total cost values of hypotheses between a Hypo1 spreadsheet and 19 random spreadsheets.

2.2.3. Test Set Analysis

Table S7. Experimental and Estimate activity of the test set compounds based on pharmacophore model Hypo1.

Comp No.	IC_{50} value(μM)		Errors[a]	Fit value[b]	Activity scale[c]	
	**Experimental**	Estimated			Experimental	Estimated
**H28**	**7.6**	5.07013	-1.49898	4.86918	++++	++++
H29	11	11.7061	1.06419	4.50579	+++	+++
H30	30	13.764	-2.1796	4.43546	+++	+++
H31	30.5	14.5216	-2.10032	4.41218	+++	+++
H32	25.4	45.1026	1.77569	3.92	+++	+++
H33	69	55.8074	-1.2364	3.82751	+++	+++
H34	78	62.5108	-1.24778	3.77825	+++	+++
H35	180	125.726	-1.43168	3.47478	+	++
H36	125	135.665	1.08532	3.44173	++	++
H37	145	141.311	-1.02611	3.42402	++	++
H38	180	158.88	-1.13293	3.37313	+	+
H39	200	255.843	1.27921	3.16623	+	+

[a] Error factor calculated as the ratio of the measured activity to the estimated activity; positive value indicates that the estimated IC_{50} is higher than the experimental IC_{50}; a negative value indicates that the estimated IC_{50} is lower than the experimental IC_{50} value.

[b] Fit value indicates how well the features in the pharmacophore map with the chemical features present in the compound.

[c] Activity scale: ++++, IC_{50} ≤ 10 μM (most active); +++, IC_{50} 10 to 100 μM (active); ++, IC_{50} 100 to 150 μM (moderately active); +, IC_{50} >150 μM (inactive).

The reliability of the selected pharmacophore model depends on its ability to predict the bioactivity of the test set compounds. 12 chemically diverse compounds with IC_{50} values ranged from 7.6 μM to 200 μM were selected as test set. We used the protocol in Discovery Studio to map the test set molecules to test the predictability ability of the pharmacophore model. Table **S7** enlisted the estimated activity values of the test set compounds. The correlation coefficients between experimental and estimated PA-PB1 endonuclease inhibitory activity values for the training set (r^2 = 0.937771) and the test set (r^2 = 0.909000) were shown in Figure **S7**. Among the 12 compounds, the best active compound T28 mapped well with the four essential features (Figure **S8**). The least active molecule T39 did not map with the essential RA feature, which signified the robustness of the pharmacophore model (Figure **S8**).

The employed three validation strategies implied that Hypo1 model could be selected as a significant pharmacophore model for further screening chemical databases with diverse structural entities.

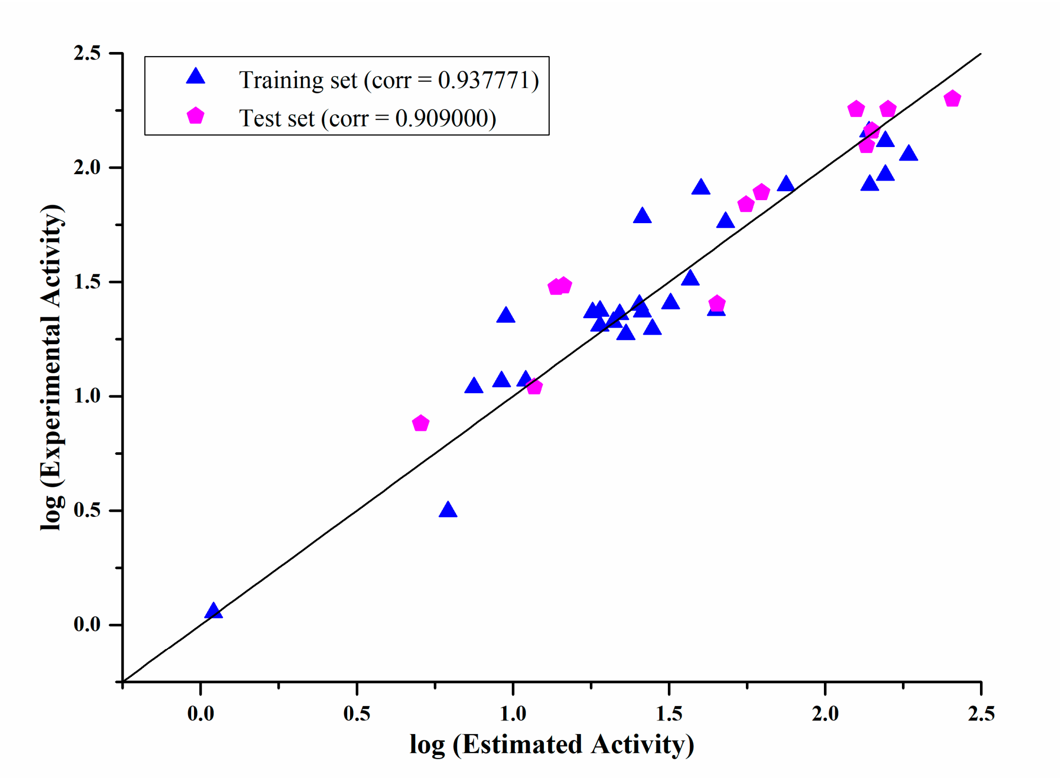

Figure S7. Correlation graph between experimental and estimated activity values in logarithmic scale for training and test set compounds based on Hypo1.

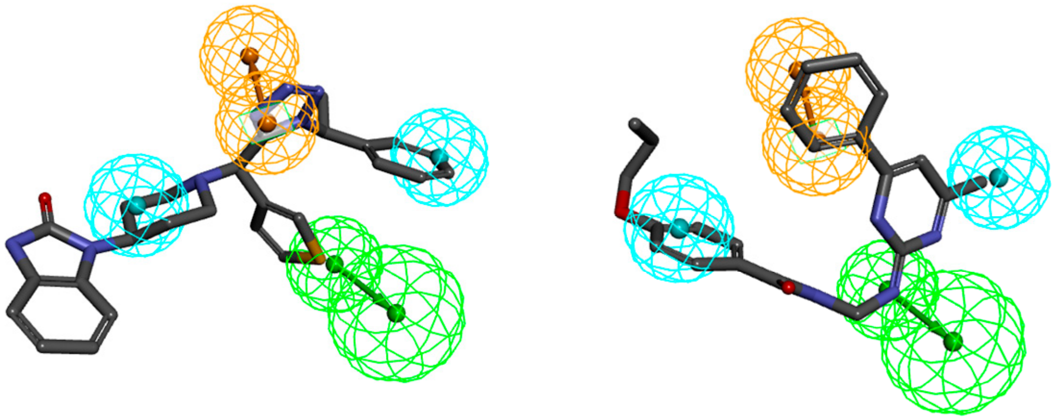

(A) Hypo1 – **H28** (B) Hypo1 – **H39**

Figure S8. Pharmacophore mapping of the most active, less active compounds in the test set. (**A**) Hypo1 mapped on to the most active compound **H28**; (**B**) Hypo1 mapped on to the least active compound **H39**.

Table S8. The results of Lipinski's rule calculation for compounds **G01-G26**.

Comp No.	Alop	MW	No. Aromatic Ring	No. HBA	No. HBD	No. rotatable bonds
**G01**	4.065	359.352	3	3	2	5
**G02**	4.443	373.379	3	3	2	5
**G03**	4.589	373.379	3	3	2	6
**G04**	3.177	375.352	3	4	3	6
**G05**	3.239	389.379	3	4	3	7

**G06**	4.615	444.502	3	4	2	10
**G07**	5.332	481.477	4	5	2	9
**G08**	3.605	458.486	3	5	2	8
**G09**	5.575	413.444	3	3	2	5
**G10**	4.338	429.444	3	4	3	5
**G11**	5.3	421.423	4	3	2	6
**G12**	3.61	401.39	3	4	1	4
**G13**	6.038	490.53	4	4	1	5
**G14**	6.038	490.53	4	4	1	5
**G15**	7.671	560.5	4	4	1	7
**G16**	6.494	544.5	4	4	1	6
**G17**	6.216	510.948	4	4	1	5
**G18**	5.884	520.557	4	5	1	7
**G19**	4.39	413.48	4	5	2	8
**G20**	4.876	427.507	4	5	2	8
**G21**	4.876	427.507	4	5	2	8
**G22**	6.51	497.477	4	6	2	10
**G23**	3.141	459.487	4	7	3	9
**G24**	5.138	492.376	4	5	2	8
**G25**	5.054	447.925	4	5	2	8
**G26**	5.332	481.477	4	5	2	9

MW: molecular weight; HBA: H-bond acceptors; HBD: H-bond donors

Table S9. The ADMET prediction results for compounds **G01-G26.**

Comp No.	ADME Solubility Level	ADME BBB Level	ADME Absorption Level	Hepatotoxic	PPB Prediction	Toxicity Probability
**G01**	2	1	0	-5.05652	true	0.0226
**G02**	2	1	0	-3.80375	true	0.0197
**G03**	2	1	0	-4.21702	true	0.0236
**G04**	2	2	0	-5.84686	true	0.0322
**G05**	2	2	0	-6.95457	true	0.0304
**G06**	2	1	0	-6.90941	true	0.0203
**G07**	1	1	0	-7.21807	true	0.0229
**G08**	2	2	0	-10.661	true	0.0165
**G09**	1	0	0	-5.17018	true	0.0249
**G10**	2	1	0	-6.97709	true	0.0296
**G11**	1	1	0	-6.33921	true	0.0237
**G12**	2	1	0	-7.2289	true	0.0236
**G13**	1	0	1	-8.26976	true	0.00342
**G14**	1	0	1	-6.33634	true	0.00431
**G15**	0	4	3	-5.20708	true	0.00504
**G16**	1	4	1	-8.60851	true	0.000342
**G17**	1	0	1	-4.6823	true	0.000893
**G18**	1	0	1	-7.27629	true	0.00412
**G19**	2	1	0	-3.18426	true	0.0459
**G20**	2	1	0	-6.19844	true	0.0549
**G21**	2	1	0	-4.72612	true	0.0541
**G22**	1	4	2	-3.67127	true	0.0443
**G23**	3	4	0	-2.89167	true	0.0211

**G24**	1	1	0	-3.07058	true	0.0218
**G25**	2	1	0	-2.25817	true	0.0155
**G26**	1	1	0	-8.09596	true	0.0508

ADME_Solubility_Level: 0 (Extremely low); 1 (No, very low, but possible); 2 (Yes, low); 3 (Yes, good); 4 (Yes, optimal); 5(No, too soluble); 6(Warning: molecules with one or more unknown AlogP98 types).

ADME_BBB_Level: 0 (Very high penetrant); 1 (High); 2 (Medium); 3 (Low); 4 (Undefined).

ADME_Absorption_Level: 0 (Good absorption); 1 (Moderate absorption); 2 (Low absorption); 3 (Very low absorption).

EXT_ Hepatotoxic: < -0.4095: non-toxic; > -0.4095: inhibitor.

EXT_PPB_Prediction: plasma protein binding ability, false: ≥ 90 %; true: ≤ 90 %.

Toxicity Probability: 0.0-0.30(Low); 0.30-0.70 (Medium); 0.70-1.0 (High).

Appendix: spectrocopic charts of the target compounds

Chemical Formula: $C_{17}H_{11}F_3N_2O_2$
MS (ESI): calcd for 333.1 ($[M+H]^+$)

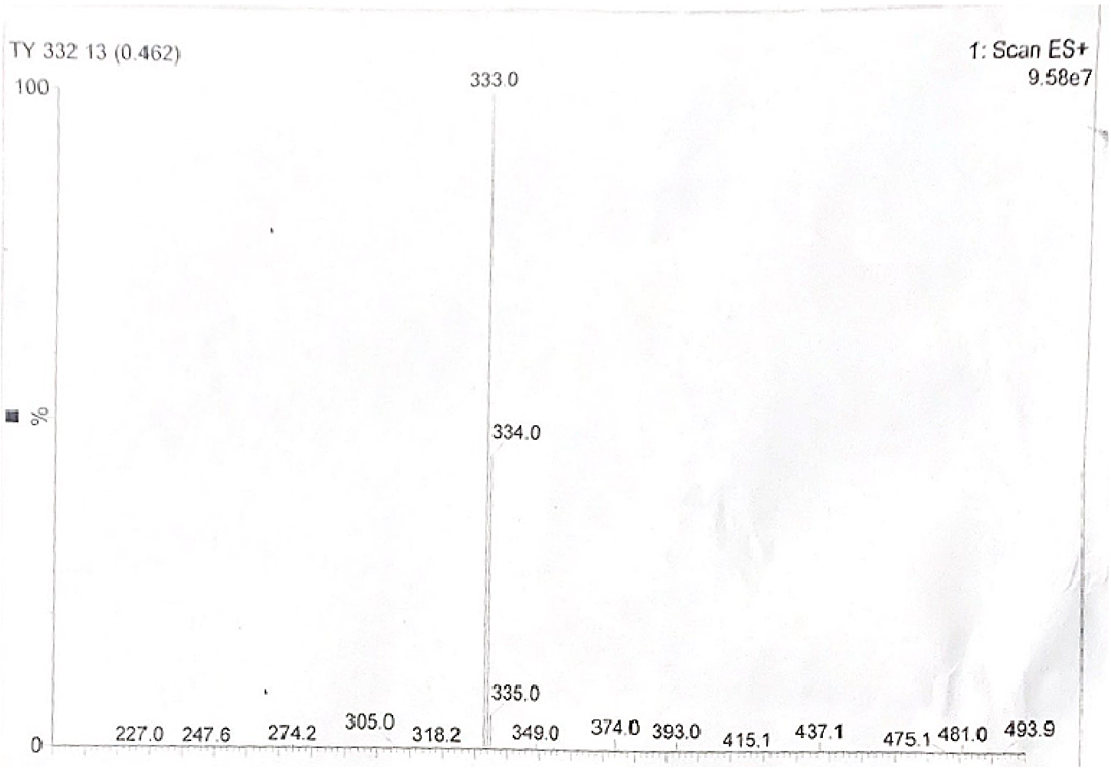

Figure S9. MS (ESI) of the target compound **F01.**

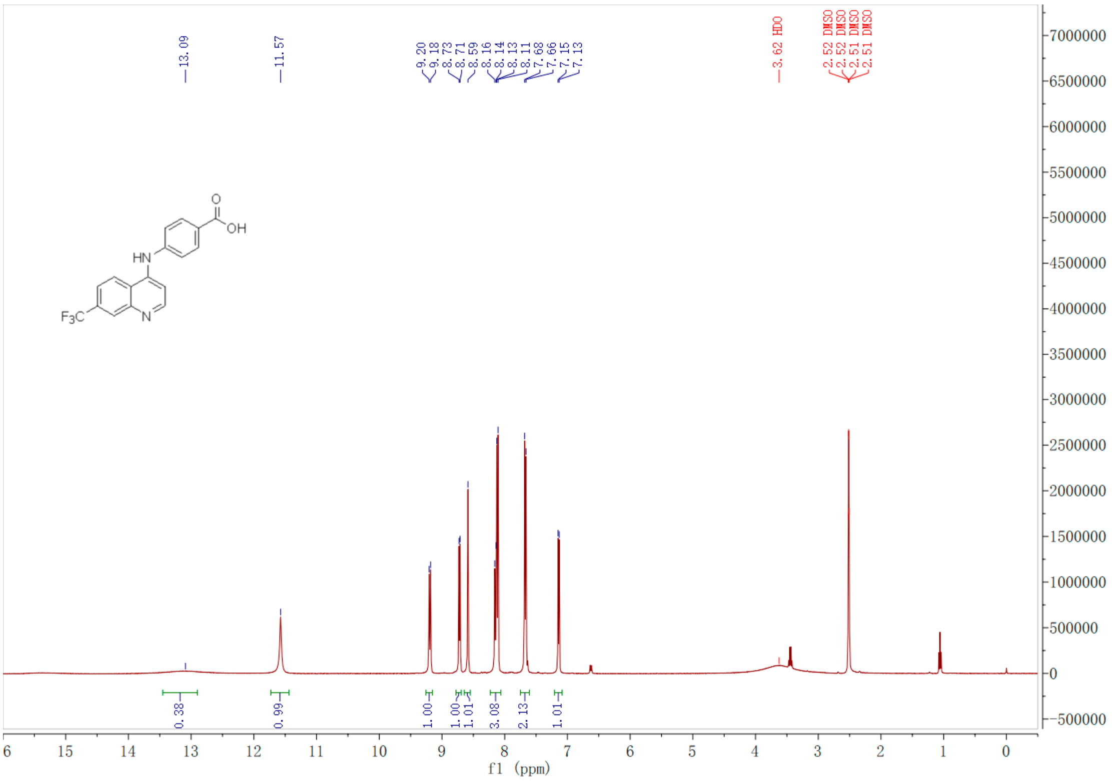

Figure S10. ^{1}H NMR spectra of the target compound **F01.**

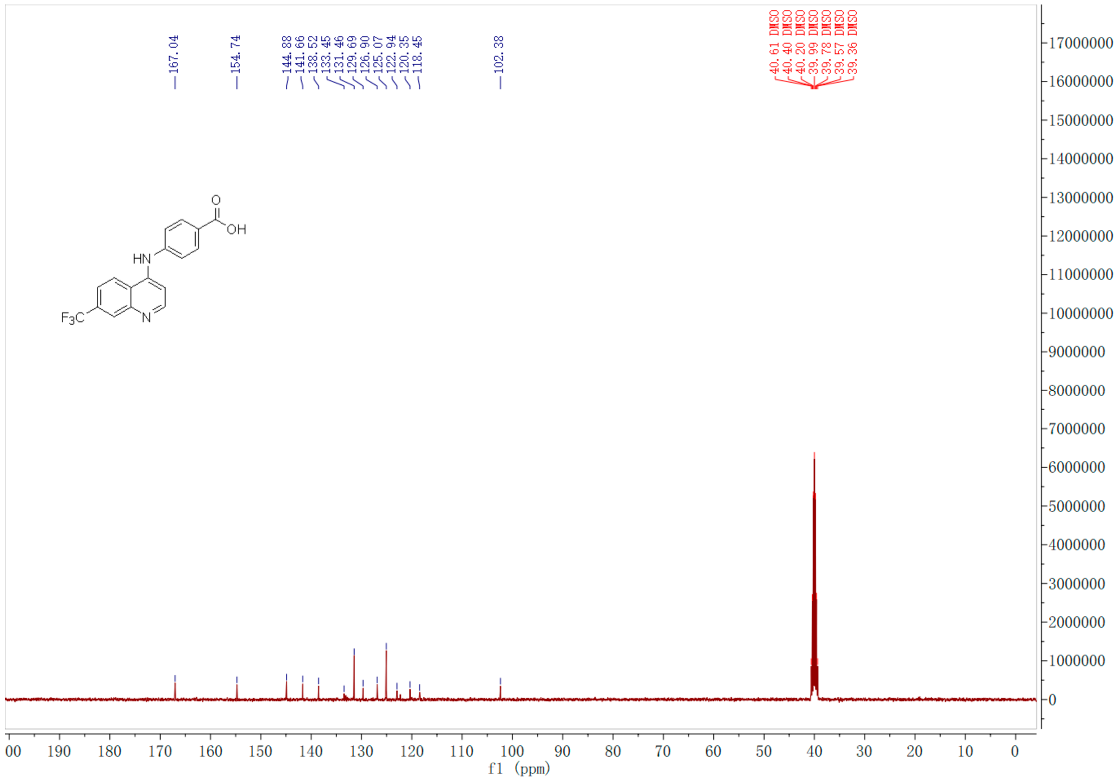

Figure S11. ^{13}C NMR spectra of the target compound **F01.**

Chemical Formula: $C_{16}H_{12}N_2O_2$
MS (ESI): calcd for 265.1 ([M+H]$^+$)

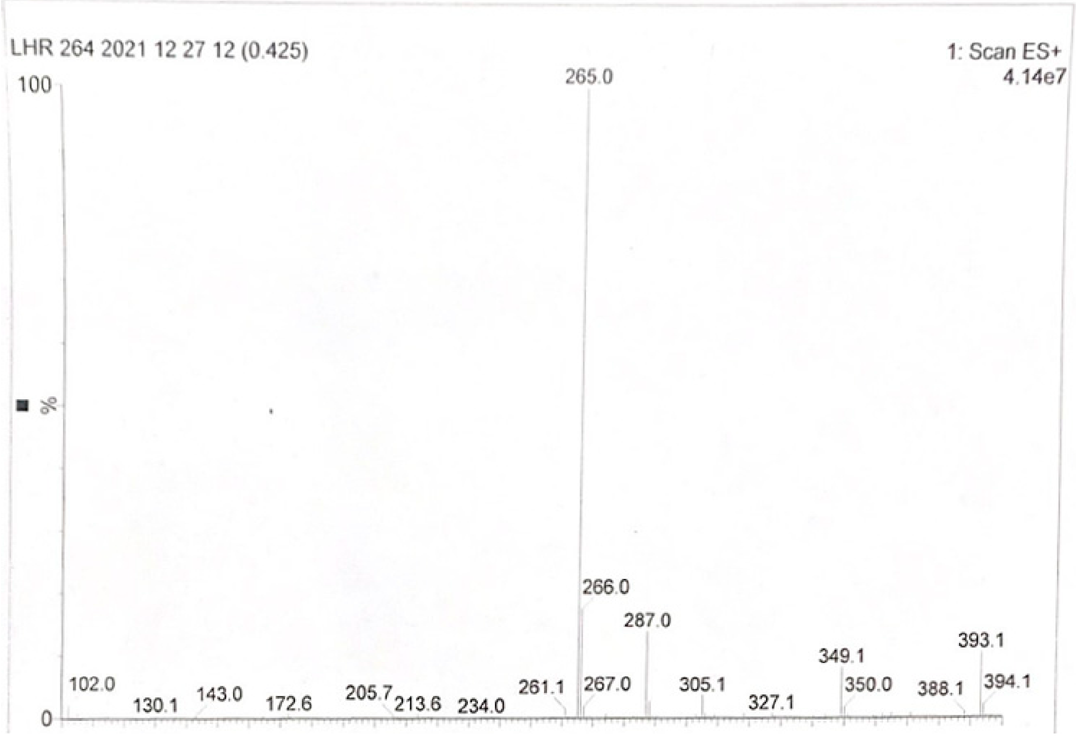

Figure S12. MS (ESI) of the target compound **F02.**

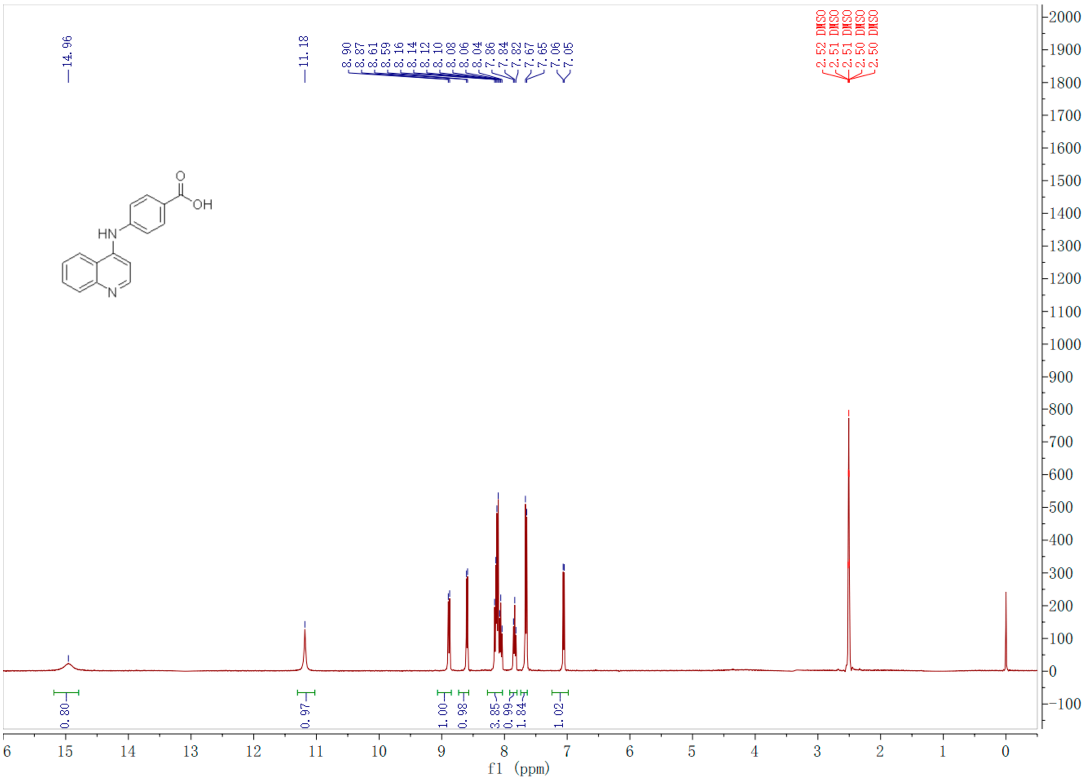

Figure S13. ^{1}H NMR spectra of the target compound **F02.**

Chemical Formula: $C_{17}H_{14}N_2O_2$
MS (ESI): calcd for 279.1 ([M+H]$^+$)

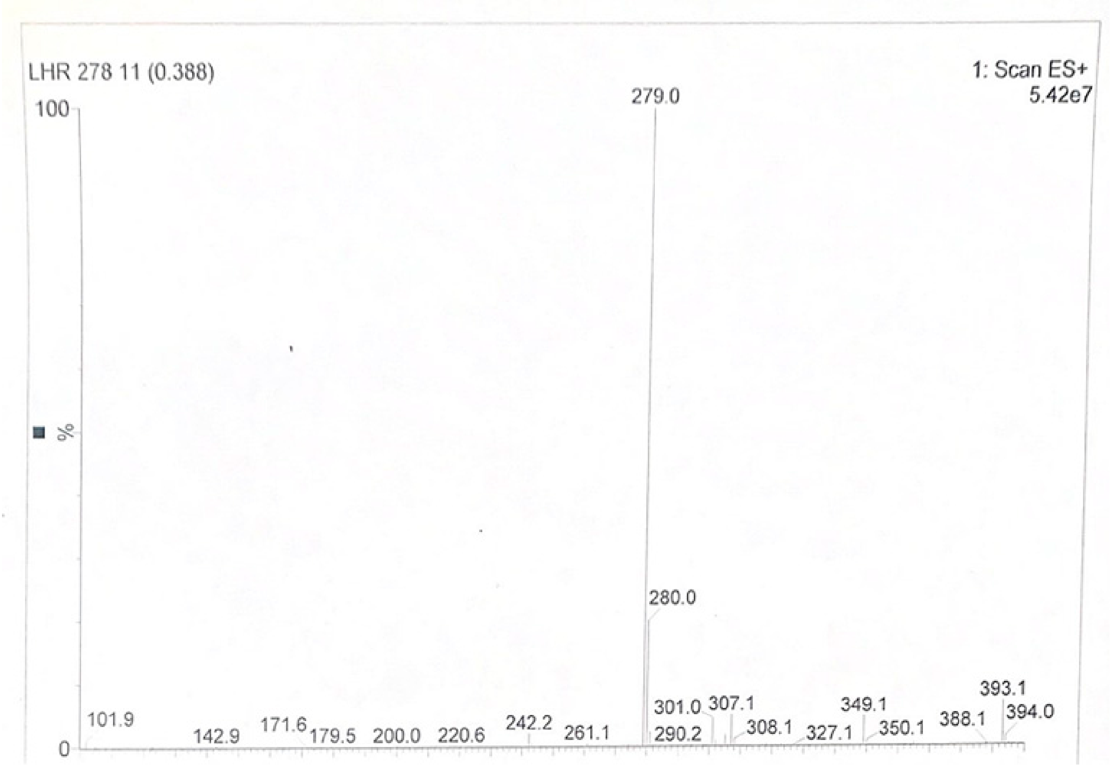

Figure S14. MS (ESI) of the target compound **F03.**

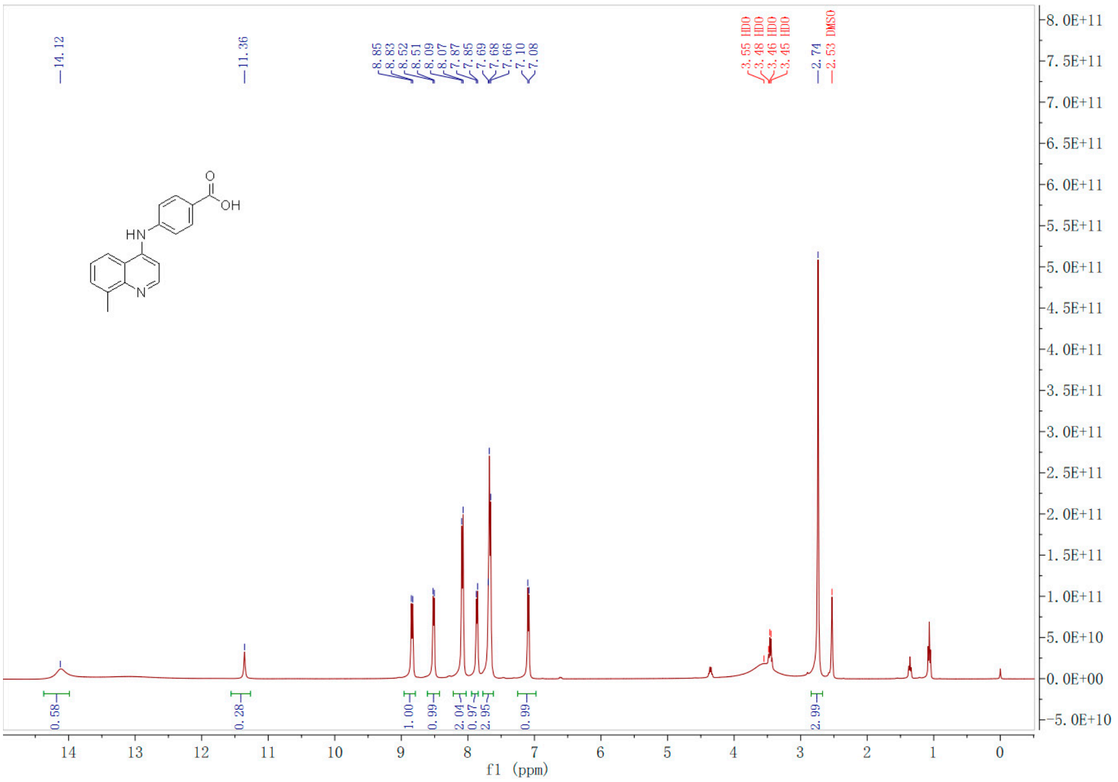

Figure S15. ^{1}H NMR spectra of the target compound **F03.**

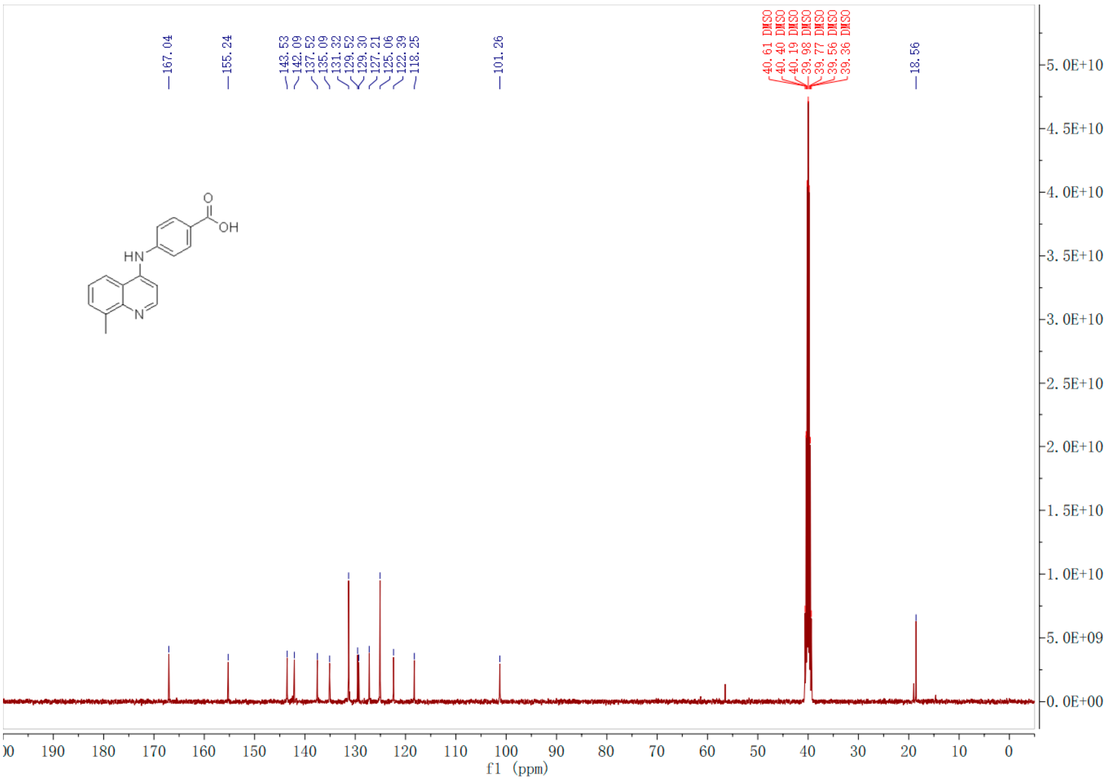

Figure S16. ^{13}C NMR spectra of the target compound **F03.**

Chemical Formula: $C_{17}H_{14}N_2O_2$
MS (ESI): calcd for 279.1 ($[M+H]^+$)

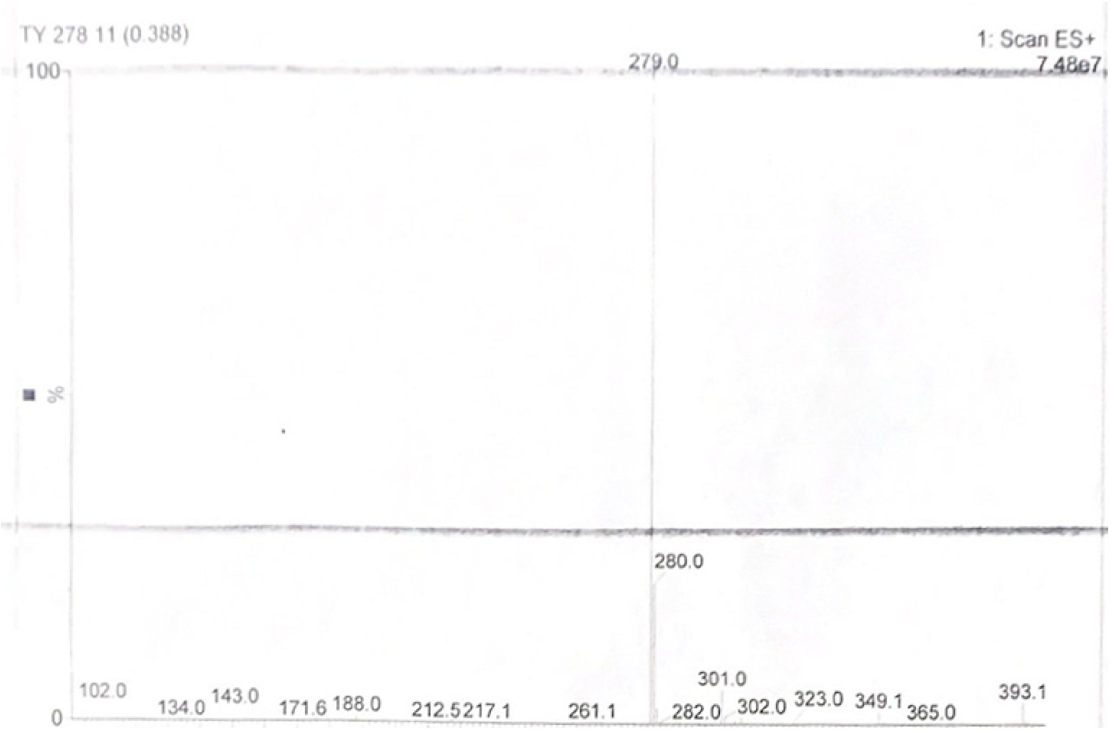

Figure S17. MS (ESI) of the target compound **F04.**

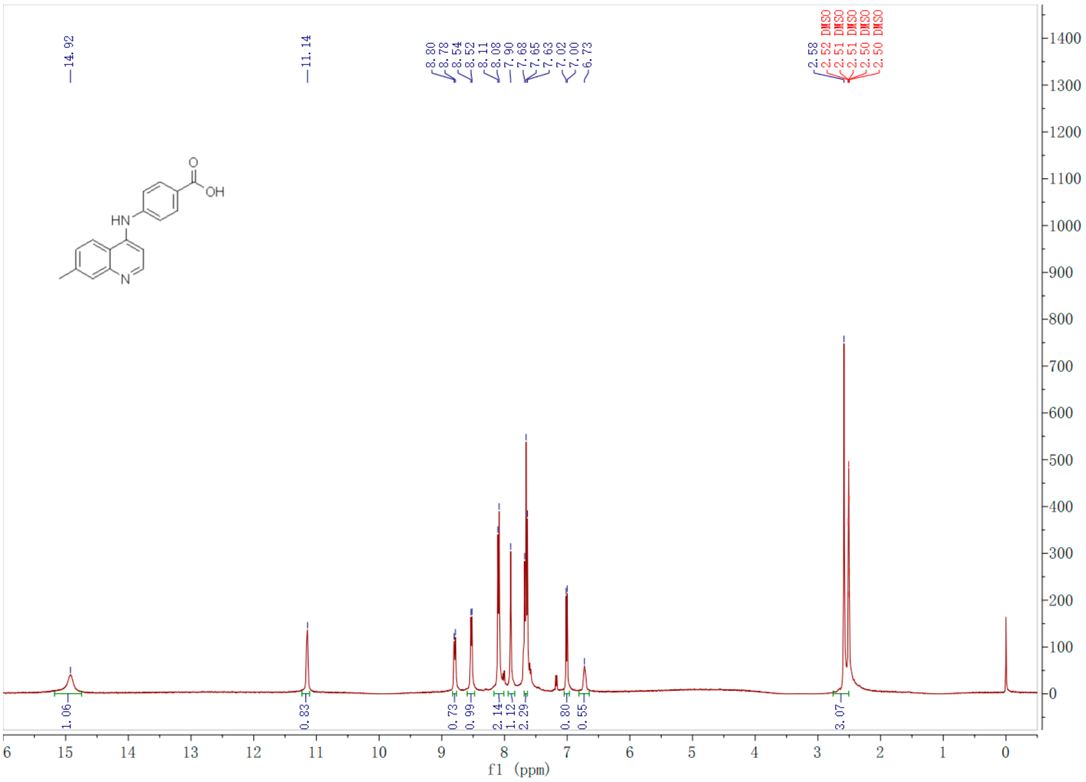

Figure S18. [1] H NMR spectra of the target compound **F04.**

Chemical Formula: $C_{16}H_{11}N_3O_4$
MS (ESI): calcd for 310.1 ([M+H]$^+$)

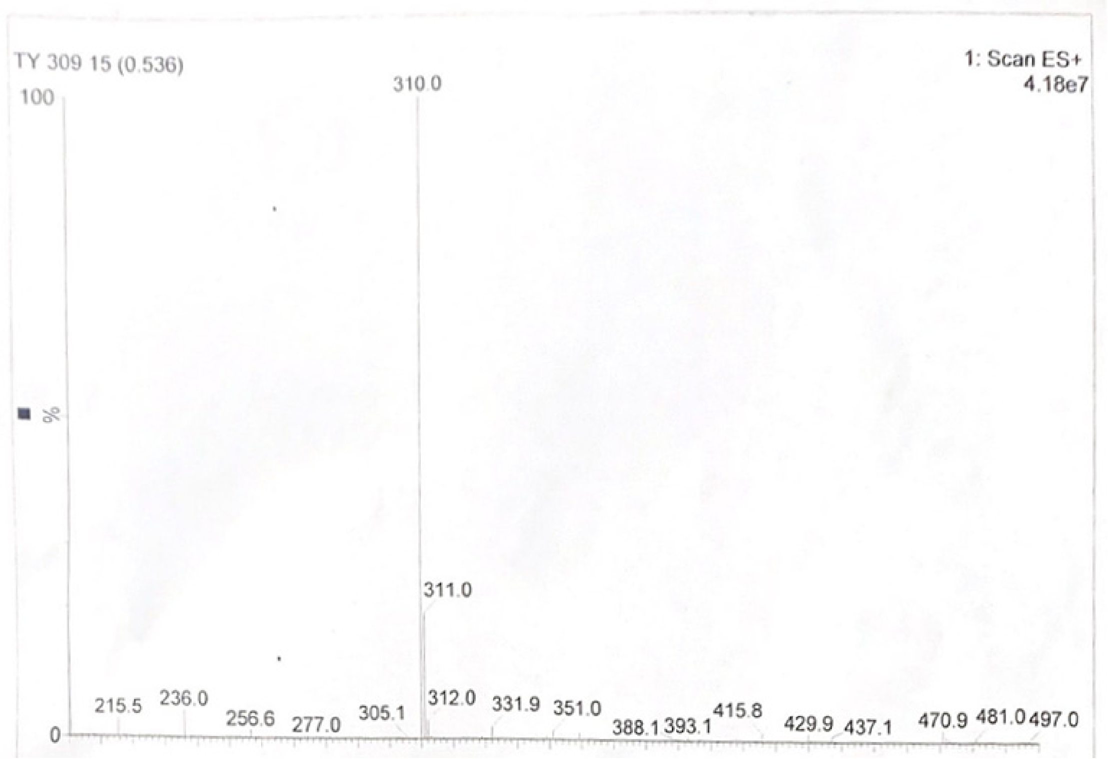

Figure S19. MS (ESI) of the target compound **F05.**

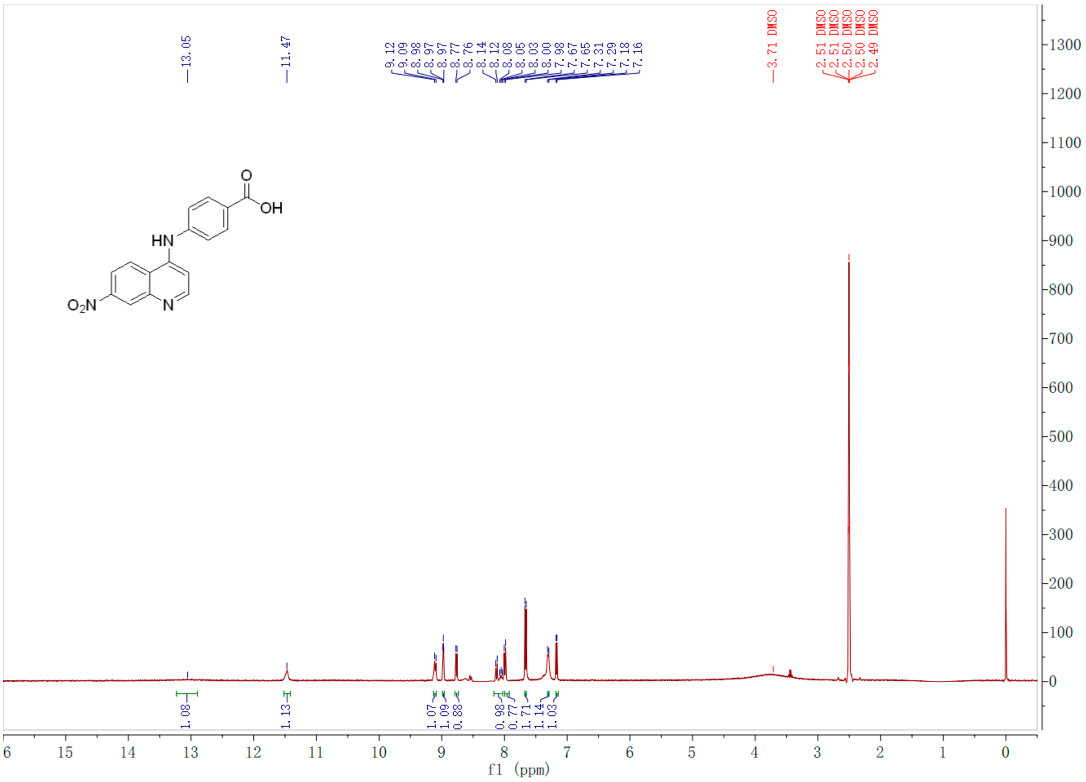

Figure S20. [1] H NMR spectra of the target compound **F05.**

Chemical Formula: $C_{16}H_{11}ClN_2O_2$
MS (ESI): calcd for 299.1 ([M+H]$^+$)

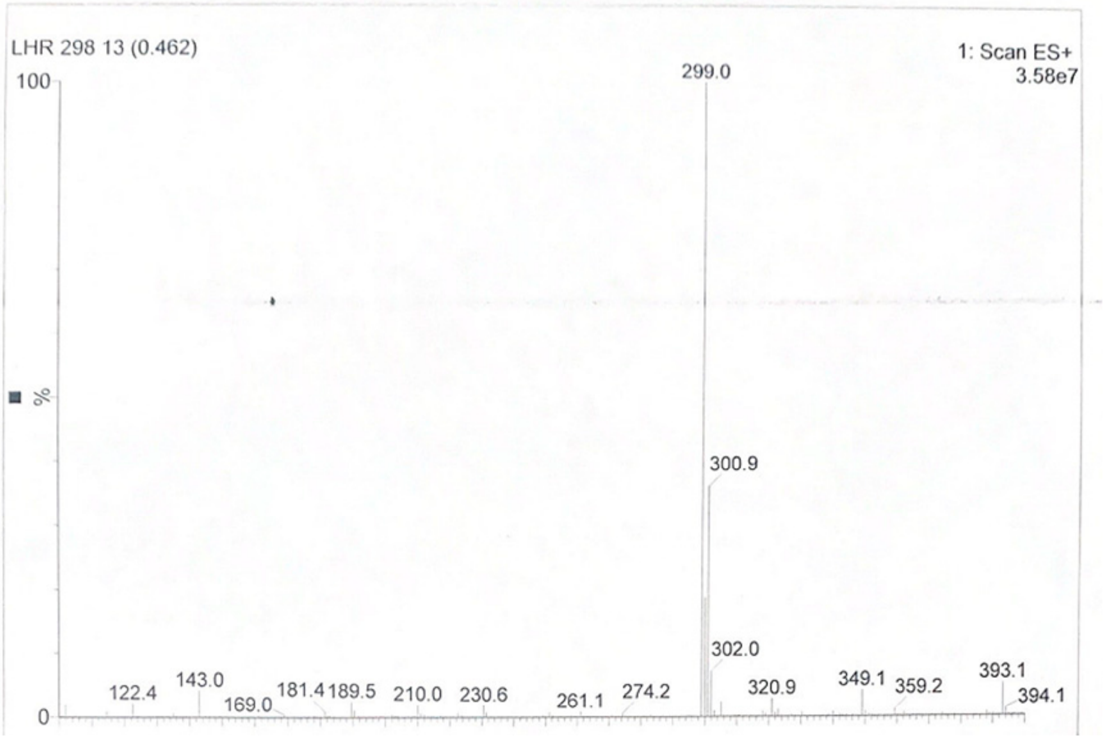

Figure S21. MS (ESI) of the target compound **F06.**

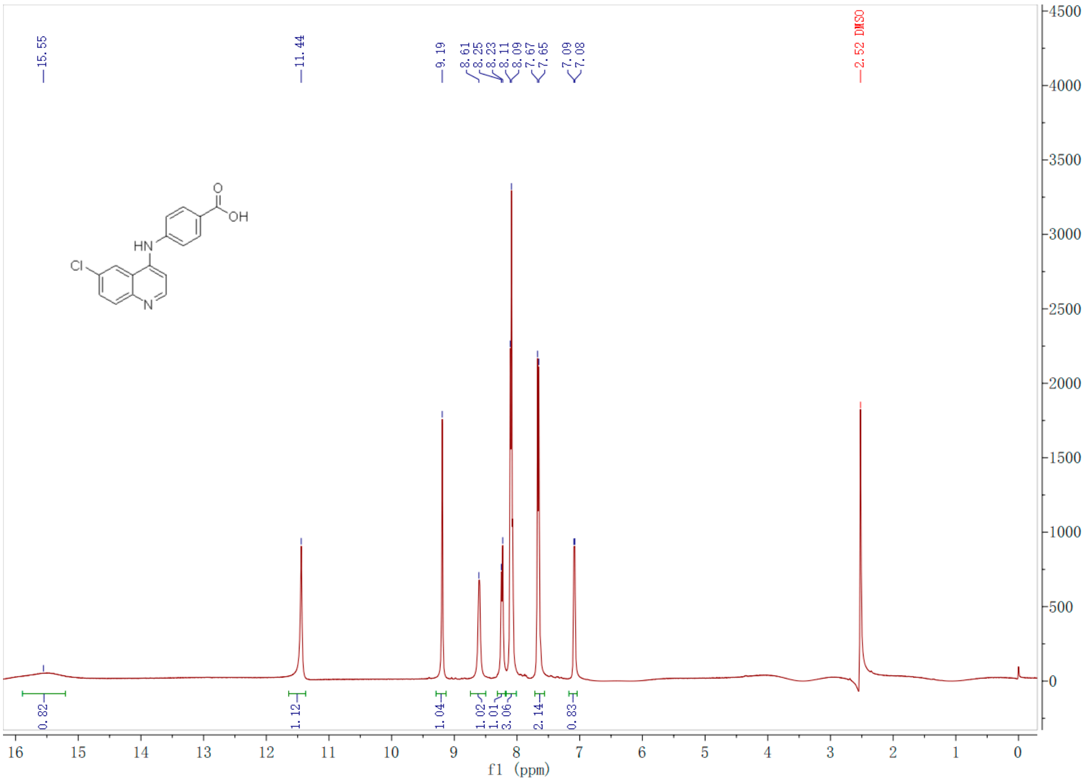

Figure S22. [1] H NMR spectra of the target compound **F06.**

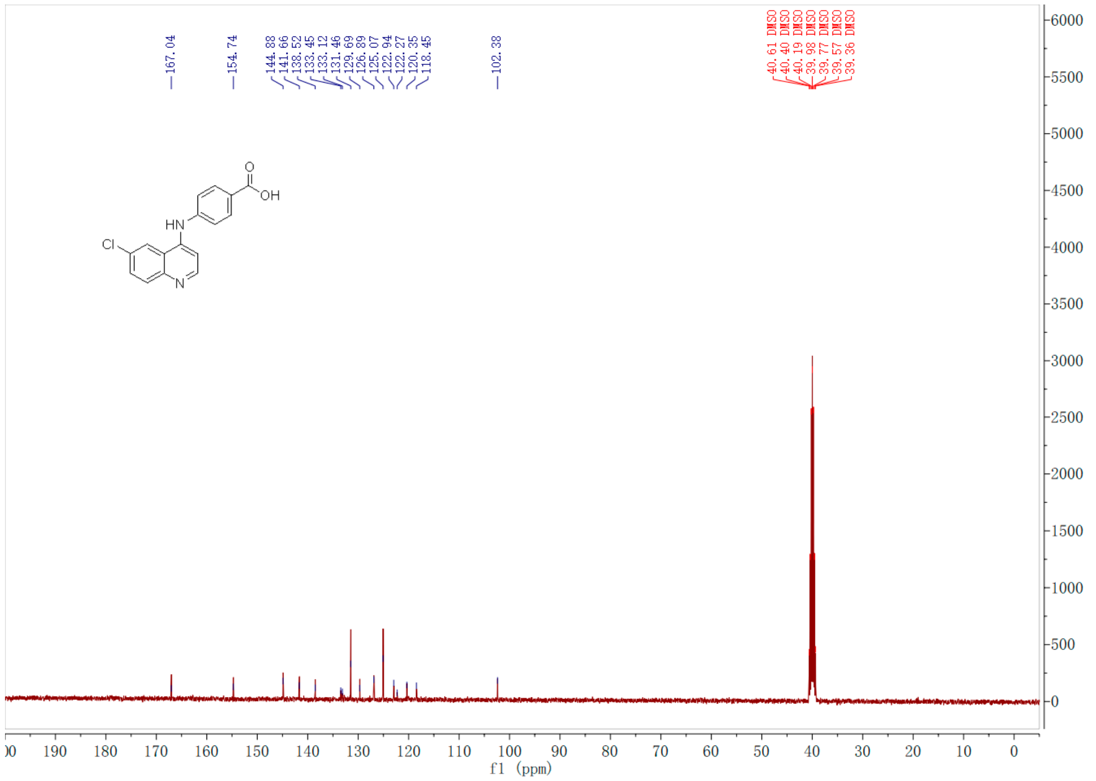

Figure S23. ^{13}C NMR spectra of the target compound **F06.**

Chemical Formula: $C_{16}H_{11}BrN_2O_2$
MS (ESI): calcd for 342.0 ($[M+H]^+$)

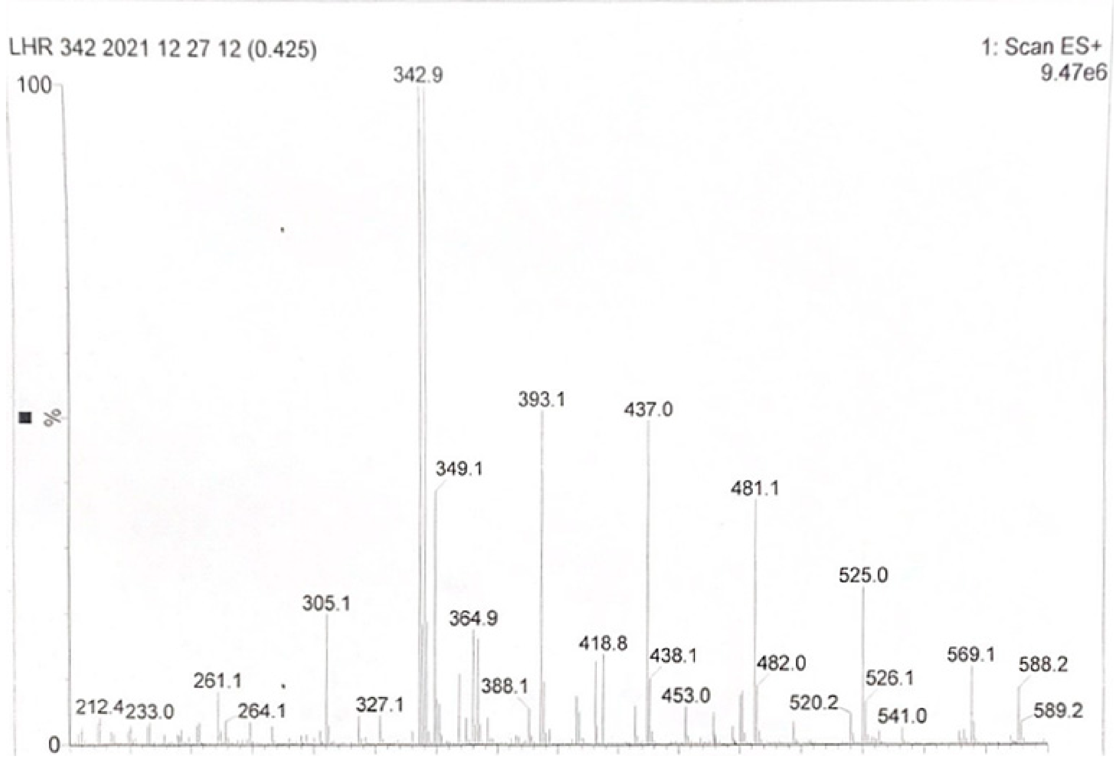

Figure S24. MS (ESI) of the target compound **F07.**

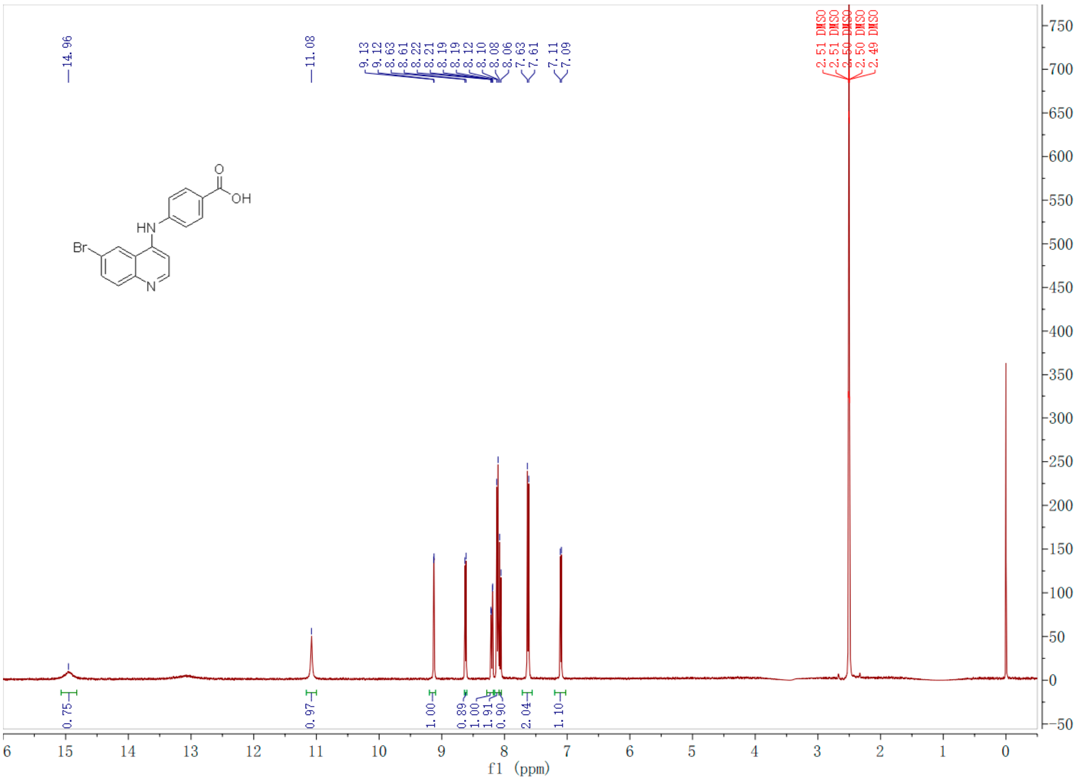

Figure S25. ^{1}H NMR spectra of the target compound **F07.**

Chemical Formula: $C_{17}H_{11}F_3N_2O_2$
MS (ESI): calcd for 333.1 ($[M+H]^+$)

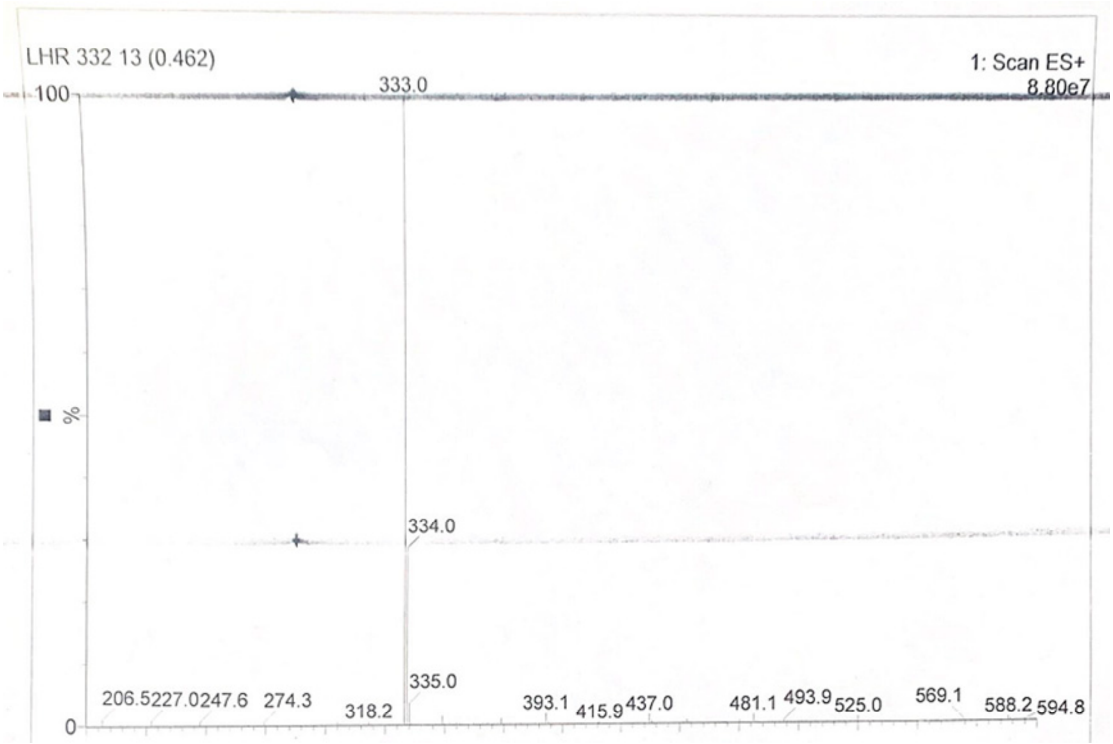

Figure S26. MS (ESI) of the target compound **F08.**

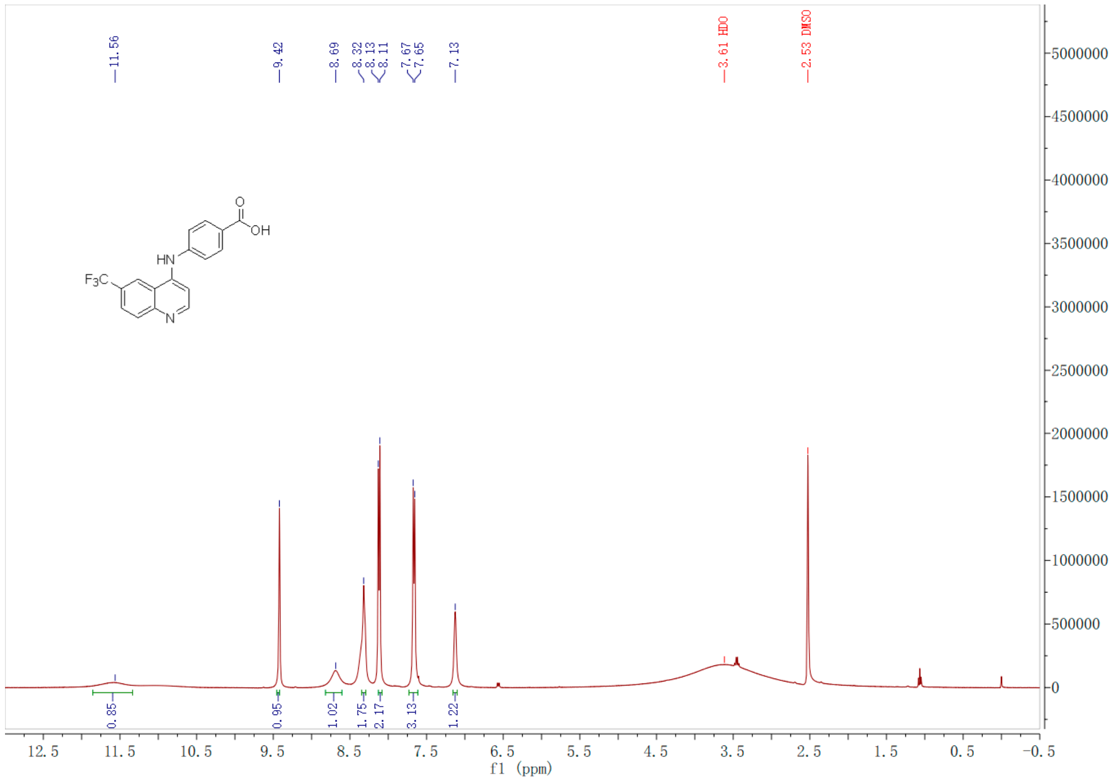

Figure S27. [1] H NMR spectra of the target compound **F08.**

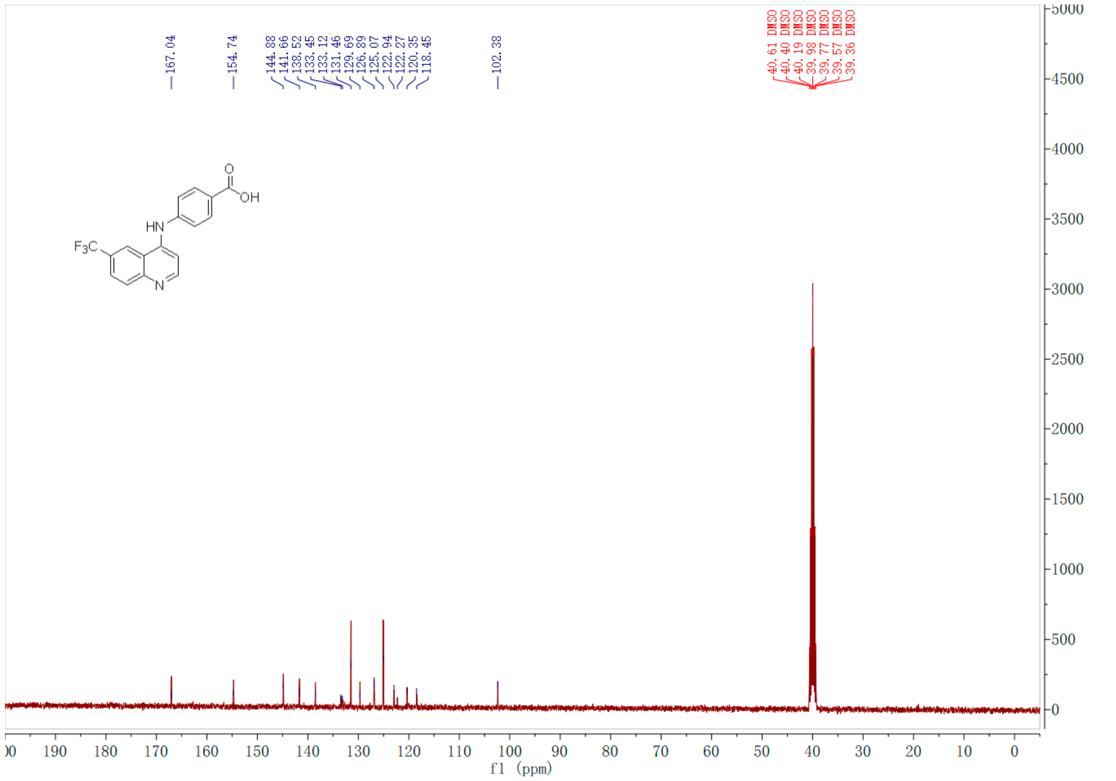

Figure S28. [13] C NMR spectra of the target compound **F08.**

Chemical Formula: $C_{17}H_{11}F_3N_2O_3$
MS (ESI): calcd for 349.1 ([M+H]$^+$)

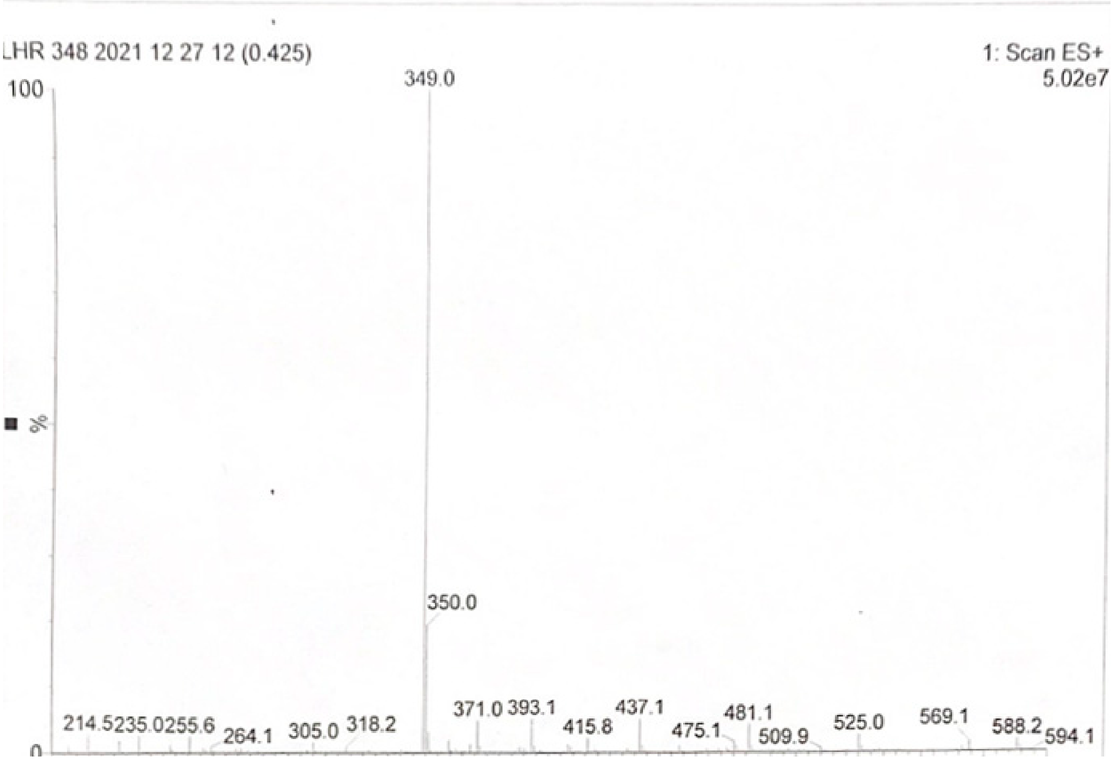

Figure S29. MS (ESI) of the target compound **F09.**

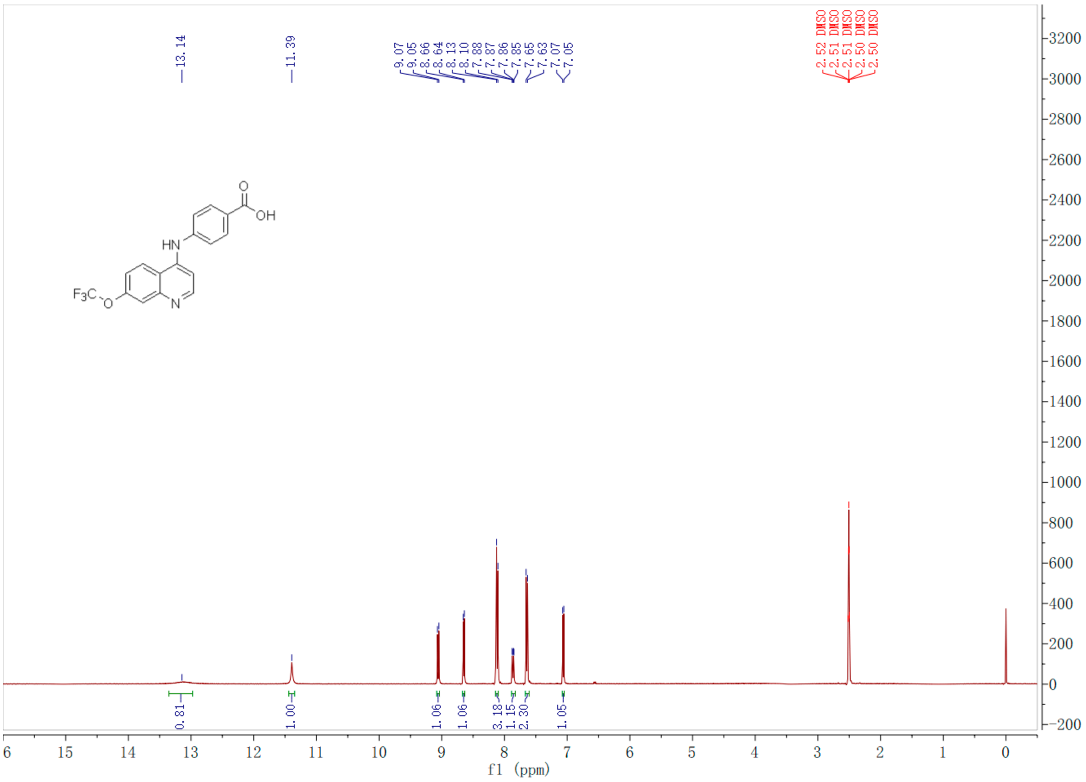

Figure S30. [1] H NMR spectra of the target compound **F09.**

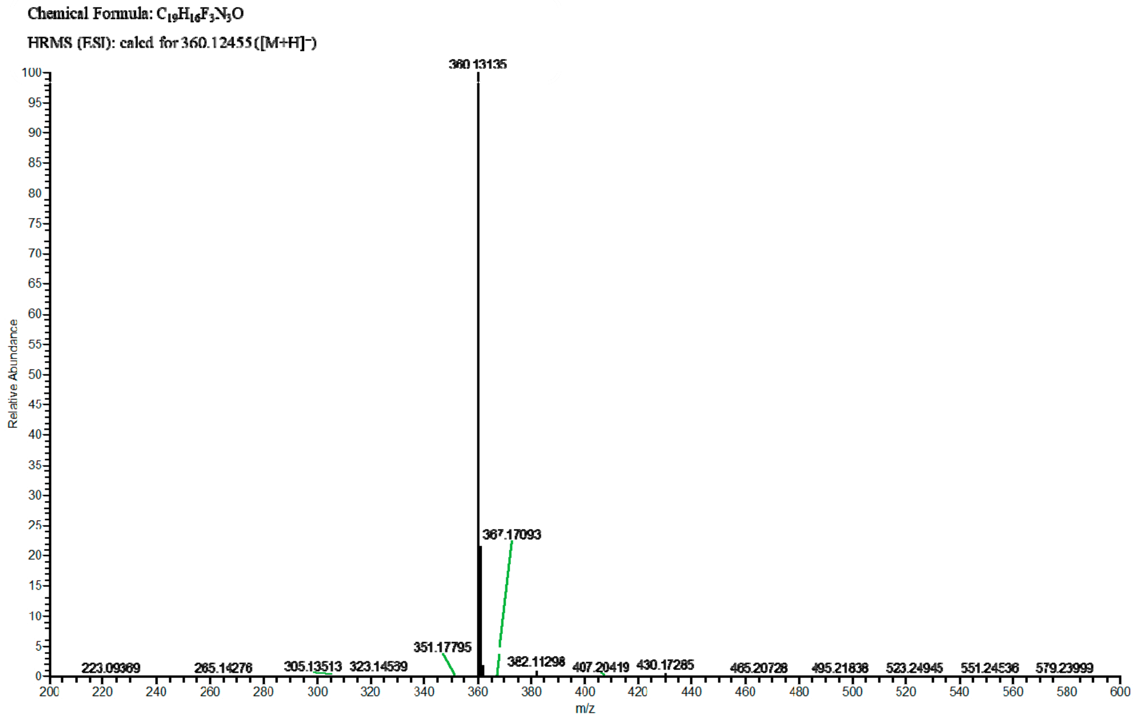

Figure S31. HRMS (ESI) of the target compound **G01**.

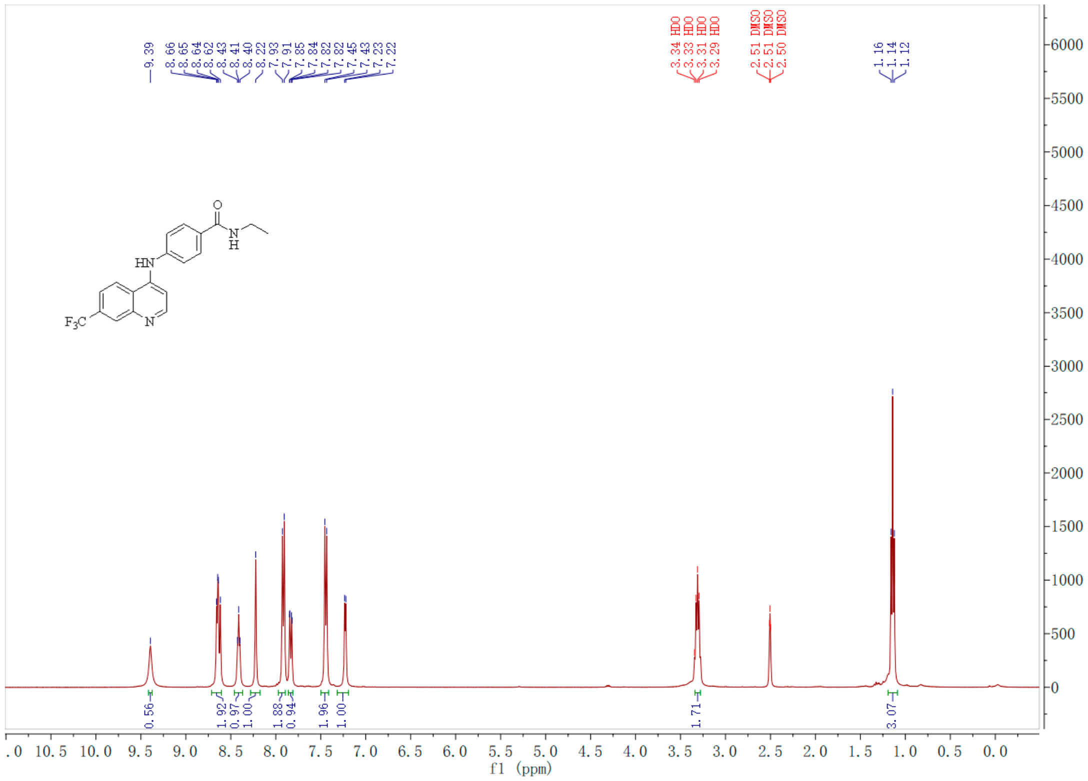

Figure S32. [1] H NMR spectra of the target compound **G01**.

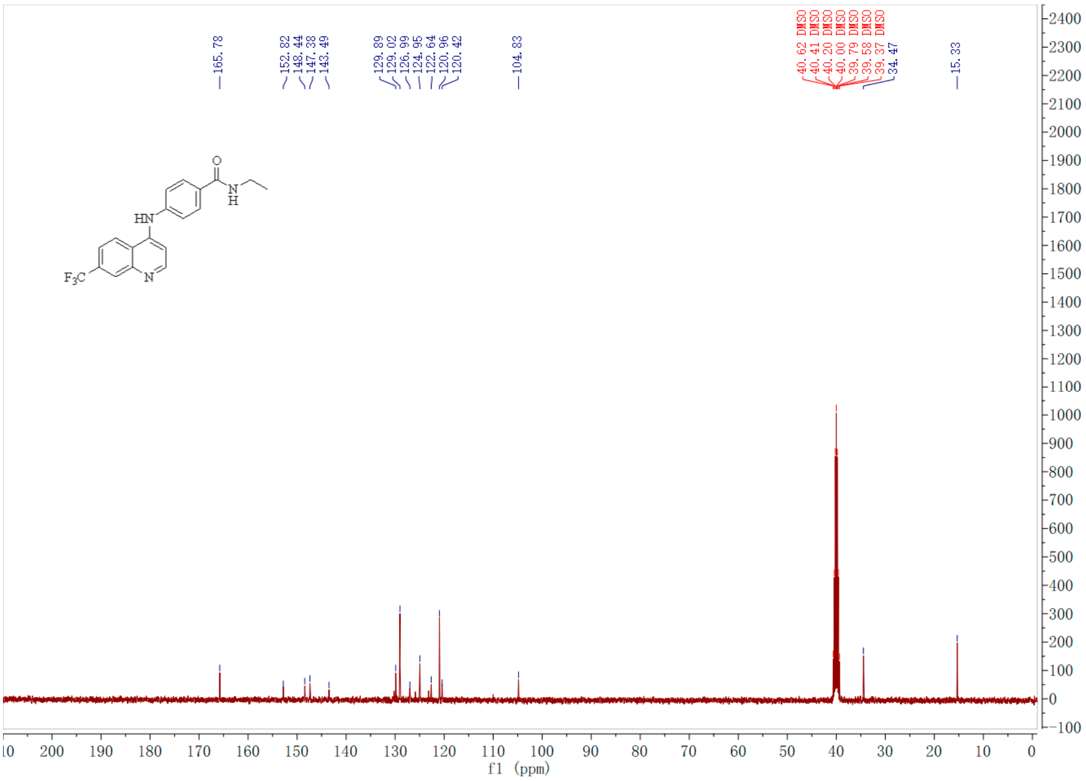

Figure S33. ^{13}C NMR spectra of the target compound **G01**.

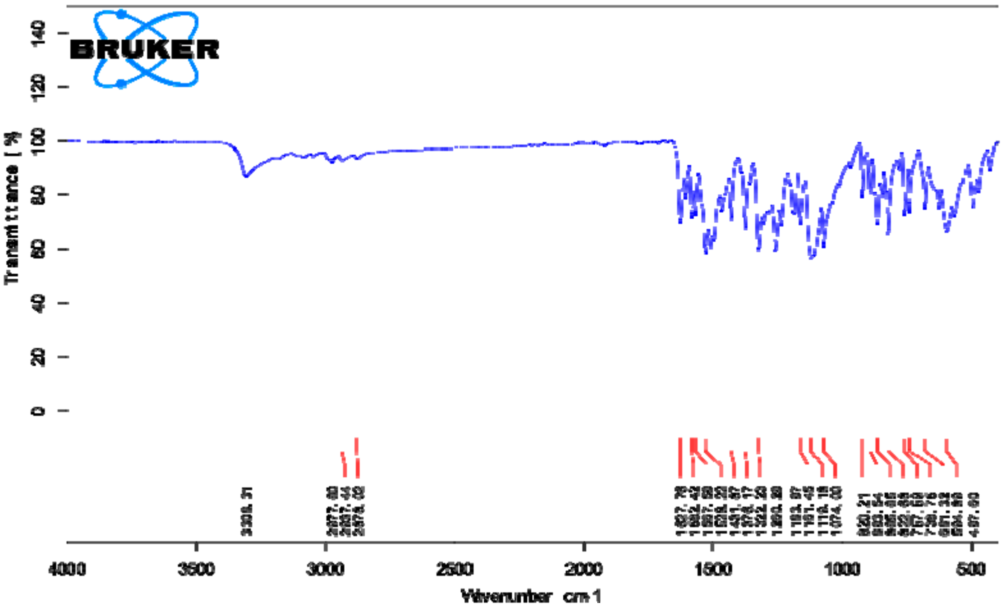

Figure S34. IR spectra of the target compound **G01**.

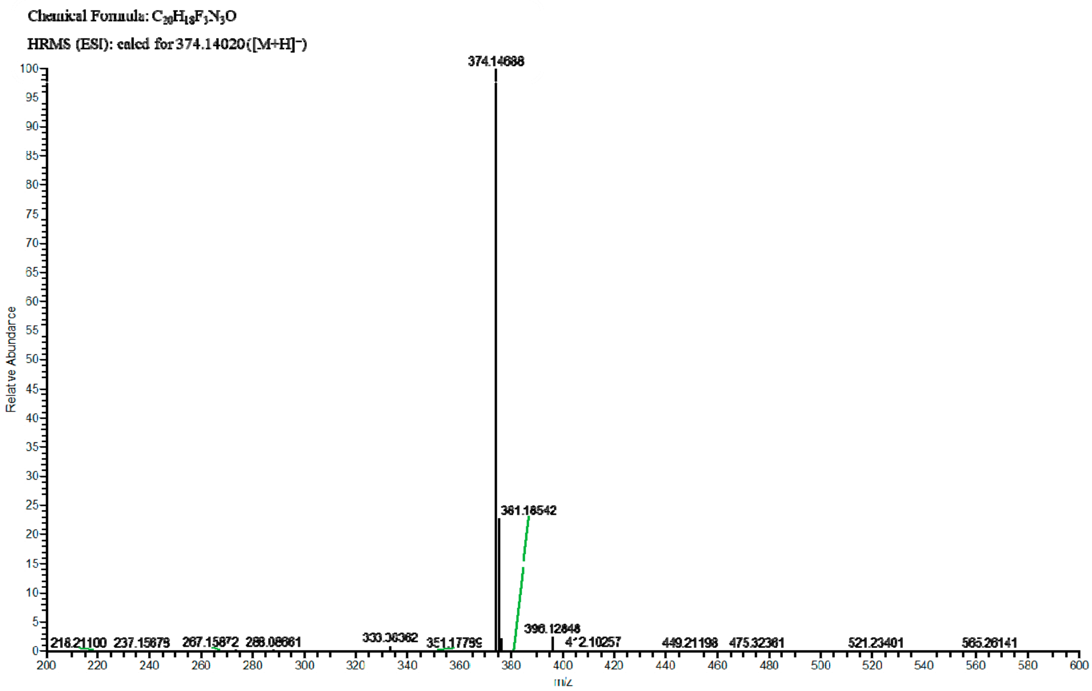

Figure S35. HRMS (ESI) of the target compound **G02**.

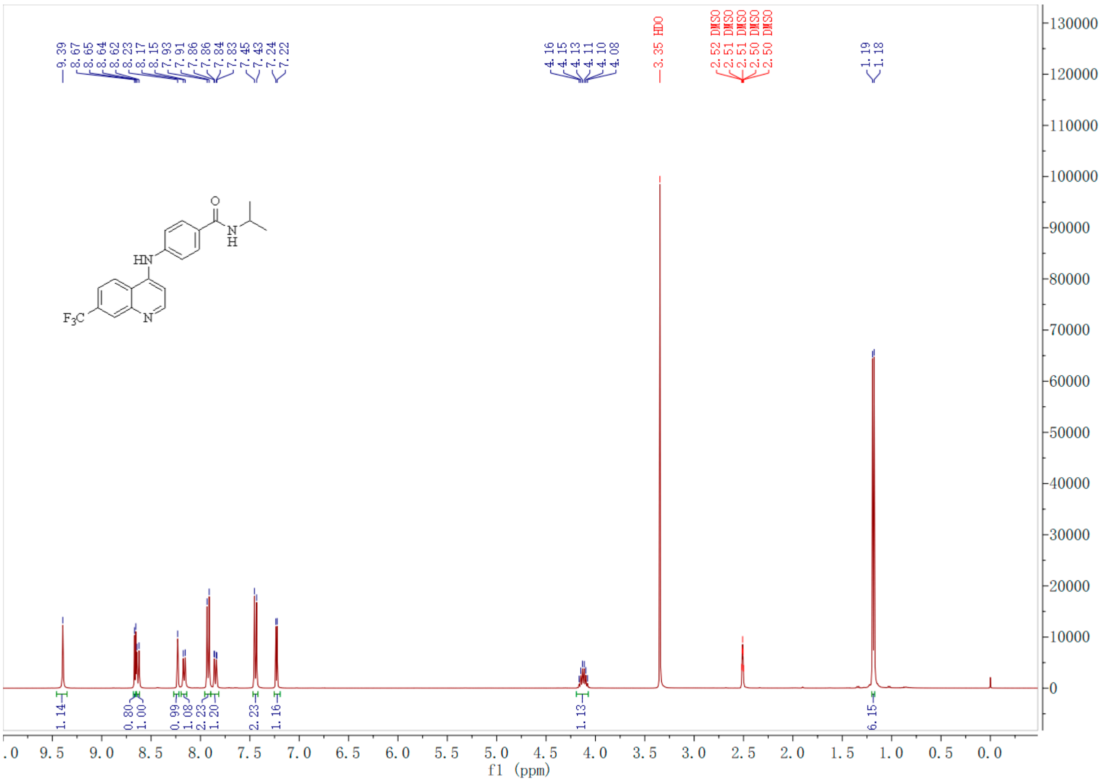

Figure S36. ^{1}H NMR spectra of the target compound **G02**.

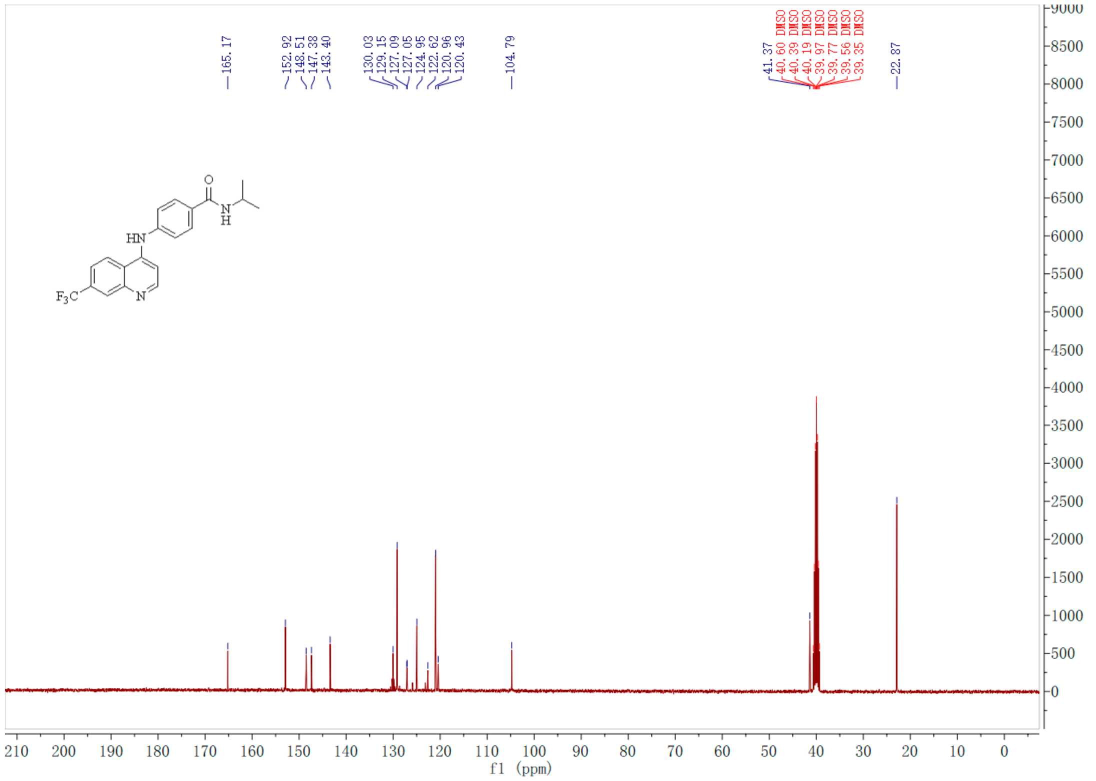

Figure S37. ^{13}C NMR spectra of the target compound **G02**.

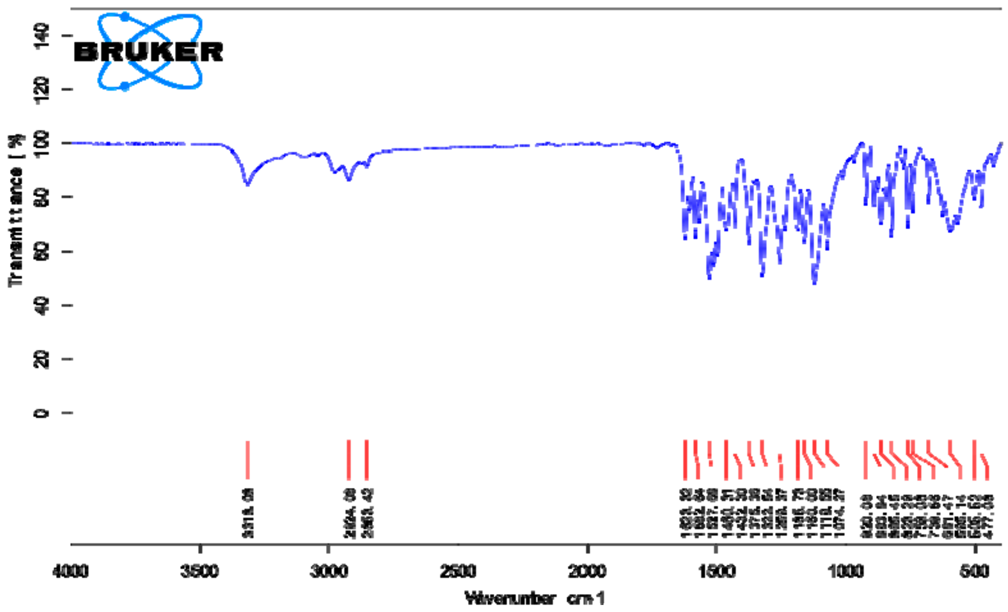

Figure S38. IR spectra of the target compound **G02**.

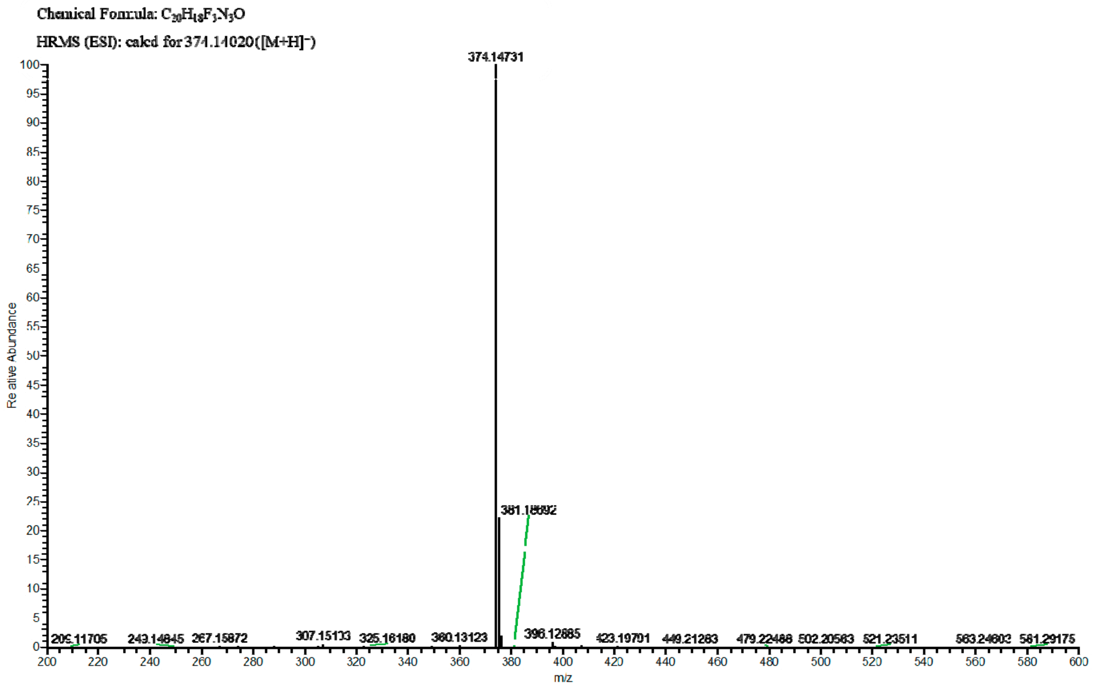

Figure S39. HRMS (ESI) of the target compound **G03**.

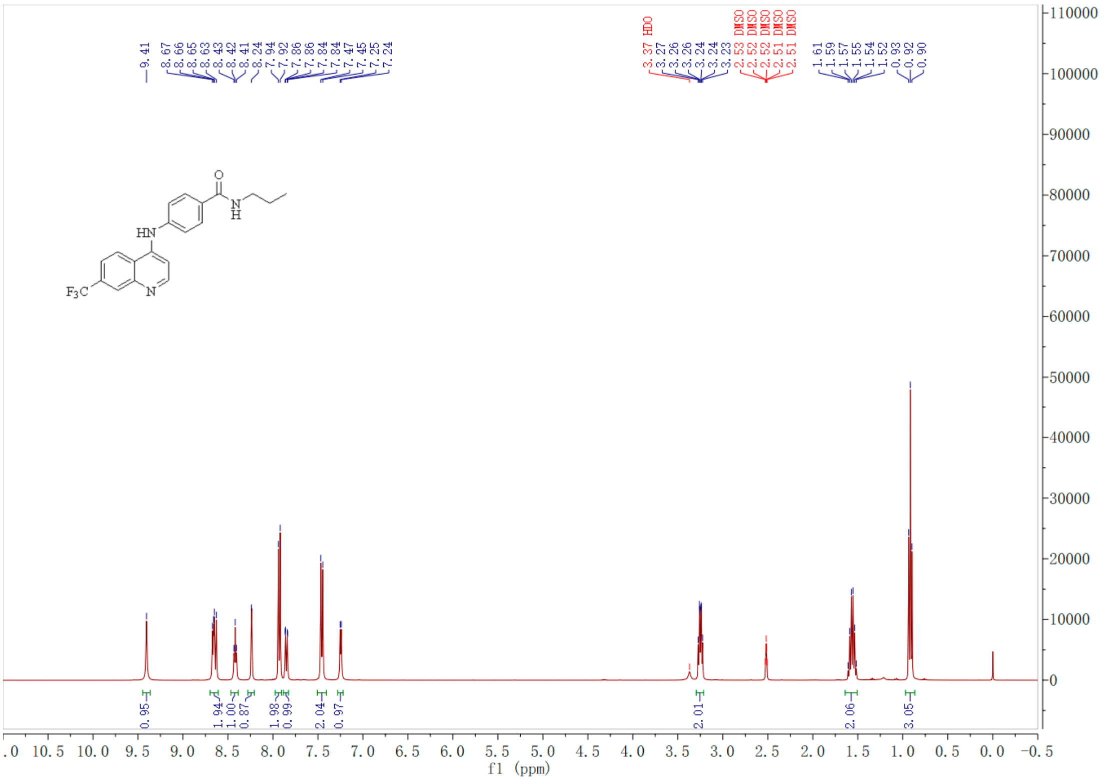

Figure S40. ^{1}H NMR spectra of the target compound **G03**.

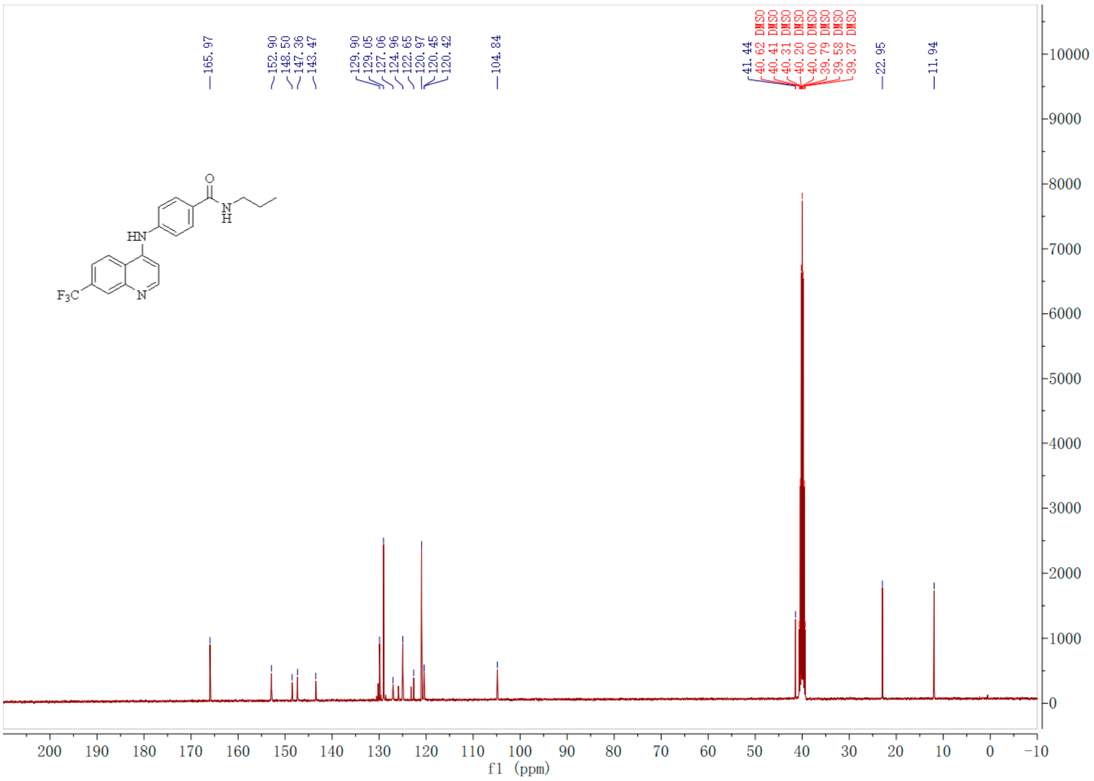

Figure S41. ^{13}C NMR spectra of the target compound **G03**.

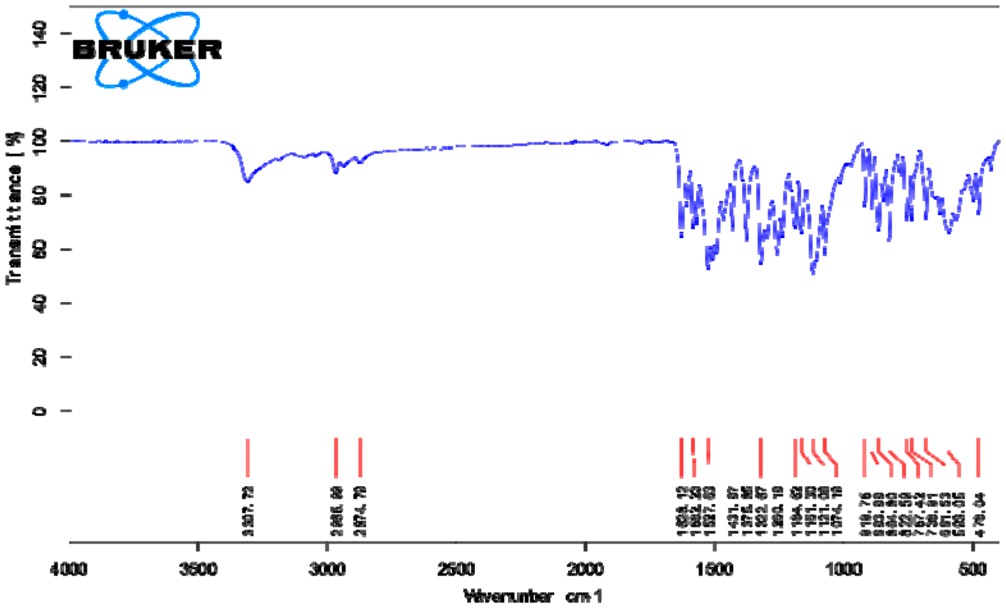

Figure S42. IR spectra of the target compound **G03**.

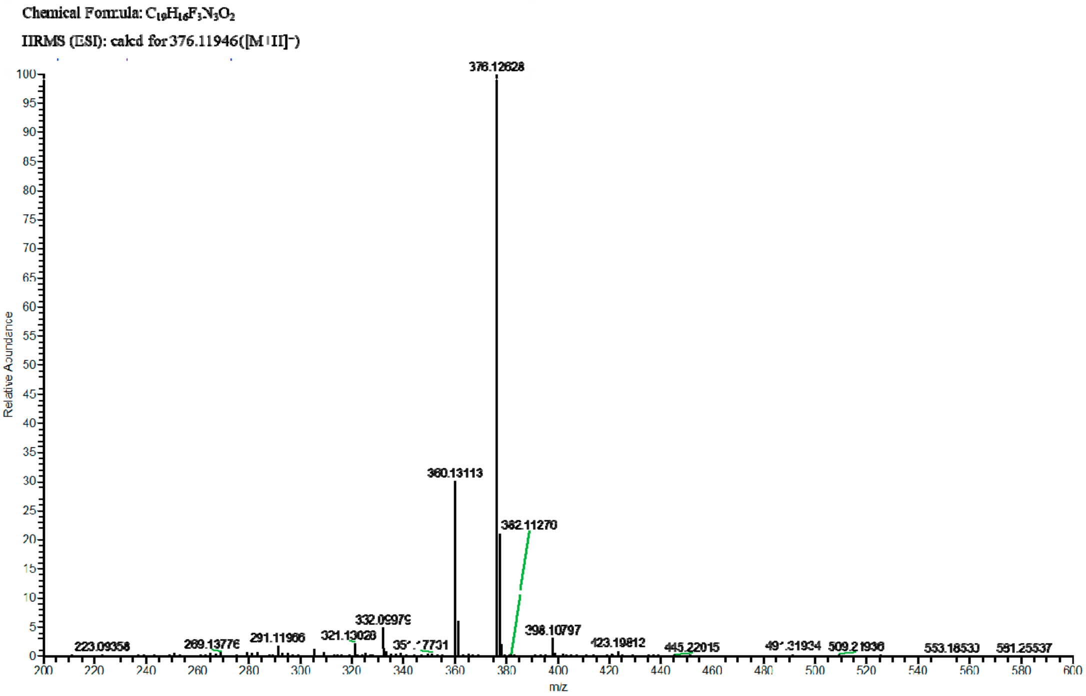

Figure S43. HRMS (ESI) of the target compound **G04**.

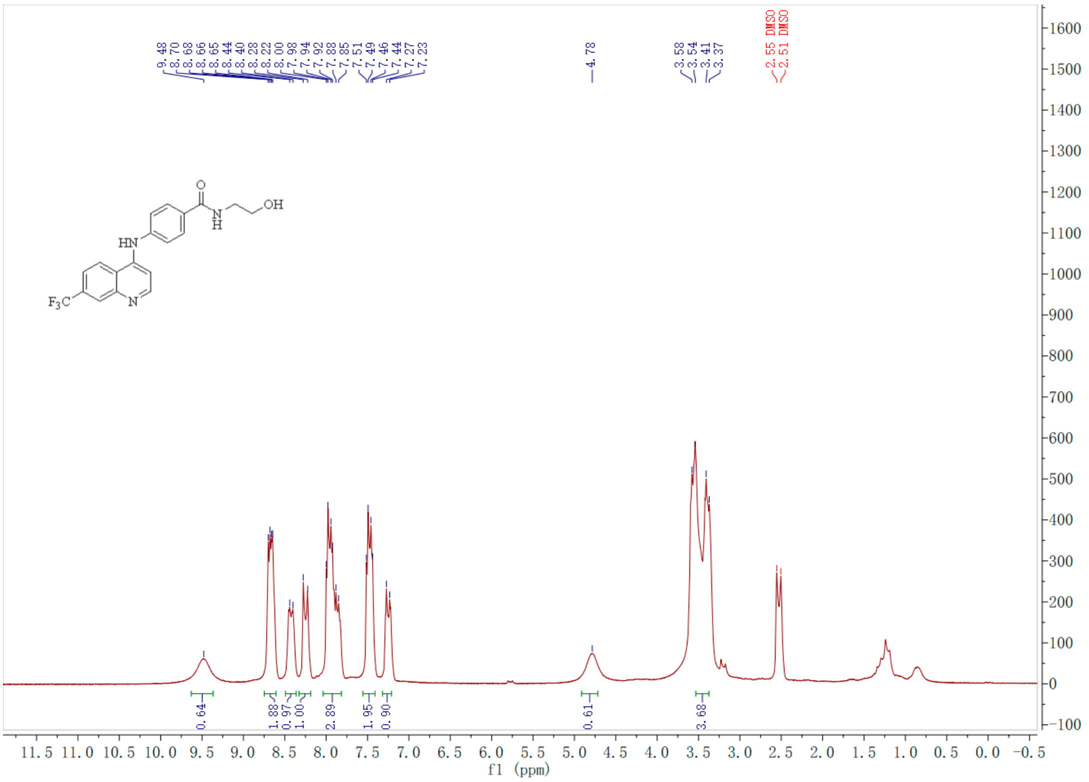

Figure S44. [1] H NMR spectra of the target compound **G04**.

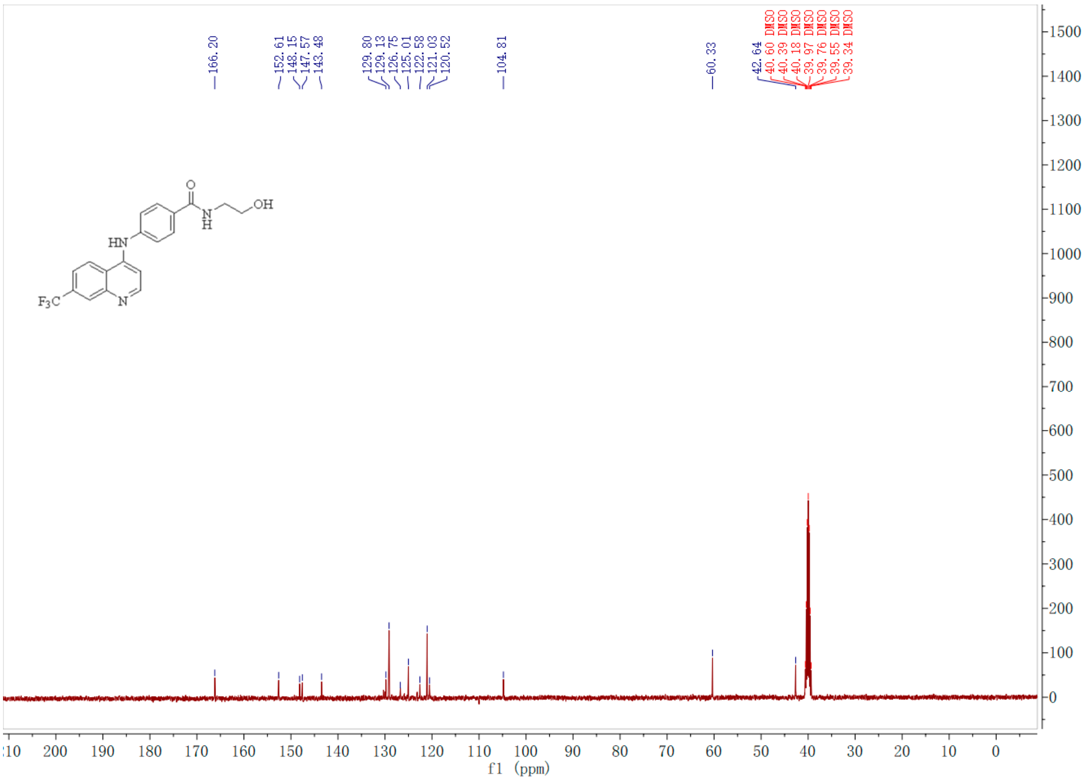

Figure S45. ^{13}C NMR spectra of the target compound **G04**.

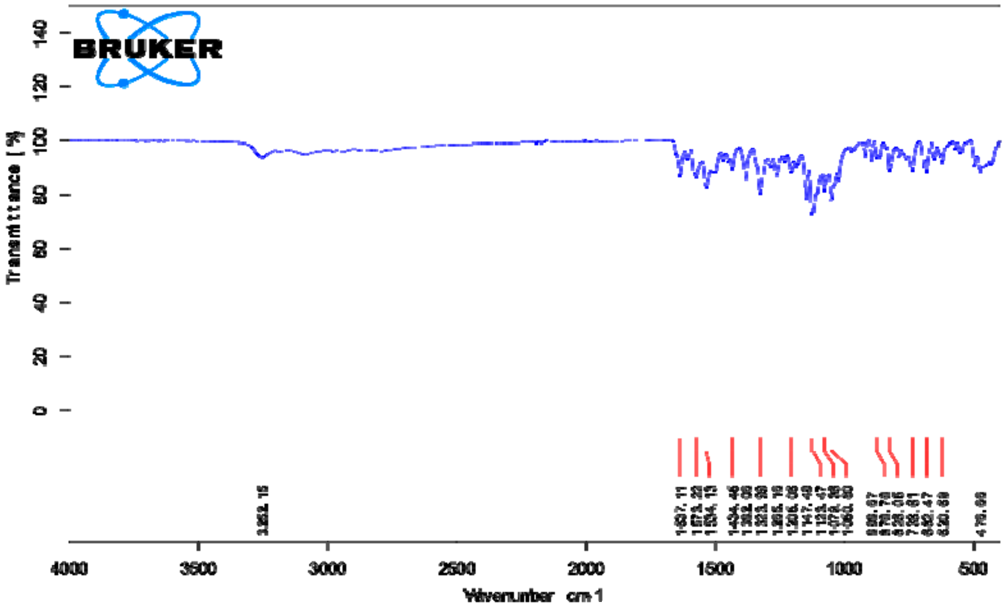

Figure S46. IR spectra of the target compound **G04**.

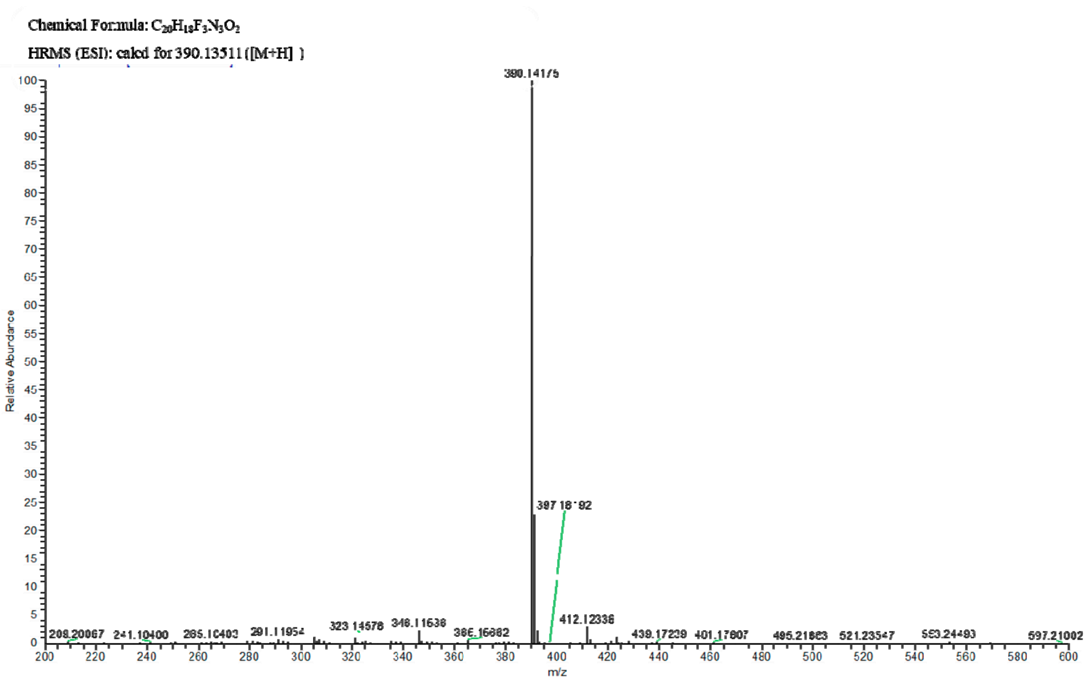

Figure S47. HRMS (ESI) of the target compound **G05**.

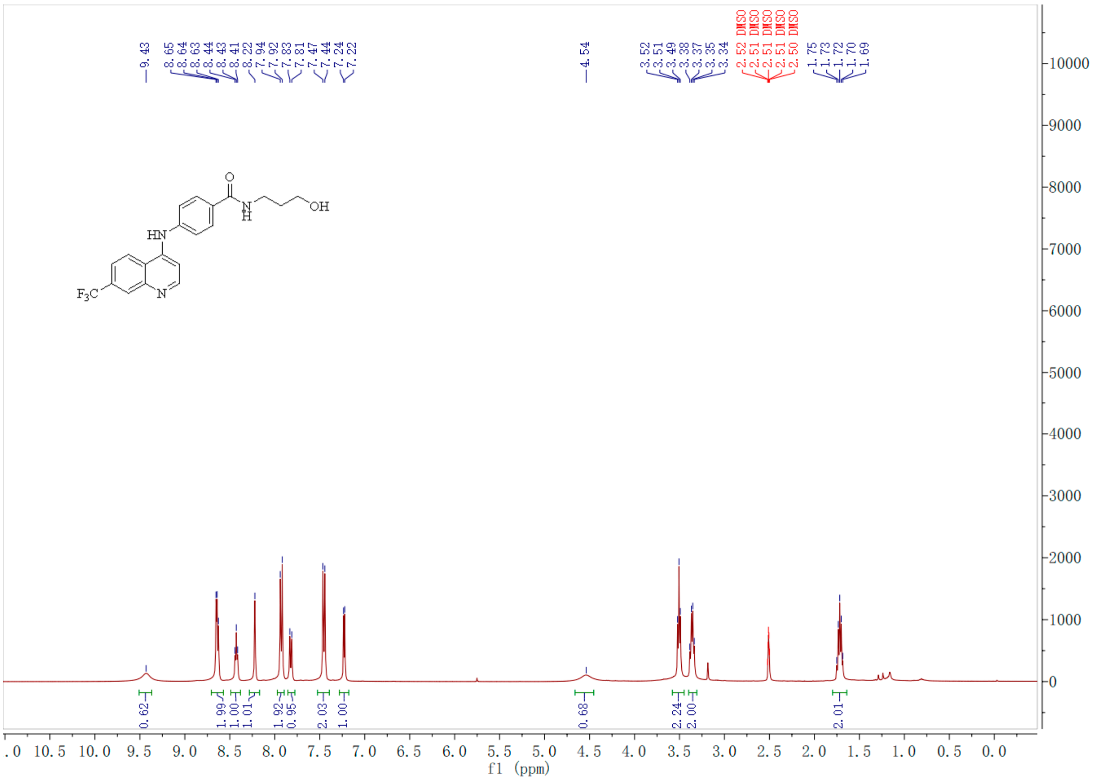

Figure S48. ^{1}H NMR spectra of the target compound **G05**.

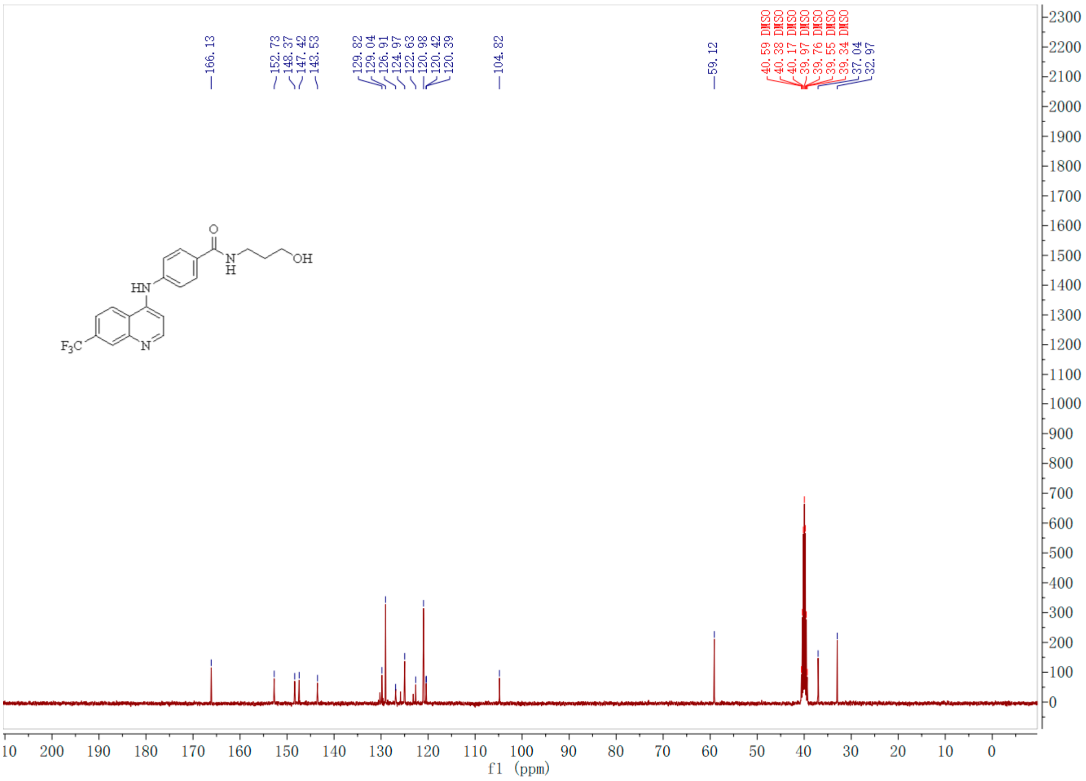

Figure S49. ^{13}C NMR spectra of the target compound **G05**.

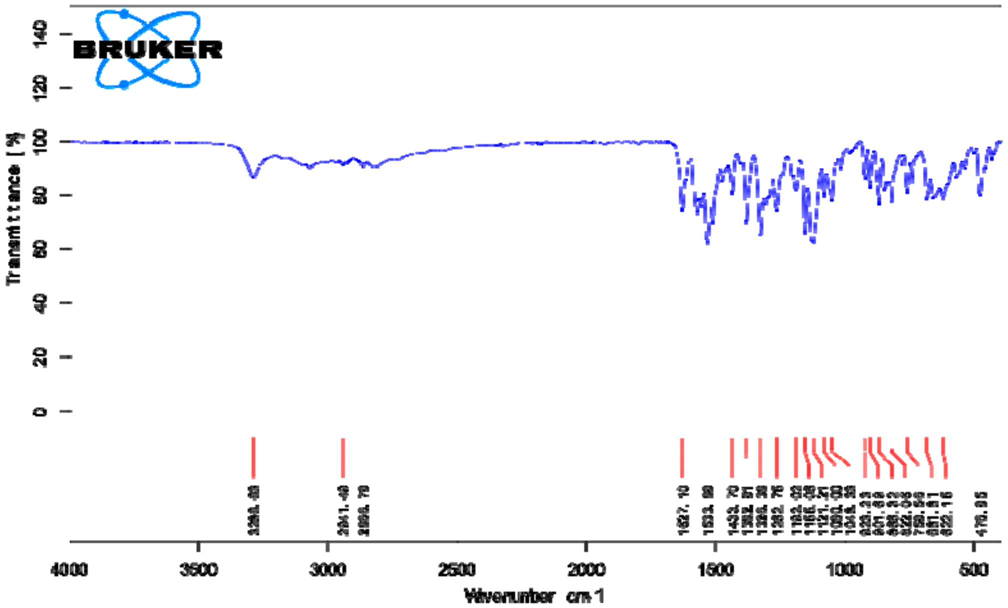

Figure S50. IR spectra of the target compound **G05**.

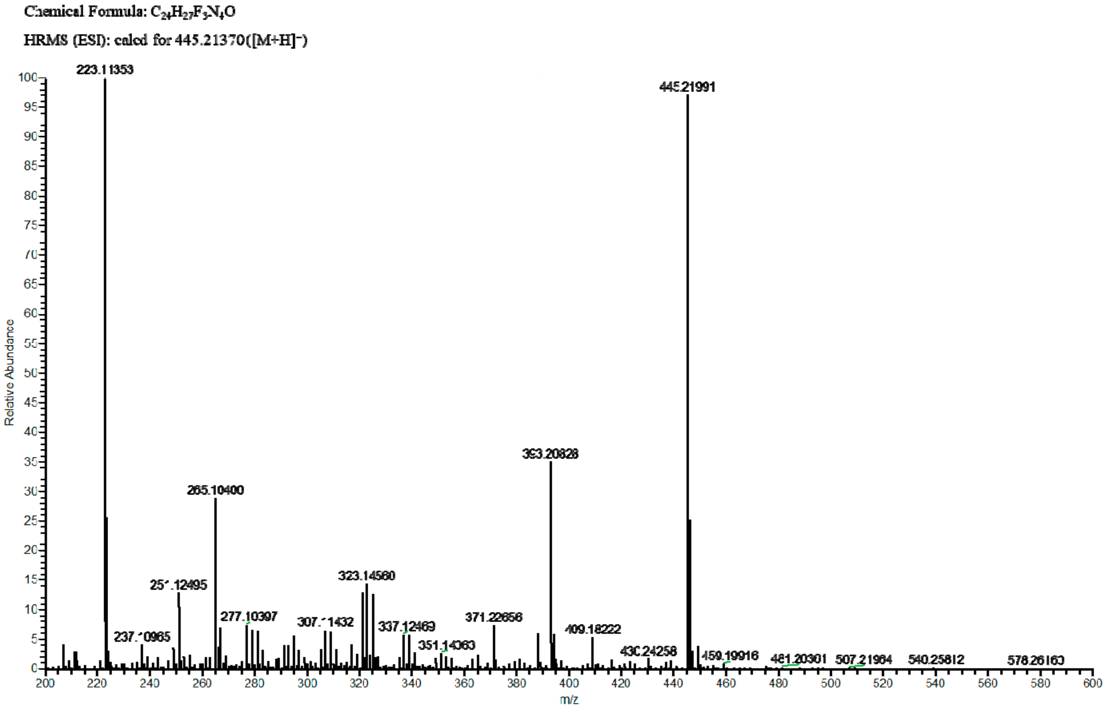

Figure S51. HRMS (ESI) of the target compound **G06**.

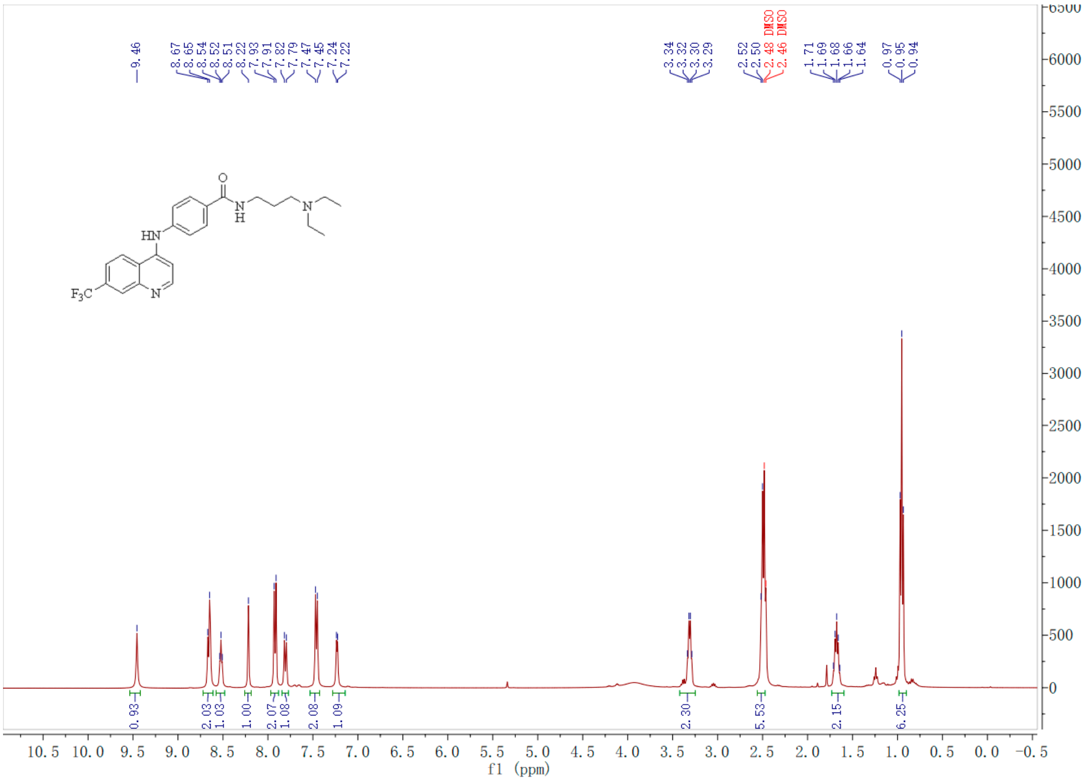

Figure S52. ¹H NMR spectra of the target compound **G06**.

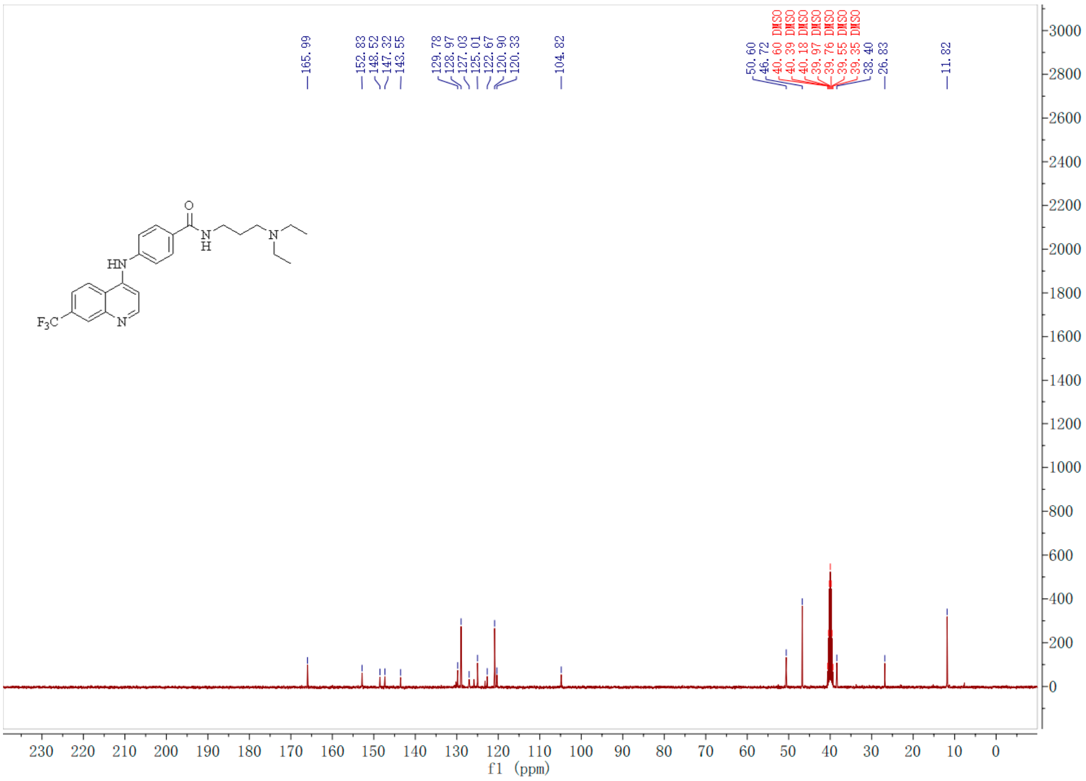

Figure S53. ^{13}C NMR spectra of the target compound **G06**.

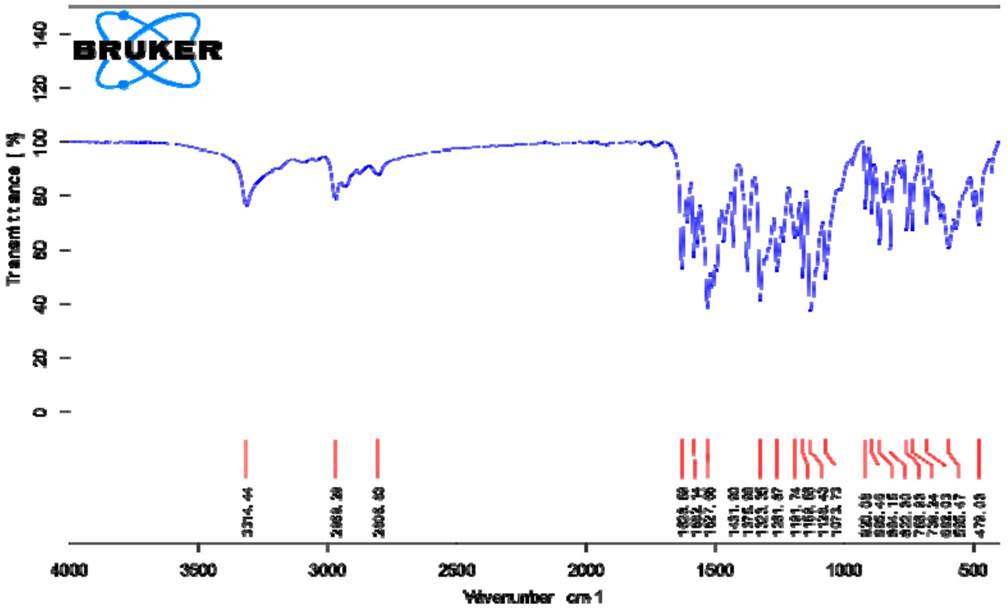

Figure S54. IR spectra of the target compound **G06**.

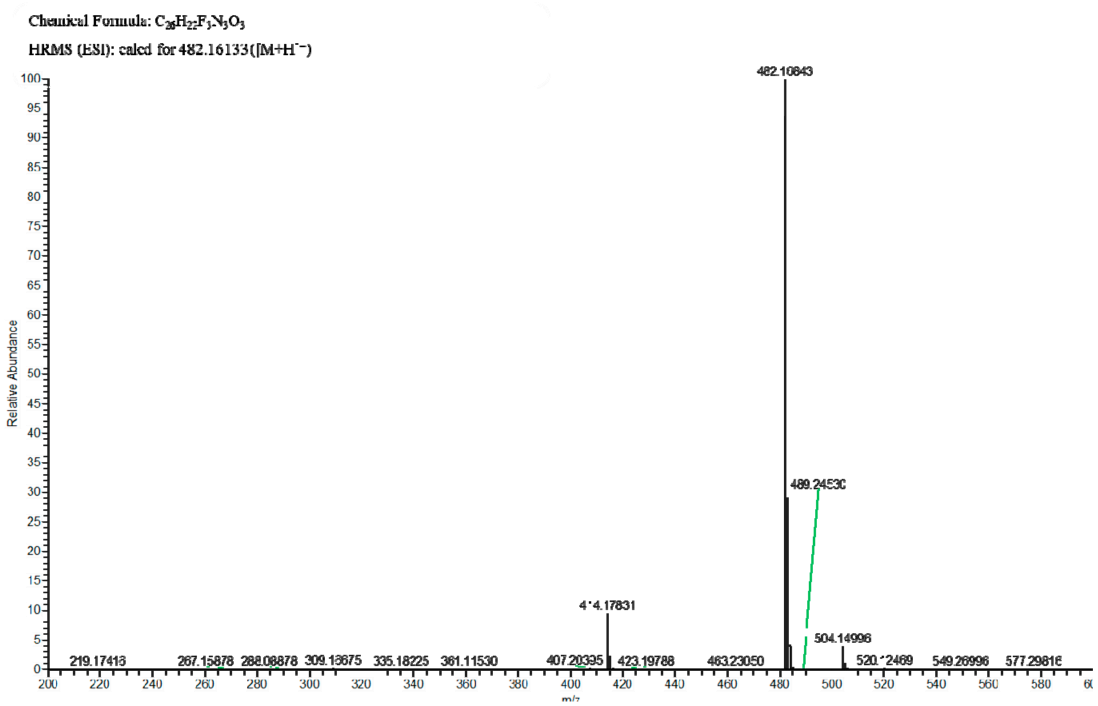

Figure S55. HRMS (ESI) of the target compound **G07**.

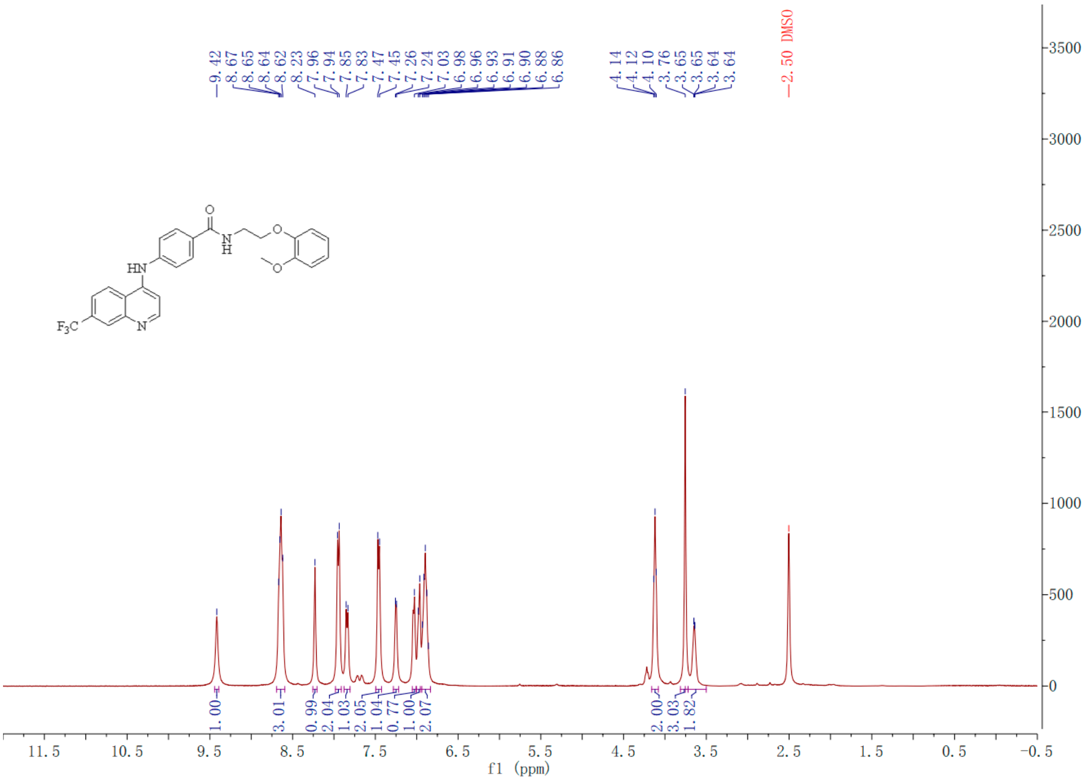

Figure S56. [1] H NMR spectra of the target compound **G07**.

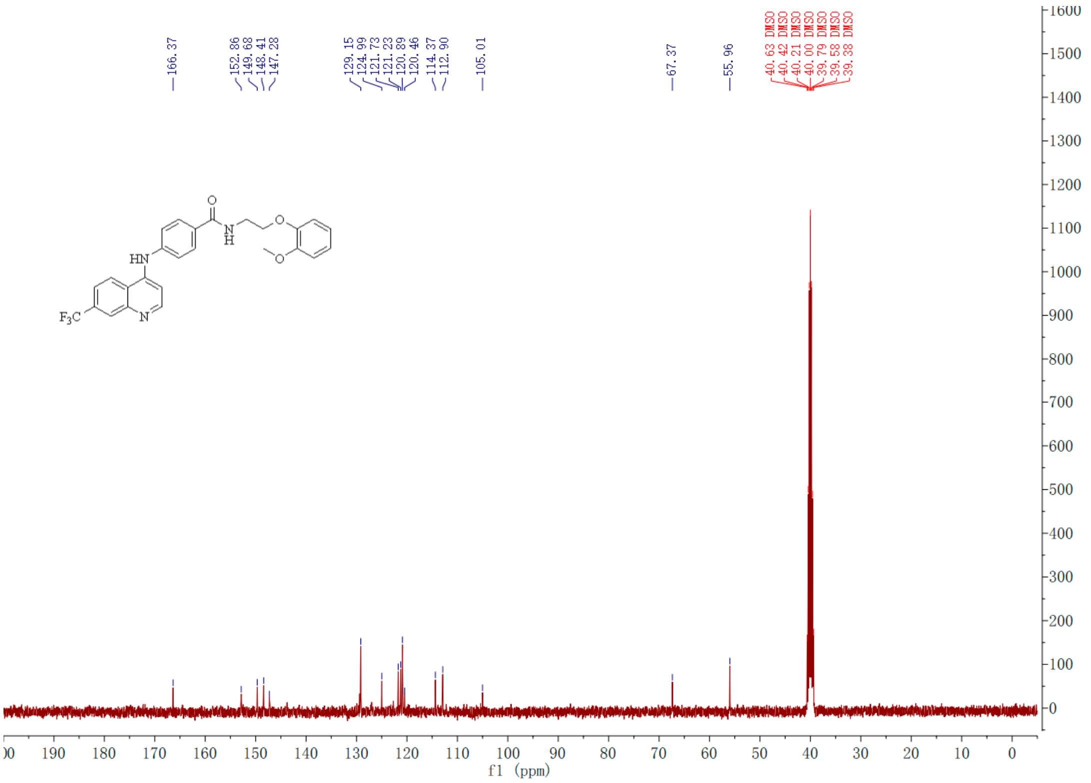

Figure S57. ^{13}C NMR spectra of the target compound **G07**.

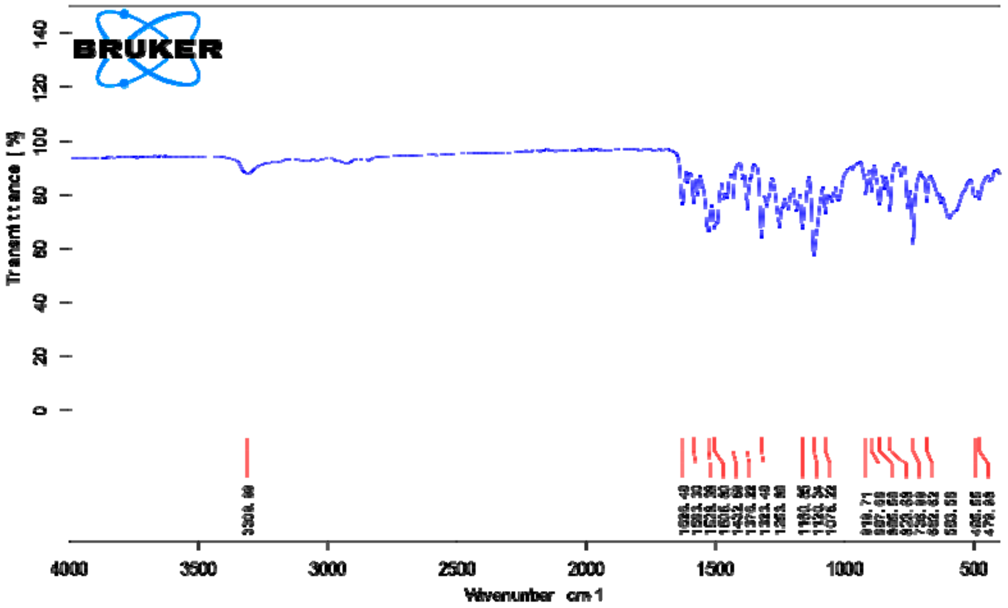

Figure S58. IR spectra of the target compound **G07**.

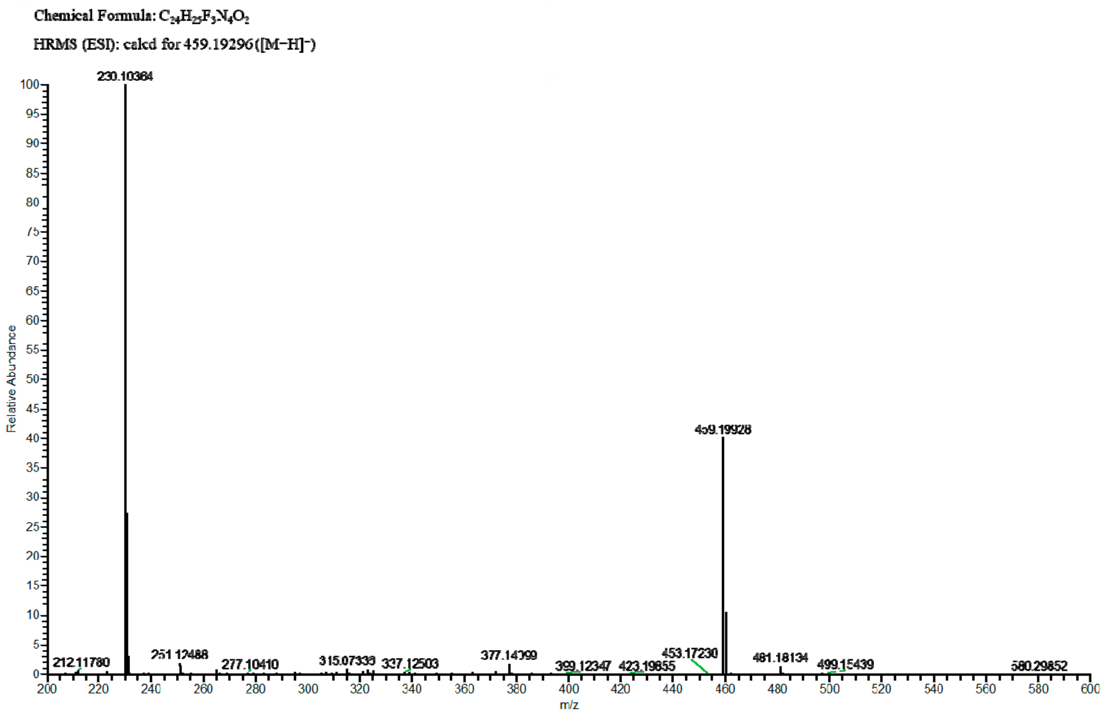

Figure S59. HRMS (ESI) of the target compound **G08**.

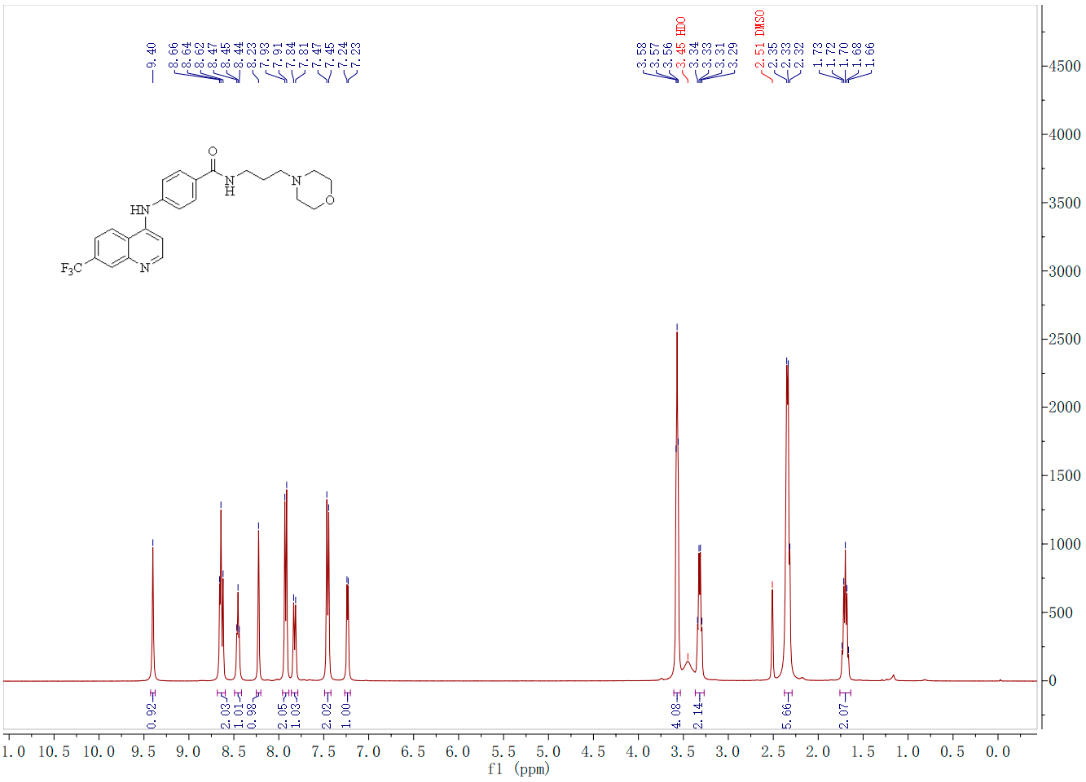

Figure S60. ^{1}H NMR spectra of the target compound **G08**.

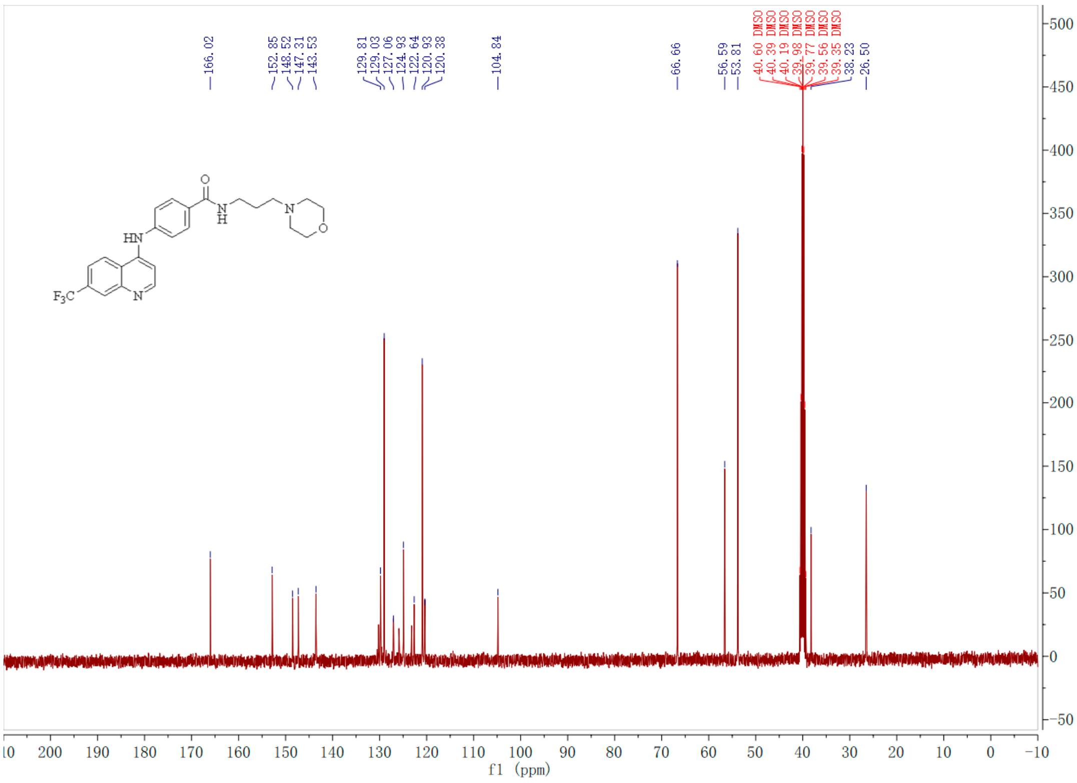

Figure S61. ^{13}C NMR spectra of the target compound **G08**.

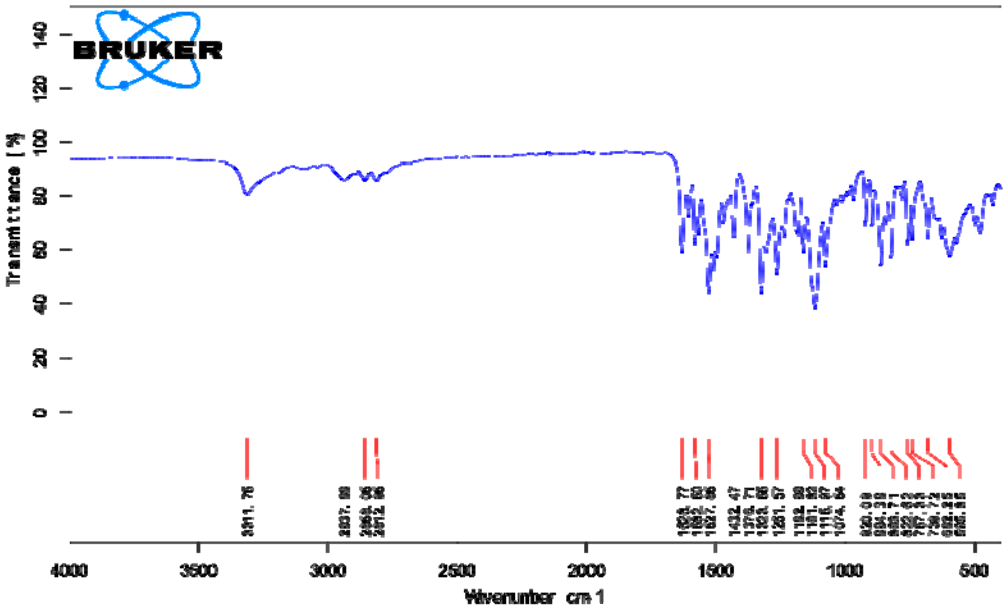

Figure S62. IR spectra of the target compound **G08**.

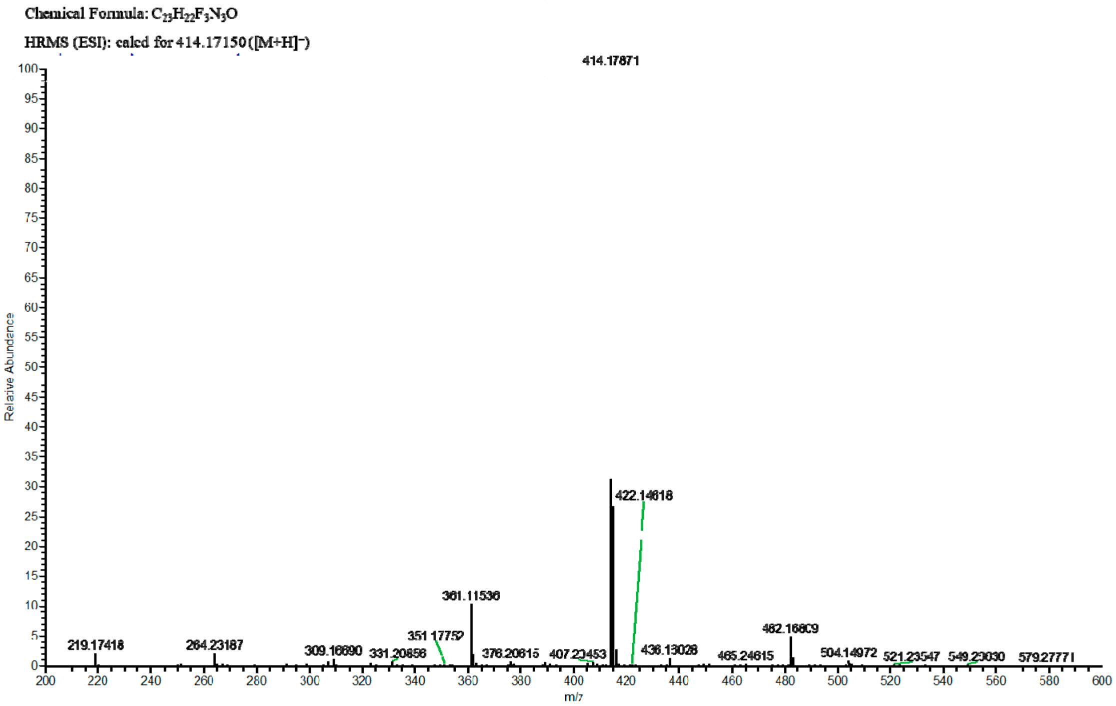

Figure S63. HRMS (ESI) of the target compound **G09**.

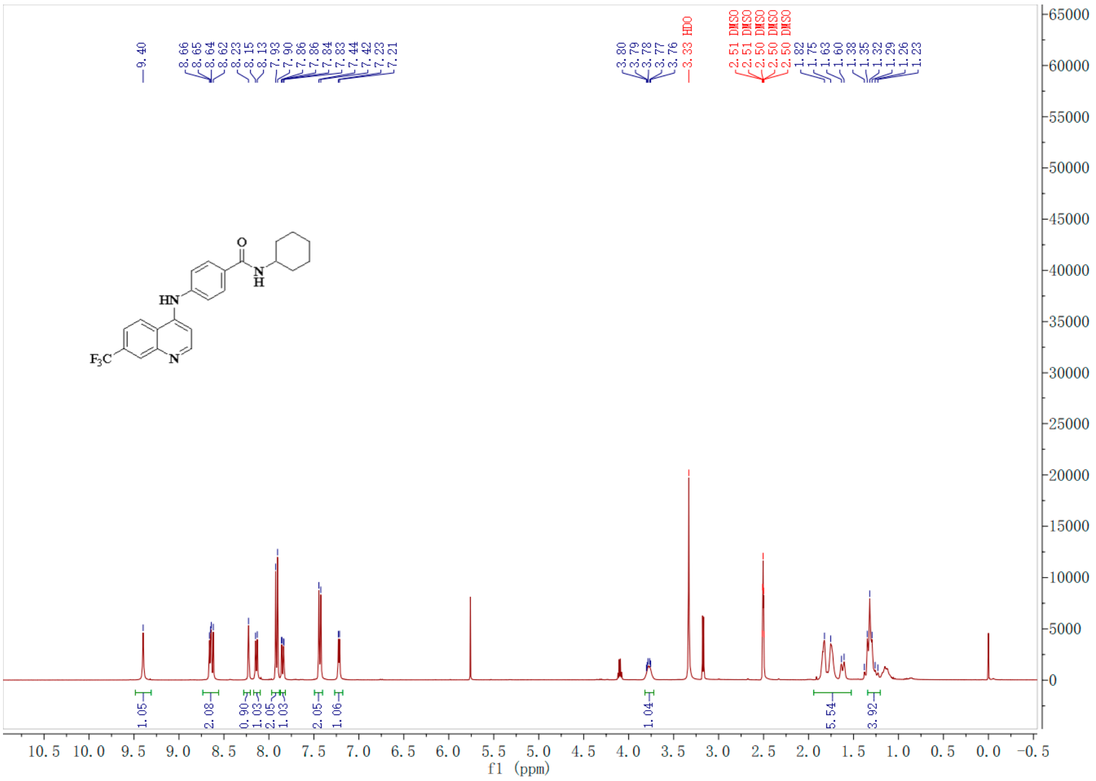

Figure S64. ^{1}H NMR spectra of the target compound **G09**.

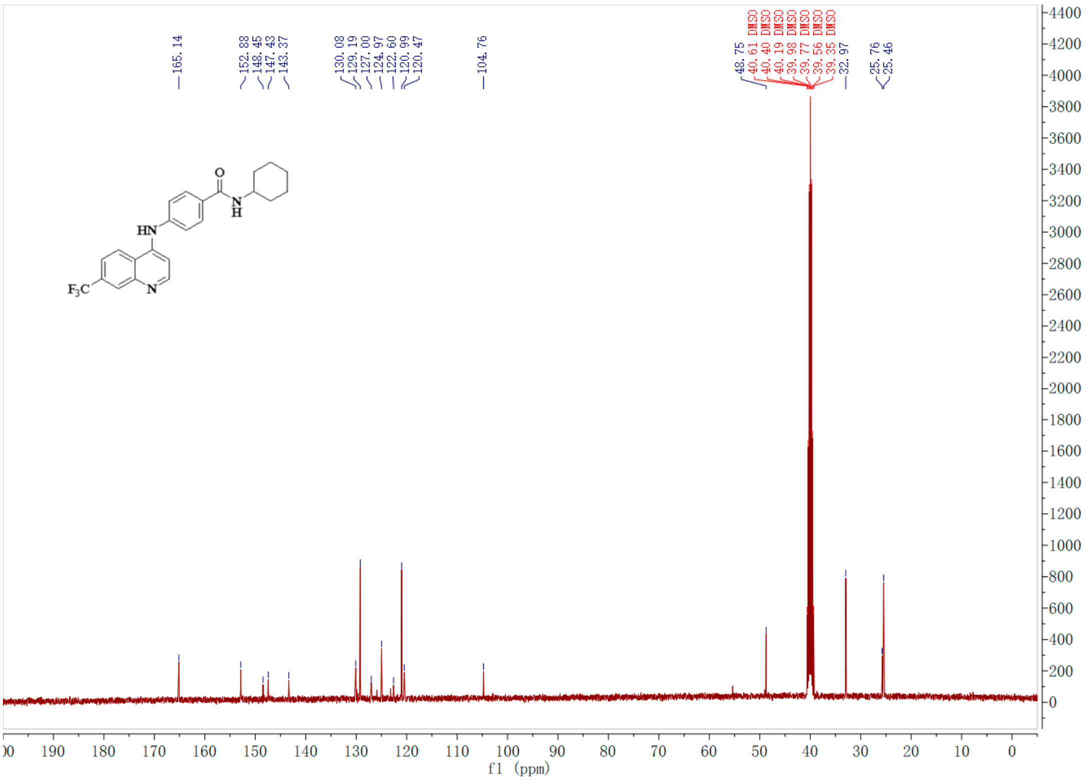

Figure S65. ^{13}C NMR spectra of the target compound **G09**.

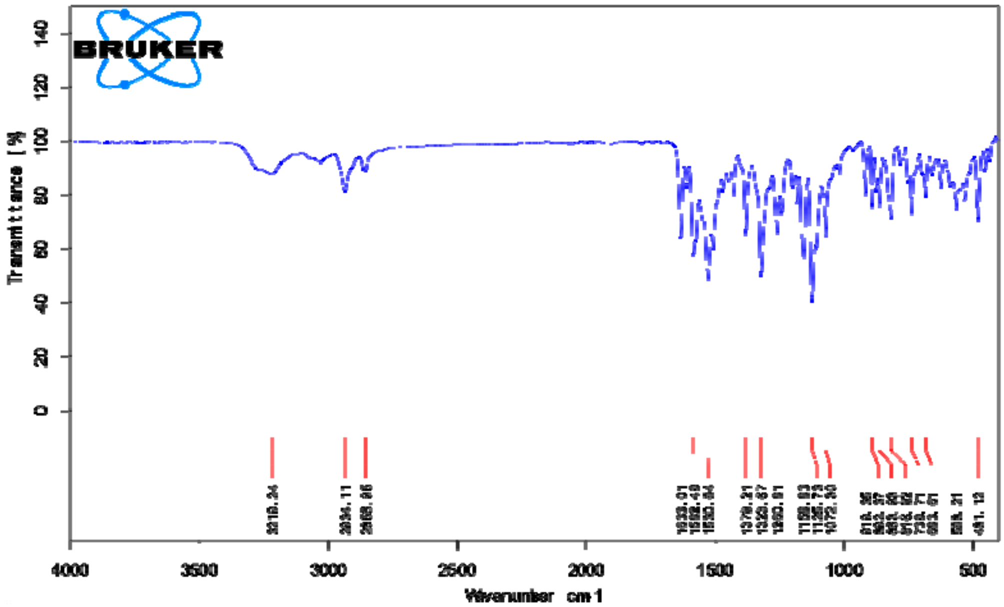

Figure S66. IR spectra of the target compound **G09**.

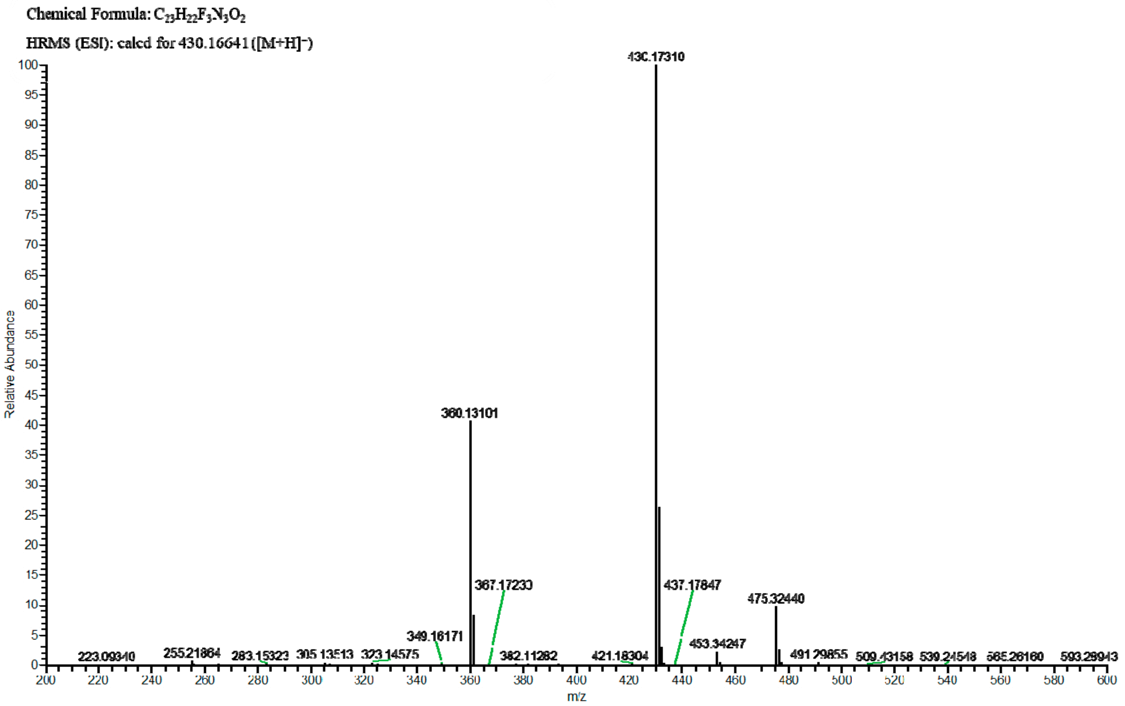

Figure S67. HRMS (ESI) of the target compound **G10**.

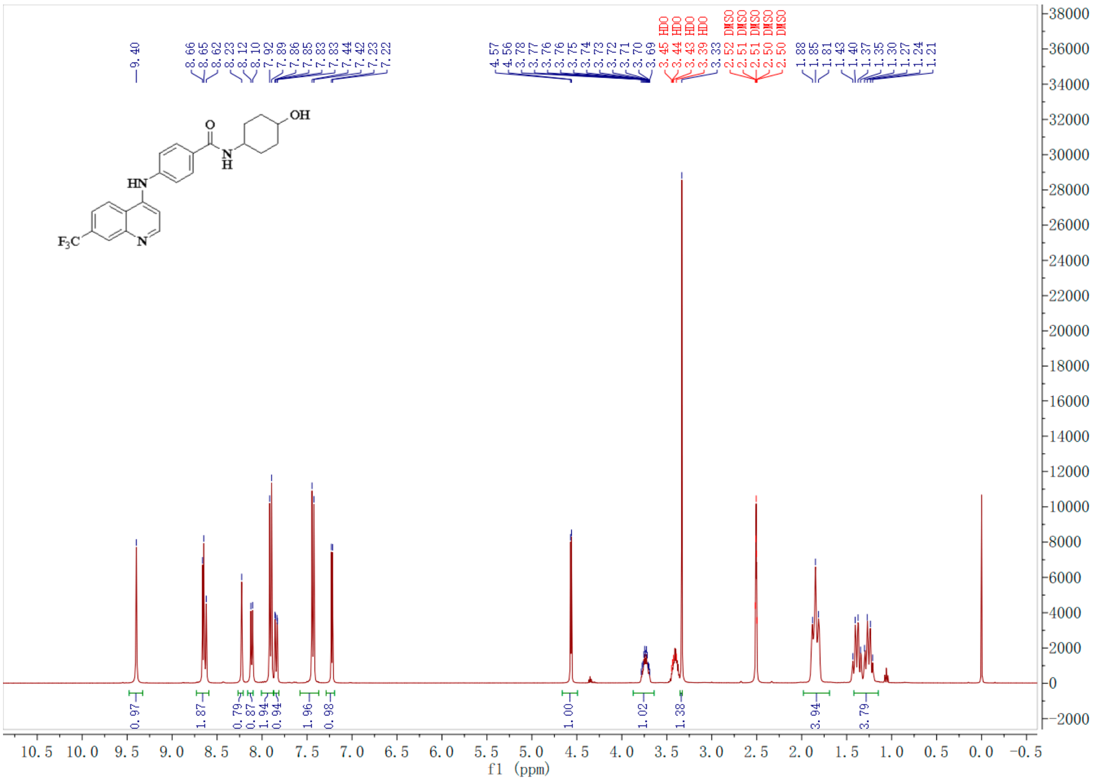

Figure S68. ^{1}H NMR spectra of the target compound **G10**.

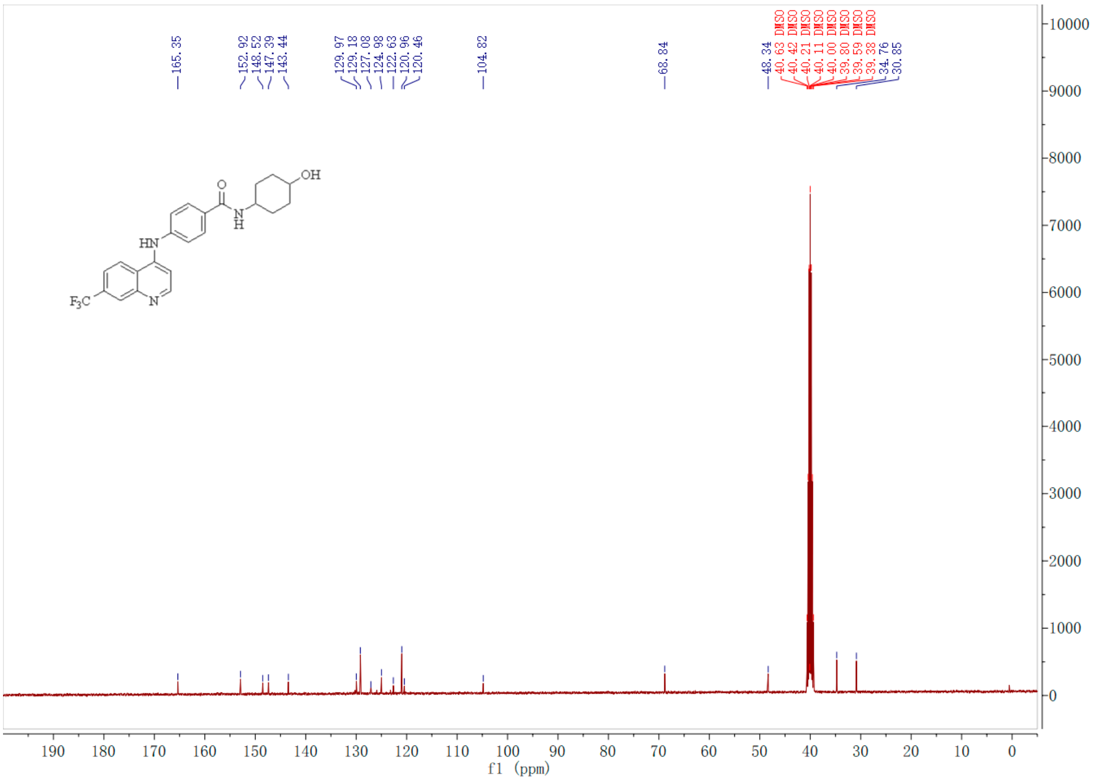

Figure S69. ^{13}C NMR spectra of the target compound **G10**.

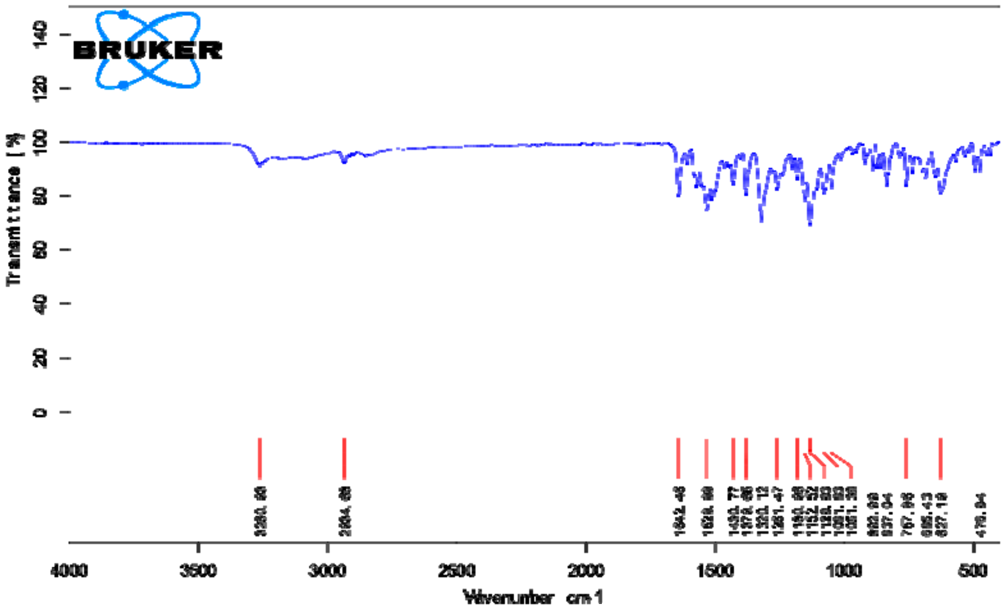

Figure S70. IR spectra of the target compound **G10**.

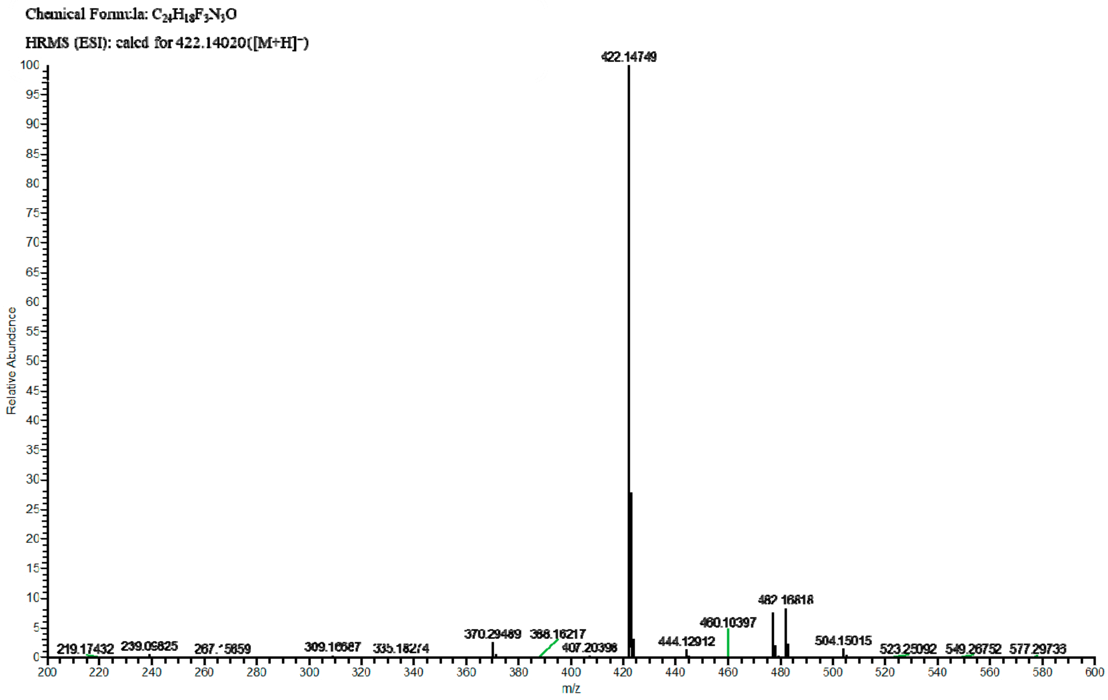

Figure S71. HRMS (ESI) of the target compound **G11**.

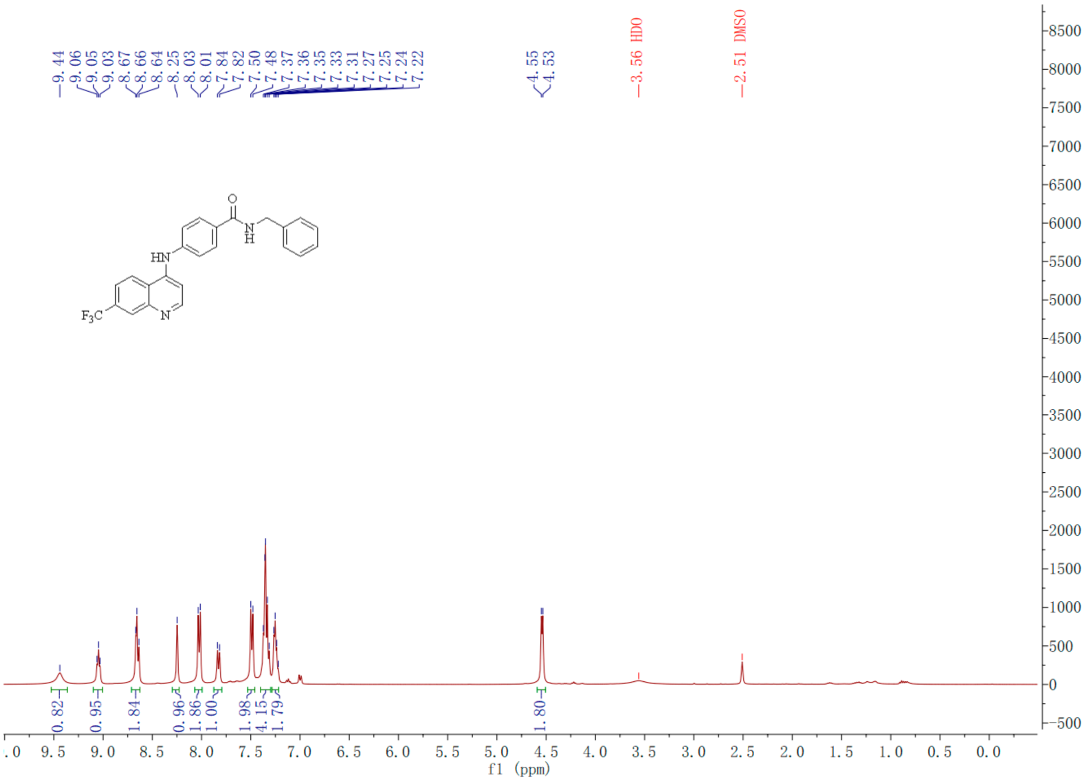

Figure S72. ^{1}H NMR spectra of the target compound **G11**.

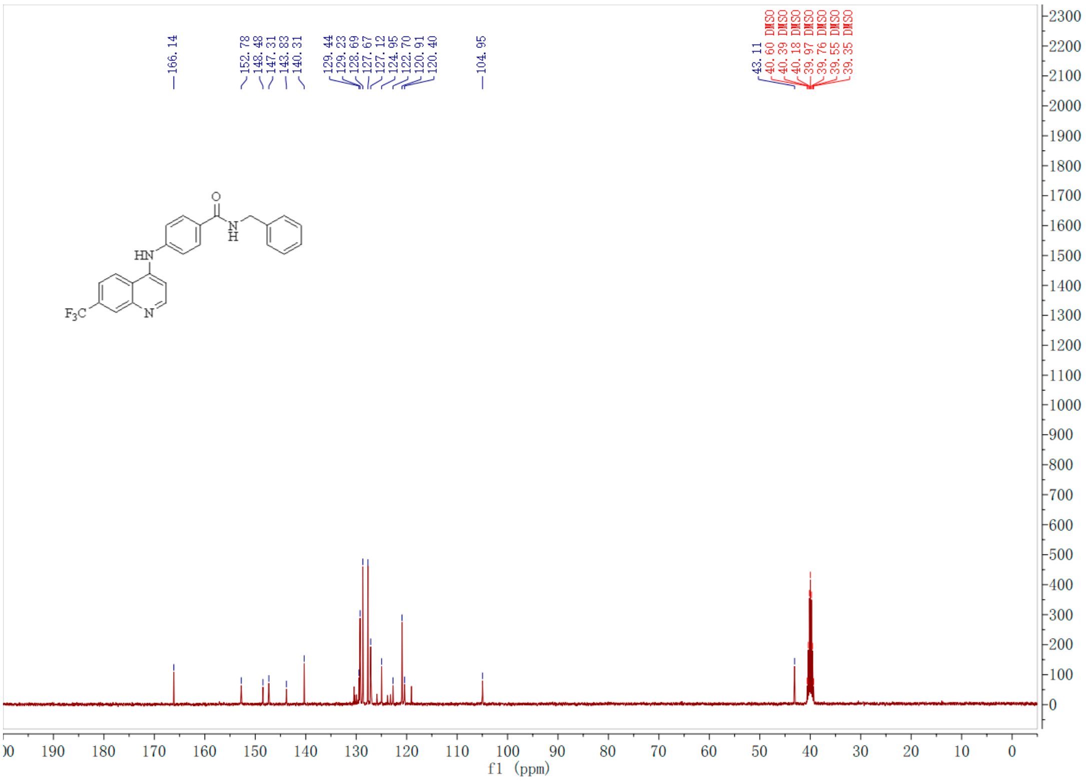

Figure S73. ^{13}C NMR spectra of the target compound **G11**.

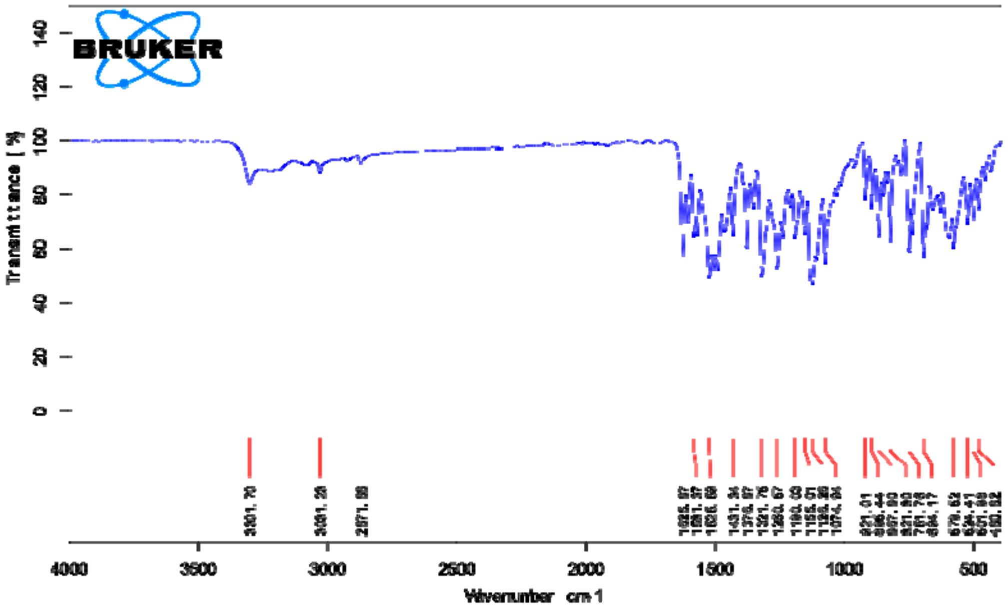

Figure S74. IR spectra of the target compound **G11**.

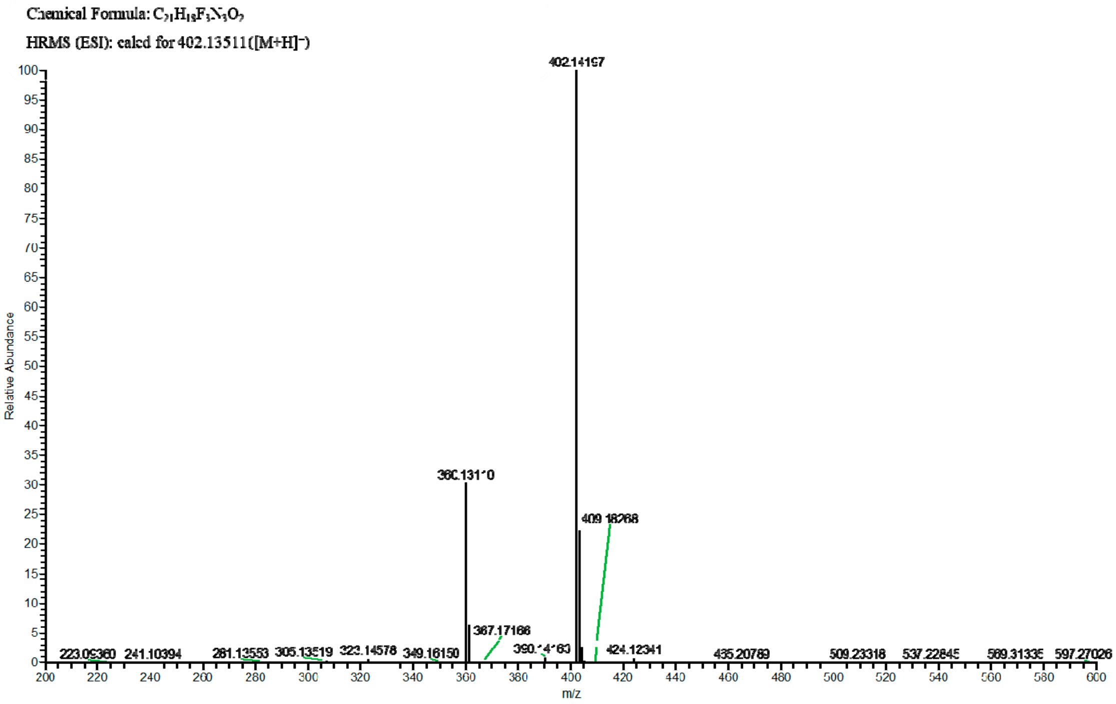

Figure S75. HRMS (ESI) of the target compound **G12**.

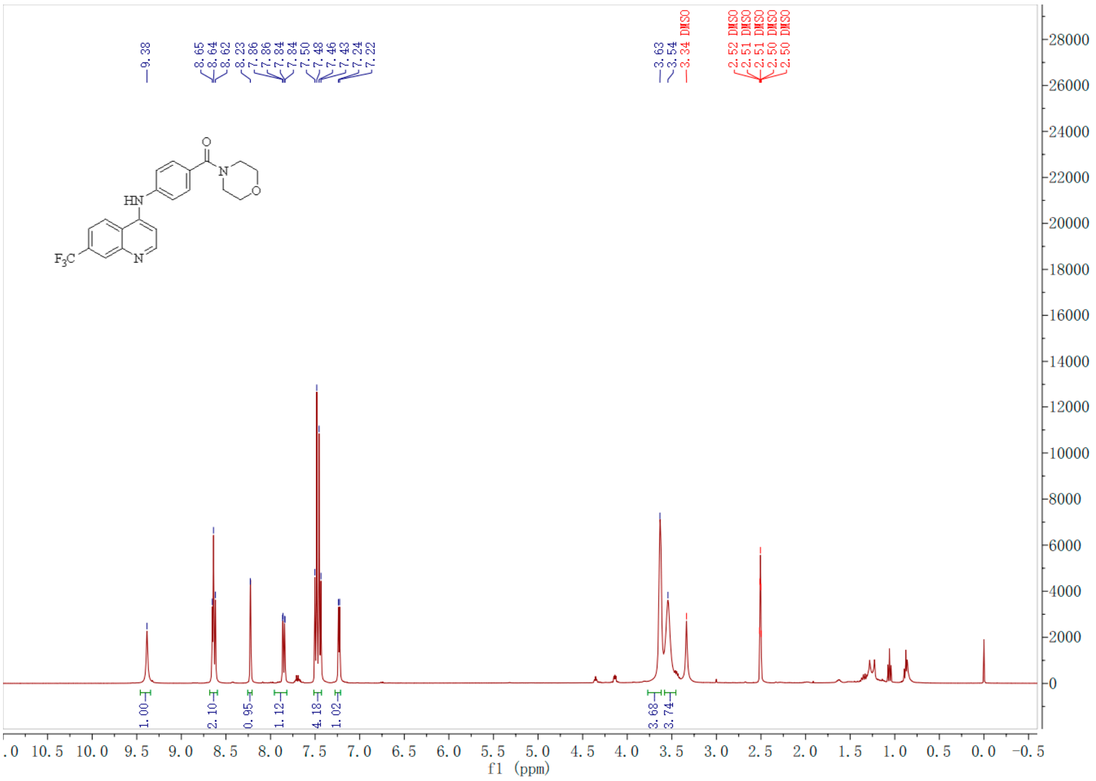

Figure S76. ^1H NMR spectra of the target compound **G12**.

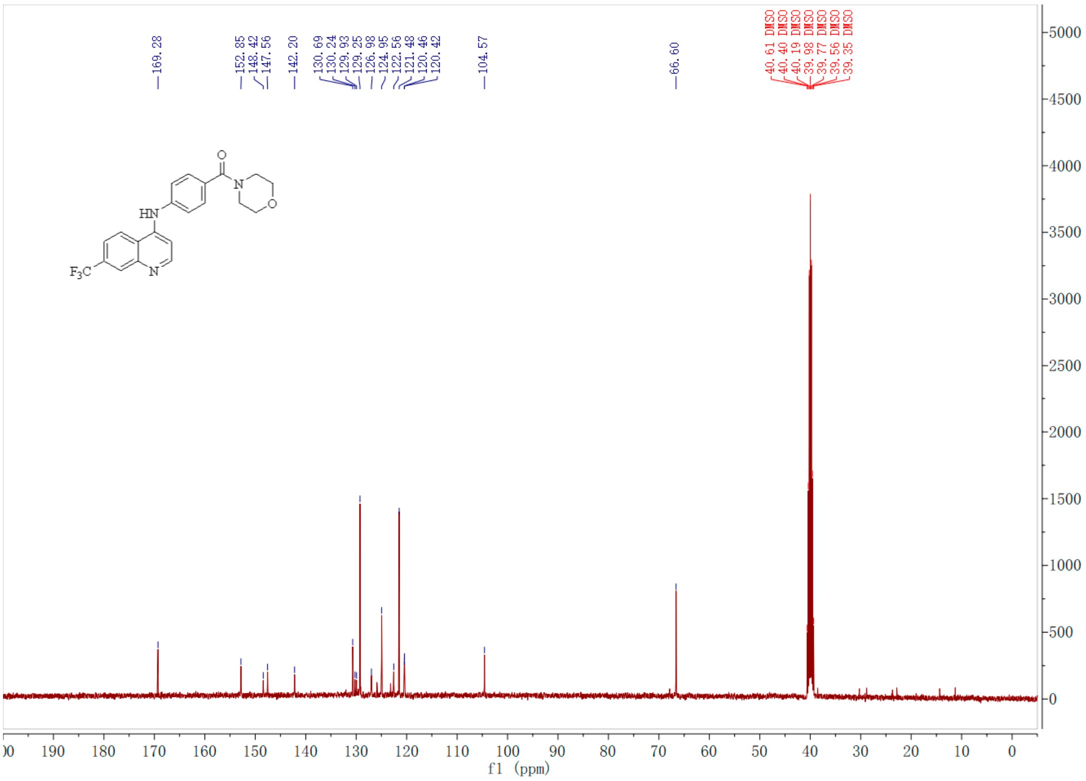

Figure S77. ^{13}C NMR spectra of the target compound **G12**.

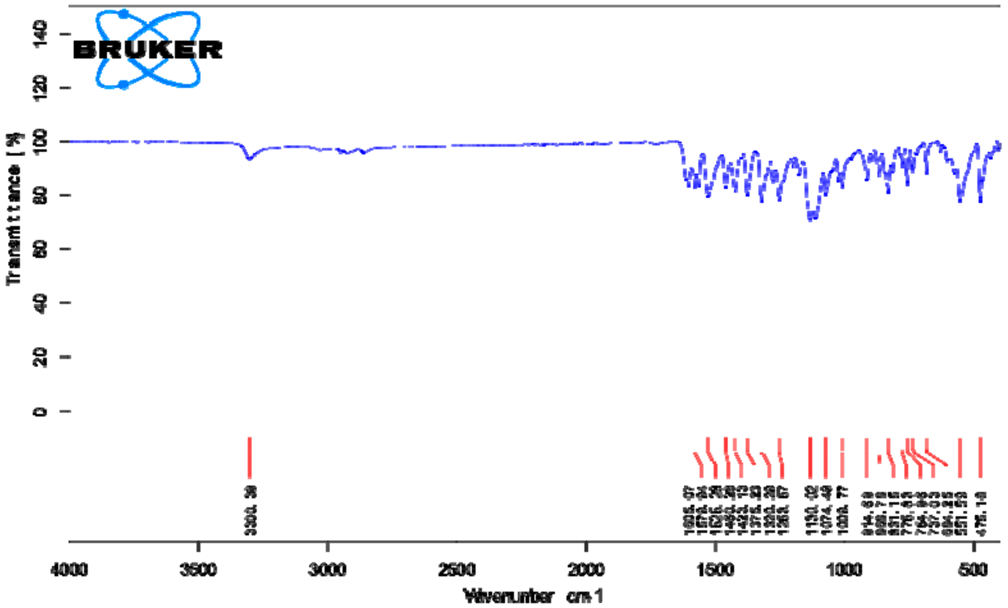

Figure S78. IR spectra of the target compound **G12**.

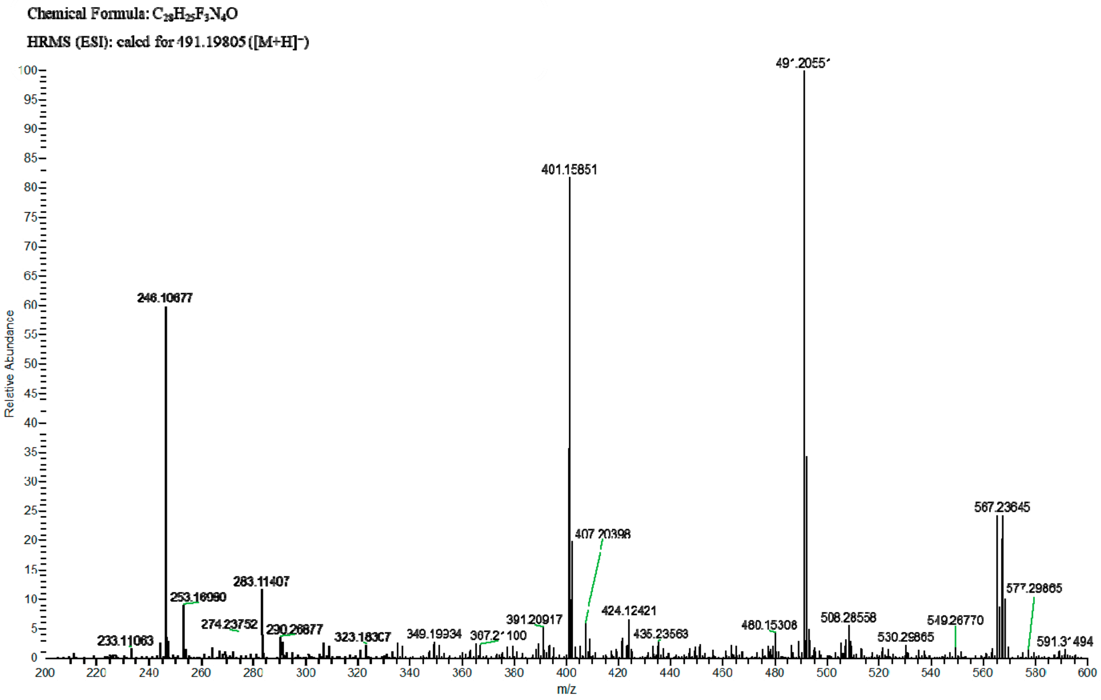

Figure S79. HRMS (ESI) of the target compound **G13**.

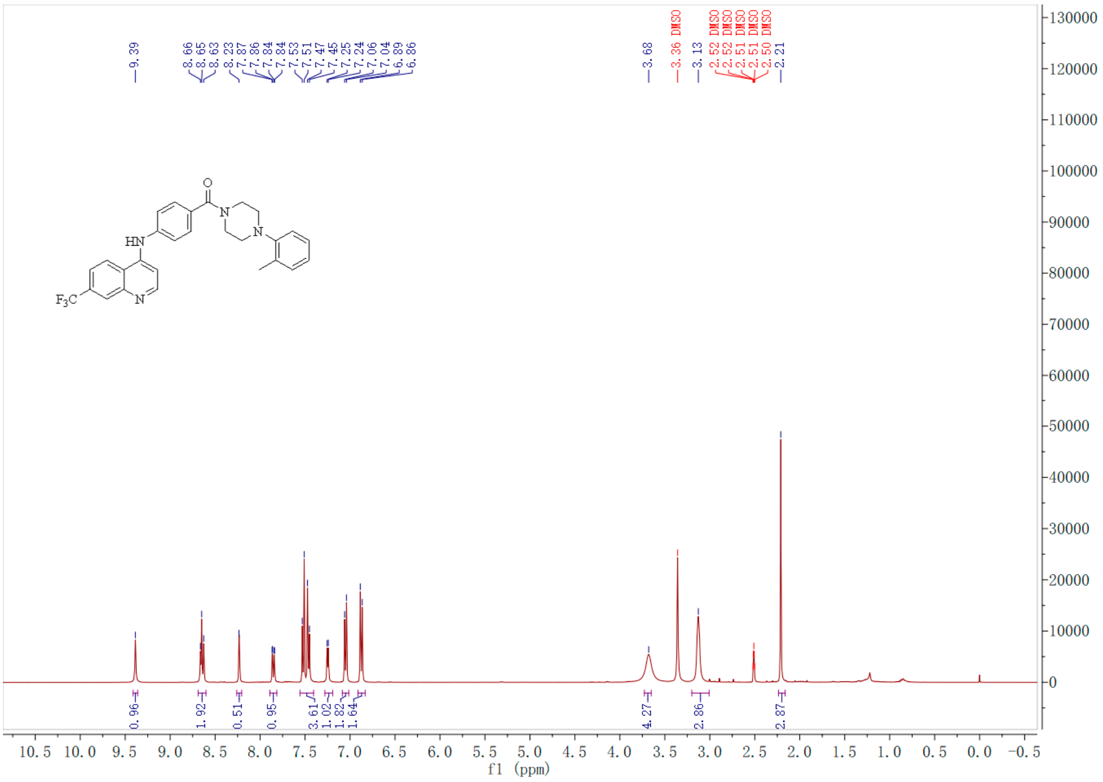

Figure S80. ^{1}H NMR spectra of the target compound **G13**.

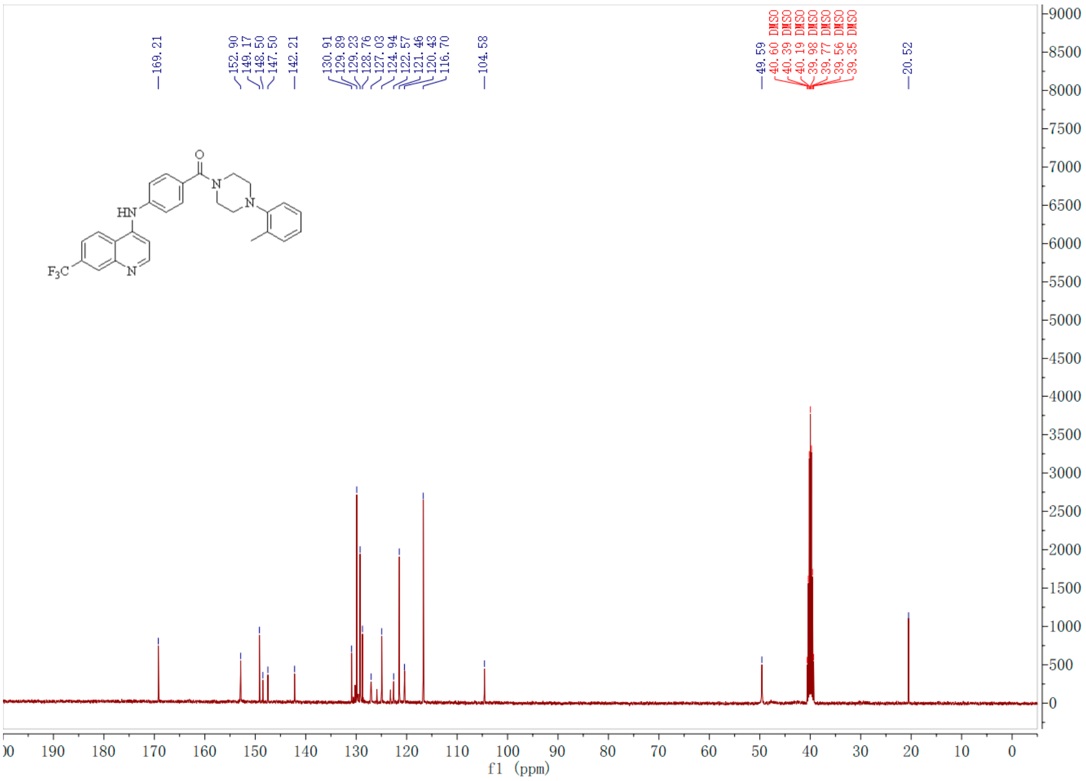

Figure S81. ^{13}C NMR spectra of the target compound **G13**.

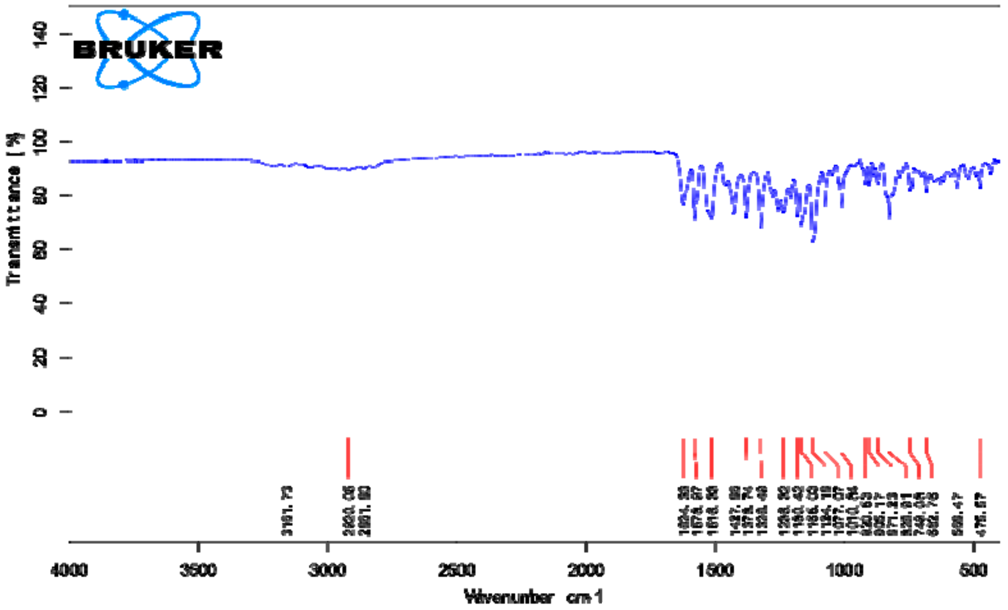

Figure S82. IR spectra of the target compound **G13**.

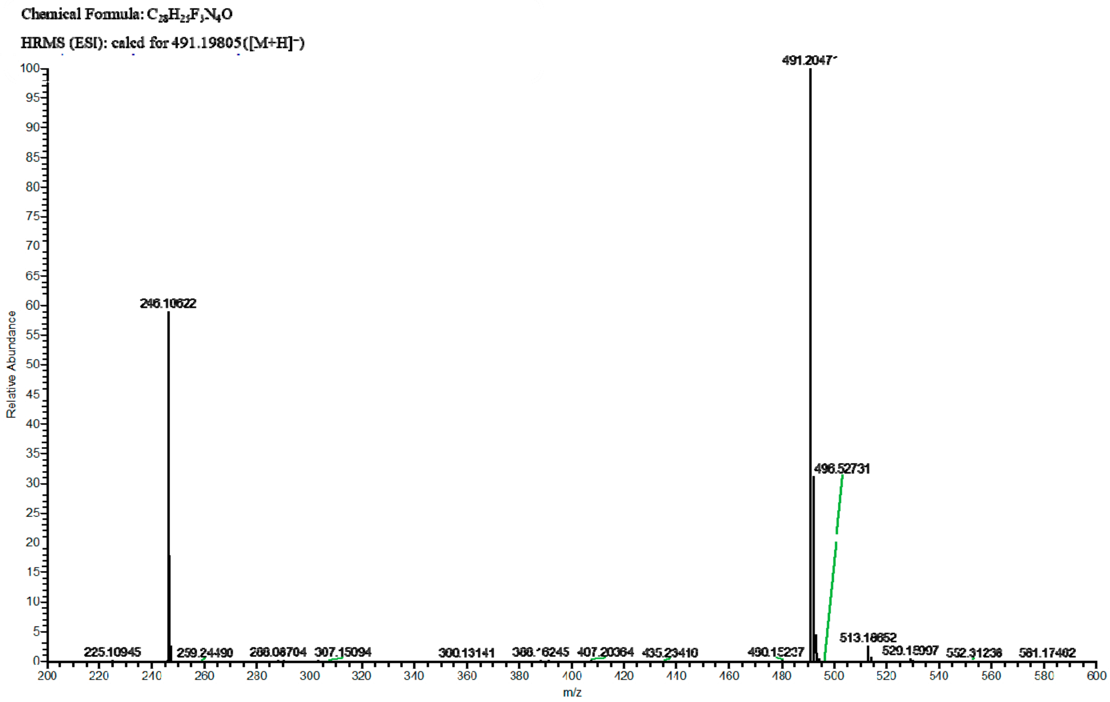

Figure S83. HRMS (ESI) of the target compound **G14**.

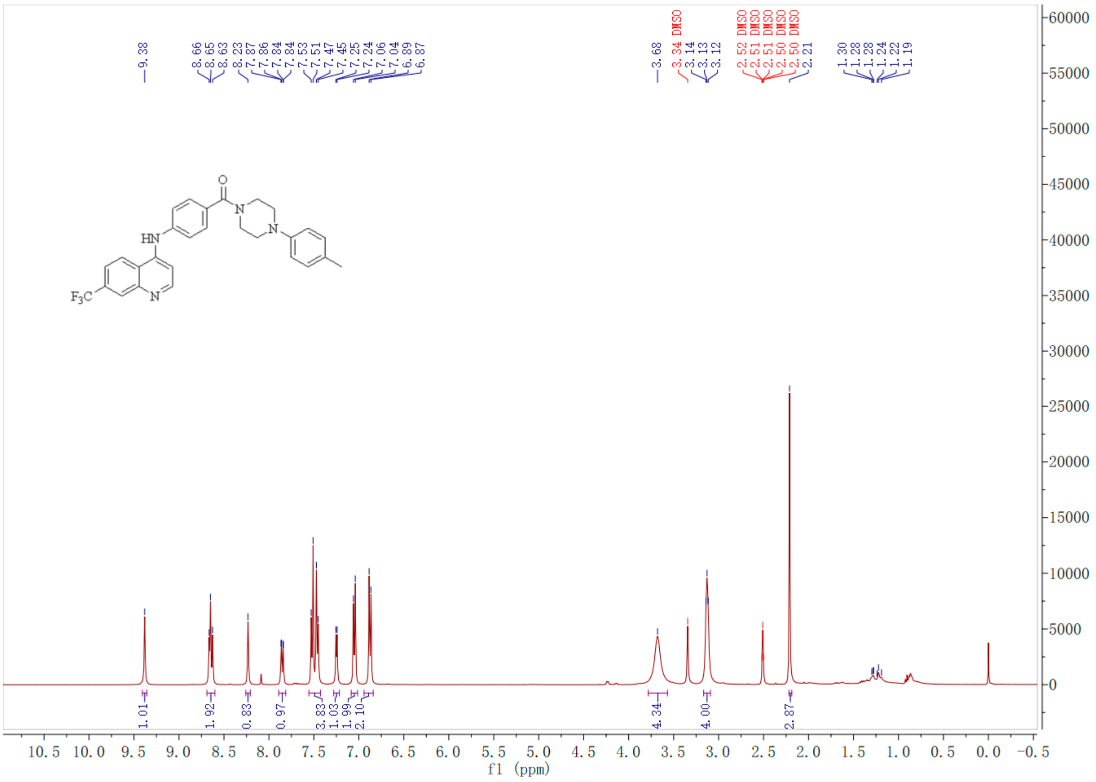

Figure S84. ^{1}H NMR spectra of the target compound **G14**.

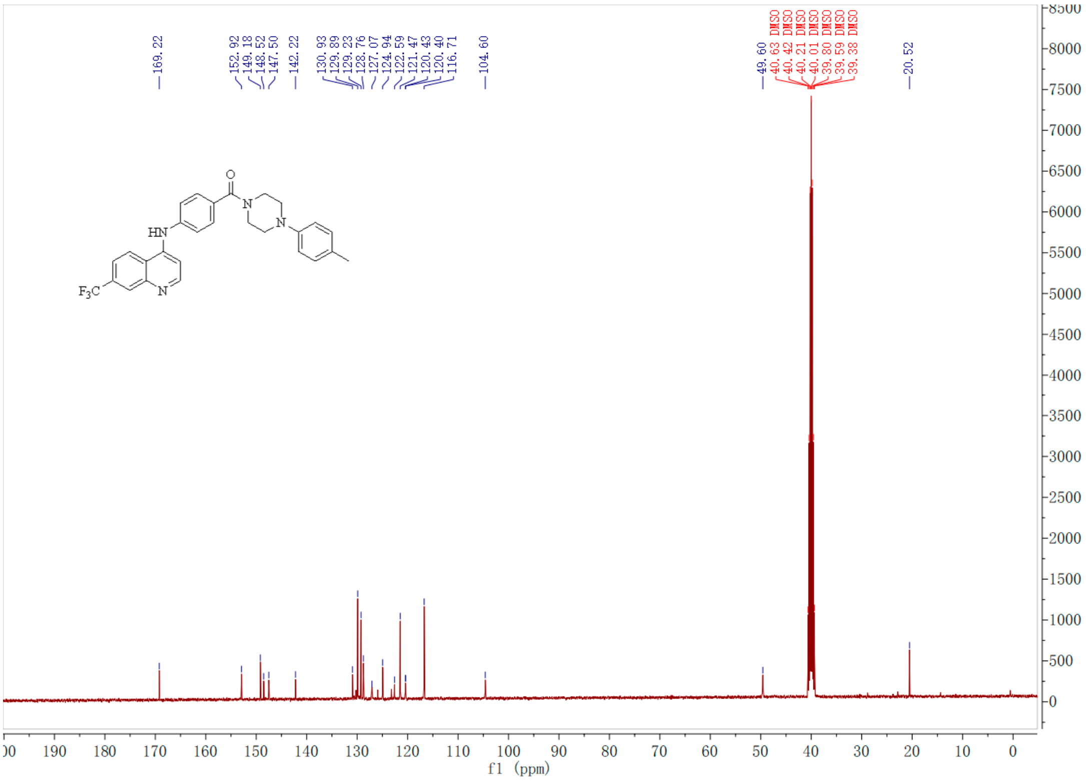

Figure S85. ^{13}C NMR spectra of the target compound **G14**.

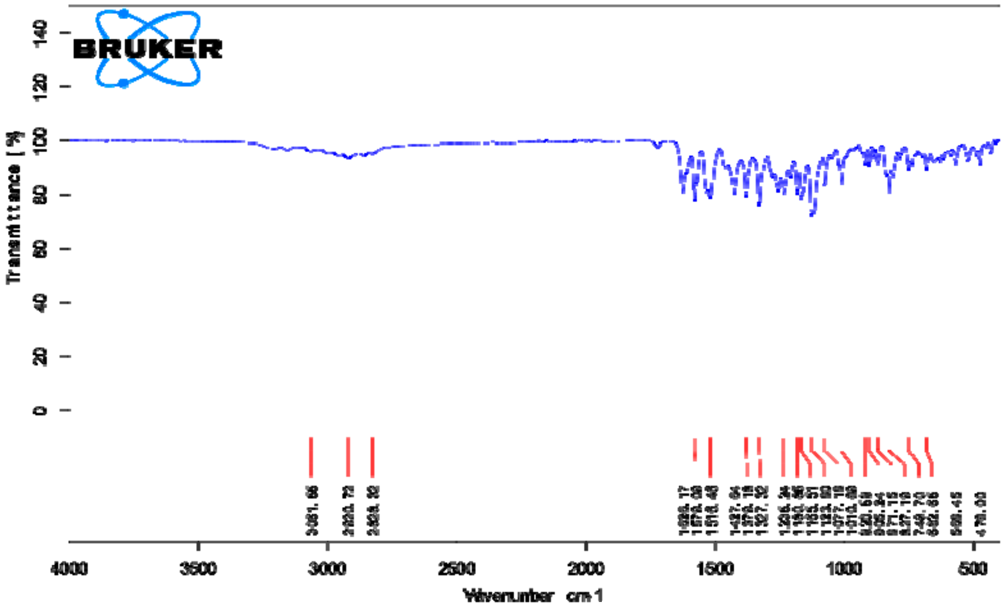

Figure S86. IR spectra of the target compound **G14**.

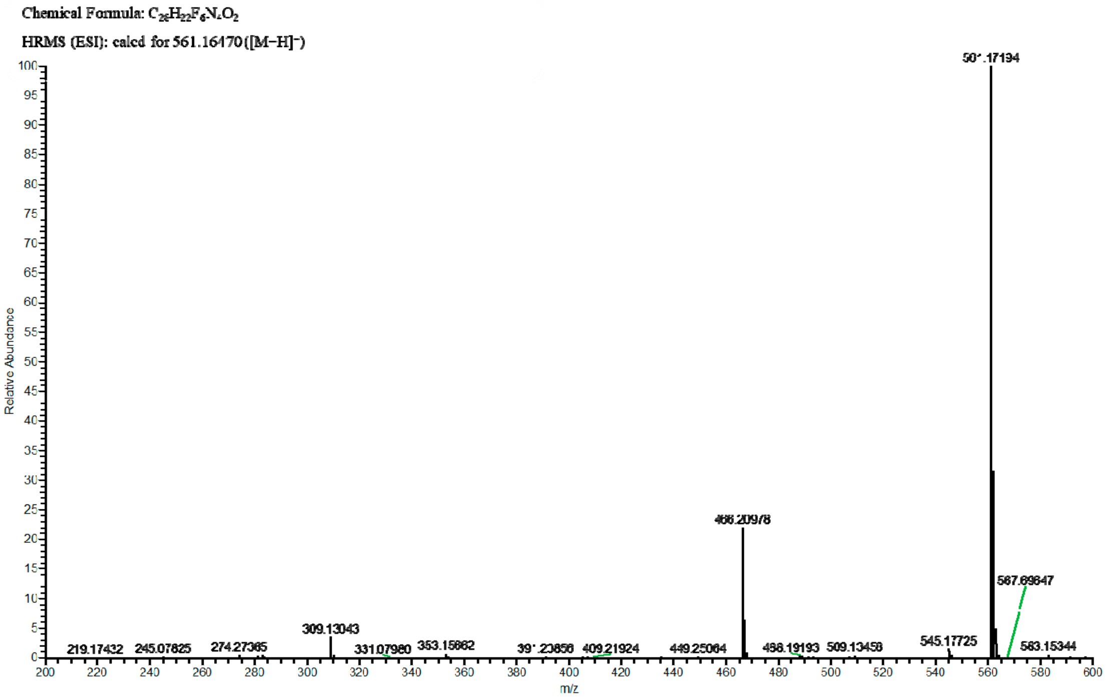

Figure S87. HRMS (ESI) of the target compound **G15**.

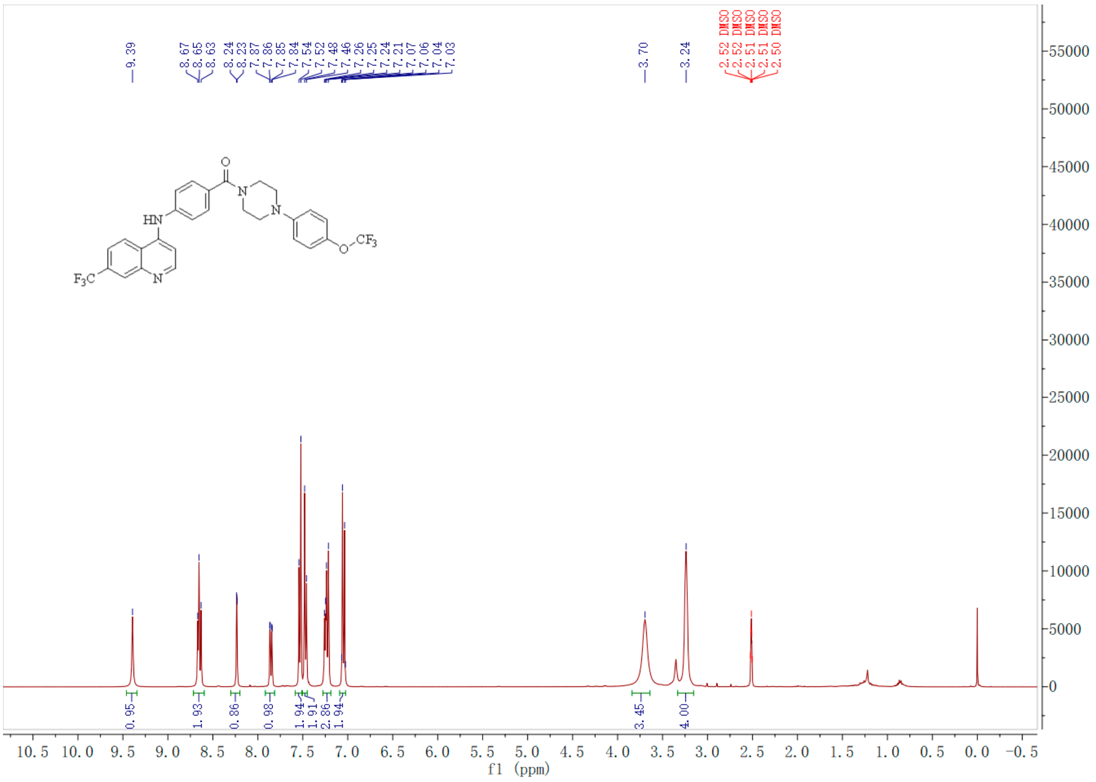

Figure S88. ^{1}H NMR spectra of the target compound **G15**.

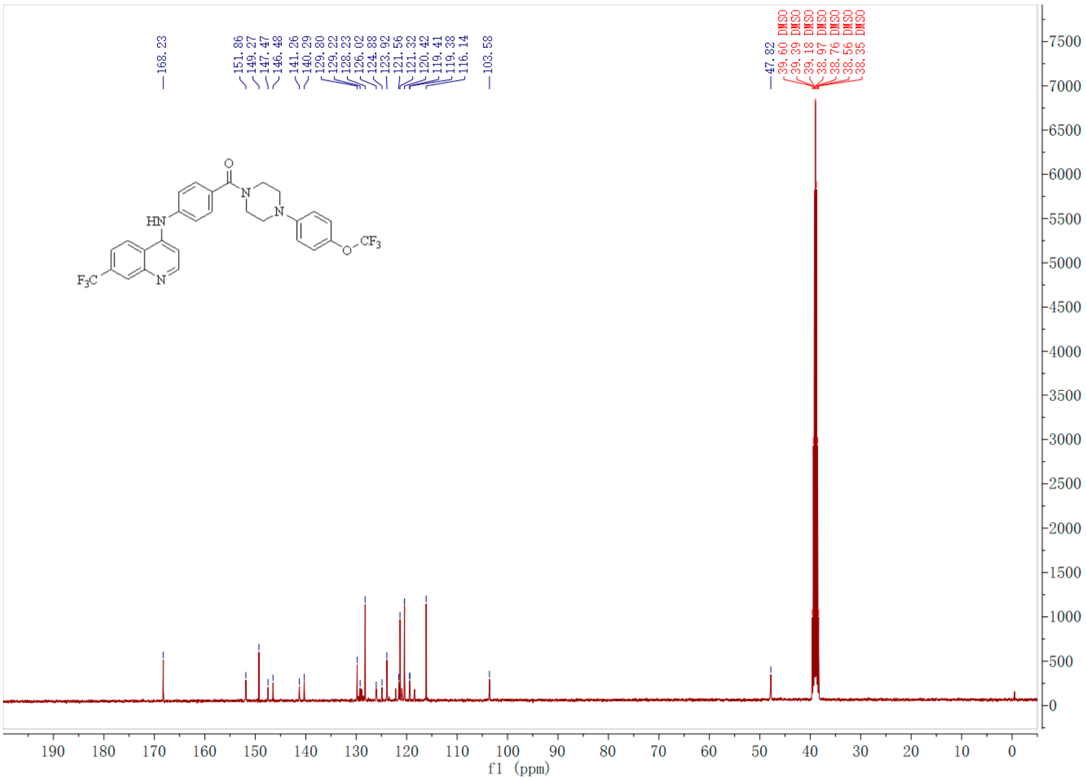

Figure S89. ^{13}C NMR spectra of the target compound **G15**.

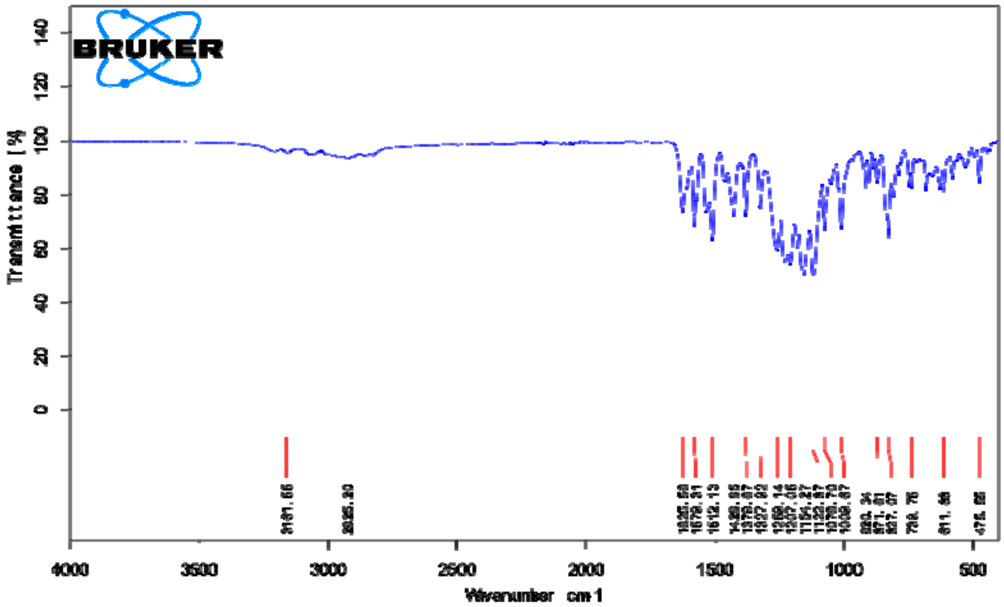

Figure S90. IR spectra of the target compound **G15**.

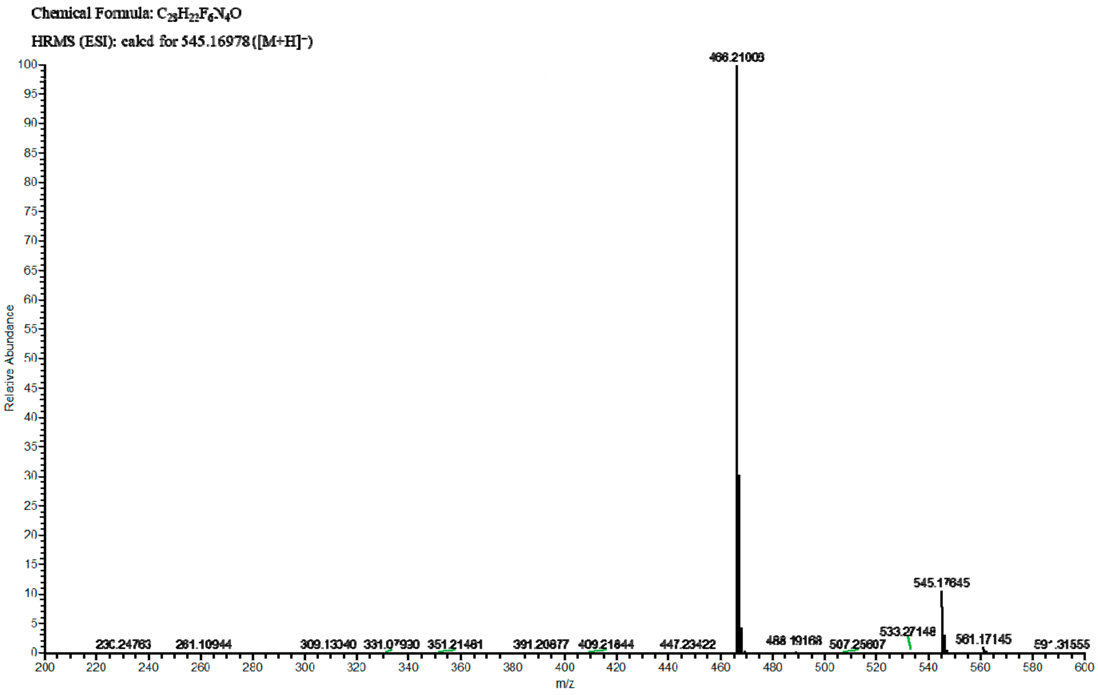

Figure S91. HRMS (ESI) of the target compound G16.

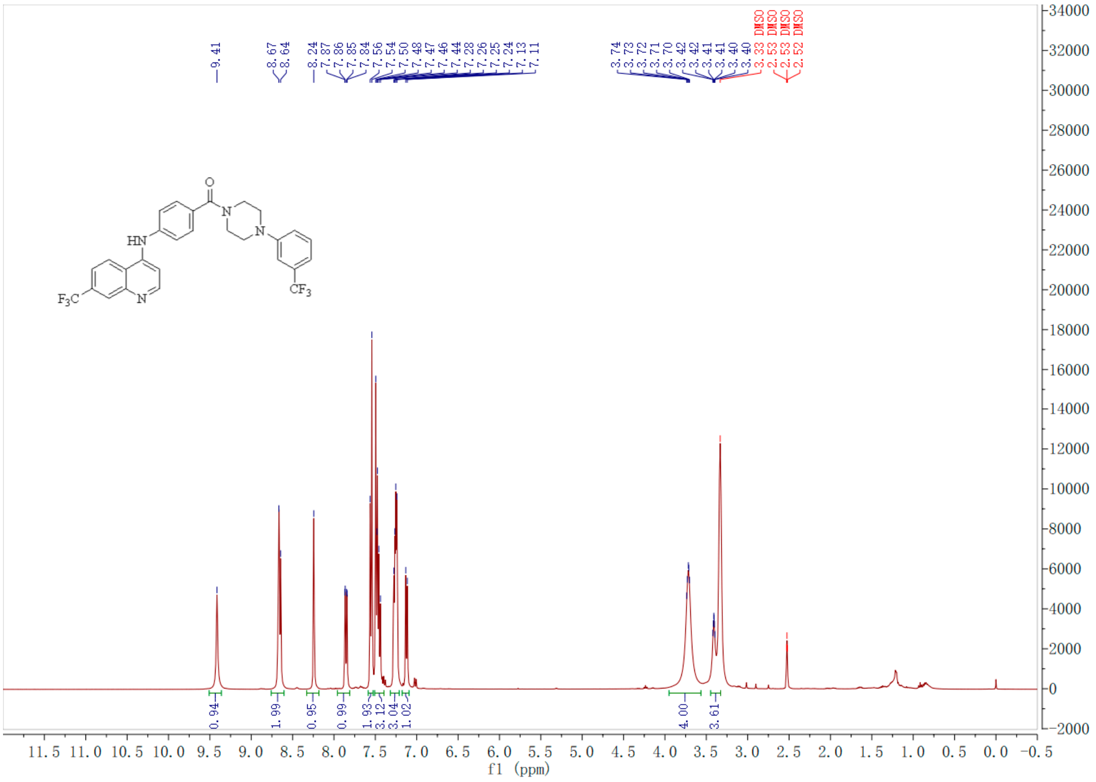

Figure S92. ^{1}H NMR spectra of the target compound G16.

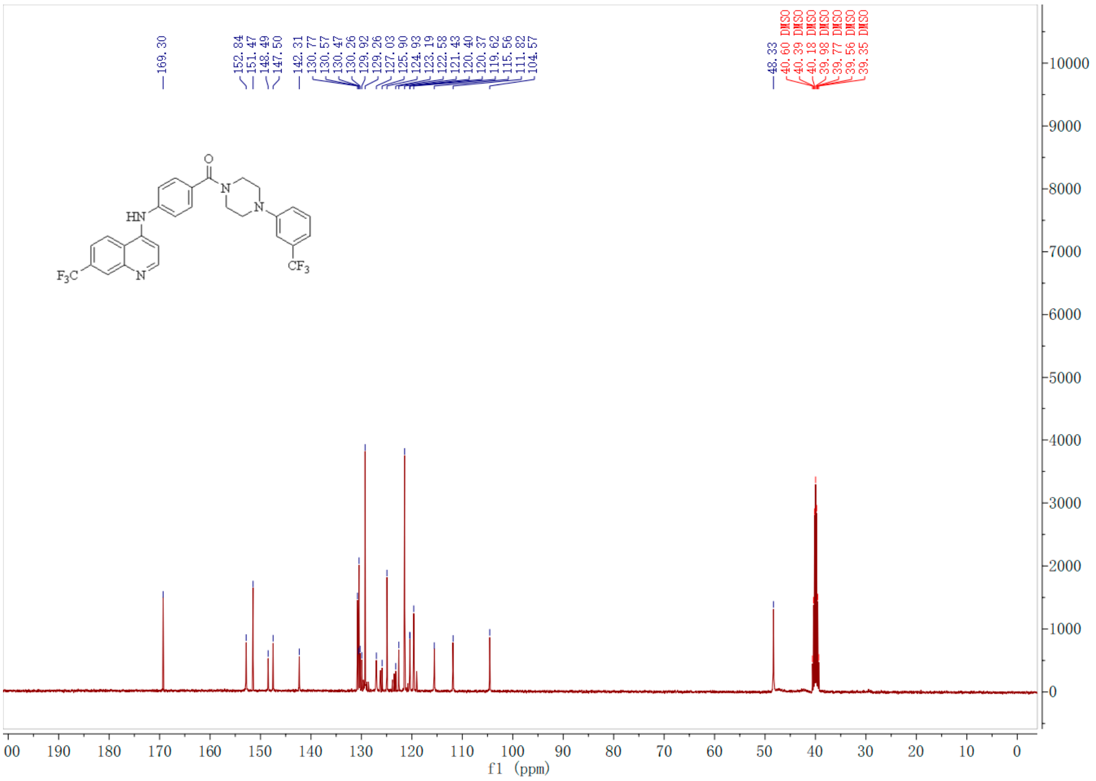

Figure S93. ^{13}C NMR spectra of the target compound **G16**.

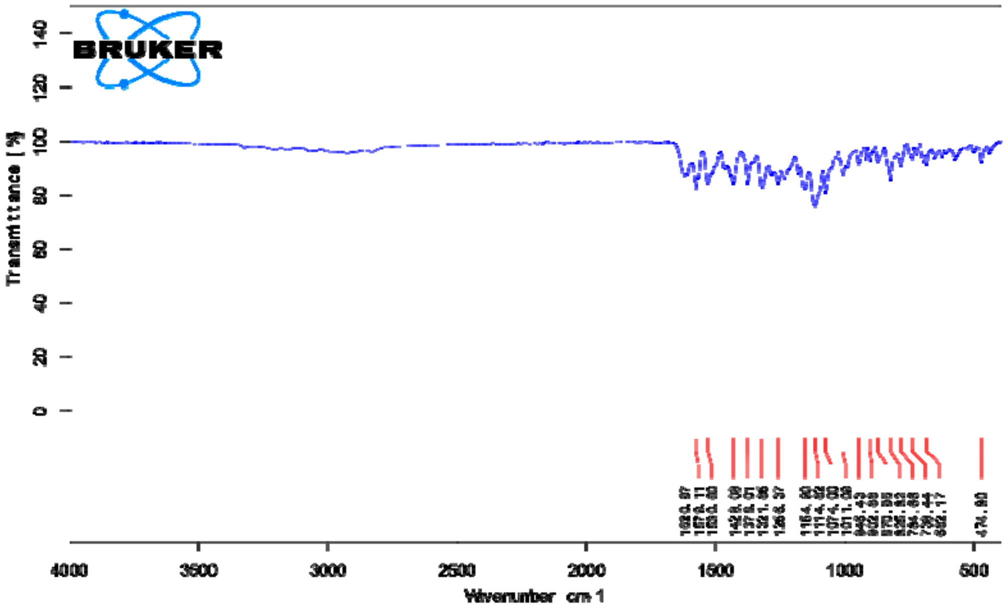

Figure S94. IR spectra of the target compound **G16**.

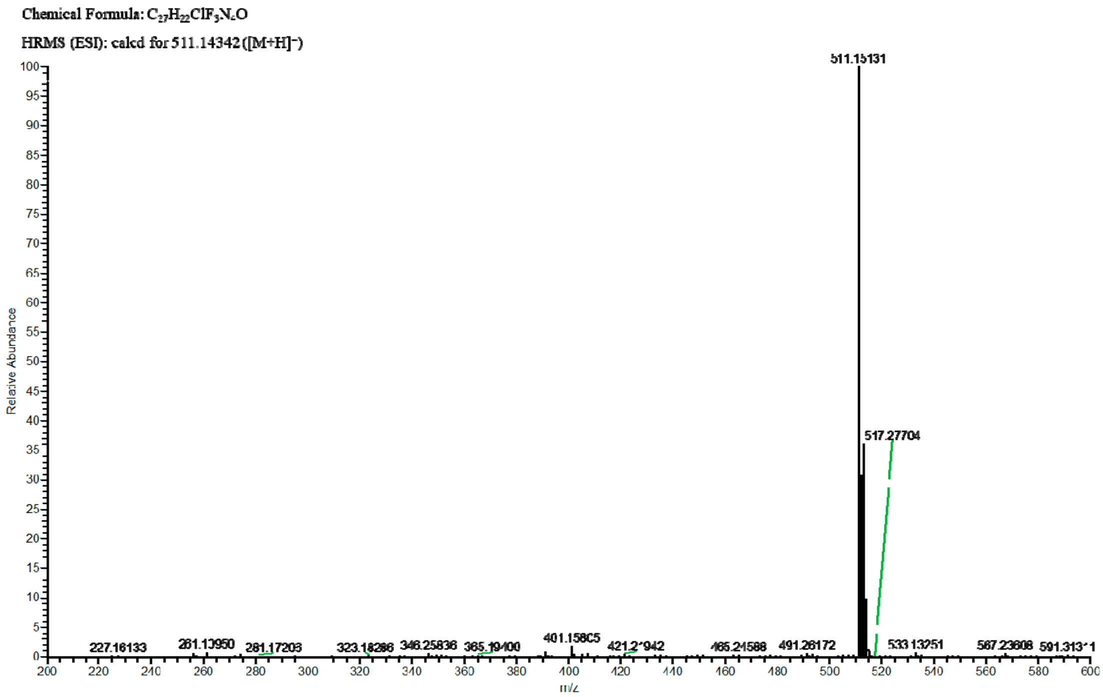

Figure S95. HRMS (ESI) of the target compound **G17**.

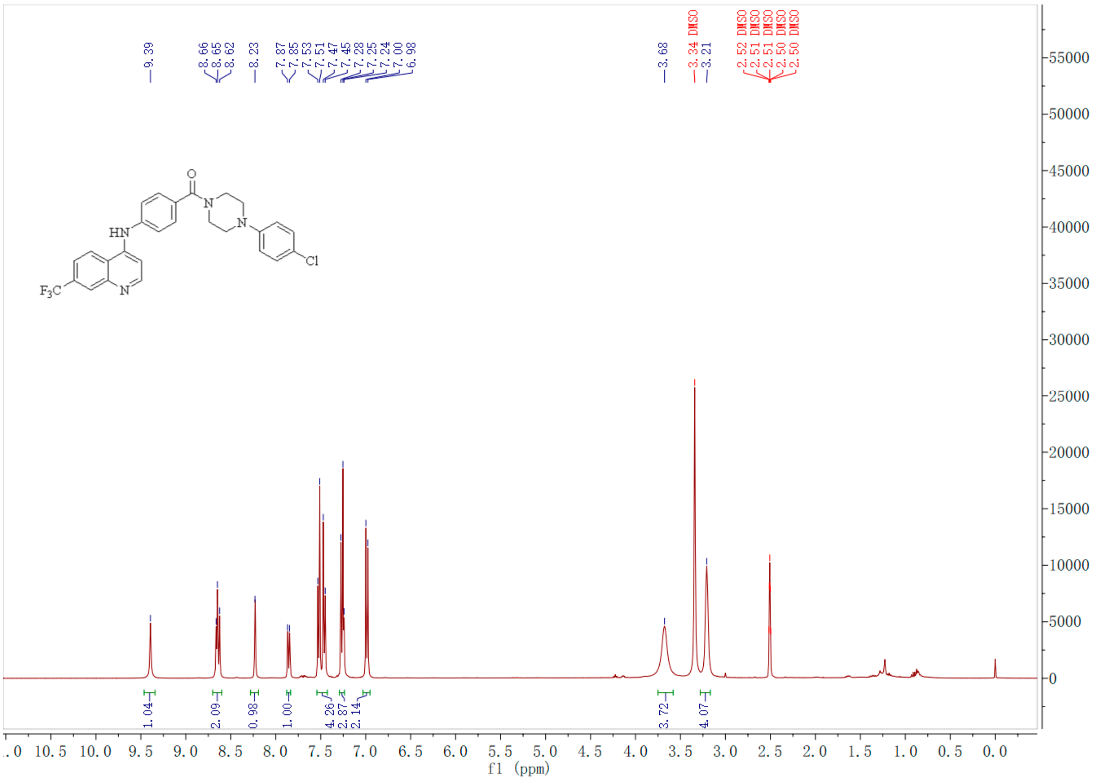

Figure S96. ^{1}H NMR spectra of the target compound **G17**.

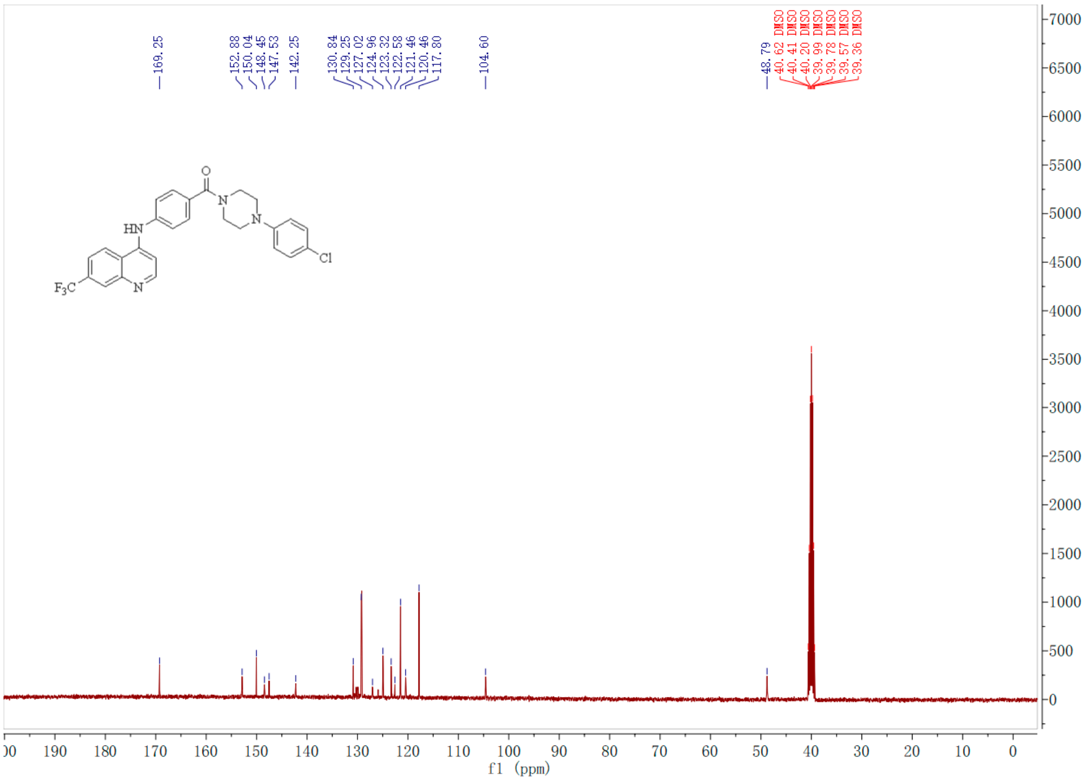

Figure S97. ^{13}C NMR spectra of the target compound **G17**.

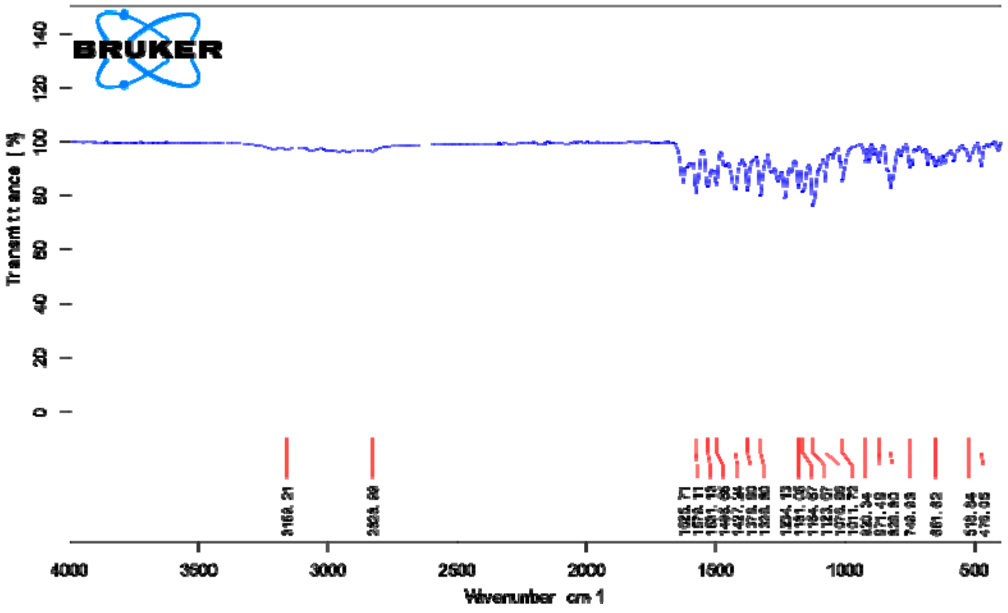

Figure S98. IR spectra of the target compound **G17**.

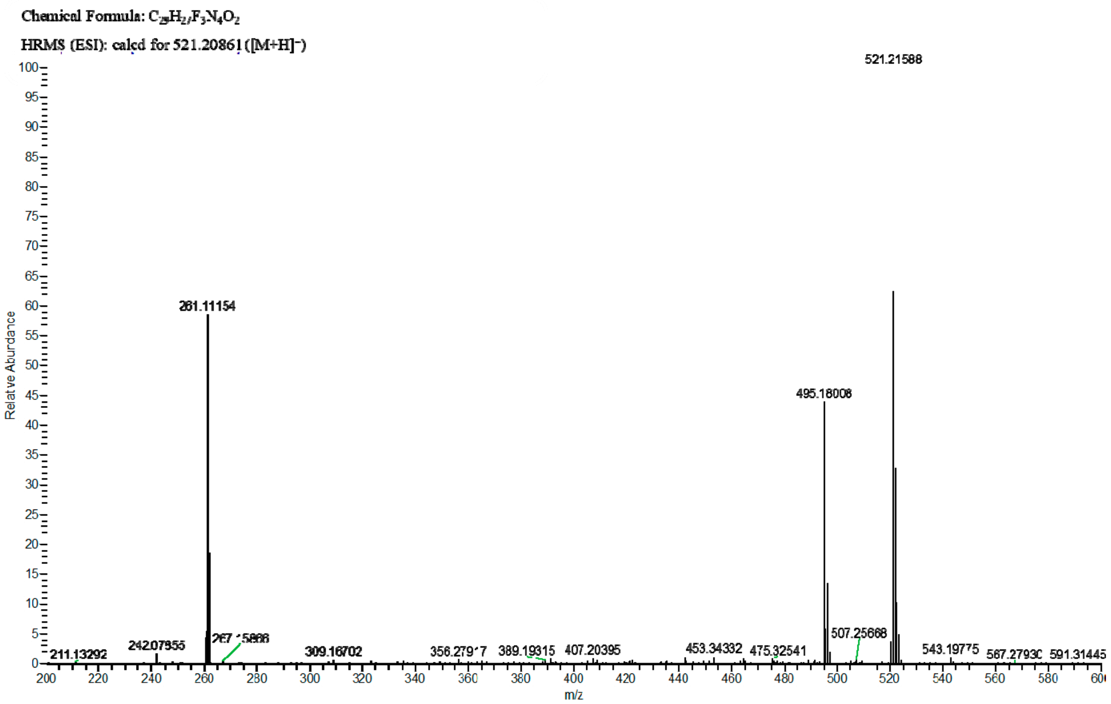

Figure S99. HRMS (ESI) of the target compound **G18**.

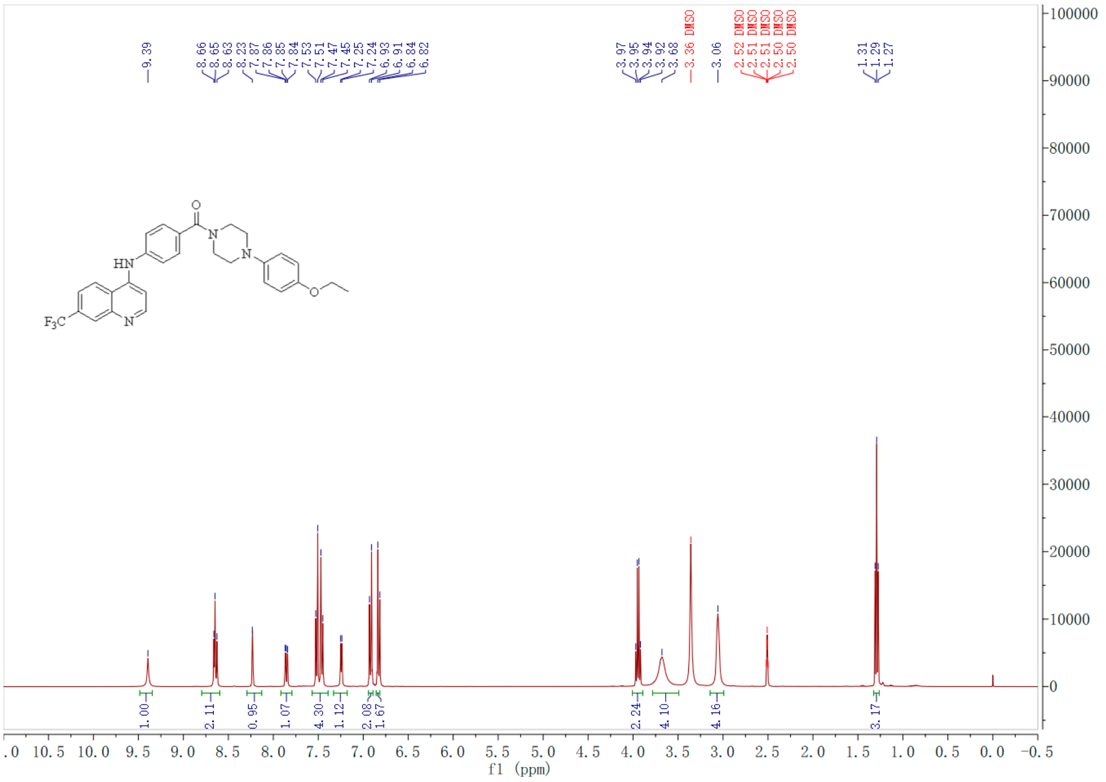

Figure S100. ^{1}H NMR spectra of the target compound **G18**.

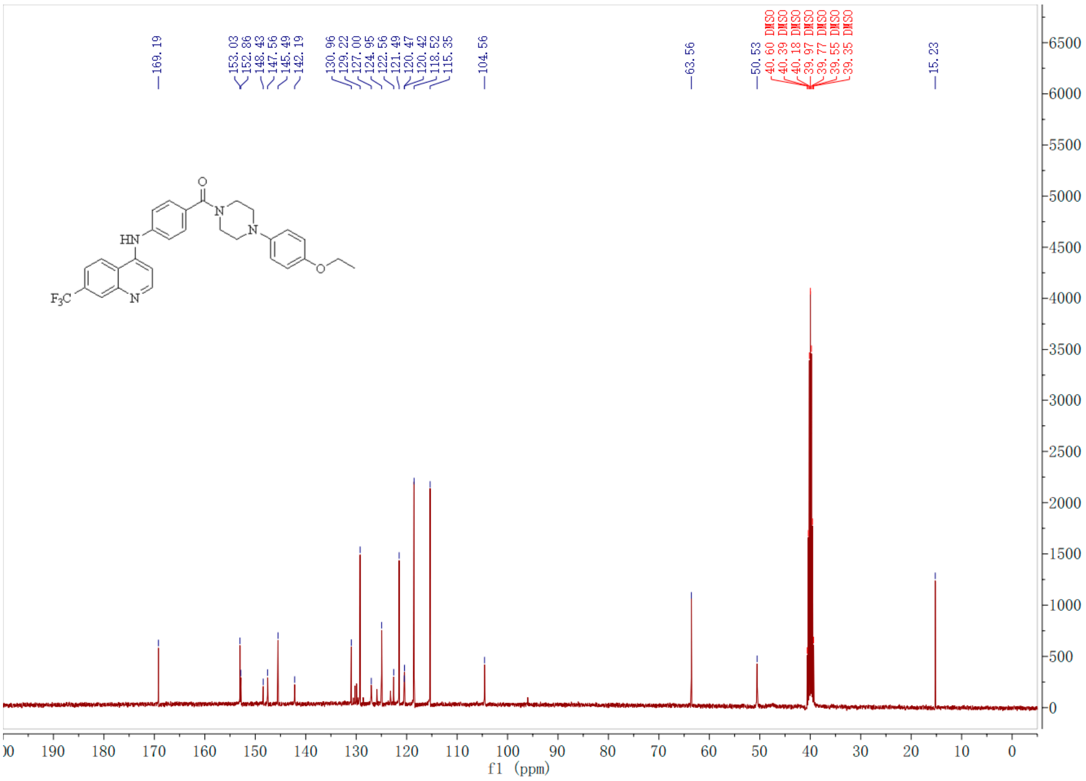

Figure S101. ^{13}C NMR spectra of the target compound **G18**.

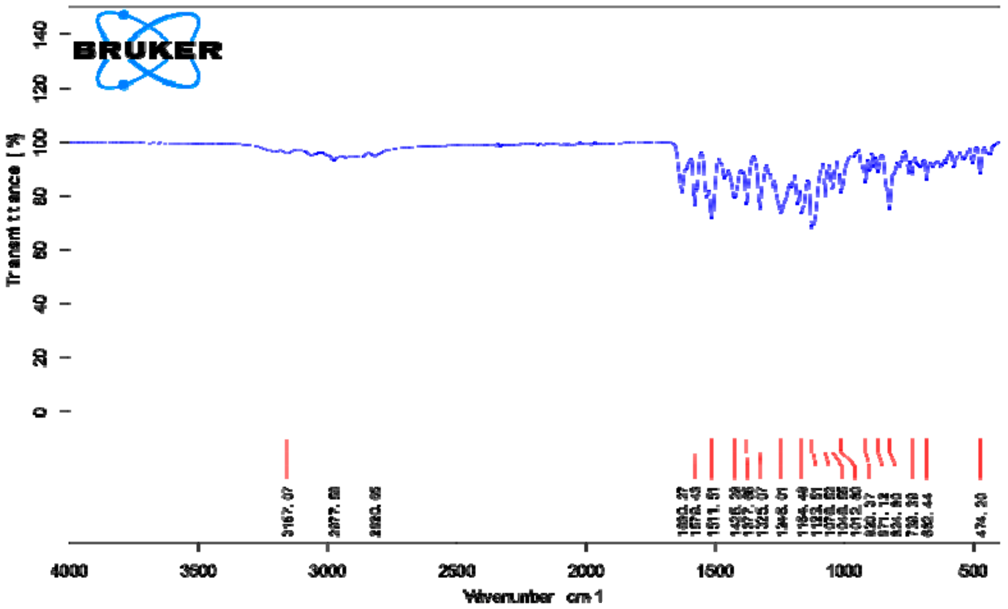

Figure S102. IR spectra of the target compound **G18**.

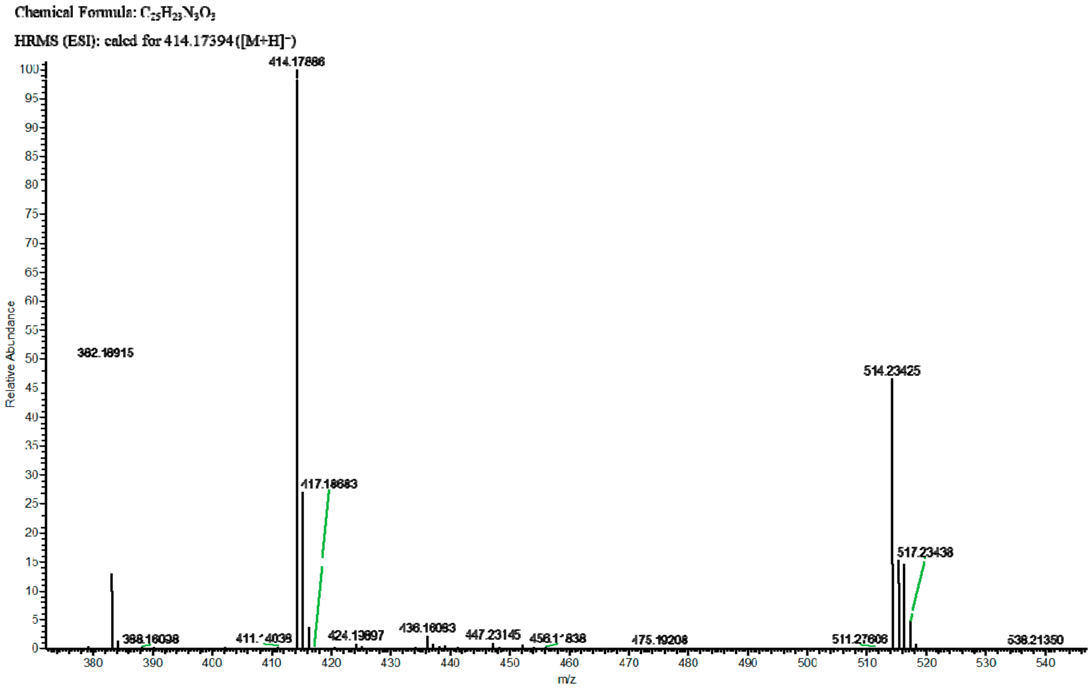

Figure S103. HRMS (ESI) of the target compound **G19**.

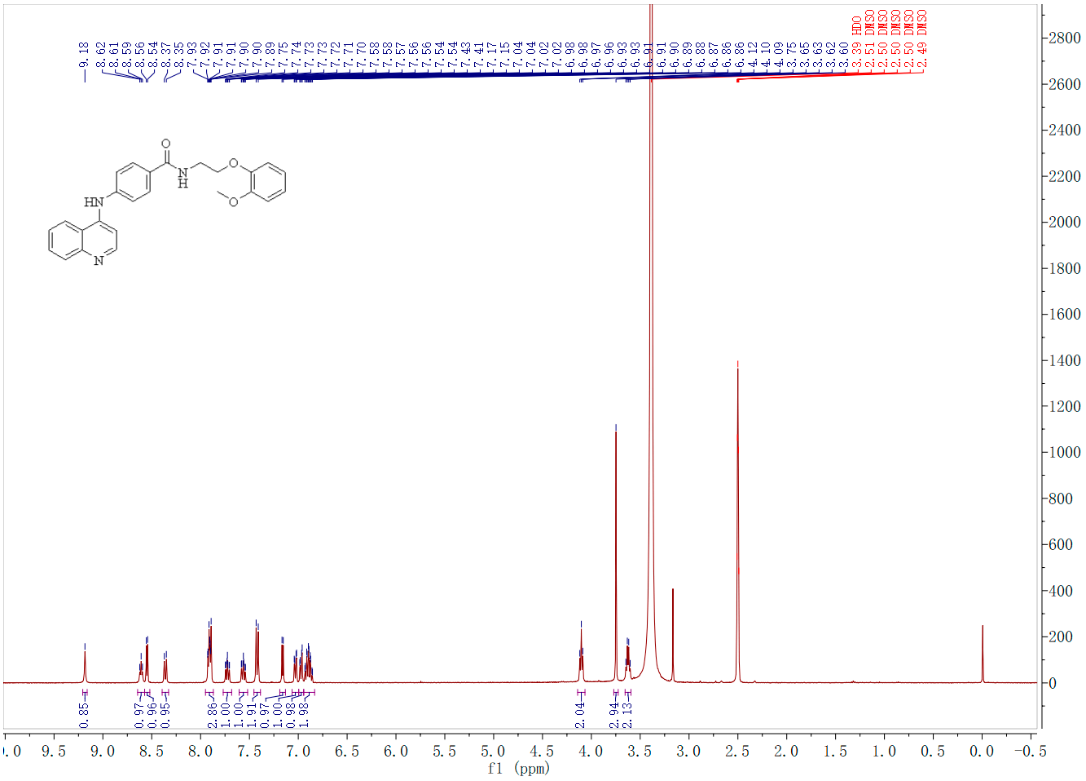

Figure S104. ^{1}H NMR spectra of the target compound **G19**.

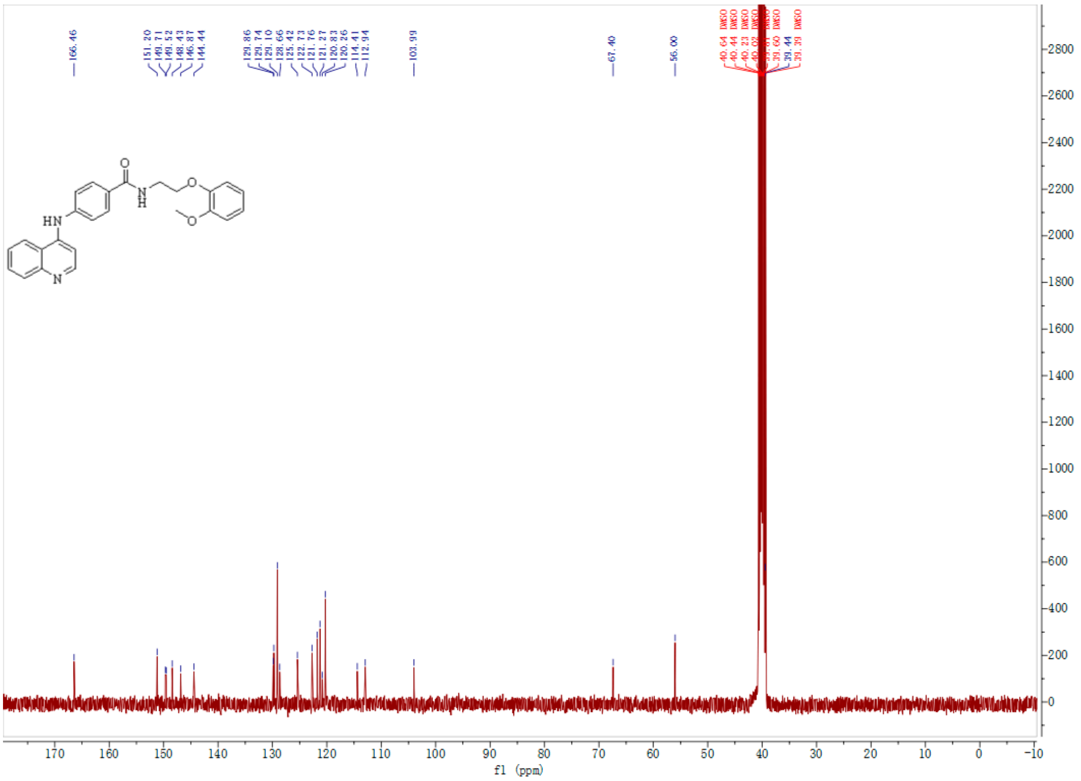

Figure S105. ^{13}C NMR spectra of the target compound **G19**.

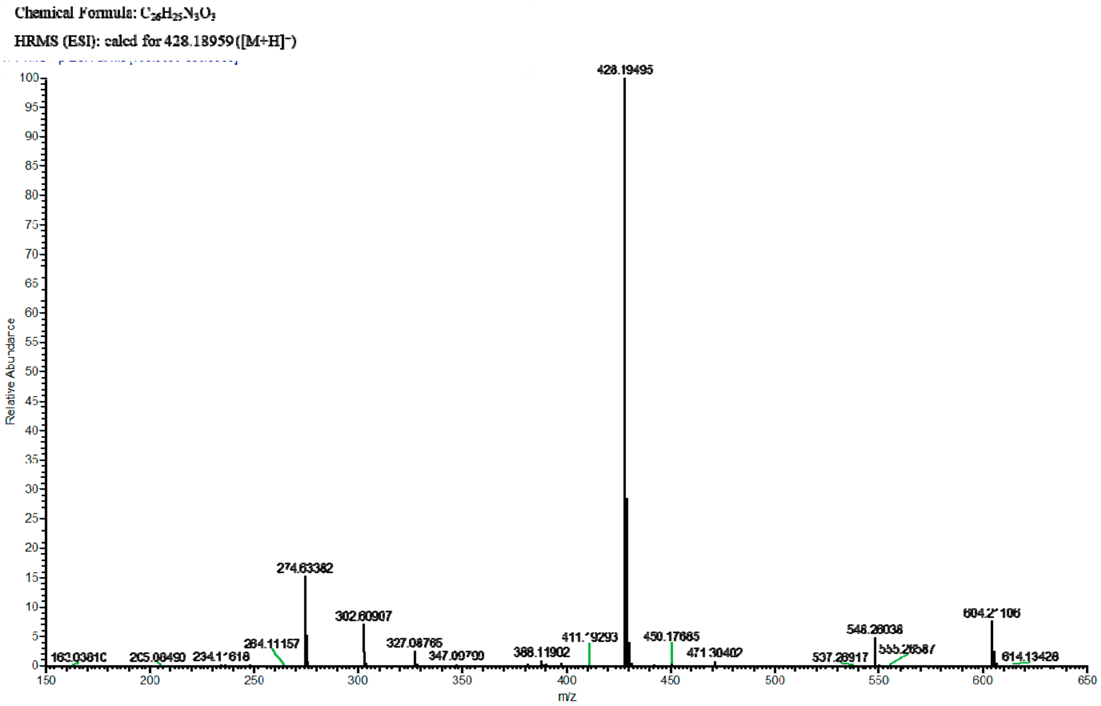

Figure S106. HRMS (ESI) of the target compound **G20**.

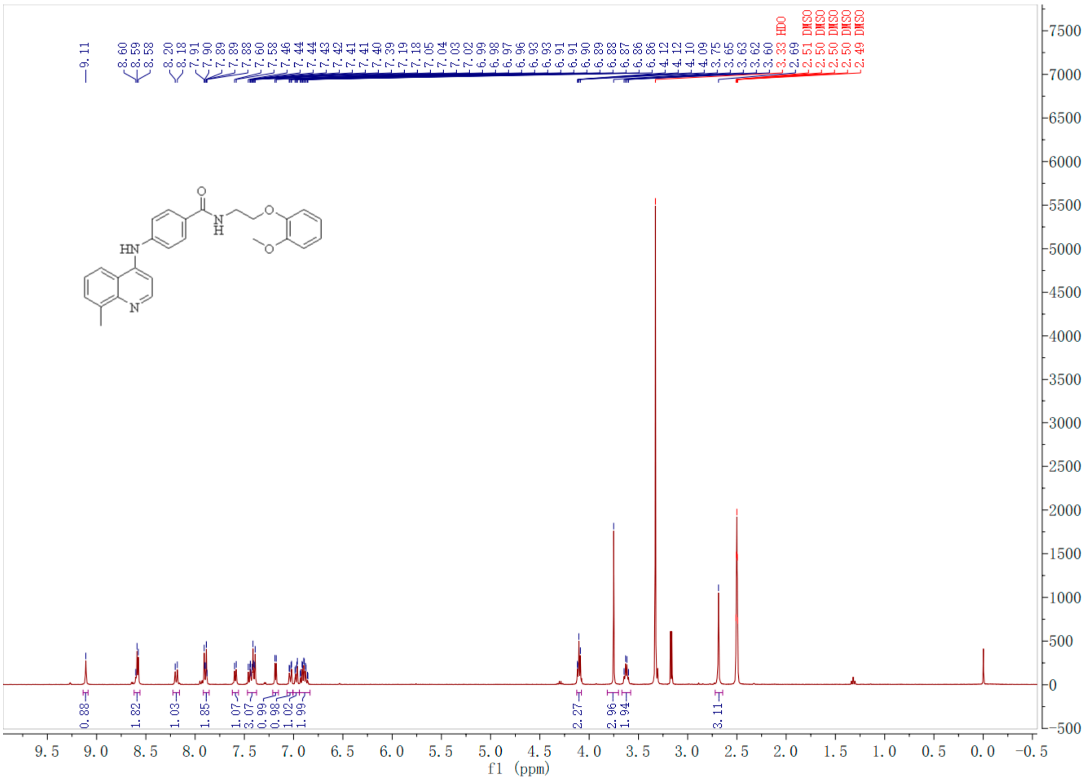

Figure S107. ^{1}H NMR spectra of the target compound **G20**.

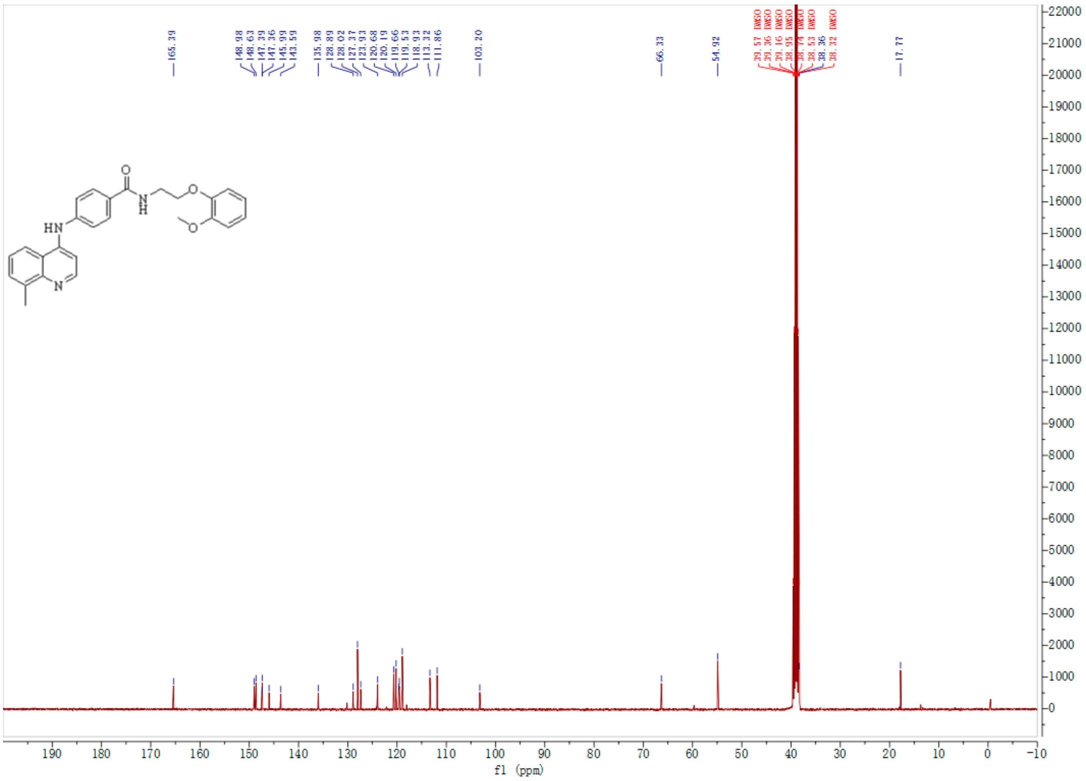

Figure S108. ^{13}C NMR spectra of the target compound **G20**.

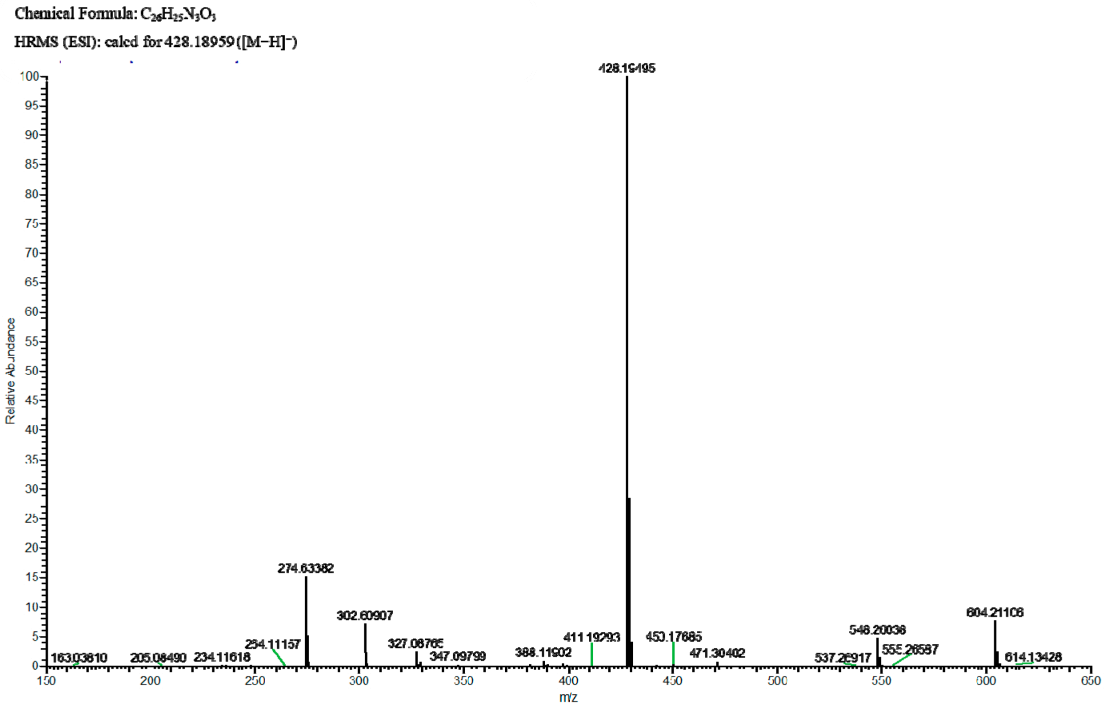

Figure S109. HRMS (ESI) of the target compound **G21**.

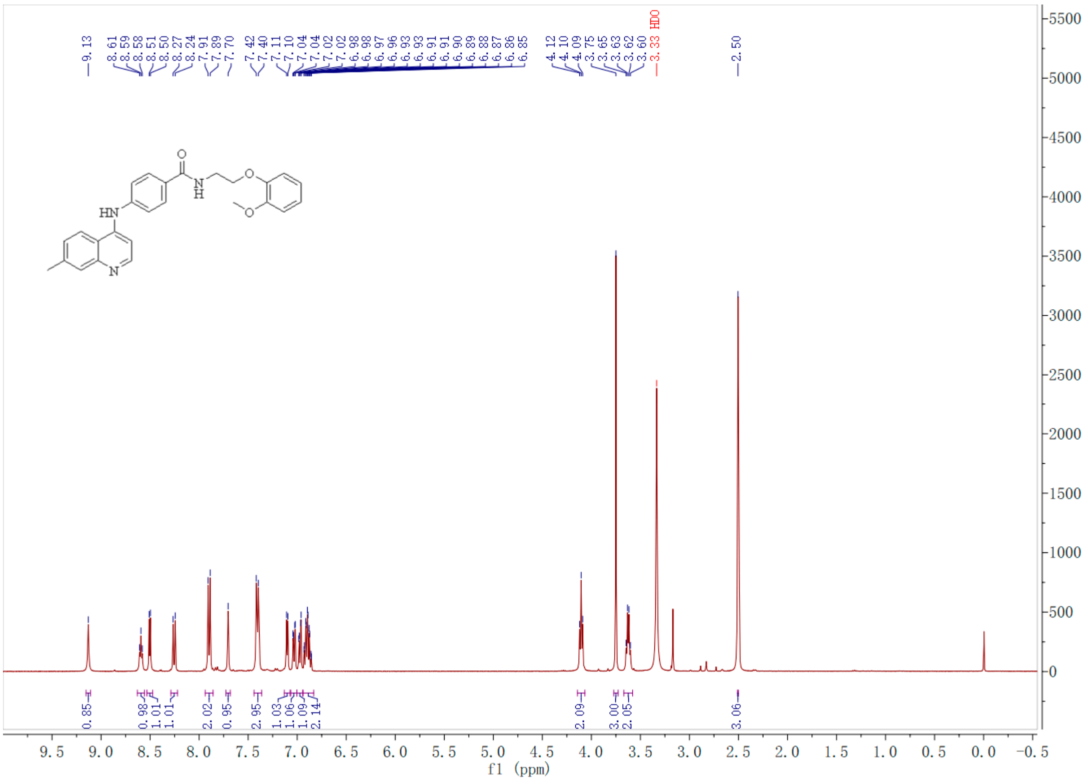

Figure S110. ^{1}H NMR spectra of the target compound **G21**.

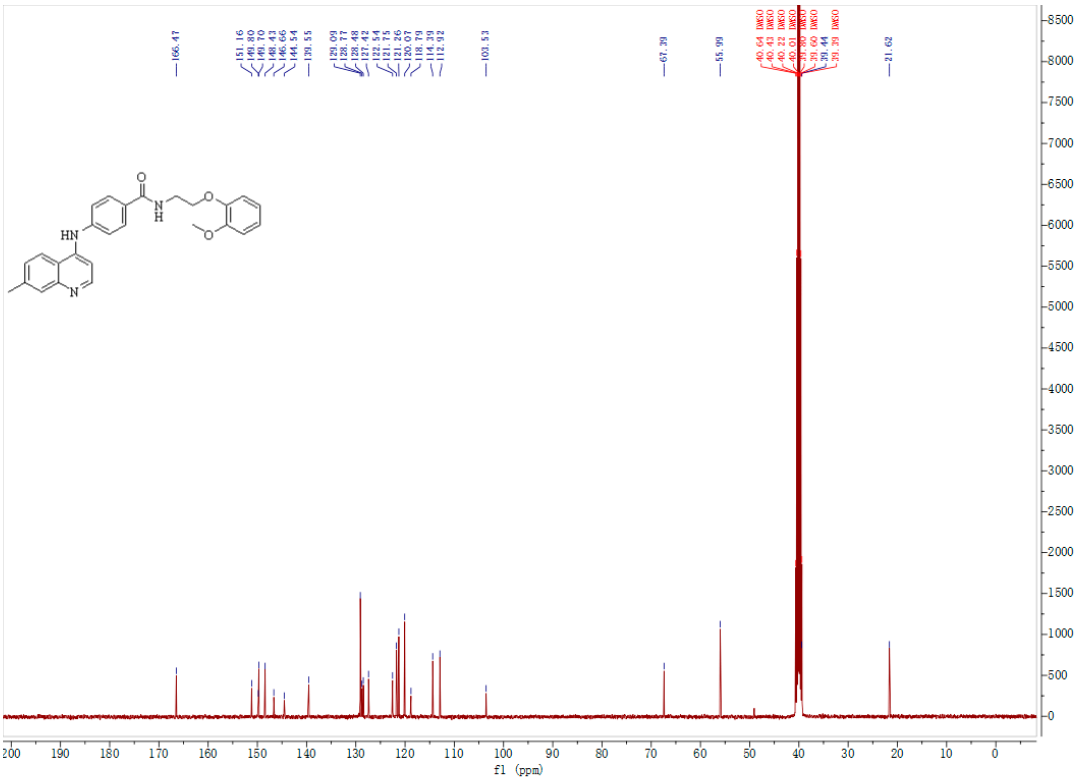

Figure S111. ^{13}C NMR spectra of the target compound **G21**.

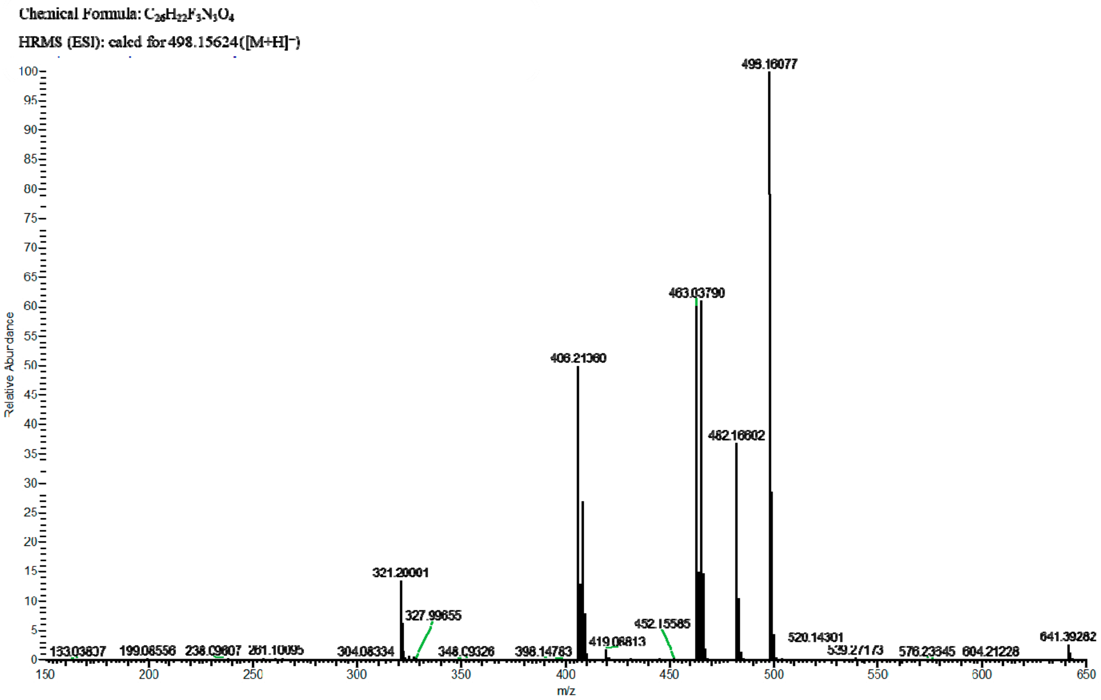

Figure S112. HRMS (ESI) of the target compound **G22**.

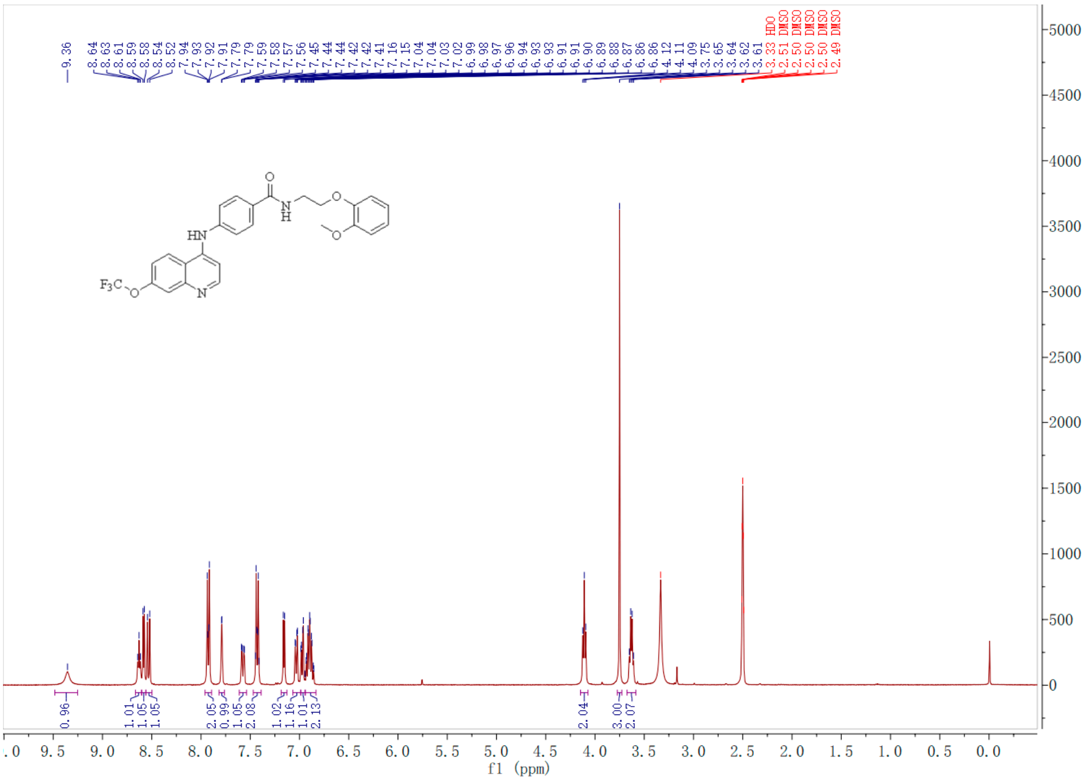

Figure S113. ^{1}H NMR spectra of the target compound **G22**.

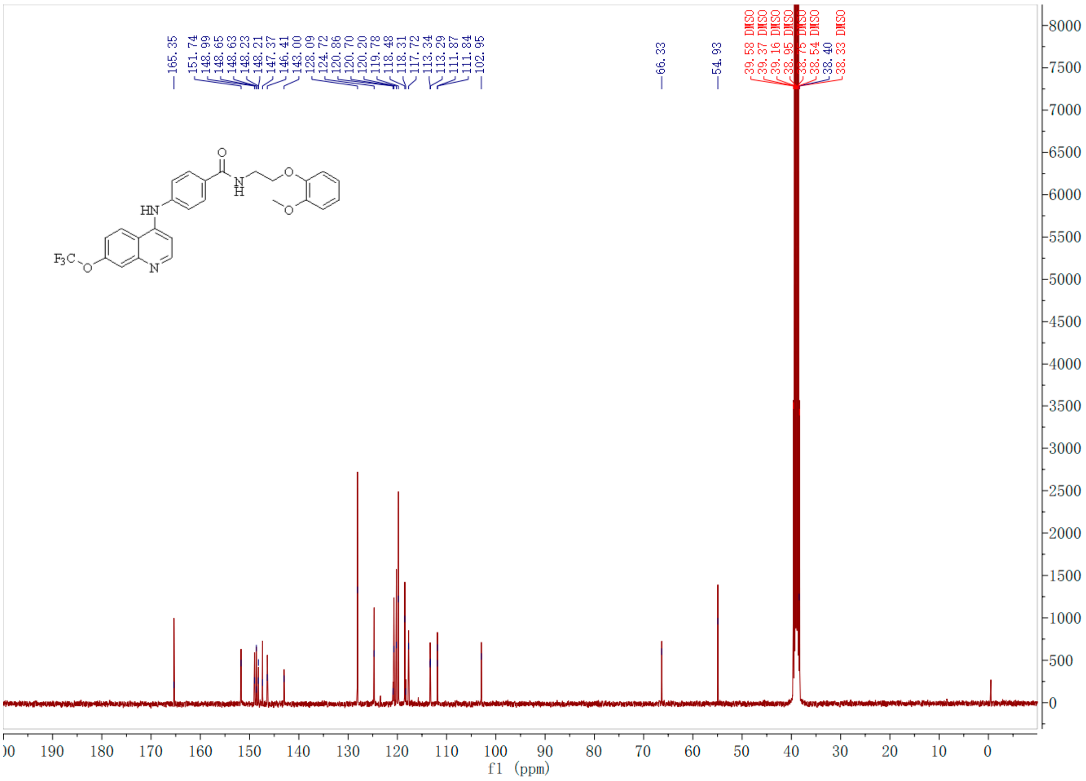

Figure S114. ^{13}C NMR spectra of the target compound **G22**.

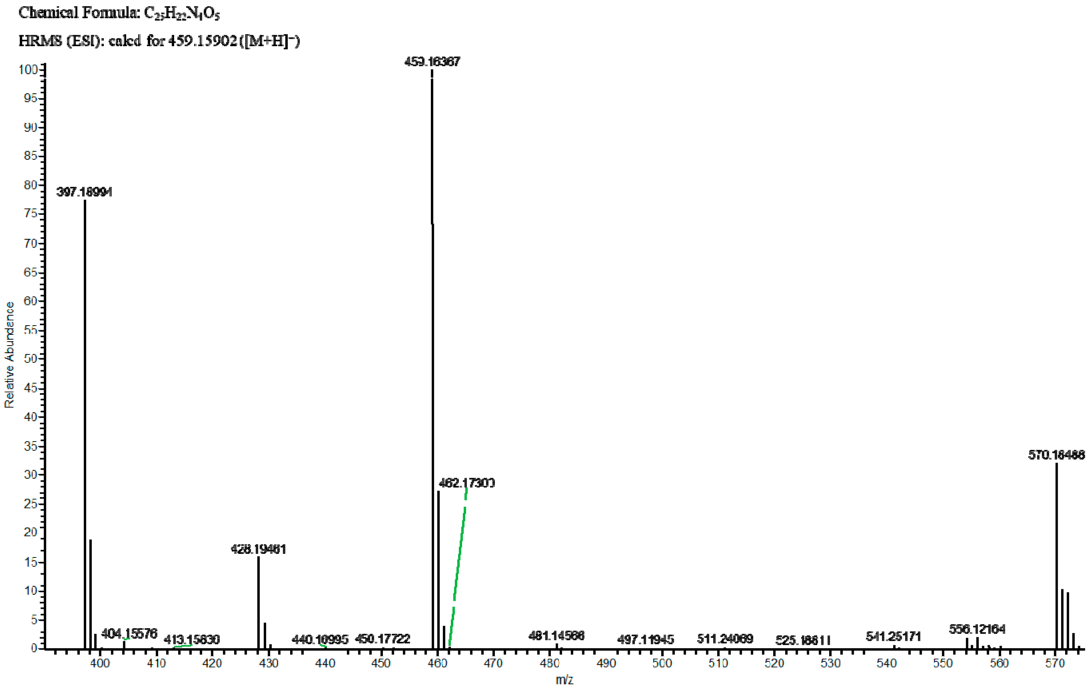

Figure S115. HRMS (ESI) of the target compound **G23**.

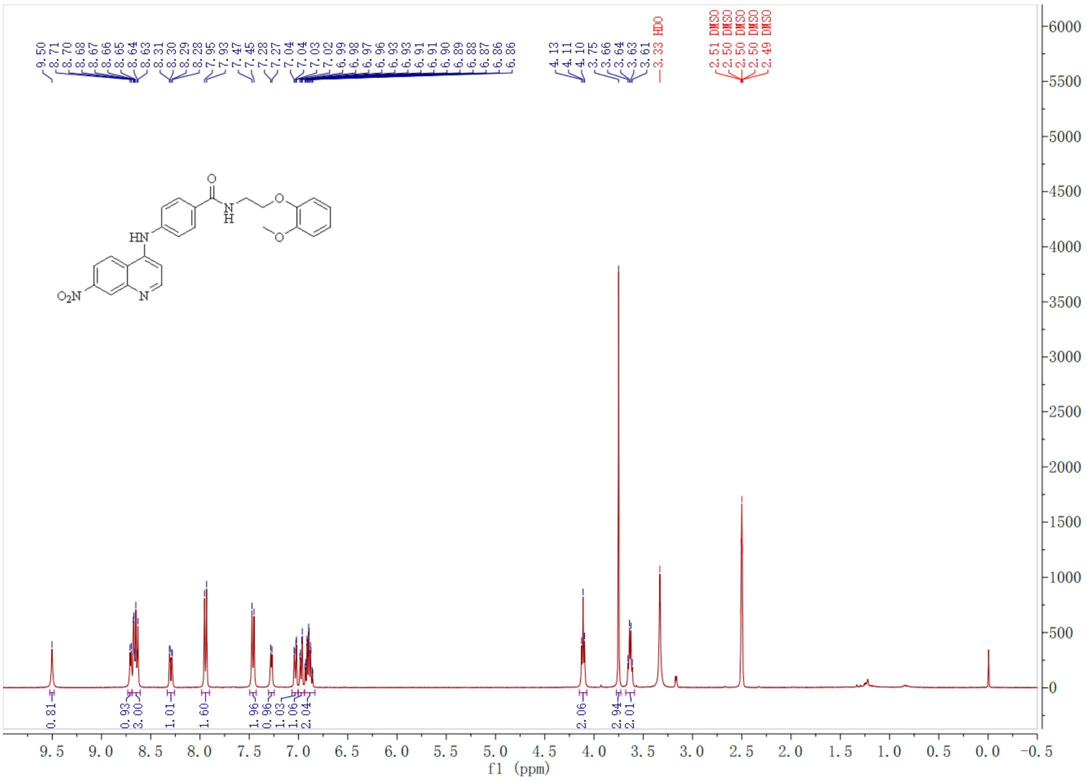

Figure S116. ^{1}H NMR spectra of the target compound **G23**.

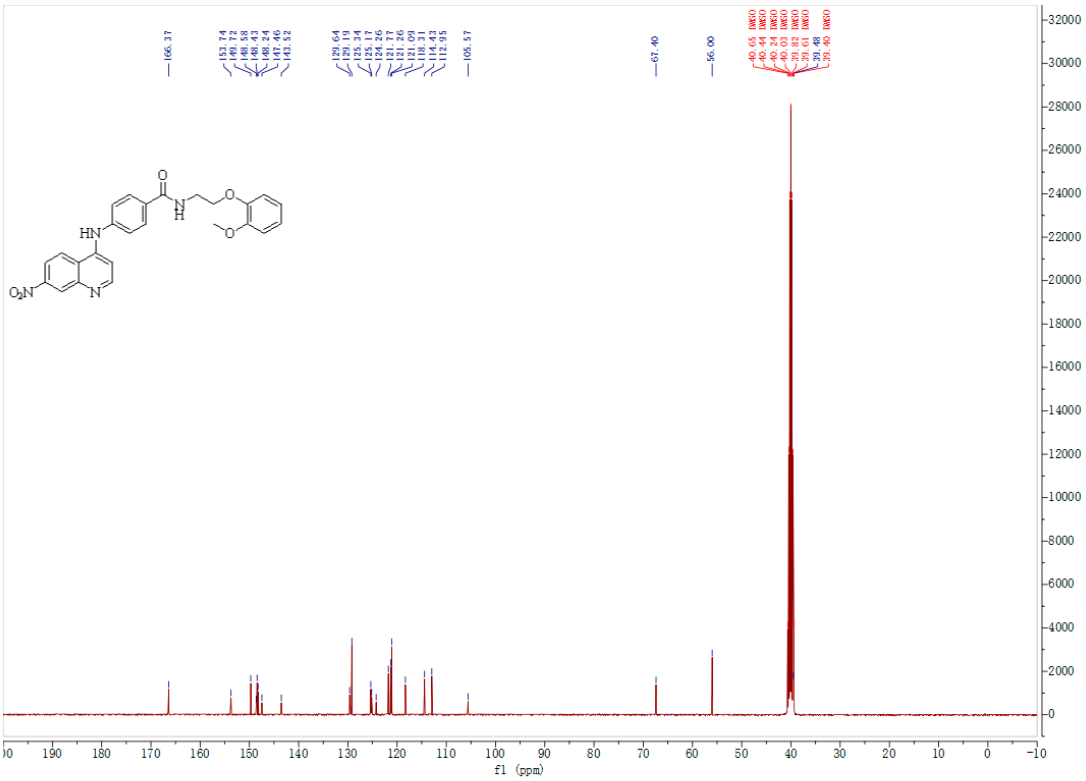

Figure S117. ^{13}C NMR spectra of the target compound **G23**.

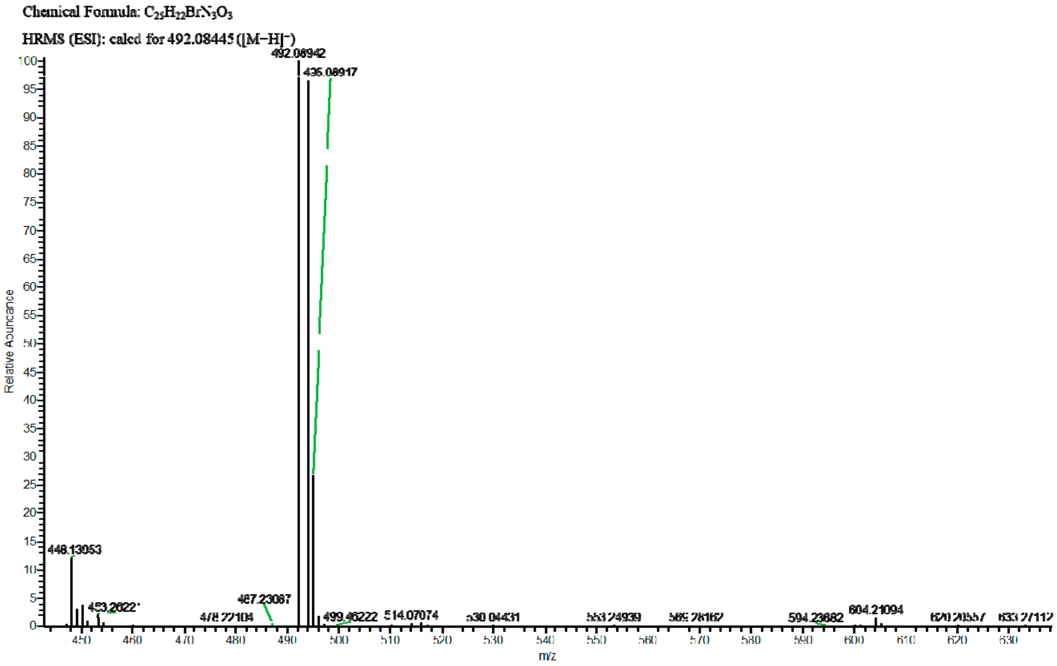

Figure S118. HRMS (ESI) of the target compound **G24**.

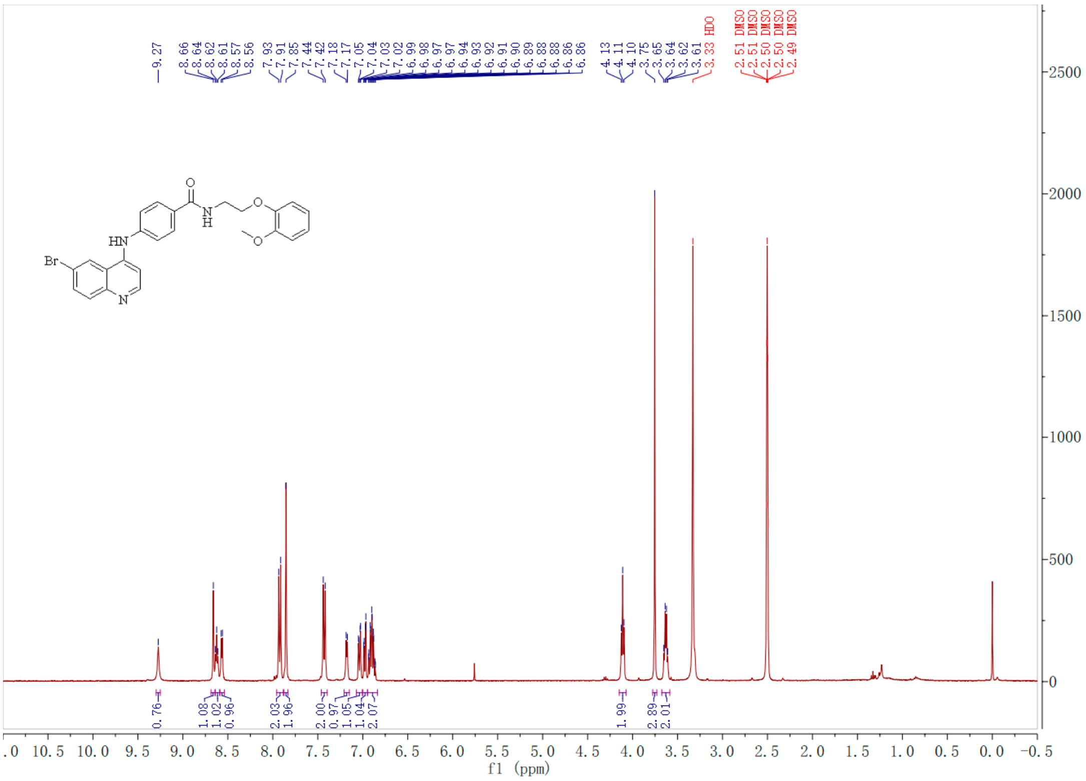

Figure S119. ^{1}H NMR spectra of the target compound **G24**.

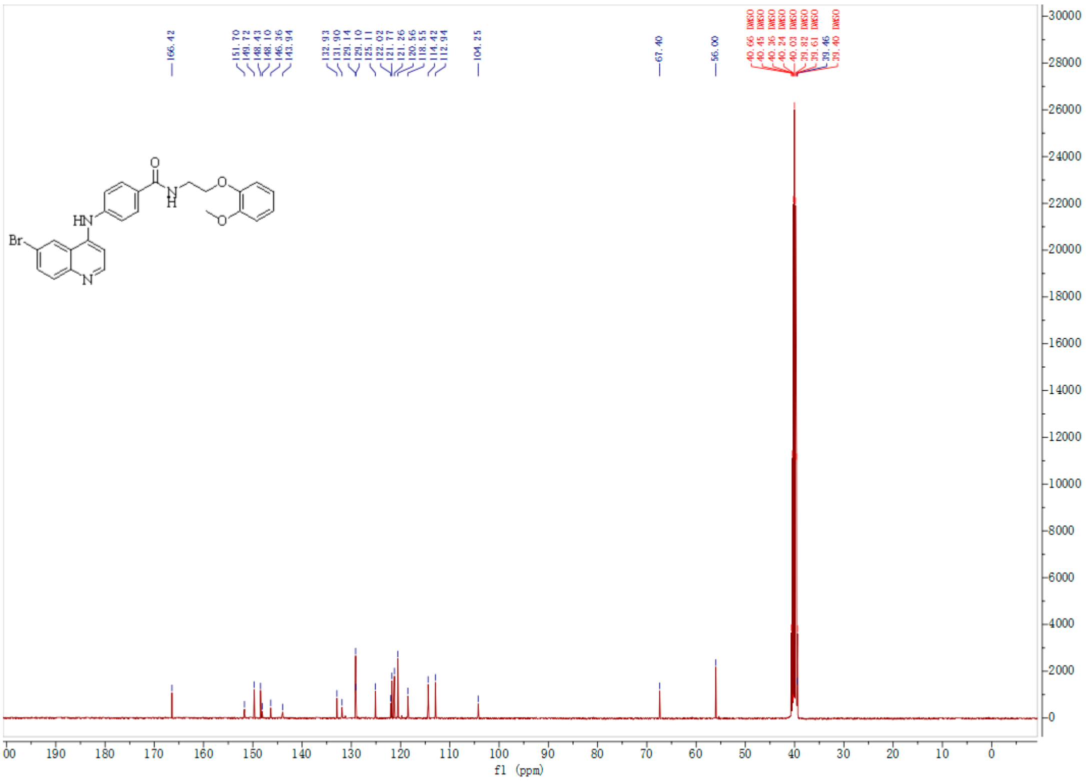

Figure S120. ^{13}C NMR spectra of the target compound **G24**.

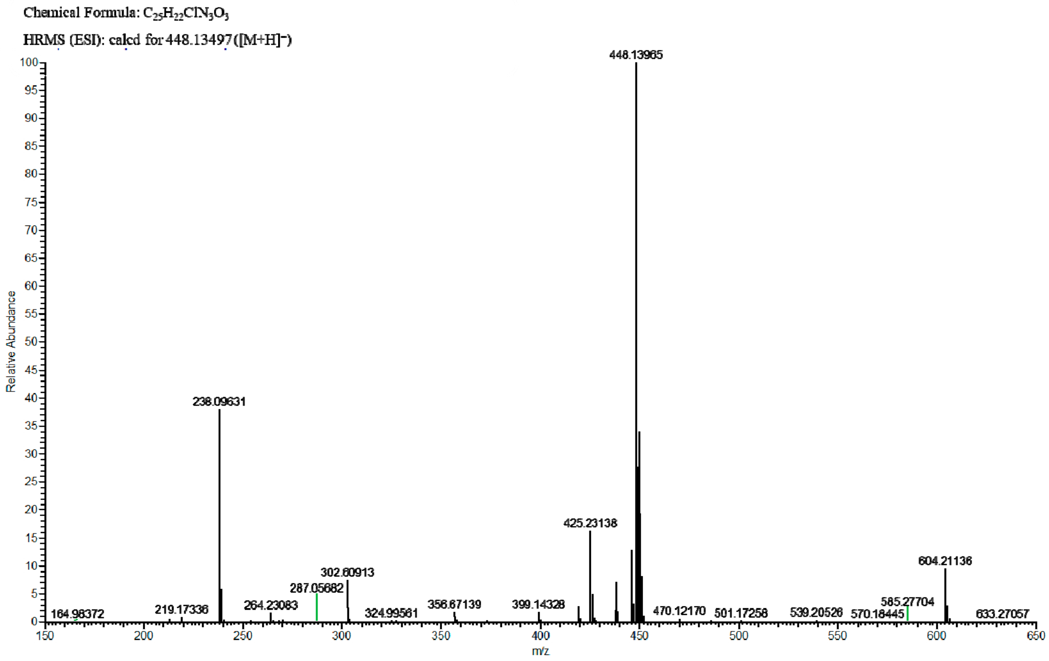

Figure S121. HRMS (ESI) of the target compound **G25**.

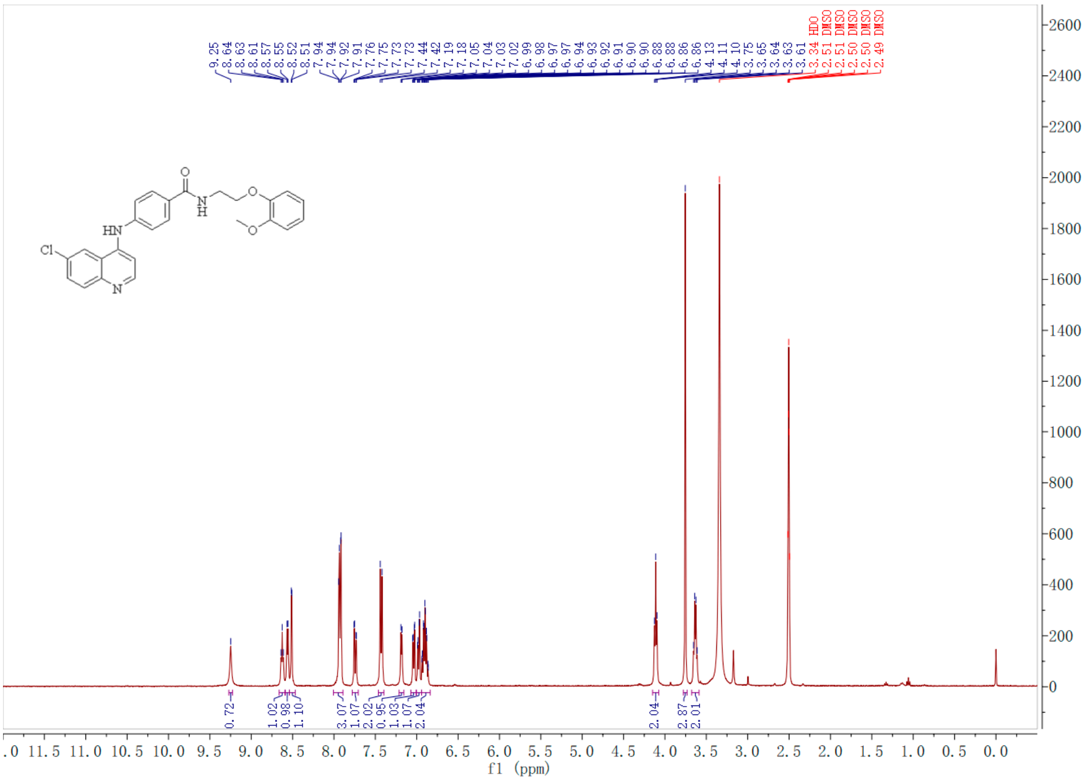

Figure S122. ^{1}H NMR spectra of the target compound **G25**.

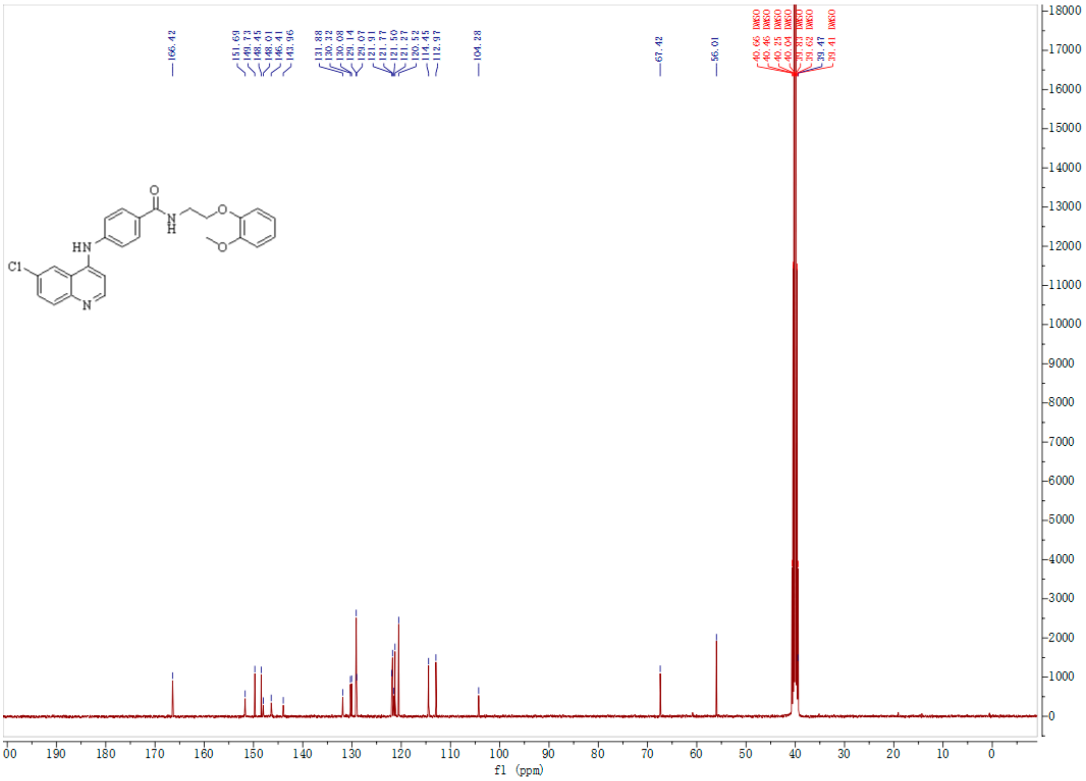

Figure S123. ^{13}C NMR spectra of the target compound **G25**.

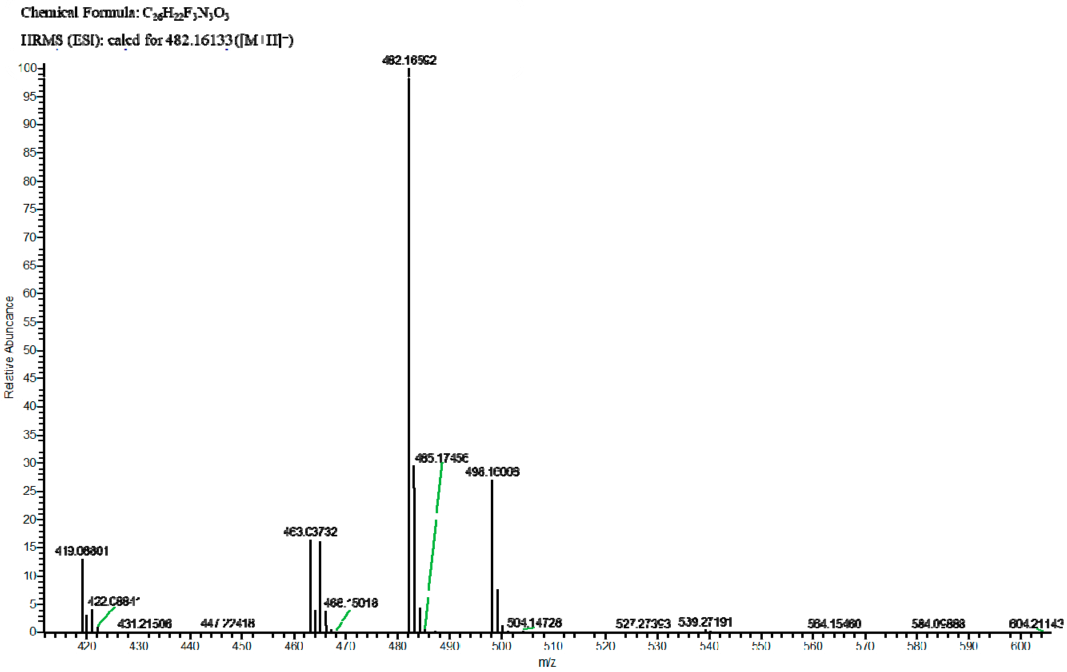

Figure S124. HRMS (ESI) of the target compound **G26**.

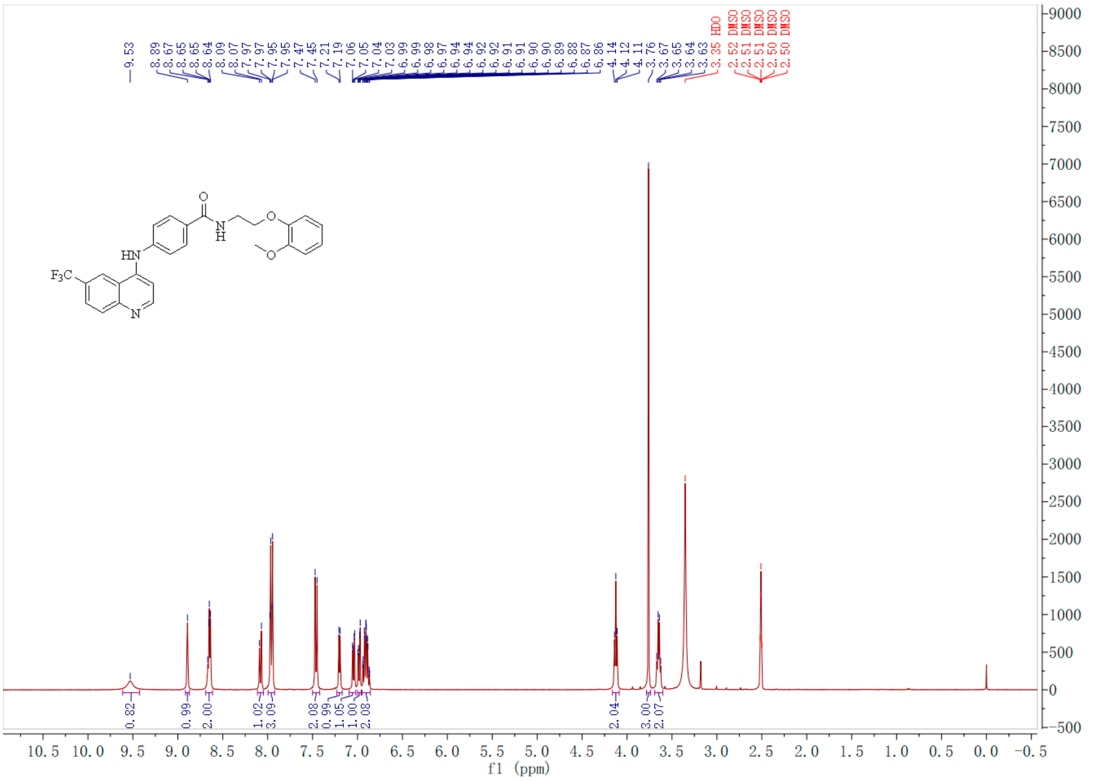

Figure S125. ^{1}H NMR spectra of the target compound **G26**.

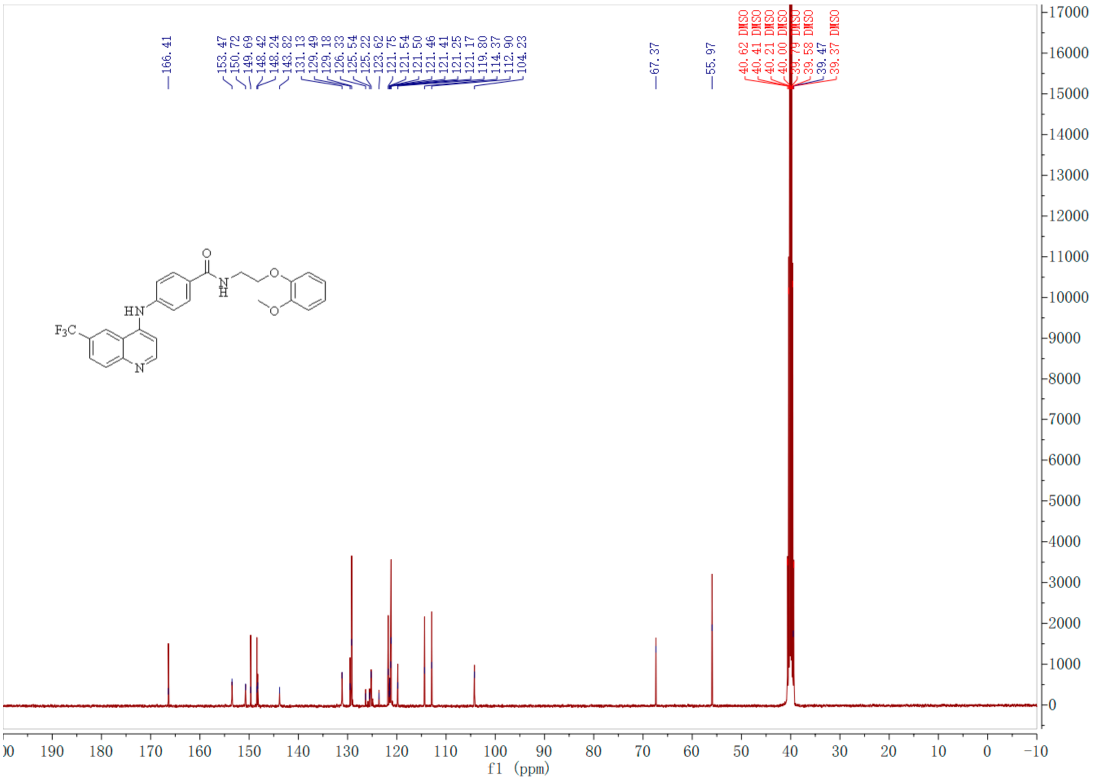

Figure S126. ^{13}C NMR spectra of the target compound **G26**.

References

1. Massari, S.; Bertagnin, C.; Pismataro, M.C.; Donnadio, A.; Nannetti, G.; Felicetti, T.; Bona, S.D.; Nizi, M.G.; Tensi, L.; Manfroni, G.; Loza, M.I.; Sabatini, S.; Cecchetti, V.; Brea, J.; Goracci, L.; Loregian, A.; Tabarrini, O. Synthesis and characterization of 1,2,4-triazolo[1,5-a]pyrimidine-2-carboxamide-based compounds targeting the PA-PB1 interface of influenza A virus polymerase. *Eur. J. Med. Chem.* **2021**, 209, 112944. https://doi.org/10.1016/j.ejmech.2020.112944.
2. D'Agostino, I.; Giacchello, I.; Nannetti, G.; Fallacara, A.L.; Deodato, D.; Musumeci, F.; Grossi, G.; Palu, G.; Cau, Y.; Trist, I.M.; Loregian, A.; Schenone, S.; Botta, M. Synthesis and biological evaluation of a library of hybrid derivatives as inhibitors of influenza virus PA-PB1 interaction. *Eur. J. Med. Chem.* **2018**, 157, 743–758. https://doi.org/10.1016/j.ejmech.2018.08.032.
3. Desantis, J.; Nannetti, G.; Massari, S.; Barreca, M.L.; Manfroni, G.; Cecchetti, V.; Palù, G.; Goracci, L.; Loregian, A.; Tabarrini, O. Exploring the cycloheptathiophene-3-carboxamide scaffold to disrupt the interactions of the influenza polymerase subunits and obtain potent anti-influenza activity. *Eur. J. Med. Chem.* **2017**, 138, 128–139. https://doi.org/10.1016/j.ejmech.2017.06.015.
4. Massari, S.; Goracci, L.; Desantis, J.; Tabarrini, O. Polymerase acidic protein–basic protein 1 (PA–PB1) protein–protein interaction as a target for next-generation anti-influenza therapeutics. *J. Med. Chem.* **2016**, 59(17), 7699–7718. https://doi.org/10.1021/acs.jmedchem.5b01474.
5. Massari, S.; Nannetti, G.; Desantis, J.; Muratore, G.; Sabatini, S.; Manfroni, G.; Mercorelli, B.; Cecchetti, V.; Palu, G.; Cruciani, G.; Loregian, A.; Goracci, L.; Tabarrini, O. A broad anti-influenza hybrid small molecule that potently disrupts the interaction of polymerase acidic protein–basic protein 1 (PA-PB1) subunits. *J. Med. Chem.* **2015**, 58(9), 3830–3842. https://doi.org/10.1021/acs.jmedchem.5b00012.
6. Lepri, S.; Nannetti, G.; Muratore, G.; Cruciani, G.; Ruzziconi, R.; Mercorelli, B.; Palu, G.; Loregian, A.; Goracci, L. Optimization of small-molecule inhibitors of influenza virus polymerase: from thiophene-3-carboxamide to polyamido scaffolds. *J. Med. Chem.* **2014**, 57(10), 4337–4350. https://doi.org/10.1021/jm500300r.
7. Tintori, C.; Laurenzana, I.; Fallacara, A.L.; Kessler, U.; Pilger, B.; Stergiou, L.; Botta, M. High-throughput docking for the identification of new influenza A virus polymerase inhibitors targeting the PA–PB1 protein–protein interaction. *Bioorg. Med. Chem. Lett.* **2014**, 24(1), 280–282. https://doi.org/10.1016/j.bmcl.2013.11.019.
8. Yesselman, J.D.; Price, D.J.; Knight, J.L.; Brooks, C.L. MATCH: an atom-typing toolset for molecular mechanics force fields. *J. Comput. Chem.* **2012**, 33(2), 189–202. https://doi.org/10.1002/jcc.21963.
9. Krovat, E.M.; Langer, T. Non-peptide angiotensin II receptor antagonists: chemical feature based pharmacophore identification. *J. Med. Chem.* **2003**, 46, 716-726. https://doi.org/10.1021/jm021032v.
10. Debnath, A.K. Pharmacophore mapping of a series of 2,4-diamino-5-deazapteridine inhibitors of mycobacterium avium complex dihydrofolate reductase. *J. Med. Chem.* **2002**, 45, 41–53. https://doi.org/10.1021/jm010360c.
11. John, S.; Thangapandian, S.; Arooj, M.; Hong, J.C.; Kim, K.D.; Lee, K.W. Development, evaluation and application of 3D QSAR pharmacophore model in the discovery of potential human renin inhibitors. *BMC Bioinformactics* **2011**, 12(S14), S4. https://doi.org/10.1186/1471-2105-12-S14-S4.
12. Jiang, Y.Y.; Gao, H.W. Pharmacophore-based drug design for the identification of novel butyrylcholinesterase inhibitors against alzheimer's disease. *Phytomedicine* **2019**, 54, 278–290. https://doi.org/10.1016/j.phymed.2018.09.199.
